# Genetic ablation of neuronal mitochondrial calcium uptake impedes Alzheimer’s disease progression

**DOI:** 10.1038/s44318-026-00809-w

**Published:** 2026-05-22

**Authors:** Pooja Jadiya, Elena Berezhnaya, Devin W Kolmetzky, Dhanendra Tomar, Henry M Cohen, Shatakshi Shukla, Manfred Thomas, Salman Khaledi, Joanne F Garbincius, Liam Kennedy, Oniel Salik, Darpan Raghav, Alycia N Hildebrand, John W Elrod

**Affiliations:** 1 Aging + Cardiovascular Discovery Center, Department of Cardiovascular Sciences, Lewis Katz School of Medicine at Temple University, Philadelphia, PA USA; 2 Department of Internal Medicine, Wake Forest University School of Medicine, Winston-Salem, NC USA

**Keywords:** Autophagy & Cell Death, Neuroscience, Organelles

## Abstract

Loss of _m_Ca^2+^ efflux capacity contributes to the pathogenesis and progression of Alzheimer’s disease (AD) by promoting mitochondrial Ca^2+^ (_m_Ca^2+^) overload. Here, we utilized loss-of-function genetic mouse models to causally evaluate the role of _m_Ca^2+^ uptake by conditionally deleting the mitochondrial calcium uniporter channel (mtCU) in a robust mouse model of AD. Loss of neuronal _m_Ca^2+^ uptake reduced Aβ and tau-pathology, synaptic dysfunction, and cognitive decline in 3xTg-AD mice. Knockdown of *Mcu* in an in vitro model of AD significantly reduced matrix Ca^2+^ content, redox imbalance, and mitochondrial dysfunction. The preservation of mitochondrial function rescued the AD-dependent decline in autophagic capacity and protected neurons against amyloidosis and cell death. This was corroborated by in vivo data showing improved mitochondrial structure and apposition in AD mice with loss of neuronal *Mcu*. These results suggest that inhibition of neuronal _m_Ca^2+^ uptake represents a powerful therapeutic target to impede AD progression.

## Synopsis

Alzheimer’s disease is associated with mitochondrial Ca^2+^ (_m_Ca^2+^) overload and dysfunction. This study provides genetic proof that ablation of mitochondrial calcium uniporter (MCU)‑dependent _m_Ca^2+^ uptake reduces amyloid‑β and tau pathology, preserves synaptic integrity, and prevents cognitive decline.

- Neuron‑specific *Mcu* deletion prevents age‑associated cognitive decline in 3xTg‑AD mice.
- Loss of neuronal _m_Ca^2+^ uptake reduces amyloid‑β accumulation and tau hyperphosphorylation.
- MCU ablation lowers mitochondrial oxidative stress and preserves synaptic protein expression.
- Decreasing _m_Ca^2+^ overload restores autophagic capacity and protects neurons from amyloid‑induced toxicity in vitro.

## Abstract (web summary)

Loss of the mitochondrial calcium uniporter (MCU) in neurons reduces amyloid-β and tau pathology and ameliorates cognitive decline in mouse models of Alzheimer’s disease.

## Graphical abstract

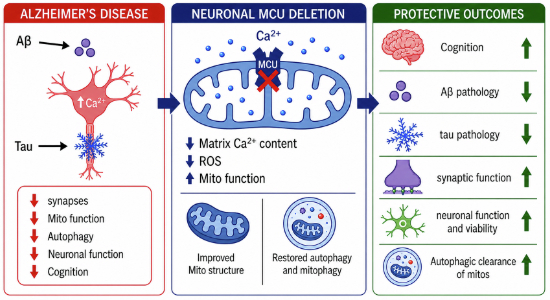

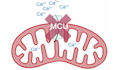

## Introduction

The mechanisms underlying Alzheimer’s disease (AD) pathogenesis remain elusive and this gap in knowledge is a significant barrier to the development of effective therapeutic interventions. AD is associated with mitochondrial dysfunction (Du et al, 2008; Gillardon et al, 2007; Rossi et al, 2020) and altered neuronal intracellular calcium (_i_Ca^2+^) homeostasis(Begley et al, 1999; Mattson et al, 1992). Many studies point to increased [_i_Ca^2+^] as an upstream mechanism that may promote β-amyloid (Aβ) and tau pathology (Barrett et al, 2014; Kipanyula et al, 2012; Verma et al, 2017). Excessive _i_Ca^2+^ can result from the dysregulation of Ca^2+^ channels, such as calcium homeostasis modulator 1 (CALHM1) (Dreses-Werringloer et al, 2008), amino-3-hydroxy-5-methyl-4-isoxazolepropionic acid receptors (AMPAR) (Chang et al, 2006), or N-methyl-D-aspartate receptors (NMDAR) (Mattson et al, 1992). Further, alterations in store-operated calcium entry (SOCE) (Tong et al, 2016) and increased ryanodine receptor (RyR) (Paula-Lima et al, 2011) and inositol trisphosphate receptor (IP3R) activity are reported to increase ER Ca^2+^ content in the context of AD (Ferreira et al, 2015). This coupled with reports of increased ER-mitochondria coupling (Area-Gomez et al, 2018; Area-Gomez et al, 2012) suggests that mitochondrial calcium (_m_Ca^2+^) overload may be a critical event in AD progression.

In contrast, other studies suggest that Aβ pathologies are upstream of impaired neuronal _i_Ca^2+^ handling (Calvo-Rodriguez et al, 2019) and mitochondrial dysfunction (Lustbader et al, 2004), leading to cognitive impairment, synaptic dysfunction, and neurodegeneration in AD. For example, mutations in PS1 are reported to cause RyR hyperactivity (Lacampagne et al, 2017) and increased IP3R channel gating (Cheung et al, 2010; Cheung et al, 2008) which leads to elevated_i_Ca^2+^ signaling. The familial AD (FAD) PS1^M146L^ mutant interacts with the IP3R, increasing Ca^2+^ release from the ER, stimulating APP processing (Cheung et al, 2008), and contributing to neuronal pathology. Studies suggest that increased _i_Ca^2+^ signaling through the IP3R-PS1 interaction is a disease-specific mechanism in FAD (Cheung et al, 2010), enhancing the production of reactive oxygen species and contributing to AD pathogenesis (Muller et al, 2011). Similarly, post-translational remodeling (PKA phosphorylation, oxidation, and nitrosylation) of neuronal RyR2 channels in human AD patients and murine models of AD induces ER-Ca^2+^ leak resulting in calpain activation, tau phosphorylation, and cognitive deficits (Lacampagne et al, 2017). Thus, the literature supports the rationale that calcium dysregulation can both precede and follow Aβ and tau pathology.

Elevations in _i_Ca^2+^ are theorized to be rapidly integrated into mitochondria due to the high electromotive force across the inner mitochondrial membrane (IMM) generated by the electron transport chain (Δψ = ~ −160 mv). Given the driving force for _m_Ca^2+^ entry and the highly dynamic nature of [_i_Ca^2+^], neuronal mitochondria require a tightly regulated _m_Ca^2+^ exchange system (Billups and Forsythe, 2002; Cardenas et al, 2010). Ca^2+^ enters the mitochondrial matrix via the mitochondrial calcium uniporter channel complex (mtCU) and modulates key metabolic control points in the TCA cycle (Denton et al, 1972; Denton et al, 1978). It is postulated that _m_Ca^2+^ is the signal that matches mitochondrial energy production to neuronal energy demand. Excessive [_m_Ca^2+^] causes increased oxidative stress, mitochondrial dysfunction, opening of the mitochondrial permeability transition pore (mPTP), and ultimately neuronal death (Du et al, 2008; Luongo et al, 2015; Nakagawa et al, 2000; Szalai et al, 1999). We recently reported that _m_Ca^2+^ overload due to decreased expression of NCLX, the mitochondrial sodium-calcium exchanger and primary mechanism for _m_Ca^2+^ efflux, contributes to AD progression in the 3xTg-AD mouse model and rescue of NCLX expression was sufficient to abrogate behavioral and histopathological hallmarks of AD (Jadiya et al, 2019). Additionally, recent work from our group as well as others has demonstrated that mitochondrial protein TMEM65 is required for NCLX-dependent _m_Ca^2+^ efflux and that loss of TMEM65 causes lethal neuromuscular deficits in mice (Garbincius et al, 2025; Zhang et al, 2026). We also noted proteomic remodeling of the mtCU in sporadic AD patients (Jadiya et al, 2019). In support of our findings, a recent study showed increased neuronal _m_Ca^2+^ levels correlated with plaque deposition in the APP/PS1-Tg mouse model and other reports have implicated _m_Ca^2+^ overload in the activation of cell death and neurodegeneration (Granatiero et al, 2019; Jadiya et al, 2019; Kostic et al, 2015; Logan et al, 2014; Luongo et al, 2015; Qiu et al, 2013). For example, deletion of cyclophilin D (CypD), a necessary activator of the mPTP, has been shown to prevent mitochondrial dysfunction and memory impairments in AD models (Du et al, 2008). Further, a recent study utilizing a *C. elegans* AD model, *sel-12* (homolog of presenilin) mutant, observed increased ER-mitochondria Ca^2+^ signaling resulting in increased ROS (reactive oxygen species) generation and neuronal dysfunction (Sarasija et al, 2018). Similarly, an AD-linked presenilin mutation (PS1) was reported to increase inositol triphosphate (IP3)-mediated Ca^2+^ transients (Stutzmann et al, 2004) and ER-mitochondrial contacts in AD (Area-Gomez et al, 2012), both of which could contribute to _m_Ca^2+^ overload. These studies strengthen the notion that _m_Ca^2+^ overload is central to AD progression.

*Mitochondrial calcium uniporter* (*MCU*) encodes the pore-forming component of the mtCU complex and is required for channel function (Baughman et al, 2011; De Stefani et al, 2011). Fibroblasts isolated from AD patients display a significant increase in MCU protein expression compared to controls (Perez et al, 2018). Notably, overexpression of MCU by stereotaxic injection of adenovirus into the cortex of mice resulted in increased mitochondrial Ca^2+^ levels, neuronal cell death, and gliosis (Granatiero et al, 2019), suggesting that _m_Ca^2+^ overload alone is sufficient to promote brain pathology. To define how changes in _m_Ca^2+^ uptake causally contribute to the development of AD, we generated 3xTg-AD mutant mice with neuronal-specific deletion of *Mcu* (*Mcu*^*fl/fl*^ x Camk2a-Cre x 3xTg-AD). Loss of acute _m_Ca^2+^ uptake prevents cognitive decline and ameliorates proteotoxic hallmarks of disease including Aβ aggregation and tau phosphorylation in 3xTg-AD mice. In an in vitro model of AD we show that inhibition of _m_Ca^2+^ uptake ameliorates mitochondrial dysfunction and ROS generation rescuing autophagy impairments and protects neurons against amyloidosis and cell death. These data demonstrate that targeting _m_Ca^2+^ uptake is a novel therapeutic strategy to impede AD development and progression.

## Results

### Loss of neuronal _m_Ca^2+^ uptake improves AD-associated cognitive deficits

**Figure 1:**
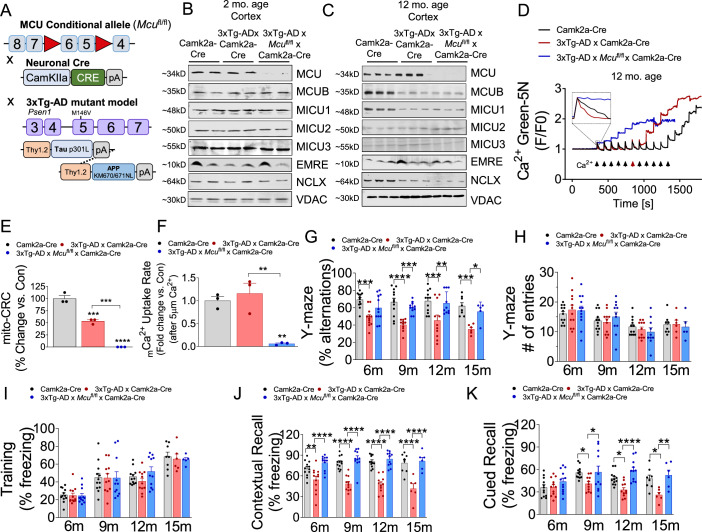
Loss of neuronal _m_Ca^2+^ uptake improves AD-associated cognitive deficits. (**A**) Schematic of *Mcu* knockout 3xTg-AD mutant mouse gene-targeting strategy. (**B**) Western blots for MCU expression and proteins associated with _m_Ca^2+^ exchange in tissue isolated from the cortex of 2-month-old 3xTg-AD x *Mcu*^fl/fl^ x Camk2a-Cre mice compared to age-matched control. MCU, mitochondrial calcium uniporter; MCUB, mitochondrial calcium uniporter β subunit; MICU1, mitochondrial calcium uptake 1; MICU2, mitochondrial calcium uptake 2; MICU3, mitochondrial calcium uptake 3; EMRE, Essential MCU Regulator; NCLX, Mitochondrial Na + /Ca2+ Exchanger. VDAC, Voltage-dependent anion channel served as mitochondrial loading controls. (**C**) Western blots for MCU expression and proteins associated with _m_Ca^2+^ exchange in tissue isolated from the cortex of 12-month- old 3xTg-AD x *Mcu*^fl/fl^ x Camk2a-Cre mutant mice compared to age-matched control. (**D**) Representative traces for _m_Ca^2+^ retention capacity (CRC). (**E**) Percent change in CRC of 3xTg-AD × Camk2a-Cre and 3xTg-AD × *Mcu*^fl/fl^ × Camk2a-Cre vs. Camk2a-Cre control. (**F**) _m_Ca^2+^ uptake rate expressed as fold-change vs. Camk2a-Cre con. calculated after first 5-µm Ca^2+^ bolus. (**G**, **H**) Y-maze spontaneous alternation test. (**G**) Percentage of spontaneous alternation. (**H**) Total number of arm entries. (**I**–**K**) Fear-conditioning test. (**I**) Freezing responses in the training phase. (**J**) Contextual recall freezing responses, (**K**) Cued recall freezing responses. All data presented as mean ± SEM, ****P  <  0.0001, ***P  <  0.001, **P  <  0.01, *P  <  0.05. One-way ANOVA with Sidak’s multiple comparisons test was used to compared data with the following adjusted *P* values shown in the graphs from left to right: (**E**) *P *= 0.0005, *P *= 0.0003, *P* = 0.000007; (**F**) *P* = 0.0042, *P *= 0.0092; (**G**) *P* = 0.0004, *P* = 0.0009, *P* = 0.0001, *P* = 0.0004, *P* = 0.0238; (**J**) *P* = 0.0059, *P* = 0.000007192, *P *= 0.000000073; *P* = 0.0000000008, *P* = 0.000000008, *P* = 0.0000000005, *P* = 0.00000046, *P* = 0.0000003557; (**K**) *P* = 0.03548, *P* = 0.02862, *P* = 0.02727, *P* = 0.00008, *P* = 0.01393, *P* = 0.00411. *n* = individual dots shown for each group in all graphs. For (**G**, **H**), *n* (Camk2a-Cre) = 12, 12, 12, 8 (6, 9, 12, 15 months), *n* (3xTg-AD × Camk2a-Cre) = 12, 11, 11, 6 (6, 9, 12, 15 months), *n* (3xTg-AD × *Mcu*^fl/fl^ × Camk2a-Cre) = 12, 11, 10, 5 (6, 9, 12, 15 months). For (**I**–**K**), *n* (Camk2a-Cre) = 12, 12, 12, 8 (6, 9, 12, 15 months), *n* (3xTg-AD × Camk2a-Cre) = 12, 11, 11, 6 (6, 9, 12, 15 months), *n* (3xTg-AD × *Mcu*^fl/fl^ × Camk2a-Cre) = 12, 11, 11, 5 (6, 9, 12, 15 months). Source data are available online for this figure.

**Figure EV1:**
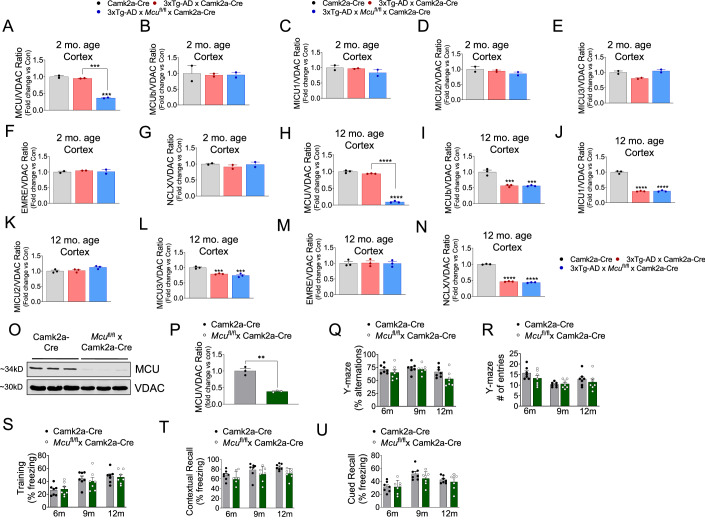
_m_Ca^2+^ exchanger expression and cognitive assay. (**A**–**N**) Quantification of protein expression associated with _m_Ca^2+^ exchange expressed as fold-change vs. Camk2a-Cre con. corrected to a mitochondrial loading control VDAC, in tissue isolated from the brain cortex of 2- and 12-month-old mice, *n* = 2 per group in (**A**–**G**), *n* = 3 per group in (**H**–**N**). (**O, P**) Western blot validation and densitometry analysis for the expression of MCU protein in tissue isolated from the cortex of *Mcu*^fl/fl^ x Camk2a-Cre mice compared to age-matched control corrected to VDAC, *n* = 3. (**Q**, **R**) Y-maze spontaneous alternation test, *n* = 7 per group. (**Q**) Percentage of spontaneous alternation. (**R**) Total number of arm entries. (**S**–**U**) Fear-conditioning test, *n* = 7 per group. (**S**) Freezing responses in the training phase. (**T**) Contextual recall freezing responses, (**U**) Cued recall freezing responses. *n* = individual dots shown for each group in all graphs. All data presented as mean ± SEM, ****P  <  0.0001, ***P  <  0.001, **P  <  0.01, *P  <  0.05. One-way ANOVA with Sidak’s multiple comparisons test with adjusted *P* values shown in the graphs left to right: (**A**) *P* = 0.0006, *P* = 0.0008; (**H**) *P* = 0.00000006, *P* = 0.00000009; (**I**) *P* = 0.00052, *P *= 0.00049; (**J**) *P* = 0.000001, *P* = 0. 000001; (**L**) *P* = 0.0000002, *P* = 0.0000002; (**N**) *P* = 0.000000007, *P* = 0.000000005. To compare data in (**P**) *t* test was used with *P* value of 0.0011. Two-way ANOVA with Sidak’s multiple comparisons test was performed to compare data in (**Q**–**S**). Source data are available online for this figure.

To examine the role of _m_Ca^2+^ uptake in AD pathology, we generated a neuronal-specific knockout of *Mcu* in the 3xTg-AD mutant mouse model. 3xTg-AD mice develop robust disease including both Aβ and tau pathology and memory decline. 3xTg-AD mice were crossed to mice expressing neuron-restricted Cre recombinase (Tsien et al, 1996) harboring the *Mcu* floxed allele (*Mcu*^fl/fl^ x Camk2a-Cre) (Luongo et al, 2015) to delete *Mcu* specifically from frontal cortex and hippocampal neurons in AD mice (3xTg-AD x *Mcu*^fl/fl^ x Camk2a-Cre, Fig. 1A). Loss of MCU expression from the frontal cortex of animals harboring both *Mcu*^*fl/fl*^ and Camk2a-Cre alleles was confirmed at both 2 and 12 months via Western blot, validating the experimental model system (Figs. 1B,C and EV1O,P). No significant changes were observed in the expression of other mitochondrial calcium uniporter channel complex (mtCU)-associated components in the 3xTg-AD x *Mcu*^fl/fl^ x Camk2a-Cre mice compared to 3xTg-AD x Camk2a-Cre mice. However, in 12-month-old mice harboring the 3xTg-AD alleles there was a reduction in MCUB, MICU1, MICU3, and NCLX expression when compared to Camk2a-Cre controls (Figs. 1B,C and EV1A–N). This decrease in the expression of mtCU regulators is likely related to elevated _m_Ca^2+^ levels, which are observed during AD progression and validates reported results in non-familial AD patient samples (Calvo-Rodriguez et al, 2020; Jadiya et al, 2023; Jadiya et al, 2019). Previous studies have shown that homozygous MICU1 deletion in neurons leads to altered Ca^2+^ homeostasis and progressive motor and cognitive dysfunction (Singh et al, 2022). As MICU1 and MICU3 regulate MCU-dependent Ca^2+^ uptake (Ashrafi et al, 2020; Singh et al, 2022), their downregulation in the transgenic AD model suggests that MICU1 and MICU3 deficiency may promote aberrant _m_Ca^2+^ uptake. Next, we examined _m_Ca^2+^ uptake and calcium retention capacity (CRC) using mitochondria isolated from the frontal cortex of 12-month-old mice with MCU deletion in the 3xTg-AD background. 3xTg-AD mice demonstrated reduced CRC with no significant increase in _m_Ca^2+^ uptake rate relative to controls (Fig. 1D–F). Loss of neuronal MCU caused near complete loss of _m_Ca^2+^ uptake and CRC (Fig. 1D–F).

Next, we sought to assess whether loss of neuronal _m_Ca^2+^ uptake was sufficient to reduce or prevent cognitive decline in 3xTg-AD mice. Mice from all groups (Camk2a-Cre, 3xTg-AD x Camk2a-Cre, and 3xTg-AD x *Mcu*^fl/fl^ x Camk2a-Cre) were tested for spatial memory by Y-maze at 6, 9, 12 and 15 months of age. Loss of neuronal MCU significantly improved AD-associated impairments in spatial working memory evidenced by increased alternations from 9-15 months of age (Fig. 1G). Importantly, we did not see any significant differences in locomotor function between groups, as indicated by an equivalent number of arm entries (Fig. 1H).

Next, we used a fear-conditioning paradigm to assess changes in contextual and cued fear conditioning. Freezing behavior was measured in response to either the training environment (contextual) or a tone previously paired with foot shock (cued). No differences in freezing response were observed during the training session between groups throughout the study, demonstrating no measurable effect on locomotor activity or differences in baseline behavior (Fig. 1I). 3xTg-AD mice displayed impairments in contextual and cued recall from 6-15 months and 12–15 months of age, respectively (Fig. 1J,K). AD mice with neuronal deletion of *Mcu* exhibited a significant improvement in contextual and cued recall from 6-15 months of age (Fig. 1J,K). Importantly, loss of neuronal MCU alone (*Mcu*^fl/fl^ x Camk2a-Cre) did not result in cognitive impairment in Y-maze testing (Fig. EV1Q,R) and demonstrated a slight decrease in contextual recall at 12 months of age (Fig. EV1S–U), perhaps suggesting a physiological role for _m_Ca^2+^ uptake in specific neuronal populations involved in long-term amygdala and/or hippocampus memory in aged mice (Curzon et al, 2009).

### Genetic ablation of neuronal _m_Ca^2+^ uptake attenuates Aβ accumulation in 3xTg-AD mice

**Figure 2:**
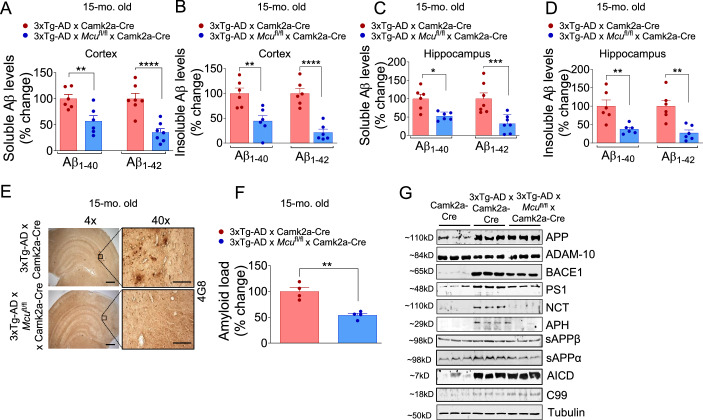
Genetic ablation of neuronal _m_Ca^2+^ uptake attenuates Aβ accumulation in 3xTg-AD mice. (**A**, **B**) Soluble and insoluble Aβ_1–40_ and Aβ_1–42_ levels in the cortex of 15 months old mice, measured by sandwich ELISA, n(Aβ_1–40_) = 6 and n(Aβ_1–42_) = 7 per group in (**A**), *n* = 6 per group in (**B**). (**C**, **D**) Soluble and insoluble Aβ_1–40_ and Aβ_1–42_ levels in the hippocampus of 15-month-old mice, measured by sandwich ELISA. *n* = individual dots shown for each group in all graphs, n(Aβ_1–40_) = 6 and n(Aβ_1–42_) = 7 per group in (**C**) and *n* = 6 per group in (**D**). (**E**) Representative immunohistochemical staining for 4G8-reactive β-amyloid; 4× scale bar = 100 μM, 40× scale bar = 50 μM. (**F**) Quantification of the integrated optical density area for Aβ immunoreactivity, *n*  =  4 for all groups. (**G**) Western blots of full-length APP, ADAM-10, BACE1, PS1, Nicastrin, APH, sAPPα, sAPPβ, AICD, C99 and tubulin (loading control) for cortex homogenate of 15-month- old mice, *n*  =  3 for all groups. All data presented as mean ± SEM, ****P  <  0.0001, ***P  <  0.001, **P  <  0.01, *P  <  0.05. One-way ANOVA with Sidak’s multiple comparisons test with following adjusted *P* values shown in the corresponding graphs left to right: (**A**) *P* = 0.007797, *P* = 0.000059; (**B**) *P* = 0.001444, *P* = 0.000031; (**C**) *P* = 0.020685, *P* = 0.000544; (**D**) *P* = 0.003989, *P* = 0.001029. Unpaired *t* test was done for (**F**) with *P* = 0.0018. Source data are available online for this figure.

**Figure EV2:**
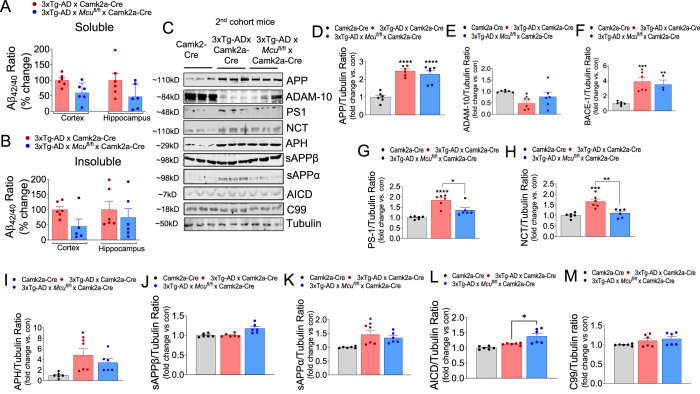
Effect of genetic ablation of neuronal _m_Ca^2+^ uptake on Aβ pathway. (**A**) Soluble Aβ_1–42_/Aβ_1–40_ ratio in cortex and hippocampus of 15-month-old mice, measured by sandwich ELISA, *n* = 6 per group. (**B**) Insoluble Aβ_1–42_/Aβ_1–40_ ratio in cortex and hippocampus of 15-month-old mice, measured by sandwich ELISA, n(3xTg-AD × *Mcu*^fl/fl^ × Camk2a-Cre, cortex) = 5, for other groups *n* = 6. (**C**) Western blots of full-length APP, ADAM-10, PS1, nicastrin, APH, sAPPα, sAPPβ, AICD, C99 and tubulin (loading control) for cortex homogenate of 15-month-old mice (second cohort mice), *n*  =  3 for all groups. (**D**–**M**) Densitometry analysis of Western blots shown in Fig. 2G and (**C**), expressed as fold-change vs. Camk2a-Cre con. corrected to a loading control tubulin. *n*  = 6 individual dots shown for each group in all graphs except for (**F**) where n(3xTg-AD × *Mcu*^fl/fl^ × Camk2a-Cre) = 3. All data presented as mean ± SEM, ****P  <  0.0001, ***P  <  0.001, **P  <  0.01, *P  <  0.05. One-way ANOVA with Sidak’s multiple comparisons test was performed with adjusted *P* values shown in the graph left to right: (**D**) *P* = 0.000012, *P* = 0.000053; (**E**) *P* = 0.0384; (**F**) *P* = 0.0006, *P* = 0.009; (**G**) *P* = 0.000064, *P* = 0.01; (**H**) *P* = 0.0003, *P* = 0.0017; (**I**) *P* = 0.0186; (**K**) *P* = 0.0127. One-way ANOVA with Tukey’s multiple comparisons test was performed in (**L**) with adjusted *P* value shown, *P* = 0.0176. Source data are available online for this figure.

We examined whether ablating neuronal _m_Ca^2+^ uptake is protective against amyloid pathology. Cortical and hippocampal homogenates isolated from aged mice (15 months) were assayed by ELISA for Aβ_1-40_ and Aβ_1-42_ peptide levels in both RIPA-soluble and -insoluble fractions. Loss of neuronal _m_Ca^2+^ uptake in AD mice resulted in a significant decrease in both soluble and insoluble Aβ_1-40_ and Aβ_1-42_ levels in cortex and hippocampus, without any change in the Aβ_42/40_ ratio (Figs. 2A–D and EV2A,B). Similarly, loss of neuronal_m_Ca^2+^ uptake caused a significant reduction (~ 40%) in amyloid deposits in AD mice (Fig. 2E,F). To further investigate the mechanism by which genetic loss of *Mcu* reduces Aβ burden, we examined the expression of all secretases involved in APP processing including α, β and γ-secretase (Figs. 2G and EV2C). As expected, aged 3xTg-AD mice demonstrated increased expression of total APP, β (BACE-1) and γ-secretase complex components (PS1, NCT and APH1 subunit), whereas α-secretase (ADAM10) expression was reduced. Interestingly, deletion of neuronal *Mcu* from 3xTg-AD mice reverted the increased expression of γ-secretase complex associated proteins presenilin-1 (PS-1) and Nicastrin (NCT) (Figs. 2G and EV2C–I). As expected for this transgenic model, no significant changes in the expression of total APP were observed (Figs. 2G and EV2C,D). Semi-quantitative analysis of western blots showed a trending decrease in cortex expression of β-secretase (BACE-1) (Fig. EV2F) and anterior pharynx-defective phenotype 1 (APH-1) of the γ-secretase complex (Fig. EV2I) in 3xTg-AD x *Mcu*^fl/fl^ x Camk2a-Cre brains. We examined the expression of several APP processing fragments, including sAPPα, sAPPβ, AICD, and C99 and found no significant changes in expression (Figs. 2G and EV2J–M). These results suggest that the reduction in Aβ levels observed in 3xTg-AD x *Mcu*^fl/fl^ x Camk2a-Cre mice is likely not due to alternative cleavage of APP. Instead, the decreased Aβ levels may result from changes in β- or γ-secretase activity or other indirect mechanisms affecting Aβ production or turnover. This implies that loss of MCU influences Aβ deposition through pathways other than direct alterations in APP processing.

To further investigate how MCU deletion impacts Aβ regulation, we examined the role of autophagy in clearing Aβ aggregates. We analyzed autophagy markers in protein lysates from the frontal cortex of 15-month-old control, 3xTg-AD, and 3xTg-AD x *Mcu*^fl/fl^ x Camk2a-Cre mice (Fig. EV5M–O). However, we did not detect significant changes in the expression levels of LC3-II/LC3-I or p62, which are typically associated with autophagy activity. This could be due to the heterogenous tissue homogenate, which contains a significant number of non-neuronal cells that may mask differences in neuronal expression. Alternatively, these findings may suggest that the clearance of Aβ in this model does not occur through the traditional LC3-associated autophagy pathway and rather via alternative autophagic mechanisms.

### Loss of neuronal MCU expression decreases AD-associated tau pathology

**Figure 3:**
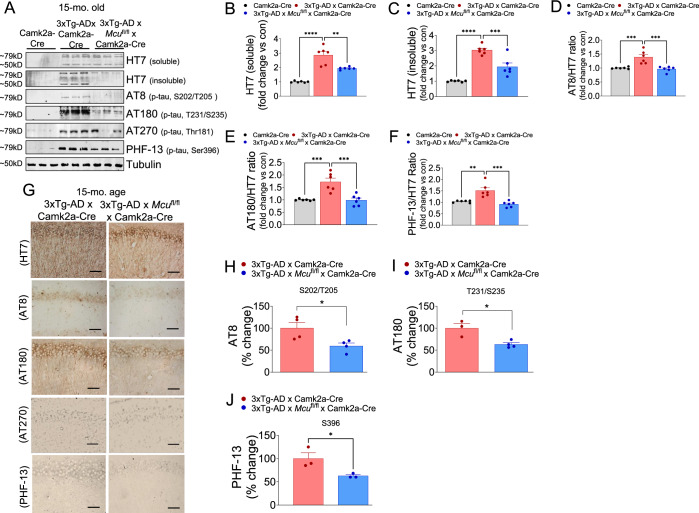
Loss of neuronal MCU expression decreases tau pathology. (**A**) Representative western blot of soluble and insoluble total tau (HT7), phosphorylated tau at residues S202/T205 (AT8), T231/S235 (AT180), T181 (AT270), and S396 (PHF13) in cortex homogenate of 15-month-old mice, *n*  =  3 for all groups. (**B**–**F**) Densitometric analysis of Western blots shown in (**A**) and Fig. EV3A, expressed as fold-change vs. Camk2a-Cre con. corrected to a loading control tubulin. Quantification is the sum of all replicates, *n* = 6. (**G**) Representative immunohistochemical staining for total tau (HT7), phospho-tau S202/T205 (AT8), phospho-tau T231/S235 (AT180) and S396 (PHF13) in the hippocampus of mice; scale bar = 50 μM. (**H**–**J**) Quantification of the integrated optical density area of AT8, AT180, AT270 and PHF-13 immunoreactivity, *n*  =  4 for all groups. All data presented as mean ± SEM, ****P  <  0.0001, ***P  <  0.001, **P  <  0.01, *P  <  0.05. One-way ANOVA with Tukey’s multiple comparisons test was performed for (**B**–**F**) with adjusted *P* values shown in the corresponding graphs left to right: (**B**) *P* = 0.0000007, *P* = 0.0014; (**C**) *P* = 0.0000006, *P* = 0.0007; (**D**) *P* = 0.0009, *P* = 0.0004; (**E**) *P* = 0.0004, *P* = 0.0003; (**F**) *P* = 0.0014, *P* = 0.0002. Unpaired *t* test was performed for (**H**–**J**) with *P* values 0.0322, 0.0137, and 0.0455, respectively, shown in the graphs. Source data are available online for this figure.

**Figure EV3:**
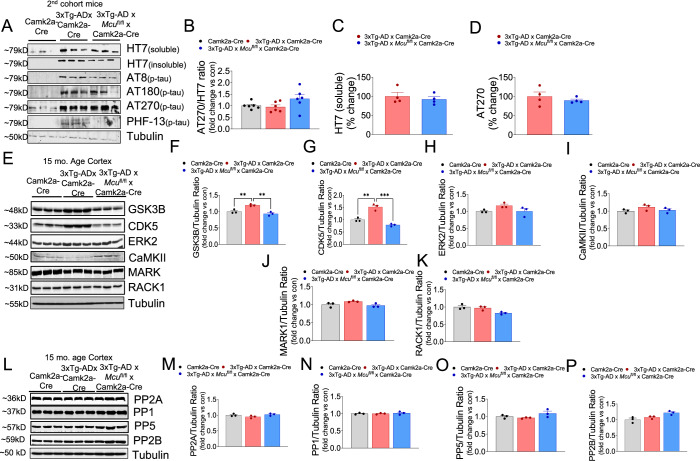
Effect of loss of neuronal MCU expression on tau pathology. (**A**) Western blots (2^nd^ cohort mice) of soluble and insoluble total tau (HT7), phosphorylated tau at residues S202/T205 (AT8), T231/S235 (AT180), T181 (AT270), and S396 (PHF13) in cortex homogenate of 15-month-old mice, *n*  =  3 for all groups. (**B**–**D**) Densitometric analysis of Western blots shown in Fig. 3A, and (**A**), expressed as fold-change vs. Camk2a-Cre con. corrected to a loading control, tubulin. Quantification is the sum of all replicates, *n* = 6. (**E**) Western blots for protein expression of GSK3β, CDK5, ERK2, CamkII, MARK, RACK1 and tubulin (loading control) for cortex homogenate of 15-month-old mice, *n*  =  3 for all groups. (**F**–**K**) Densitometric analysis of western blots shown in (**E**), expressed as fold-change vs. Camk2a-Cre con. corrected to a loading control, tubulin, *n* = 3 for all groups. (**L**) Western blots for protein expression of PP2A, PP1, PP5, PP2B and tubulin (loading control) for cortex homogenate of 15-month-old mice, *n*  =  3 for all groups. (**M**–**P**) Densitometric analysis of western blots shown in (**L**), expressed as fold-change vs. Camk2a-Cre con. corrected to a loading control, tubulin, *n* = 3 per group. All data presented as mean ± SEM, ****P  <  0.0001, ***P  <  0.001, **P  <  0.01, *P  <  0.05. To compare data in (**C**, **D**) *t* test was used. One-way ANOVA with Tukey’s multiple comparisons test was performed for other panels with adjusted *P* value shown from right to left: (**F**) *P* = 0.009, *P* = 0.002; (**G**) *P* = 0.0012, *P* = 0.0002. Source data are available online for this figure.

Neurofibrillary tangles are a histopathological hallmark of AD and are predominantly comprised of hyperphosphorylated tau. In all, 15-month-old 3xTg-AD mice showed a significant increase in the expression of total soluble and insoluble tau, and increased phosphorylation at all residues examined compared to non-AD controls (Figs. 3A–F and EV3A,B). Deletion of *Mcu* from AD mice caused a significant reduction in soluble and insoluble tau (Figs. 3A–C and EV3A), a striking reduction in S202/T205 (AT8 immunoreactivity), T231/ S235 (AT180 immunoreactivity) and Ser396 (PHF-13 immunoreactivity) tau phosphorylation, (Figs. 3A,D–F and EV3A) and a variable reduction in T181 (AT270 immunoreactivity) (Figs. 3A and EV3A,B). To validate these findings we performed immunohistochemistry to examine tau phosphorylation specifically in pyramidal neurons of the CA1 region of the brain and found a significant reduction in S202/T205 (~40%), T231/ S235 (~34%), and PHF-13 (~45%) tau phosphorylation in *Mcu*-deleted 3xTg-AD mice with no observed change in total soluble tau or AT270 (Figs. 3G–J and EV3C,D). These results demonstrate that loss of neuronal _m_Ca^2+^ uptake is sufficient to reduce tau pathology in AD.

To address the mechanism underlying reduced tau phosphorylation in MCU-deficient AD mice, we examined the levels of key tau kinases and phosphatases in protein lysate samples isolated from frontal cortex of 15-month-old Camk2a-Cre, 3xTg-AD x Camk2a-Cre, and 3xTg-AD x *Mcu*^fl/fl^ x Camk2a-Cre mice. We investigated the expression of GSK-3β, CDK5, ERK2, CaMKII, MARK, and RACK1 (Fig. EV3E–K). Increased GSK-3β expression has been associated with reduced neurogenesis and increased neuronal cell death in the hippocampus of AD patients (Llorens-Martin et al, 2013). CDK5, a serine/threonine kinase, plays a role in neuronal survival and cognitive function by regulating synaptic morphology in the central nervous system. The CDK5/p25 complex, which is significantly elevated in AD patients, phosphorylates APP, Thr-252 of β-secretase, and STAT3, and is reported to exacerbate AD pathology (Cruz et al, 2003; Iijima et al, 2000). We found that the expression of GSK-3β and CDK5 was significantly increased in 3xTg-AD mice and expression was reduced in 3xTg-AD x *Mcu*^fl/fl^ x Camk2a-Cre mice (Fig. EV3E–G). No significant changes were observed in the expression of MAPKs, CaMKII, MARK1, and ERK2 (Fig. EV3E,H–K).

We also examined the expression of major tau-related protein phosphatases, including Protein Phosphatase 2A (PP2A), Protein Phosphatase 1 (PP1), Protein Phosphatase 5 (PP5), and Calcineurin (PP2B), but found no significant changes (Fig. EV3L–P) between 3xTg-AD x Camk2a-Cre and 3xTg-AD x *Mcu*^fl/fl^ x Camk2a-Cre mice. Overall, our comprehensive analysis highlights that while tau-related kinases show differential regulation, tau-related phosphatases remain unchanged. This sheds light on the molecular mechanisms by which preserved mitochondrial function may impact tau pathology and underscores its potential as a therapeutic target.

### Loss of neuronal mitochondrial calcium uptake reduces oxidative stress, improves synaptic integrity, and reduces neuroinflammation

**Figure 4:**
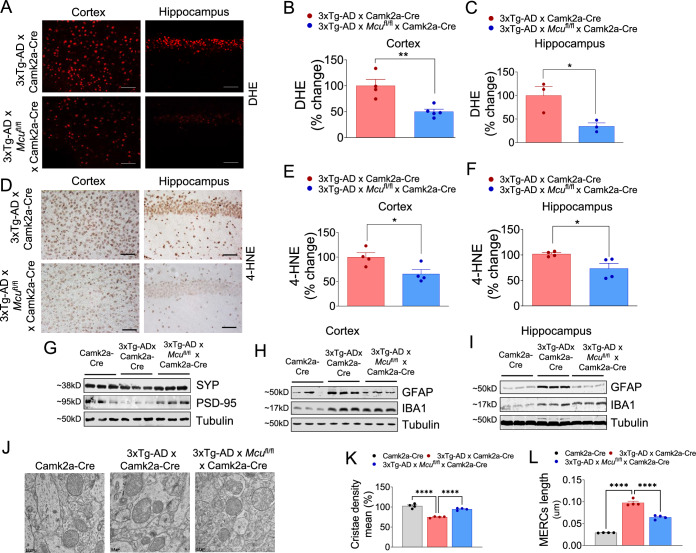
Neuronal deletion of *Mcu* reduces oxidative stress, improves synaptic integrity, and reduces neuroinflammation by preserving mitochondrial function. (**A**) DHE staining for ex vivo detection of superoxide production in freshly prepared cortical and hippocampal sections from 15-month-old mice. (**B**, **C**) DHE fluorescent intensity, percent change vs. 3xTg-AD × Camk2a-Cre controls. *n* (3xTg-AD × Camk2a-Cre) = 4, *n* (3xTg-AD × *Mcu*^fl/fl^ × Camk2a-Cre) = 5 in (**B**) and *n*  =  3 for all groups in (**C**). (**D**) Representative image of 4-HNE immunohistochemistry in cortex and hippocampus to detect lipid peroxidation in 15-month-old mice, *n* = 4 for all groups. (**E**, **F**) Percent change in 4-HNE-integrated optical density area corrected to 3xTg-AD × Camk2a-Cre controls. *n * =  4 for all groups. (**G**–**I**) Western blots for SYP, PSD-95, GFAP and IBA1 expression in tissue isolated from the cortex and hippocampus of 15-month-old mice, *n*  =  3 for all groups. (**J**) Representative transmission electron microscopy (TEM) images in the frontal cortex of 12-month-old mice; scale bar = 0.2 μM, *n*  =  4 for all groups. (**K**) Cristae density (%). (**L**) Mitochondria-endoplasmic reticulum contacts (MERC) distance (µM), *n* = 4 per group. All data presented as mean ± SEM, ****P  <  0.0001, ***P  <  0.001, **P  <  0.01, *P  <  0.05. One-way ANOVA with Tukey’s multiple comparisons test was performed for (**K**) with adjusted *P* values shown in the graphs left to right *P* = 0.000007 and *P *= 0.000099. One-way ANOVA with Sidak’s multiple comparisons test was performed for (**L**) with the following adjusted p values shown in the graphs left to right, *P* = 0.00000004 and *P* = 0.00001883. Unpaired *t* test was performed for (**B**, **C**, **E**, **F**) with corresponding *P* values shown in the graphs 0.0044, 0.0306, 0.0340, 0.0357. Source data are available online for this figure.

Increased oxidative stress, neuroinflammation, and synaptic dysfunction are early cellular occurrences in AD pathogenesis (Butterfield and Boyd-Kimball, 2018; Hong et al, 2016). To examine the effect of neuronal loss of _m_Ca^2+^ uptake on redox status, we utilized dihydroethidium (DHE) to monitor real-time superoxide generation in fresh brain slices. Deletion of *Mcu* from 3xTg-AD mice caused a significant reduction in ROS generation in both the cortex and hippocampus (Fig. 4A–C). Next, we assessed lipid peroxidation by staining for 4-hydroxy-2-nonenal (4-HNE) in aged AD mice and controls. Deletion of *Mcu* from 3xTg-AD mice caused a ~30% decrease in 4-HNE staining in the cortex and hippocampus (Fig. 4D–F).

**Figure EV4:**
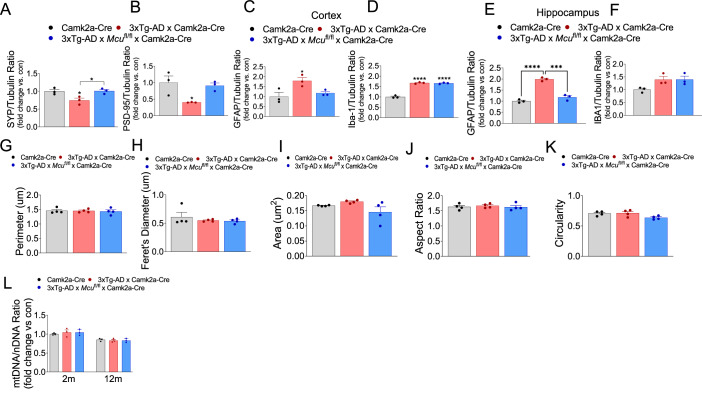
Loss of neuronal MCU preserves synaptic integrity and mitochondrial ultrastructure in AD mice. (**A**–**F**) Densitometric analysis of Western blots shown in Fig. 4G–I for SYP, PSD-95, GFAP and IBA1 expression. *n*  =  3 for all groups. (**G**, **K**) Quantification of the shape descriptors and morphological parameters for mitochondria. *n* = 4 for all groups. (**G**) Perimeter (μM). (**H**) Feret’s diameter (μM). (**I**) Area (μM^2^). (**J**) Aspect ratio. (**K**) Circularity. (**L**) Mitochondrial DNA (mtDNA)/nuclear DNA (nDNA) ratio in tissue isolated from the cortex of 2- and 12-months old mice, fold change vs. 2-month-old Camk2a-Cre controls, *n* = 3 for all groups. All data presented as mean ± SEM, ****P  <  0.0001, ***P  <  0.001, **P  <  0.01, *P  <  0.05. One-way ANOVA with Sidak’s multiple comparisons test was performed in (**A**, **G**–**K**) with adjusted *P* values in (**A**), *P* = 0.046 and *P* = 0.0409. One-way ANOVA with Tukey’s multiple comparisons test was performed in (**B**–**F**) with adjusted *P* values shown in the graphs left to right: (**B**) *P* = 0.0379; (**C**) *P* = 0.031; (**D**) *P* = 0.000005, *P* = 0. 000005; (**E**) *P* = 0.000045, *P* = 0. 000135. Two-way ANOVA with Sidak’s multiple comparisons test was performed in (**L**). Source data are available online for this figure.

Decreased synaptic plasticity and altered dendritic spines in excitatory glutamatergic synapses are early indicators of AD pathology (Almeida et al, 2005; Benarroch, 2018; Jackson et al, 2019). The vesicular protein, synaptophysin (SYP) is a marker of pre-synaptic integrity (Tarsa and Goda, 2002). Post-synaptic density protein (PSD-95) is a scaffolding protein localized at excitatory synapses (El-Husseini et al, 2000) which plays an important role in the process of learning (Migaud et al, 1998) and in the coupling of NMDA receptors and K^+^ channels at the post-synaptic membrane (Kim et al, 1996). Thus, we used these proteins as markers to assess synaptic plasticity and dendritic spine integrity. We observed a significant reduction in both SYP and PSD-95 expression in the brains of AD mice which was corrected to non-disease, control levels in the brains of 3xTg-AD x *Mcu*^fl/fl^ x Camk2a-Cre mice (Figs. 4G and EV4A,B). Similar findings were reported in (Cai et al, 2022) where MCU knockdown increased the numbers of synapses and dendritic spines in 3xTg-AD mouse model.

Astrocyte and microglia activation are early features of AD-associated inflammation (Habib et al, 2020; Park et al, 2021). The expression of glial fibrillary acidic protein (GFAP), a marker of astrocyte reactivity (Caruso et al, 2013), was significantly decreased in brain cortex homogenates isolated from 3xTg-AD x *Mcu*^fl/fl^ x Camk2a-Cre, when compared to 3xTg-AD mice, without any changes in Ionized calcium binding adaptor molecule 1 (IBA1) expression, a marker of microglia activation (Figs. 4H and EV4C,D). Similarly, GFAP expression was significantly decreased, while IBA1 expression was unchanged, in the brain hippocampus of 3xTg-AD x *Mcu*^fl/fl^ x Camk2a-Cre, as compared to 3xTg-AD mice (Figs. 4I and EV4E,F). This suggests that the astrocyte-mediated inflammatory response is rescued; however, there is no effect on microglia-mediated inflammation in the hippocampus region of the AD mice brain with MCU inhibition.

To determine if changes in mitochondrial content occurred in our model system, we performed transmission electron microscopy (TEM) to examine in situ mitochondrial morphology. Four mice per genotype were examined and >200 mitochondria were analyzed per sample by a blinded investigator. No significant differences were observed in mitochondrial number per area or in mitochondrial shape (area, perimeter, circularity, Feret’s diameter, aspect ratio) in the frontal cortex of 12-month-old mice (Figs. 4J and EV4G–K). However, we observed significant changes in cristae density and mitochondrial apposition, the length of mitochondria and endoplasmic reticulum contacts (MERCs). Specifically, cristae density was increased in 3xTg-AD x *Mcu*^fl/fl^ x Camk2a-Cre mice compared to 3xTg-AD mice. Additionally, the mean MERC length was reduced in 3xTg-AD x *Mcu*^fl/fl^ x Camk2a-Cre mice, as compared to 3xTg-AD mice (Fig. 4J–L). The quantification of the ratio of mtDNA-to-nDNA corroborated that there was no change in mitochondrial content. In addition, there was no change in mitochondria mass between the groups at two timepoints (Fig. EV4L), but our data does support that mitochondrial mass decreases with age. All together, these results suggest that decreasing _m_Ca^2+^ uptake during AD pathogenesis reduces oxidative stress and inflammation and preserves synaptic integrity with improvements in mitochondrial morphology and sub-cellular apposition.

### Loss of _m_Ca^2+^ uptake prevents AD-associated mitochondrial dysfunction and promotes autophagic clearance of amyloid

**Figure 5:**
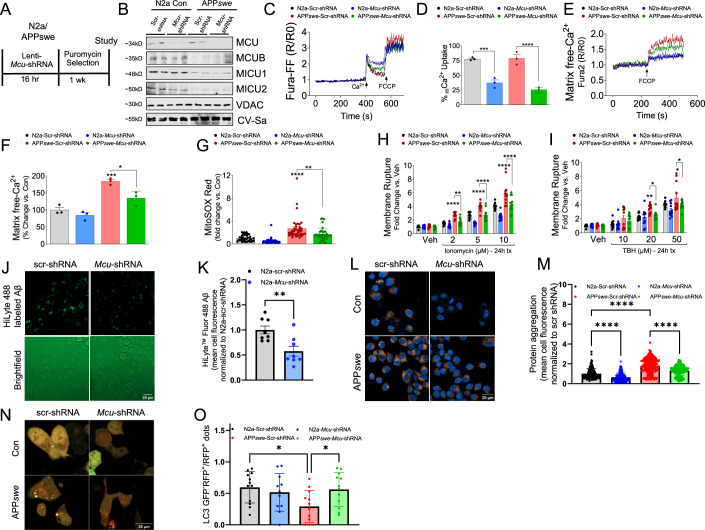
MCU loss in neurons prevents protein aggregation and amyloidogenic pathology by reducing mitochondrial ROS production and upregulating autophagy. (**A**) An Experimental protocol timeline for generation of stable Mcu knockdown N2a/APPswe cells. (**B**) Western blots for MCU expression and proteins associated with _m_Ca^2+^ uptake in N2a and APPswe cells transduced with lentivirus encoding shRNA targeting *Mcu. (***C**) Representative recordings of _m_Ca^2+^ uptake. (**D**) Percent change in _m_Ca^2+^ uptake. controls (**E**) Representative traces for basal _m_Ca^2+^ content, *n*  =  3. (**F**) Quantification of _m_Ca^2+^ content, *n* = 3 per group. (**G**) Quantification of MitoSOX fluorescent intensity; fold change vs. N2a Scr-shRNA controls, *n* = 37. (**H**, **I**) Assessment for plasma membrane rupture, using Sytox Green after treatment with (**H**) Ionomycin (Ca^2+^ ionophore, 2–10 µM) and (**I**) tert-Butyl hydroperoxide (TBH, oxidizing agent, 10–50 µM), *n* = 12 per group. (**J**) Representative images for HiLyte™ Fluor 488-labeled Aβ 24 h after addition to the cells, *n* = 8 per group. (**K**) Quantification for (**J**), normalized to scr-shRNA control. (**L**) Representative images of the fluorescent reporter Proteostat - protein aggregation. (**M**) Quantification for L, normalized to scr-shRNA control. *n* (scr-shRNA) = 120, *n* (*Mcu*-shRNA) = 173, *n* (APP*swe*- scr-shRNA) = 235, *n* (APP*swe-Mcu*-shRNA) = 326. (**N**) Representative images for cells expressing tandem fluorescent-tagged LC3 (mRFP-EGFP-LC3). (**O**) Quantification of autolysosome percentage to the total number of LC3 positive structures measured as ratio of LC3-GFP^-^RFP^+^ dots to LC3-RFP^+^ dots, n(*Mcu*-shRNA) = 11, *n* = 12 for other groups. *n* = individual dots shown for each group in all graphs. Data in (**A**–**H**) presented as mean ± SEM; Data in (**J**–**O**) presented as mean ± SD, ****P  <  0.0001, ***P  <  0.001, **P  <  0.01, *P  <  0.05. To compare data in (**K**) *t* test was used with *P* value 0.0047. To compare data in (**D**) one-way ANOVA with Tukey’s multiple comparisons test was used with adjusted *P* values shown in the graph left to right, *P* = 0.000601 and *P* = 0.000084. To compare data in (**F**, **G**, **M**, **O**) one-way ANOVA with Sidak’s multiple comparisons test was used with adjusted *P* values shown in the graphs left to right: (**F**) *P* = 0.0007, *P* = 0.0203; (**G**) *P* = 0.00000003, 0.00202189; (**M**) *P* = 0.00000056, *P* < 0.000000000000001, *P* < 0.000000000000001; (**O**) *P* = 0.0161, *P* = 0.0345. Two-way ANOVA with Sidak’s multiple comparisons test in (**H**, **I**) was used with adjusted *P* values shown in the graphs left to right: (**H**) *P* = 0.000032, *P* = 0.001087, *P* = 0.000039, *P* = 0.000043, *P* = 0.00000000008, *P* = 0.00000000838; (**I**) *P* = 0.0058, *P* = 0.0322, *P* = 0.0446, *P* = 0.0299. Source data are available online for this figure.

**Figure EV5:**
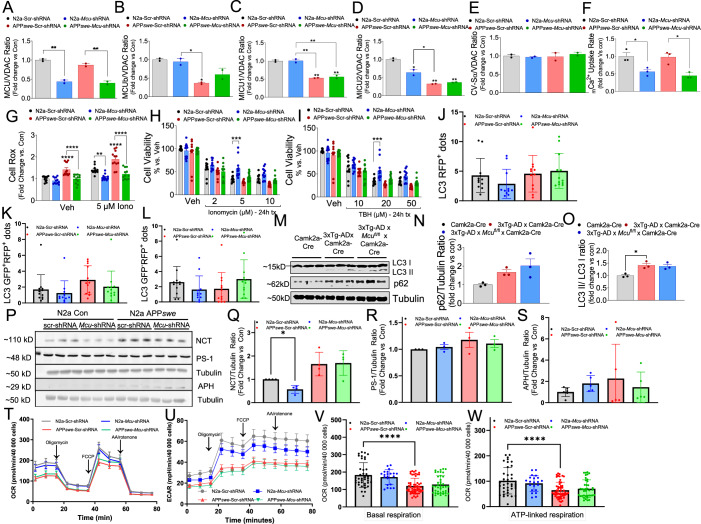
Loss of MCU reduces mitochondrial Ca^2+^ uptake, oxidative stress, and protein aggregation and restores autophagic flux in APPswe cells. (**A**–**E**) Quantification of protein expression associated with _m_Ca^2+^ exchange expressed as fold-change vs. N2a Scr-shRNA con. and corrected to a mitochondrial loading control, VDAC, *n* = 2 for each group. (**F**) Fold-change in _m_Ca^2+^ uptake rate of N2a-*Mcu*-shRNA, APPswe and APPswe -*Mcu*-shRNA vs. N2a Scr-shRNA controls, *n* = 3 per group. (**G**) Quantification of CellRox green fluorescent intensity (total cellular ROS production); fold change vs. N2a Scr-shRNA controls. (**H**, **I**) Assessment for cell viability using Cell Titer Blue after treatment with, (**H**) Ionomycin (Ca^2+^ overload, 2–10 µM), (**I**) tert-Butyl hydroperoxide (TBH, oxidizing agent, 10–50 µM), *n* = 12 individual dots shown for each group. (**J**–**L**) Quantification of autophagic structures per cell (*n* (*Mcu*-shRNA) = 11, *n* = 12 for other groups), including: (**J**) RFP⁺ puncta representing total red puncta (**K**) GFP⁺RFP⁺ puncta representing autophagosomes (**L**) GFP⁻RFP⁺ puncta representing autolysosomes. (**M**) Representative western blot of LC3-I, LC3-II, p62 and tubulin (loading control) for cortex homogenate of 12-month-old mice, *n*  =  3 for all groups. (**N**, **O**) Densitometry analysis of western blots shown in Fig. EV2M expressed as fold-change vs. Camk2a-Cre con. corrected to tubulin *n*  =  3/groups. (**P**) Western blots for protein expression of NCT, PS-1, APH and tubulin (loading control) for cells homogenate of N2a Scr-shRNA, N2a-*Mcu*-shRNA, APPswe and APPswe -*Mcu*-shRNA. (**Q**–**S**) Densitometric analysis of western blots shown in (**P**), expressed as fold-change vs. N2a Scr-shRNA con. corrected to a loading control, tubulin, *n* = 4 per group in (**Q**), *n *= 3 per group in (**R**), *n* = 5 per group in (**S**) except for *n* (Scr-shRNA) = 6. (**T–W**) Metabolic profiling of neuronal cells, *n* (scr-shRNA) = 39, *n* (*Mcu*-shRNA) = 21, *n* (APP*swe*- scr-shRNA) = 54, *n* (APP*swe-Mcu*-shRNA) = 39. (**T**) Representative traces of oxygen consumption rate (OCR) analysis. (**U**) Representative extracellular acidification rate (ECAR) traces. (**V**, **W**) Quantification of basal respiration and ATP-linked respiration in N2a Scr-shRNA, N2a Mcu-shRNA, APPswe, and APPswe Mcu-shRNA cells. Data presented as mean ± SEM, ****P  <  0.0001, ***P  <  0.001, **P  <  0.01, *P  <  0.05. One-way ANOVA with Sidak’s multiple comparisons test was performed in (**A**, **C**–**F**, **J**–**L**, **V**, **W**) with adjusted *P* values shown in the graphs left to right: (**A**) *P* = 0.0025, *P* = 0.005; (**C**) *P* = 0.0036, *P* = 0.0037, *P* = 0.0045, *P* = 0.0047; (**D**) *P* = 0.0221, *P* = 0.0371, *P* = 0.0221, *P *= 0.0227; (**F**) *P* = 0.0398, *P* = 0.0136; (**V**) *P* = 0.000000286; (**W**) *P* = 0.000025. One-way ANOVA with Tukey’s multiple comparisons test was performed in (**B**, **N**, **O**) with adjusted *P* values shown in the graphs left to right: (**B**) *P *= 0.0149, *P* = 0.0208; (**C**) *P* = 0.031. Repeated measures ANOVA with Sidak’s multiple comparisons test was performed in (**Q**–**R**) with adjusted *P* value in (**Q**) *P *= 0.0401. Two-way ANOVA with Sidak’s multiple comparisons test was performed in (**G**–**I**, **S**) with adjusted *P* values shown in the graphs left to right: (**G**) *P* = 0.000095, *P* = 0.000076, *P* = 0.0026, *P* = 0.0000049, *P* = 0.0000000005; (**H**) *P* = 0.000117; (**I**) *P* = 0.0002. Source data are available online for this figure.

To directly examine the mitochondrial consequences of *Mcu* loss and the molecular mechanisms affording neuroprotection, we utilized a neuroblastoma cell line harboring a transgene for the well characterized APP Swedish mutation (N2a-APPswe: K670N, M671L)(Thinakaran et al, 1996). We transduced control (N2a) and APPswe cells with lentivirus encoding shRNA targeting *Mcu* (Fig. 5A). Western blot analysis revealed ~55% loss of MCU protein in shRNA stable knockdown cells, as compared to scramble shRNA control cells (Scr-shRNA) (Figs. 5B and EV5A). We did not see any significant changes in the expression of other mtCU components in *Mcu* knockdown cells (Fig. EV5B–D). However, similar to what we previously reported in sporadic AD patients (Jadiya et al, 2019), we noted significant decreases in the expression of the mtCU regulators MICU1, MICU2, and MCUB (Mallilankaraman et al, 2012; Perocchi et al, 2010; Plovanich et al, 2013; Raffaello et al, 2013) in AD neurons and the loss of these proteins suggest a predisposition towards increased _m_Ca^2+^ uptake and overload (Fig. EV5B–D). Attenuation of _m_Ca^2+^ uptake in *Mcu* knockdown cells was validated in a permeabilized cell system using the ratiometric reporters FuraFF (Ca^2+^) and JC1 (Δψ) (Figs. 5C,D and EV5F). To evaluate matrix free-Ca^2+^ content, cells from all groups were permeabilized with digitonin and treated with thapsigargin to inhibit SERCA, and then treated with FCCP to release all matrix free-Ca^2+^, as previously reported (Jadiya et al, 2019; Luongo et al, 2015). APPswe cells were found to have increased _m_Ca^2+^ as compared to N2a controls. Further, knockdown of *Mcu* from APPswe cells significantly reduced _m_Ca^2+^ content (Fig. 5E,F).

A number of studies suggest a strong association between _m_Ca^2+^ overload, oxidative stress and metabolic dysfunction (Gandhi et al, 2009; Luongo et al, 2017; Luongo et al, 2015; Sarasija et al, 2018) and both are hypothesized to be causal contributors to AD progression. We monitored the cells for changes in oxidative phosphorylation (OxPhos) by measuring mitochondrial oxygen consumption rates (OCR) in a Seahorse assay (Fig. EV5T–W). Stable expression of mutant APPswe elicited a significant decrease in basal respiration and ATP-linked respiration and these parameters were not affected with loss of MCU (Fig. EV5T–W). This result might be explained by a smaller role for mitochondrial calcium uptake in mitochondrial bioenergetics in immature neurons that mostly rely on glycolysis rather than oxidative phosphorylation. In support of this, APPswe cells displayed a more pronounced change in glycolysis, measured as extracellular acidification rate (ECAR), as opposed to OCR (Fig. EV5U). To examine redox status, we measured total cellular ROS content using the CellRox assay and mitochondrial superoxide production using the MitoSOX Red reporter. AD-mutant cell lines displayed enhanced ROS levels (total and mitochondrial), and both were reduced with loss of MCU (Figs. 5G and EV5G). To further evaluate MCU-mediated mechanisms of cytoprotection, cells from all groups were examined for plasma membrane rupture and viability following treatment with ionomycin (Ca^2+^ stress), and *tert*-butyl hydroperoxide (ROS stress). We observed a significant decrease in membrane rupture, a characteristic of necrotic cell death, and tendency toward enhanced general cellular viability in *Mcu* knockdown APPswe cell lines (Figs. 5H,I and EV5H,I).

To further define the molecular mechanisms affording cytoprotection against amyloid aggregation and AD pathology, we extensively examined the gamma-secretase components, Nicastrin (NCT), Presenelin-1, and APH in N2a APPswe cells (Fig. EV5P–S), but found no significant changes in expression. However, MCU loss did reduce Aβ accumulation 24-h after the addition of HiLyte™ Fluor 488-labeled Aβeta to the cells (Fig. 5J,K) and protein aggregation as measured by the Proteostat reporter dye (Fig. 5L,M) indicating a possible upregulation of clearance. Indeed, we found that APPswe reduces basal autophagy in N2a neurons, and this is rescued by MCU ablation measured as the percentage of autolysosomes corrected to the total number of LC3 positive structures in the resting condition using tandem fluorescent-tagged LC3 (mRFP-EGFP-LC3) (Figs. 5N,O and EV5J–L). Autophagic capacity can be impaired by the accumulation of damaged mitochondria thus creating a potential bottleneck in the clearance of amyloid and damaged material (Call and Nichenko, 2020; Nichenko et al, 2020). Autophagic capacity has also been reported to be reduced by mitochondrial dysfunction and excessive ROS generation (Roca-Agujetas et al, 2019). Importantly, all of these are partially rescued with the loss of MCU, providing a direct link between the inhibition of _m_Ca^2+^ uptake in neurons and protection against AD pathology.

## Discussion

Our group has previously reported that loss of _m_Ca^2+^ efflux capacity contributes to the pathogenesis and progression of Alzheimer’s disease (AD) by promoting mitochondrial Ca^2+^ (_m_Ca^2+^) overload. Here, we report that neuronal loss of mitochondrial Ca^2+^ uptake prevents AD-pathology and age-dependent cognitive decline in a robust mouse model of familial AD. While previous studies provide supportive anecdotal evidence of mitochondrial dysfunction resulting from _m_Ca^2+^ overload (Begley et al, 1999; Ferreira et al, 2015; Paula-Lima et al, 2011), our results demonstrate a causal role for dysregulated _m_Ca^2+^ transport mechanisms as an early pathogenic mechanism underlying the development and progression of AD and potentially other neurodegenerative diseases including ALS and Parkinson’s disease. In support, glutamate excitotoxicity due to excessive _i_Ca^2+^ promotes neuronal dysfunction in many diseases (Fan and Raymond, 2007; Lau and Tymianski, 2010; Wu et al, 2004), and MCU overexpression worsens excitotoxic cell death (Qiu et al, 2013). Previous studies demonstrate that _m_Ca^2+^ overload is sufficient to cause dendritic degeneration in a model of late-onset familial Parkinson’s disease (PD) (Verma et al, 2017). Further, pharmacologic and genetic inactivation of MCU is neuroprotective in a *pink1*^*−/−*^ zebrafish model of PD (Soman et al, 2017; Soman et al, 2019). Strong correlative evidence that mtCU dysregulation is sufficient to cause brain and skeletal muscle disorders is seen in patients with loss-of-function mutations in *MICU1* (Logan et al, 2014*)*. Indirect evidence from reports of increased ER-mitochondria crosstalk (Area-Gomez et al, 2012) and Ca^2+^ transfer in AD, also may contribute to increased _m_Ca^2+^ content. Indeed, neuronal cells expressing apolipoprotein E4 (apoE4), a major genetic risk factor for AD, displayed increased levels of _m_Ca^2+^ and ER-mitochondria tethering which promoted mitochondrial impairments and neuronal dysfunction (Orr et al, 2019). In totality, these reports coupled with our current results suggest alterations in mitochondrial Ca^2+^ handling are upstream of classical histopathological markers of AD such as Amyloid and Tau aggregation and deposition.

The exact mechanism for the neuroprotective effect of abrogating neuronal mitochondrial Ca^2+^ overload is likely multifactorial and involves decreasing mitochondrial ROS, preserving energetics, inhibiting cell death signaling, and enhancing autophagic capacity, ultimately preserving mitochondrial function. The AD brain is extremely vulnerable to redox imbalance due to the high neuronal energy demand. The increased expression and activity of β and γ secretase, and increased phosphorylation of tau are associated with oxidative stress (Jo et al, 2010), mitochondrial dysfunction (Gabuzda et al, 1994; Rhein et al, 2009), and impaired cellular energetics (Gabuzda et al, 1994). The link between Ca^2+^ and oxidative stress is supported by several previous reports. Elevated _i_Ca^2+^ enhances oxidative stress in neurons and astrocytes(Petersen et al, 2000). Moreover, the loss of MICU1 and a resultant increase in _m_Ca^2+^ content elicits mitochondrial superoxide generation (Mallilankaraman et al, 2012) and is linked with excitotoxicity (Starkov et al, 2004). In contrast, the inhibition of _m_Ca^2+^ uptake reduces NMDAR-induced excitotoxicity and neuronal cell death (Stout et al, 1998). Previous experiments support that inhibition of mtCU-dependent _m_Ca^2+^ uptake by either Ru360 treatment or siRNA knockdown of *Mcu* decreases oxidative stress in microglia cells in vitro (Xie et al, 2017) and in primary cerebellar granule neurons (Liao et al, 2015). Consistent with these findings, increased MCU activity is sufficient to promote oxidative stress (De Stefani et al, 2011; Liao et al, 2015). Moreover, increased ER-mitochondrial Ca^2+^ transfer in *PSEN* mutants is linked to high ROS production (Sarasija et al, 2018). Thus, as reported here, increased _m_Ca^2+^ content in AD can drive excess ROS production, furthering neuronal compromise and disease progression.

Another contributing mechanism underlying to the neuroprotective consequence of *Mcu* deletion may be reduced gliosis. Recently, MCU overexpression in cortical neurons of mice showed increased immunohistochemical staining for GFAP, suggestive of increased gliosis (Granatiero et al, 2019). Our current results agree with this observation, as we found a reduction in neuroinflammation with the ablation of _m_Ca^2+^ uptake. Furthermore, a consequence of reduced gliosis in AD may be preservation of synaptic integrity, as studies correlate enhanced gliosis with loss of PSD-95 (Gylys et al, 2004). However, the specific mechanisms by which defective _m_Ca^2+^ exchange modulates the functional synaptic unit and gliosis in AD remain to be elucidated.

Precisely why _m_Ca^2+^ uptake increases during AD pathogenesis remains unclear. Undoubtedly, neurons require a high energy supply for numerous functions such as synaptic transmission, action potential firing, and synapse development (Oyarzabal and Marin-Valencia, 2019; Vergara et al, 2019) and are highly dependent on mitochondrial metabolism to produce ATP (∼93%) via OxPhos (Harris et al, 2012). We hypothesize that neurons initially elevate _m_Ca^2+^ content via proteomic remodeling of transport machinery (NCLX, mtCU) to increase ATP production by increasing activity of the TCA cycle rate-limiting enzymes (PDH, α-KGDH, and ICDH). Proteomic remodeling of mtCU and NCLX were observed in our in vivo and in vitro AD models (Jadiya et al, 2019), suggesting that initially it may be an adaptive response to augment cellular energetics during stress, which subsequently becomes maladaptive and results in _m_Ca^2+^ overload, oxidative stress, and the accumulation of dysfunctional mitochondria causing a bottleneck in the clearance of damaged cell material by autophagy (Call and Nichenko, 2020; Nichenko et al, 2020). Altogether, this exacerbates amyloidosis and ultimately induces cell death. It is also known that aged or damaged mitochondria have a lower calcium buffering capacity and are more susceptible to calcium overload (Panel et al, 2018) which further supports mtCU dysregulation in AD. We previously demonstrated that _m_Ca^2+^ homeostasis can be restored by increasing NCLX _m_Ca^2+^ efflux in AD (Jadiya et al, 2019), and here we provide strong evidence that reducing mtCU _m_Ca^2+^ uptake lessens AD progression.

The mechanism underlying the neuroprotective effect of MCU ablation is likely multifactorial given the many consequences of targeting _m_Ca^2+^ signaling. In the present study, ablation of MCU in an in vitro model of Aβ accumulation restored basal autophagic flux. However, the same effect was not observed in frontal cortex tissue lysates from 15-month-old mice when measured by LC3-II/LC3-I and p62. Although this is likely a function of the complexities and heterogeneity of in vivo versus in vitro systems, additional mechanisms cannot be ruled out. Additionally, recent studies have suggested that TMEM65 and NCLX act as Na^+^/Ca^2+^ and H^+^/Ca^2+^ exchangers, respectively (Fan et al, 2025; Zhang et al, 2025) although this remains hotly debated (Garbincius and Elrod, 2025). Future studies should provide greater mechanistic insight while also interrogating the potential of TMEM65 as a therapeutic target in neurodegeneration.

Even so, what is most impactful regarding this study remains the validation of mitochondrial calcium overload as a viable therapeutic target for halting the development and progression of AD. Despite significant investment and repeated attempts to leverage protein aggregation as a therapeutic strategy for AD, clinical trials on such drugs have been met with limited success and these drugs have not been widely adopted in the clinic due to limited benefit and safety concerns. Given the growing population of AD patients worldwide, there is an urgent need for novel therapeutic targets and strategies. Here, we have provided our second means of genetic proof-of-concept that mitochondrial calcium overload is a viable, safe, and effective strategy for the treatment of AD. Further studies are needed to determine the most efficacious pharmacological strategy for targeting _m_Ca^2+^ dysregulation in AD. Even so, our studies provided strong evidence that mitochondrial calcium is a causal contributor to AD pathogenesis and, most importantly, a promising therapeutic target in AD.

## Methods

**Table:** Reagents and tools table

Reagent/resource	Reference or source	Identifier or catalog number
**Experimental models**		
*Mcu* floxed mice	Luongo et al, 2015	N/A
B6.Cg-Tg(Camk2a-cre)T291-Stl/J	The Jackson Laboratory, USA	Stock # 005359
3xTg-AD	The Jackson Laboratory, USA	Stock # 34830
*Mcu*^fl/fl^ × Camk2a-Cre	This study	N/A
3xTg-AD × *Mcu*^fl/fl^ × Camk2a-Cre	This study	N/A
Neuro-2a cells (N2a)	ATCC	CCL-131
N2a-APPswe	Jadiya et al, 2019	N/A
N2a-*Mcu*-shRNA	This study	N/A
APPswe-*Mcu*-shRNA	This study	N/A
**Recombinant DNA**		
Tandem-tagged LC3-EGFP-RFP	Addgene	21074
CMV-mito-GEM-GECO1	Addgene	32461
**Antibodies**		
Anti-β-amyloid	BioLegend	800701
Aβ-4G8	Covance	SIG-39220
4 hydroxynonenal	Abcam	Ab48506
MCU	Cell Signaling	14997S
MCUB	Abgent	AP12355b
MICU2	Abcam	ab101465
MICU3	Sigma	HPA024771
EMRE	Santa Cruz Biotechnology Inc	sc-86337
NCLX	Santa Cruz Biotechnology Inc.	sc-161921
VDAC	Abcam	ab14734
Total OXPHOS	Abcam	ab110413
APP (22C11)	Millipore Sigma	MAB343
BACE1	Millipore Sigma	MAB5308
ADAM-10	Millipore Sigma	AB19026
PS1	Sigma-Aldrich	P7854-2ML
Nicastrin	Cell Signaling	3632
APH1	Millipore Sigma	AB9214
HT7	Thermo Fisher Scientific	
AT180	Thermo Fisher Scientific	MN1040
AT270	Thermo Fisher Scientific	MN1050
AT8	Thermo Fisher Scientific	MN1020
PHF13	Cell Signaling Technology	9632
Beta-tubulin	Cell Signaling Technology	3623S
SYP	Santa Cruz Biotechnology	sc-55507
PSD-95	Invitrogen	MA1-045
GFAP	Santa Cruz Biotechnology	sc-33673
IR secondary antibodies	Licor	32210, 926-32211, 926-68071, 926-68071
**Oligonucleotides and other sequence-based reagents**		
*CoxII-F*	GCCGACTAAATCAAGCAACA	N/A
*CoxII-R*	CAATGGGCATAAAGCTATGG	N/A
**Chemicals, enzymes and other reagents**		
Paraformaldehyde	Sigma-Aldrich	P6148
Fetal bovine serum	VWR	45001-108
RIPA lysis buffer	EMD Millipore	20-188
SIGMAFAST™ Protease Inhibitor	Sigma-Aldrich	S8830-20TAB
Phosphatase inhibitor	Roche	04906837001
Formic acid	Sigma-Aldrich	399388
Sodium hydroxide	Research Products International	S24000-500
Bio-Rad Protein Assay Dye Reagent	Bio-Rad	5000006
Blocking buffer	Rockland	MB-070
TBS-T	Thomas Scientific	C993H69
Dihydroethidium (DHE)	Thermo Fisher Scientific	D11347
CellROX Green Reagent	Thermo Fisher Scientific	C10444
PVDF Immobilon-FL membrane	MD Millipore	IPFL00010
SYTOX Green	Invitrogen	S7020
MitoSOX Red	Thermo Fisher Scientific	M36008
Ionomycin	Cayman Chemical Company	11932
Tert-Butyl hydroperoxide	Sigma-Aldrich	458139
Cell Titer Blue Reagent	Promega	G8080
Thapsigargin	Enzo Life Sciences	BML-PE180-0005
Digitonin	Sigma-Aldrich	D141
succinate	Sigma-Aldrich	S3674
Chelex 100	Bio-Rad	1422822
Fura-FF	Cayman Chemical Company	20415
FCCP	Sigma-Aldrich	C2920-10MG
Calcium Green 5 n	Life Technology/Invitrogen	C3737
Fugene HD	Promega	e2312
Oligomycin	Sigma-Aldrich	75351
DMEM	Corning	15-013-CV
Antimycin A	Sigma-Aldrich	A8674
Poly-L-lysine	Sigma-Aldrich	P4707
**Software**		
PACKWIN	Panlab, Harvard Apparatus, USA	https://www.harvardapparatus.com/packwin-software-panlab.html
Fiji ImageJ	Fiji ImageJ	https://imagej.net/software/fiji/
GraphPad Prism 9	GraphPad Software	https://www.graphpad.com/scientific-software/prism/
Zen 2010	Carl Zeiss	https://www.zeiss.com/microscopy/en/products/software/zeiss-zen.html
ImageJ Pro plus software	Meyer Instruments	https://www.meyerinst.com/mediacybernetics/image-pro-plus/
**Other**		
*Mcu*-shRNA	Mission shRNA, Sigma	TRCN138929
Human Beta-Amyloid (1-40) ELISA Kit	Wako Chemicals USA	298-64601
Human Beta-Amyloid (1-42) ELISA Kit	Wako Chemicals USA	298-62401
HiLyte™ Fluor 488-labeled Aβ	Anaspec	AS-60479-01
Proteostat aggresome detection kit	Enzo	ENZ-51035-K100
DNeasy blood and tissue kit	Qiagen	69504

### Neuronal specific *Mcu* knockout 3xTg-AD mutant mouse

*Mcu* floxed mice were generated as reported previously by our lab (Luongo et al, 2015) by acquiring targeted ES cells made by recombinant insertion of a construct containing loxP sites flanking exons 5–6 of the *Mcu* gene (ch10: 58,930,544–58,911,529). Mutant ES cell lines were confirmed by PCR and injected into C57BL/6N blastocysts with subsequent transplantation into pseudo- pregnant females. Germline mutant mice were crossed with ROSA26-FLPe knock-in mice for removal of the FRT-flanked neomycin resistance cassette. Resultant homozygous *Mcu*^fl/fl^ mice were crossed with neuron specific-Cre transgenic mice, *Camk2a*, to generate neuron-specific *Mcu* knockouts. The Calcium/calmodulin-dependent protein kinase II alpha (*Camk2a*) promoter drives Cre recombinase expression in the forebrain, specifically in the cortex and hippocampus (Tsien et al, 1996). Resultant neuronal-specific loss-of-function models (*Mcu*^fl/fl^ x Camk2a-Cre) were crossed with 3xTg-AD mutant mouse (Oddo et al, 2003) to generate 3xTg-AD x *Mcu*^fl/fl^ x Camk2a-Cre mutant mice. 3xTg-AD mice harbors three mutations: human Psen1 mutation (PS1^M146V^ knock-in), human amyloid precursor protein Swedish mutation (APP^Swe^ KM670/671NL), and P301L mutation of human tau (tau^P301L^).

### Cell cultures and stable cell lines

Mouse neuro-2a cells (N2a) and N2a cells stably expressing human APP with the K670N/M671L Swedish mutation (APPswe) were propagated in Dulbecco’s modified Eagle’s medium containing 10% fetal bovine serum and 1% penicillin/streptomycin. Cells were maintained at 37 °C in a humidified 5% CO_2_ incubator. APPswe cells were grown in the presence of 400 µg/mL G418 (Jadiya et al, 2019). Differentiation of N2a and APPswe was performed using standard protocols described earlier (Evangelopoulos et al, 2005; Jadiya et al, 2019) in media containing 50% Dulbecco’s modified Eagle’s medium (DMEM) and 50% OPTI-MEM with 1% penicillin/streptomycin for 72 h (Jadiya et al, 2019). Cells were seeded onto poly-D-lysine coated glass coverslips for all imaging studies.

To generate *Mcu* knockdown stable N2a/APP cells, we transduced cells with lentivirus encoding shRNA targeting *Mcu* for 16 h. in the presence of 4 mg/ml polybrene. Stably transduced cells were selected with puromycin (2 mg/ml) 48 h. post transduction for one week and expanded. Knockdown of *Mcu* was evaluated by Western blot.

### Immunohistochemistry

Mouse brains were dissected longitudinally at the center, and half of the brain was frozen on dry ice for biochemical analysis. The other was used for immunohistochemistry as previously described (Jadiya et al, 2019). In brief, brains were immersion-fixed in 4% paraformaldehyde (PFA) for 24 h. and embedded in paraffin. Serially sectioned (6-μm thick) brains were then deparaffinized, hydrated, and blocked in 2% fetal bovine serum. Sections were incubated overnight at 4 °C with following primary antibodies, monoclonal anti-β-amyloid, 17-24 (Aβ-4G8) dilution 1:150, HT7 dilution 1:150, phospho-tau (pThr231) monoclonal AT180 dilution 1:50, phospho-Tau (Ser202, Thr205) monoclonal AT8 dilution 1:50, anti-4 hydroxynonenal antibody (4-HNE) dilution 1:20, and then incubated with secondary antibody. The Vector Elite ABC system, an avidin/biotin-based peroxidase method, was used with diaminobenzidine, as the chromogen, to visualize immunoreactivity.

### Biochemical and western blot analysis

Protein samples from frontal cortex and hippocampus of mouse brains as well as from cell lysates were lysed using a 1x RIPA lysis buffer with SIGMAFAST™ Protease Inhibitor Cocktail and phosphatase inhibitor for the soluble fractions. After ultracentrifugation at 90,720 × *g* for 45 min at 4 °C, the supernatant was kept as the soluble fraction. The pellet was then sonicated in the presence of 70% formic acid and ultracentrifuged at 90,720 × *g* for 45 min at 4 °C. The resulting formic acid-extracted supernatant representing the insoluble fraction was neutralized with 6 N sodium hydroxide. Aβ_1–40_ and Aβ_1–42_ levels both in soluble and insoluble fractions were assayed using the method described in the manual of Human Beta-Amyloid (1–40) ELISA Kit and Human Beta-Amyloid (1–42) ELISA Kit, respectively. The monoclonal antibody BAN50, specifically detects the N-terminal of human Aβ_1–16_, was used to capture Aβ_1–40_ and Aβ_1–42_ in samples. Captured Aβ_1–40_ and Aβ_1–42_ was recognized by BA27 F(Aβ’)2-HRP antibody, and BC05 F(Aβ’)2-HRP, respectively. Both BA27 and BC05 are mAβ that specifically detect the C-terminal of Aβ. The TMB based color development was used to assess the HRP activity, and absorbance was then measured at 450 nm. Values were presented as a percentage of Aβ_1–40_ and Aβ_1–42_ secreted relative to control.

For western blot analysis, the protein samples were quantified using Bio-Rad Protein Assay Dye Reagent. Equal amounts of proteins were resolved by electrophoresis on SDS-PAGE gels and transferred to a PVDF Immobilon-FL membrane (EMD Millipore, Catalog # IPFL00010). The PVDF membrane was incubated with blocking buffer (Rockland, Catalog # MB-070) at room temperature for one hr. The membrane was incubated with primary antibody at 4 °C overnight. The following primary antibodies were used in the study: MCU, MCUB, MICU1, MICU2, VDAC, ETC respiratory chain complexes, anti-APP N-terminal raised against amino acids 66–81 for total APP 22C11, BACE1, ADAM-10, PS1 (Sigma, P7854, 1:500), Nicastrin (NCT, 3632S, cell signaling #14997, 1:1000), APH1 (Millipore AB9214, 1:500), total tau HT7, phospho-tau (pThr231, AT180), phospho-Tau (Ser202, Thr205, AT8), phospho-tau (pThr181, AT270), phospho-tau (pS396, PHF13), beta-Tubulin, SYP dilution, PSD-95 dilution, GFAP. After incubation with primary antibody, the membrane was washed three times with TBS-T (TBS containing 0.1% Tween 20) for 10 min each and then incubated with specific secondary antibody for 1 h at room temperature. Licor IR secondary antibodies were used at dilutions 1:10,000. All blots were imaged on a Licor Odyssey system. All full-length western blots are available in the Appendix.

#### Memory tests

Mice at 6, 9, 12, and 15 months of age were assessed for spatial working memory in the Y-maze and hippocampal-dependent associative learning memory in fear conditioning assay. In our study, we used the same cohort of mice for the behavioral experiments, testing them at multiple time points. This longitudinal approach allows us to track changes within the same subjects over time, providing valuable insights into the progression of behavioral changes. The use of the same mice across different time points impacts the statistical analysis as it introduces intra-subject correlations. To account for this, we employed repeated measures ANOVA which is specifically designed for analyzing data from repeated measurements on the same subjects.

### Y-maze

We assessed spatial working memory by measuring spontaneous alternation in a Y-maze. In this assay, test animals were placed in the center of the Y-shaped maze (San Diego Instruments, 32 cm (long) 610 cm (wide) with 26-cm walls) for 5 min and the total number of arms entered, as well as the sequence of entries, were recorded. A spontaneous alternation was defined when a mouse enters a different arm of the maze in each of three consecutive arm entries (i.e., 1, 2, 3, or 2, 3, 1, or 3,1,2). Spontaneous alternation % was then calculated with the following formula: total alternation number/total number of entries-2 × 100. Prior to initial use, the arms of the maze were clearly designated as ‘1’, ‘2’ & ‘3’, and the maze was always wiped clean with 70% ethanol, followed by water between each animal.

### Fear conditioning

The fear-conditioning was performed in a fear-conditioning apparatus (StartFear, Panlab Harvard Apparatus, 25 cm height × 30 cm width × 25 cm depth). The chamber was consisted of black methacrylate walls, a transparent front door, a light, a speaker, and a removable grid stainless-steel rod floor (3.2 mm diameter, 4.7 mm apart) through which a foot shock was administered. Automated fear conditioning FREEZING software was used to record and analyze signals generated by the animal movement throughout the procedure. Before each test, the chamber was cleaned with 70% ethanol, followed by water. In this test, mice were trained and tested on two consecutive days. On day 1 (training phase), each mouse was placed in the chamber and underwent three cycles of 30 s of sound and 10 s of electric shock (1.5 mA) within a 6-minute time interval. On day 2, the mouse returned to the same chamber without receiving electric shock or hearing the sound (contextual recall), and freezing behavior was recorded for 5 min. Two hours later, the animal spent 6 min in the same chamber but in an altered chamber environment, for example, different flooring, walls, smells, and lighting, and heard the cued sound for 30 s (cued recall), but without a foot shock. Freezing % was equal to (freezing time/total time) ×  100%. All procedures were coordinated via PACKWIN (Panlab, Harvard Apparatus, USA) on a computer connected to the device, and data analysis was performed using the same software.

### Assessment of reactive oxygen species production

We employed Dihydroethidium (DHE) staining for in vivo detection of superoxide levels, as described (Jadiya et al, 2019). In brief, we freshly prepared cortical, and hippocampal slices from mice brains and stained them with 20 μM DHE for 30 min at 37 °C and imaged at 518/excitation and 606/emission on a Carl Zeiss 710 confocal microscope. To examine the total cellular ROS, cells from all groups were loaded with 5 μM CellROX Green Reagent for 30 min at 37 °C. CellROX exhibits a strong fluorogenic signal upon oxidation. The fluorescence at 485/excitation and 520/ emission was measured using a Tecan Infinite M1000 Pro plate reader. Next, we measured mitochondrial superoxide production using MitoSOX staining. Cells were loaded with 10 μM MitoSOX Red for 45 min at 37 °C and imaged at 490/20 excitation and 585/40 emission on Carl Zeiss 510 confocal microscope. All images were quantified for fluorescent optical density using ImageJ.

### Membrane rupture and cell viability assay

We used SYTOX Green nucleic acid stain to examine membrane rupture and Cell Titer Blue to evaluate cell viability. SYTOX Green is a membrane-impermeable fluorescent stain that becomes permeable with compromised plasma membranes and intercalates in DNA and increases fluorescence. The Cell Titer Blue assay uses the indicator dye resazurin to measure cells’ metabolic capacity that an indicator of cell viability. In a 96-well plate, equal numbers of cells from all groups were treated with Ionomycin (2–10 µM) and an oxidizing agent tert-butyl hydroperoxide solution (TBH: 10–50 µM) for 24 h. On the day of the experiment, SYTOX green dye was added to wells at 1 μM final concentration for 15 min at 37 °C. Fluorescence was measured using a Tecan Infinite M1000 Pro plate reader at 504/excitation and 523/emission. To measure the number of viable cells, Cell Titer Blue Reagent (10 µl/well in 96-well plates) was added directly to each well and incubated at 37 °C for 2 h. Fluorescence was measured using a Tecan Infinite M1000 Pro plate reader at 560/ excitation and 590/ emission. Data were normalized to vehicle control to avoid any differences in cell numbers between the groups.

### Evaluation of _m_Ca^2+^ retention capacity and content

Assessment of _m_Ca^2+^ uptake and content were performed as reported previously (Jadiya et al, 2019). Cells (2 × 10^6^) from all groups were washed in Ca^2+^ -free DPBS and resuspended in an intracellular-like medium (120 mM KCl, 10 mM NaCl, 1 mM KH2PO4, 20 mM HEPES-Tris) containing thapsigargin (3 μM) to block the SERCA pump, digitonin (80-μg/ml), protease inhibitor, and succinate (10 μM) at pH 7.2. All solutions were cleared with Chelex 100 to remove trace Ca^2+^. To measure _m_Ca^2+^ uptake, cells were gently stirred and loaded with the ratiometric reporters Fura-FF at concentration of 1 μM to monitor extra-mitochondrial Ca^2+^. To monitor mitochondrial membrane potential (Δψ), JC1 was added at 20 s. Fluorescence signals were monitored at 340- and 380-nm excitation/510-nm emission for Fura-FF to calculate ratiometric changes and at 490 nm excitation/535 nm emission for the monomer 570 nm excitation/595 nm emission for the J-aggregate of JC-1. After acquiring baseline recordings, a 10 µM Ca^2+^ bolus was added at 400 s. Clearance of extra-mitochondrial Ca^2+^ was representative of _m_Ca^2+^ uptake. At 550 s, a protonophore, 10 μM FCCP (a protonophore) was added to uncouple the Δψ_m_ and release matrix-free-Ca^2+^. All experiments (three replicates) were conducted at 37 °C and recorded on a PTI spectrofluorometer (Delta RAM, Photon Technology Int.). The _m_Ca^2+^ uptake rate was recorded over 50 s post Ca^2+^ bolus. To evaluate _m_Ca^2+^ content, permeabilized cells from all the groups were loaded with Fura-2 for ratiometric monitoring of extra-mitochondrial Ca^2+^ using a fluorescent spectrofluorometer. Upon reaching a steady-state recording, FCCP was used to collapse ΔΨ_m_ and liberate all matrix-free-Ca^2+^. To measure basal _m_Ca^2+^ in intact cells, cells were transfected with mitochondrial genetically encoded calcium indicator, CMV-mito-GEM-GECO1 (Addgene, plasmid #32461) using FuGENE HD (Promega), and imaged 48 h after using a Carl Zeiss 900 confocal microscope (405 nm laser excitation and blue (470 nm) and green (535 nm) emission). Obtained images were analyzed in Fiji 2.14.0 using custom macros (https://sites.imagej.net/Theweave) for background subtraction. Individual cells were masked and mean gray values were exported and further processed in Excel and GraphPad Prism 10.4.0. _m_Ca^2+^ was calculated as the ratio of blue to green emission.

To assess _m_Ca^2+^ retention capacity in vivo, we next isolated mitochondria from frontal cortex of mice and performed Calcium Green 5 n to monitor extra-mitochondrial Ca^2+^ as described previously(Jadiya et al, 2019). After baseline recordings at 400 s, 5 µM- repetitive Ca^2+^ boluses were added and fluorescent signal at (488Ex/530Em) was measured using plate reader.

#### Autophagy assay

To estimate basal autophagy, tandem-tagged LC3-EGFP-RFP assay was used. Cells were transfected with tandem-tagged LC3-EGFP-RFP (Addgene, plasmid #21074) using FuGENE HD (Promega), and imaged 48 h after using a Carl Zeiss 900 confocal microscope using GFP and RFP settings. Obtained images were used to count the number of GFP^+^RFP^+^ and GFP^-^RFP^+^ dots per cell representing autophagosomes and autolysosomes, respectively. Percentage of autolysosomes to the total number of dots were calculated.

#### Estimation of Aβ accumulation

To estimate amyloid accumulation, HiLyte™ Fluor 488-labeled Aβ (Anaspec, AS-60479-01) was added to cell media at 1 µM. The cells were examined 24 h after using a Carl Zeiss 900 confocal microscope using GFP settings. Obtained images were analyzed in Fiji 2.14.0 using custom macros (https://sites.imagej.net/Theweave) for background subtraction. Individual cells were masked and mean gray values were exported and further processed in Excel and GraphPad Prism 10.4.0.

#### Detection of protein aggregates

Protein aggregates were detected as described previously (Jadiya et al, 2019). Briefly, for determination of misfolded protein aggregates, cells were fixed with 4% paraformaldehyde at RT for 15 min and, permeabilized in PBST (0.15% TritonX-100 in PBS) at RT for 15 min. Cells were then stained with Proteostat aggresome detection dye at RT for 30 min and Hoechst 33342 nuclear stain, using the method described in the manual (Enzo Life Science Inc., Farmingdale, NY, USA). Proteostat (Enzo), a molecular rotor dye that becomes fluorescent when binding to the β-sheet structure of misfolded proteins. All components of the Proteostat aggresome detection kit were prepared according to the manufacturer’s instructions. Aggregated protein accumulation was detected using a Carl Zeiss 900 confocal microscope (standard red laser set for the aggresome signal and DAPI laser set for the nuclear signal imaging). Obtained images were analyzed in Fiji 2.14.0 using custom macros (https://sites.imagej.net/Theweave) for background subtraction. Individual cells were masked and mean gray values were exported and further processed in Excel and GraphPad Prism 10.4.0.

#### Mitochondrial bioenergetics

Mitochondrial bioenergetics was estimated as described previously (Jadiya et al, 2019). Briefly cells were subjected to OCR measurement at 37 °C in an Agilent Seahorse XF Pro analyzer (Agilent). Cells (4 × 10^4^) were plated in regular medium and next day assayed in bicarbonate-free phenol red-free DMEM media (Corning 90-113-PB) supplemented with 10 mM glucose, 2 mM L-glutamine, 1 mM sodium pyruvate, and 5 mM HEPES pH 7.4. During the assay cells were sequentially exposed to oligomycin (2 µM), FCCP (2 µM), and rotenone plus antimycin A (1 µM). Quantification of basal respiration (base OCR—non-mito respiration (post-Rot/AA), ATP-linked respiration (post-oligo OCR−base OCR), Max respiratory capacity (post-FCCP OCR−post-Rot/AA), Spare respiratory capacity (post-FCCP OCR−basal OCR) and Proton leak (post-Oligo OCR−post-Rot/AA OCR) was performed.

### Transmission electron microscopy

Mice were anesthetized and perfused with PBS for 5 min and then with a fixative solution of 2% paraformaldehyde/2% glutaraldehyde in 0.1 M sodium cacodylate buffer. After dissection, intact brain was placed in a fresh solution of the fixative overnight at 4 °C under agitation. Coronal sections (100–200 µm) were cut with the vibratome and then processed the tissue slices and embed in epoxy resin over a period of 48 h. The areas of interest (cortex, three blocks per mouse) were cut out of the slices with Reichert-Jung Ultracut E ultramicrotome and mounted for ultrathin sectioning for the TEM on Athene 200 mesh thin bar formvar-carbon coated copper grids. Sections were visualized with a Zeiss Libra 120 transmission electron microscope operating at 120 kV, using a Gatan Ultrascan 1000 CCD camera. We used ImageJ software to analyze mitochondria ultrastructure and mitochondrial shape descriptors (area, perimeter, circularity, Feret’s diameter, aspect ratio). Minimum of 200 mitochondria were analyzed per mouse, *n* = 4 mice per group.

### Quantification of mitochondrial copy number

The genomic DNA was isolated from mice brain cortex tissue using the DNeasy blood and tissue kit (Qiagen 69504) according to manufacturer’s instructions. Quantification of mtDNA copy number was performed via quantitative PCR (qPCR) using the primers mitochondrial COXII gene and nuclear β-Globin (Jadiya et al, 2019).

### Statistics

Prism 6.0 GraphPad software was used for statistical analysis and to generate plots. All results are shown as mean ± SEM. All experiments were repeated at least three times and measurements were taken on distinct samples. Individual data points mean and s.e.m. were displayed in the figures. Where appropriate column analyses were performed using an unpaired, two-tailed *t* test (for two groups) or one-way ANOVA with Bonferroni correction (for groups of three or more). For grouped analyses either multiple unpaired *t* test with correction for multiple comparisons using the Holm–Sidak method or where appropriate two-way ANOVA with Tukey post hoc analysis was performed. The specific statistical tests for each experiment are indicated throughout the manuscript. Results were significant if *P* values <  0.05 (95% confidence interval).

### Study approval

Animal studies were approved by Temple University’s IACUC and followed AAALAC guidelines.

## Supplementary information

Appendix

Peer Review File

Source data Fig. 1

Source data Fig. 2

Source data Fig. 3

Source data Fig. 4

Source data Fig. 5

Figure EV1 Source Data

Figure EV2 Source Data

Figure EV3 Source Data

Figure EV4 Source Data

Figure EV5 Source Data

Raw Western Blot and Microscopy Images

Expanded View Figures

## Data Availability

All data supporting the findings of this study, including all raw data and complete statistical analysis files, are available as downloadable ZIP archives via the EMBO Journal submission system. Our study includes no large datasets deposited in public repositories. The source data of this paper are collected in the following database record: biostudies:S-SCDT-10_1038-S44318-026-00809-w.
