# Supplementary material for: Genetic ablation of neuronal mitochondrial calcium uptake impedes Alzheimer’s disease progression
Source: EMBO J. 2026 May 22;45(13):4469–91. doi: 10.1038/s44318-026-00809-w (PMC13324160; doi:10.1038/s44318-026-00809-w)
Supplement: Supplementary file 1 — Appendix [file 44318_2026_809_MOESM1_ESM.pdf]

Appendix for

“Genetic ablation of neuronal mitochondrial calcium uptake impedes disease progression in models of Alzheimer’s disease”

Table of contents

Appendix Figure S1. Full length blots for Figure 1B **(p2)**
Appendix Figure S2. Full length blots for Figure 1C **(p2)**
Appendix Figure S3. Full length blots for Figure 2G **(p2)**
Appendix Figure S4. Full length blots for Figure 3A **(p3)**
Appendix Figure S5. Full length blots for Figure 4G **(p3)**
Appendix Figure S6. Full length blots for Figure 4H **(p3)**
Appendix Figure S7. Full length blots for Figure 4I **(p3)**
Appendix Figure S8. Full length blots for Figure 5 **(p3)**
Appendix Figure S9. Full length blots for Figure EV1O**(p4)**
Appendix Figure S10. Full length blots for Figure EV2C **(p4)**
Appendix Figure S11. Full length blots for Figure EV3A**(p4)**
Appendix Figure S12. Full length blots for Figure EV3E **(p5)**
Appendix Figure S13. Full length blots for Figure EV3L **(p5)**
Appendix Figure S14. Full length blots for Figure EV5M **(p5)**
Appendix Figure S15. Full length blots Figure EV5P **(p6)**

Appendix Figure S1. Full length blots for Figure 1B

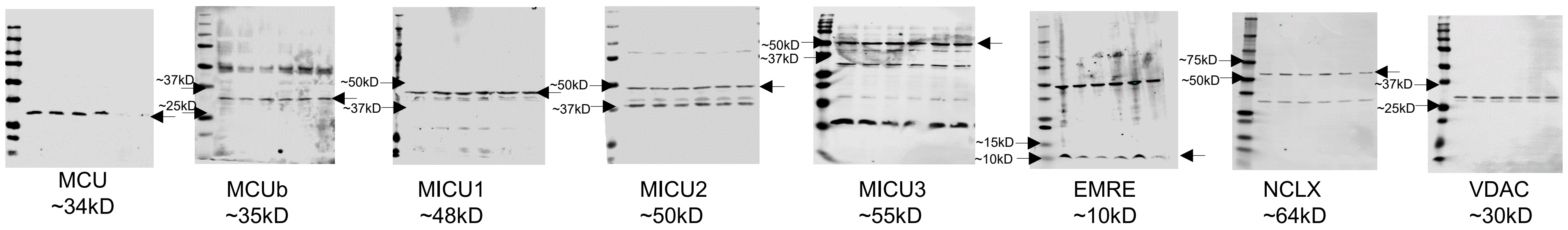

Appendix Figure S2. Full length blots for Figure 1C

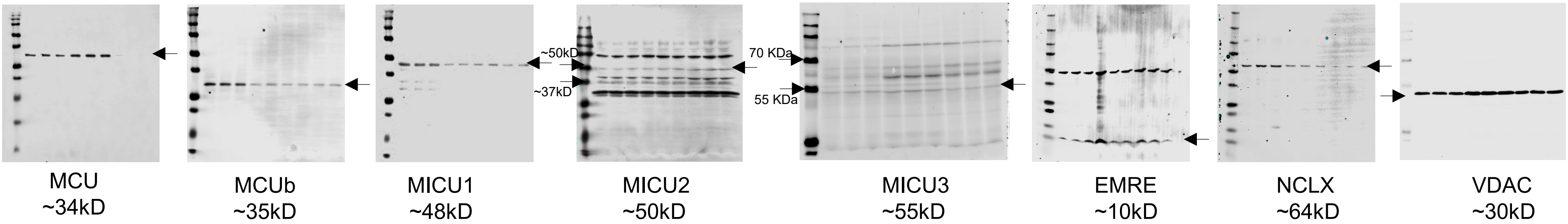

Appendix Figure S3. Full length blots for Figure 2G

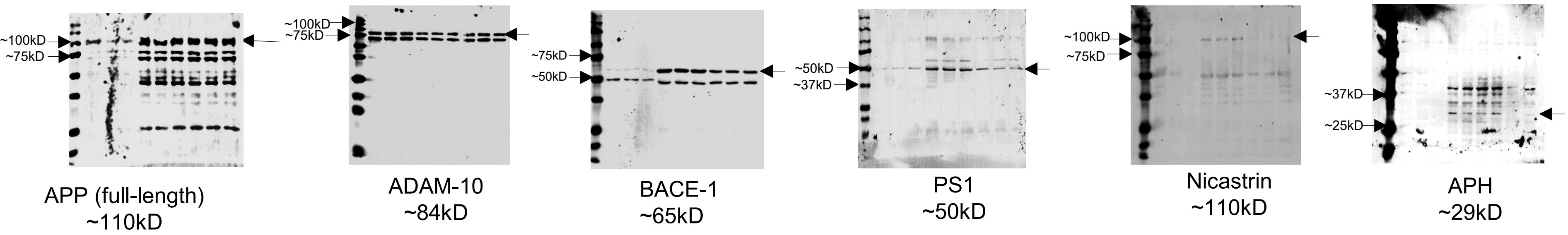

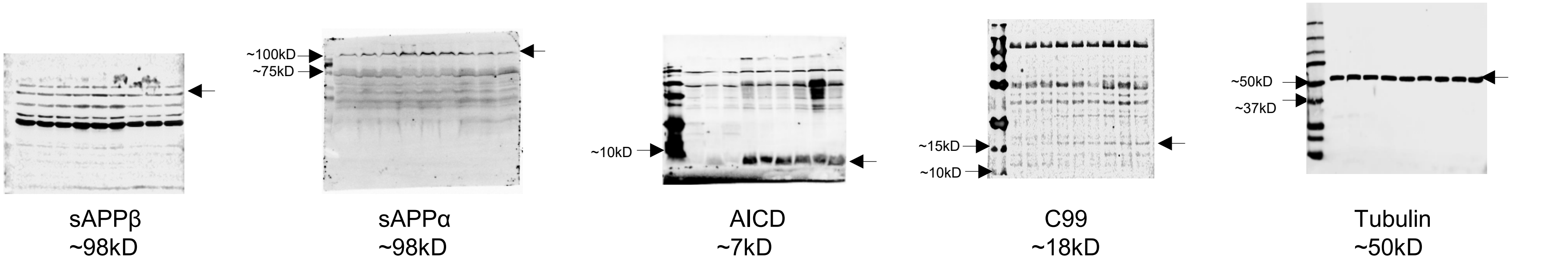

Appendix Figure S4. Full length blots for Figure 3A

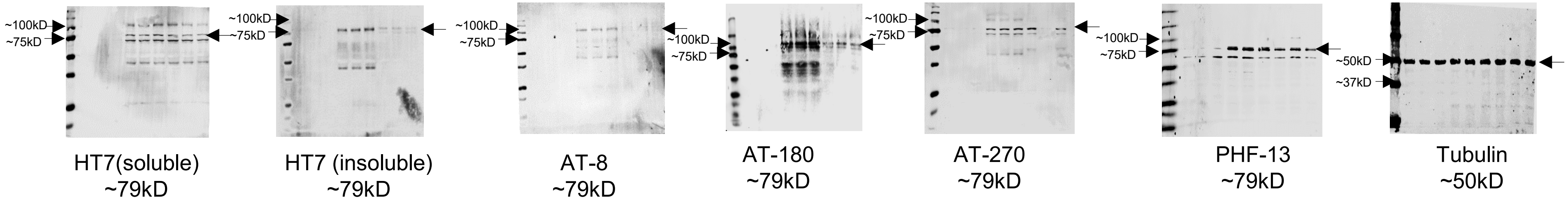

Appendix Figure S5. Full length blots for Figure 4G

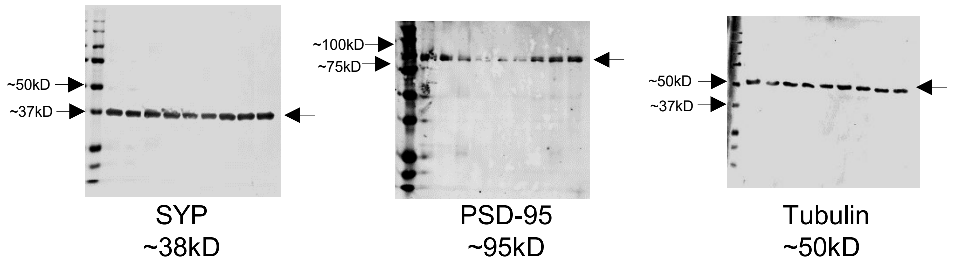

Appendix Figure S6. Full length blots for Figure 4 H

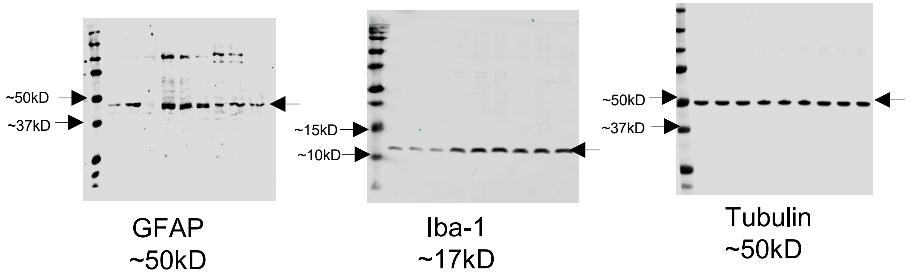

Appendix Figure S7. Full length blots for Figure 4 I

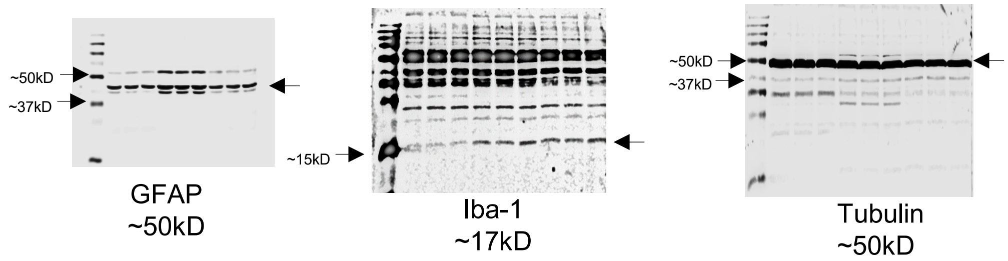

Appendix Figure S8. Full length blots for Figure 5

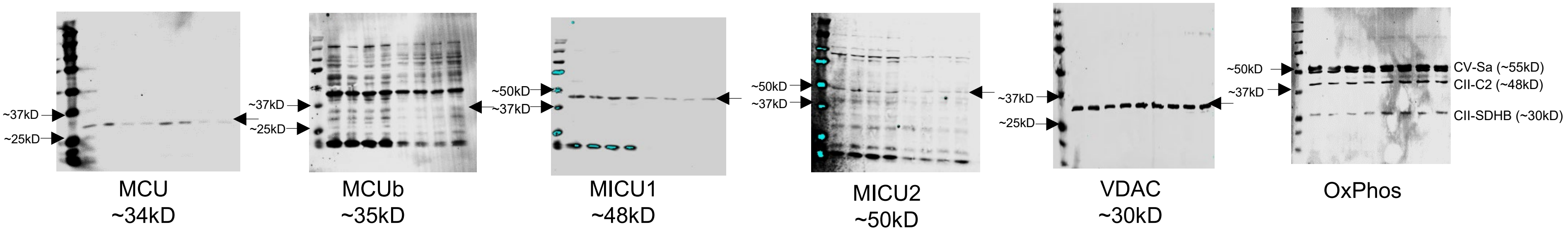

Appendix Figure S9. Full length blots for Figure EV1O

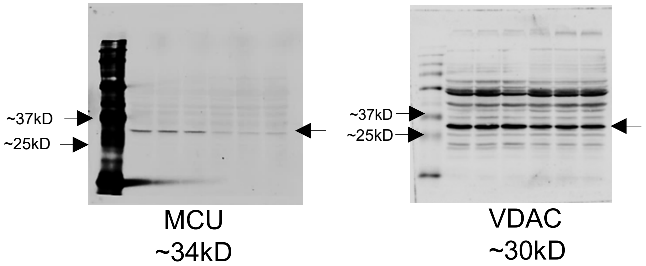

Appendix Figure S10. Full length blots for Figure EV2C

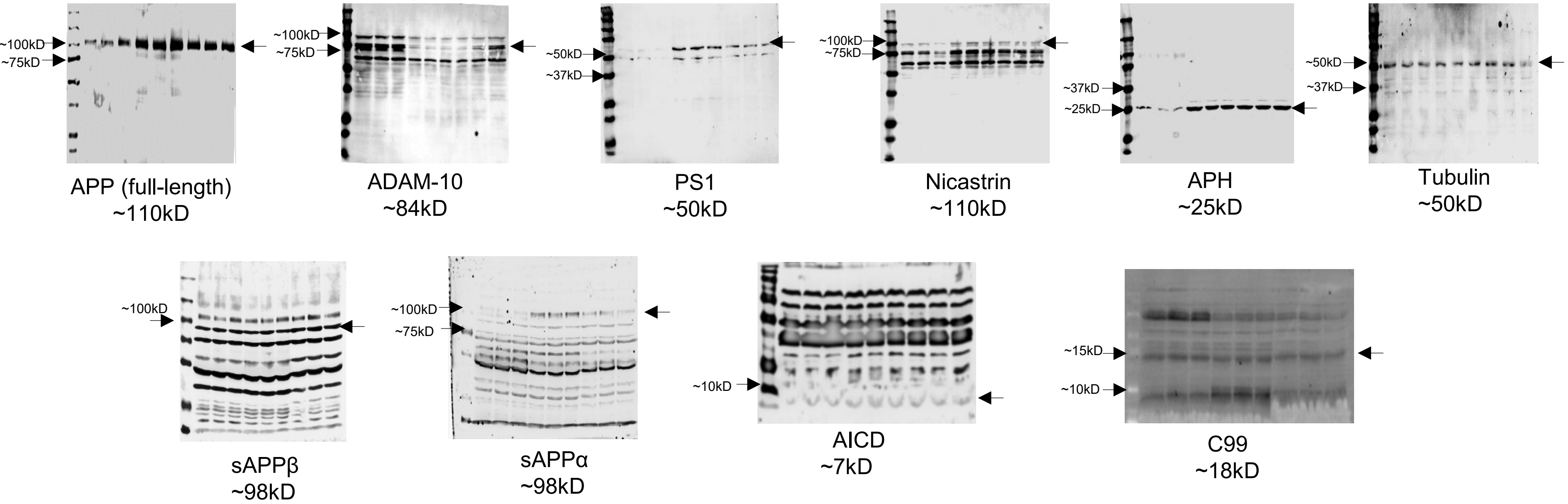

Appendix Figure S11. Full length blots for Figure EV3A

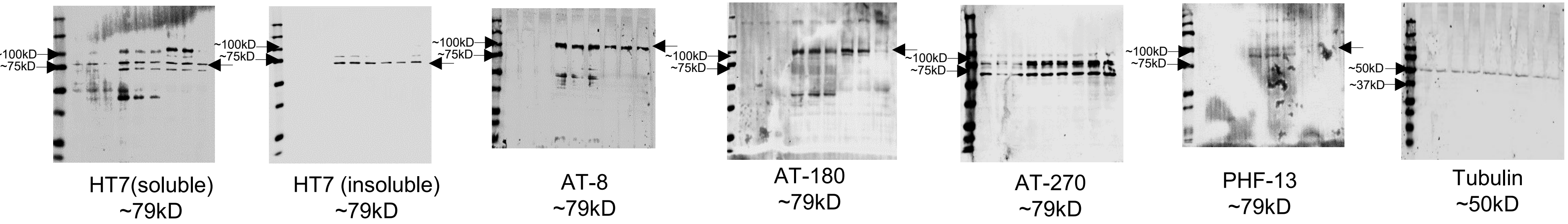

Appendix Figure S12. Full length blots for Figure EV3E

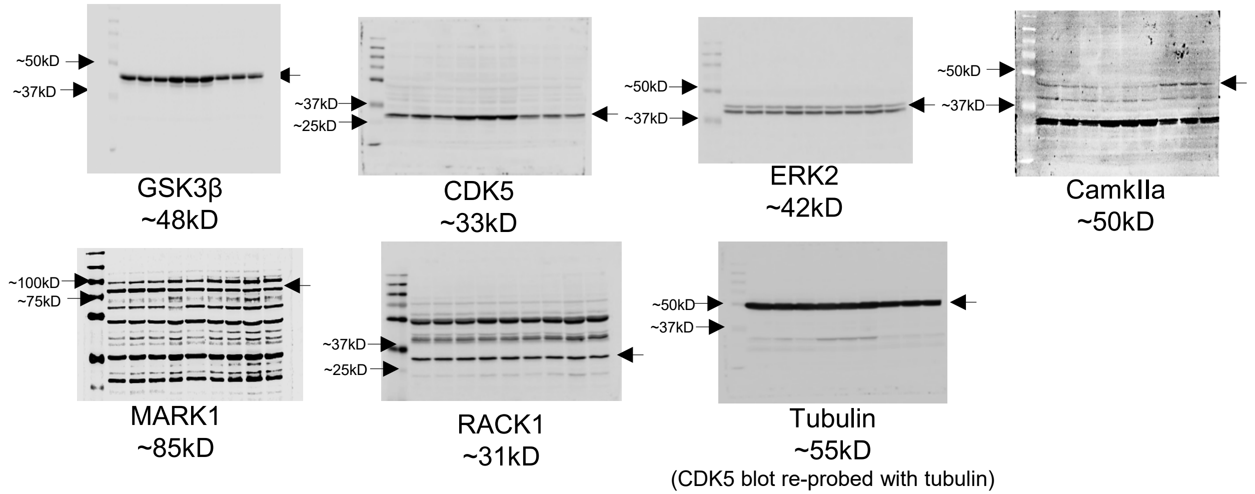

Appendix Figure S13. Full length blots for Figure EV3L

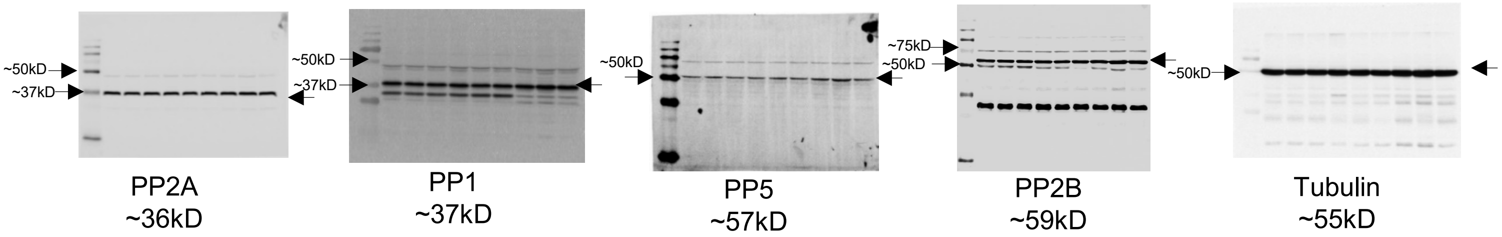

Appendix Figure S14. Full length blots for Figure EV5M

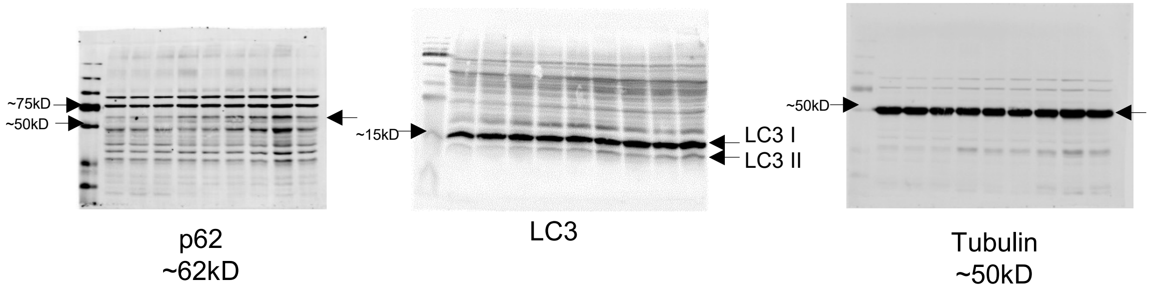

Appendix Figure S15. Full length blots for Figure EV5P

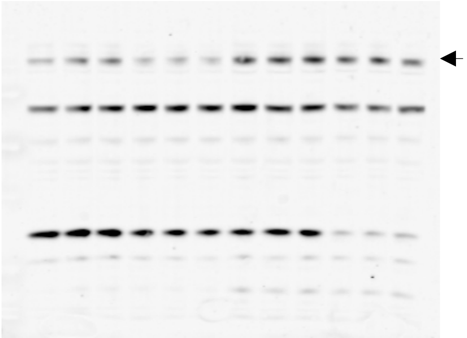

NCT
~110 kDa

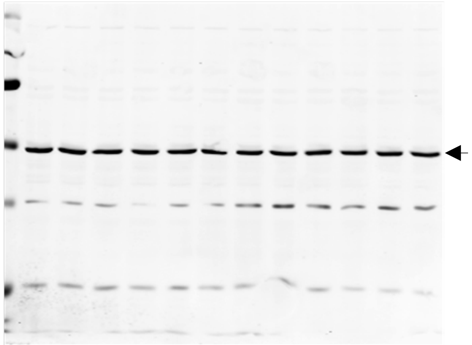

PS-1
~48 kDa

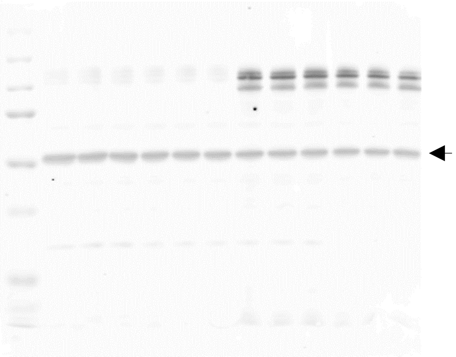

Tubulin
~48 kDa

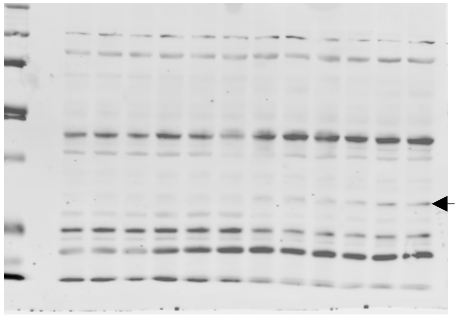

APH
~29 kDa

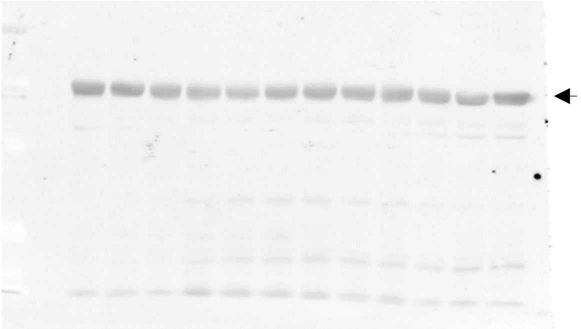

Tubulin
~48 kDa
