# Supplementary figures and images for: Genetic ablation of neuronal mitochondrial calcium uptake impedes Alzheimer’s disease progression (part 1 of 2)
Source: EMBO J. 2026 May 22;45(13):4469–91. doi: 10.1038/s44318-026-00809-w (PMC13324160; doi:10.1038/s44318-026-00809-w)

## Slide 1
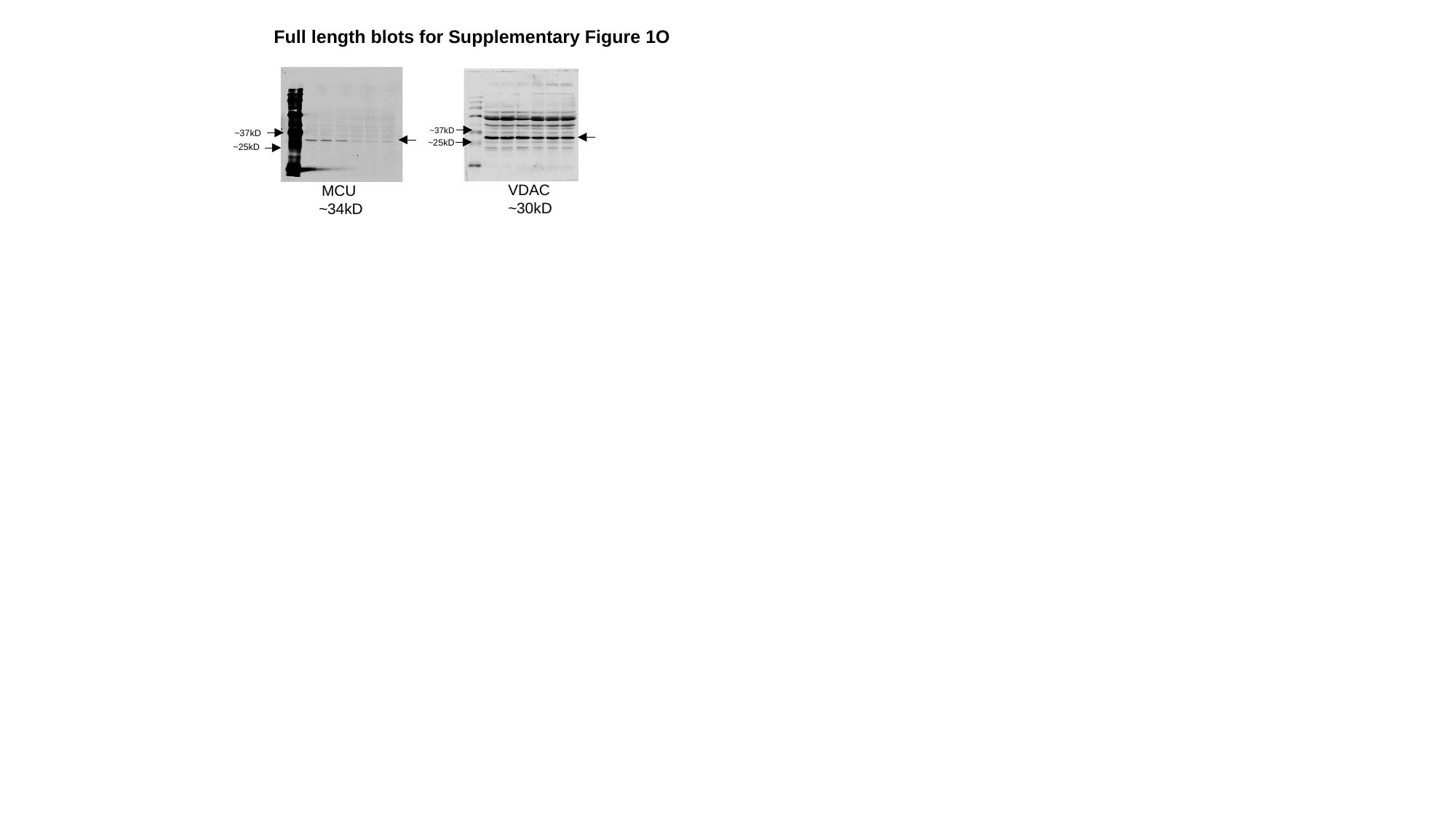

Full length blots for Supplementary Figure 1O
~37kD
VDAC
~30kD
MCU
~34kD
~37kD
~25kD
~25kD

Supplement: Supplementary file 8 — Figure EV1 Source Data [file 44318_2026_809_MOESM8_ESM.zip › Source data for Figure EV1/EV1O.pptx]

## Slide 1
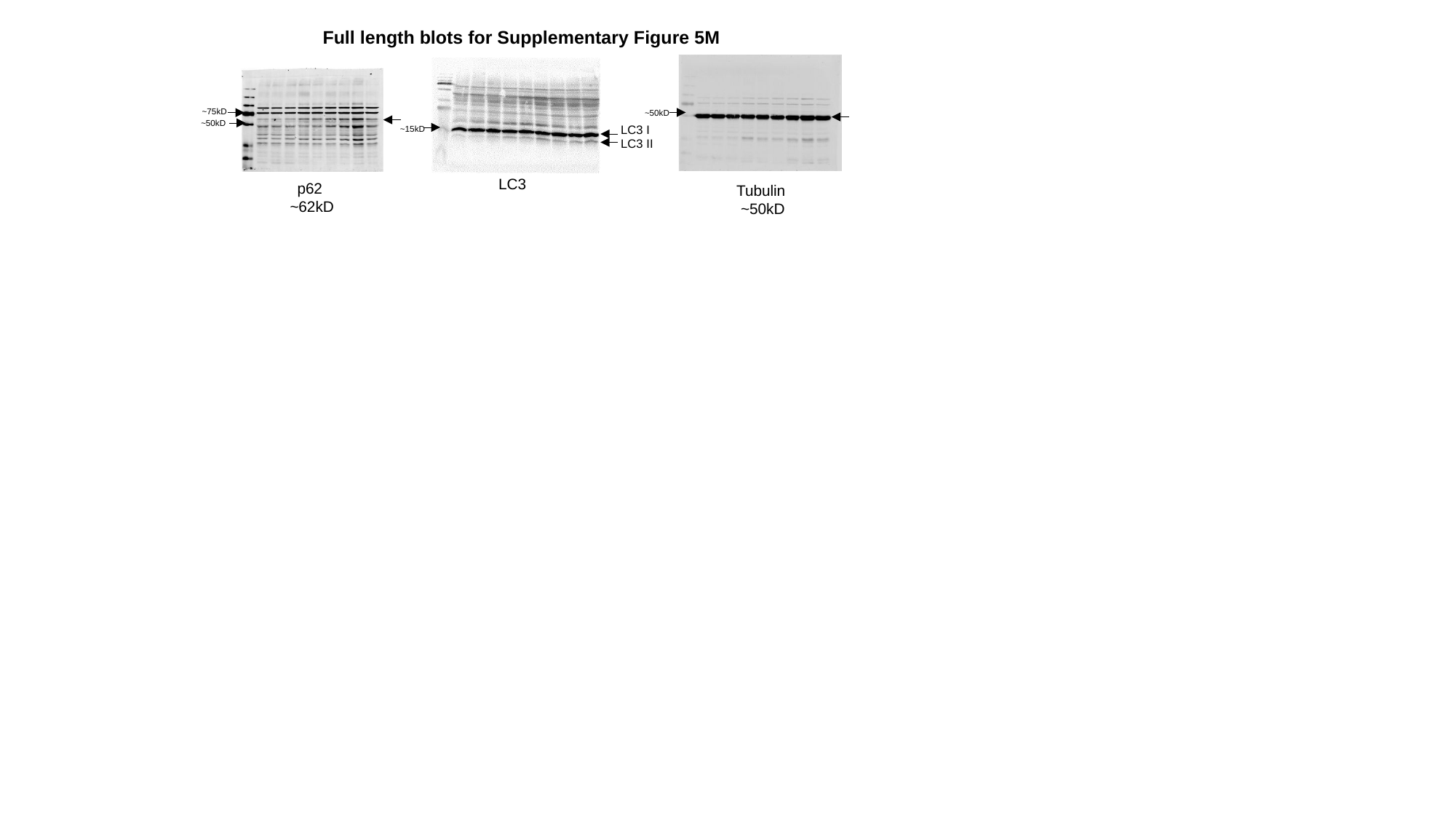

Full length blots for Supplementary Figure 5M
~50kD
~15kD
LC3
p62
~62kD
Tubulin
~50kD
~75kD
~50kD
LC3 I
LC3 II

Supplement: Supplementary file 12 — Figure EV5 Source Data [file 44318_2026_809_MOESM12_ESM.zip › Source data for Figure EV5/EV5M.pptx]

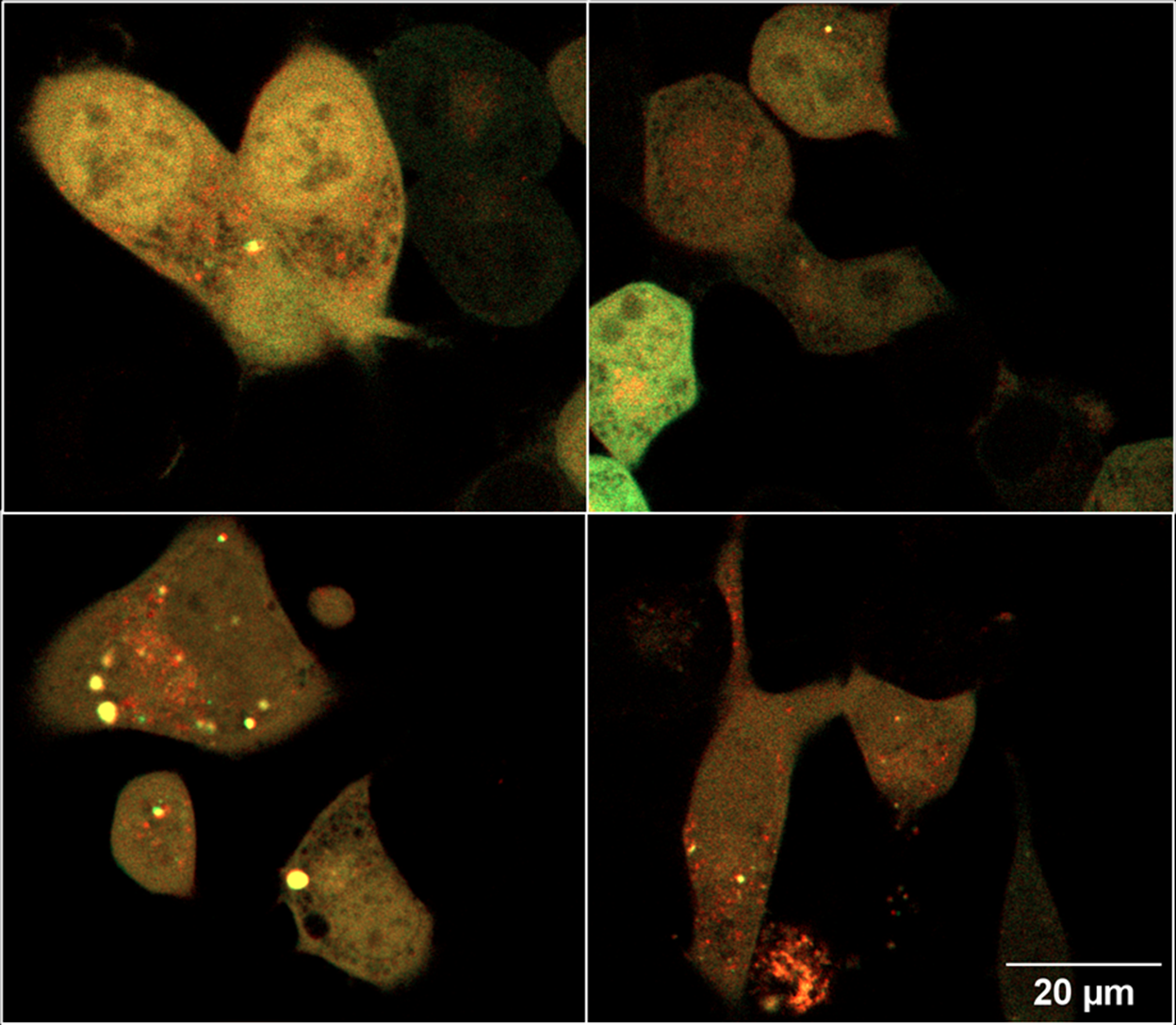

Supplement: Supplementary file 13 — Raw Western Blot and Microscopy Images [file 44318_2026_809_MOESM13_ESM.zip › SD_Images/SD 5N/5N.tif]

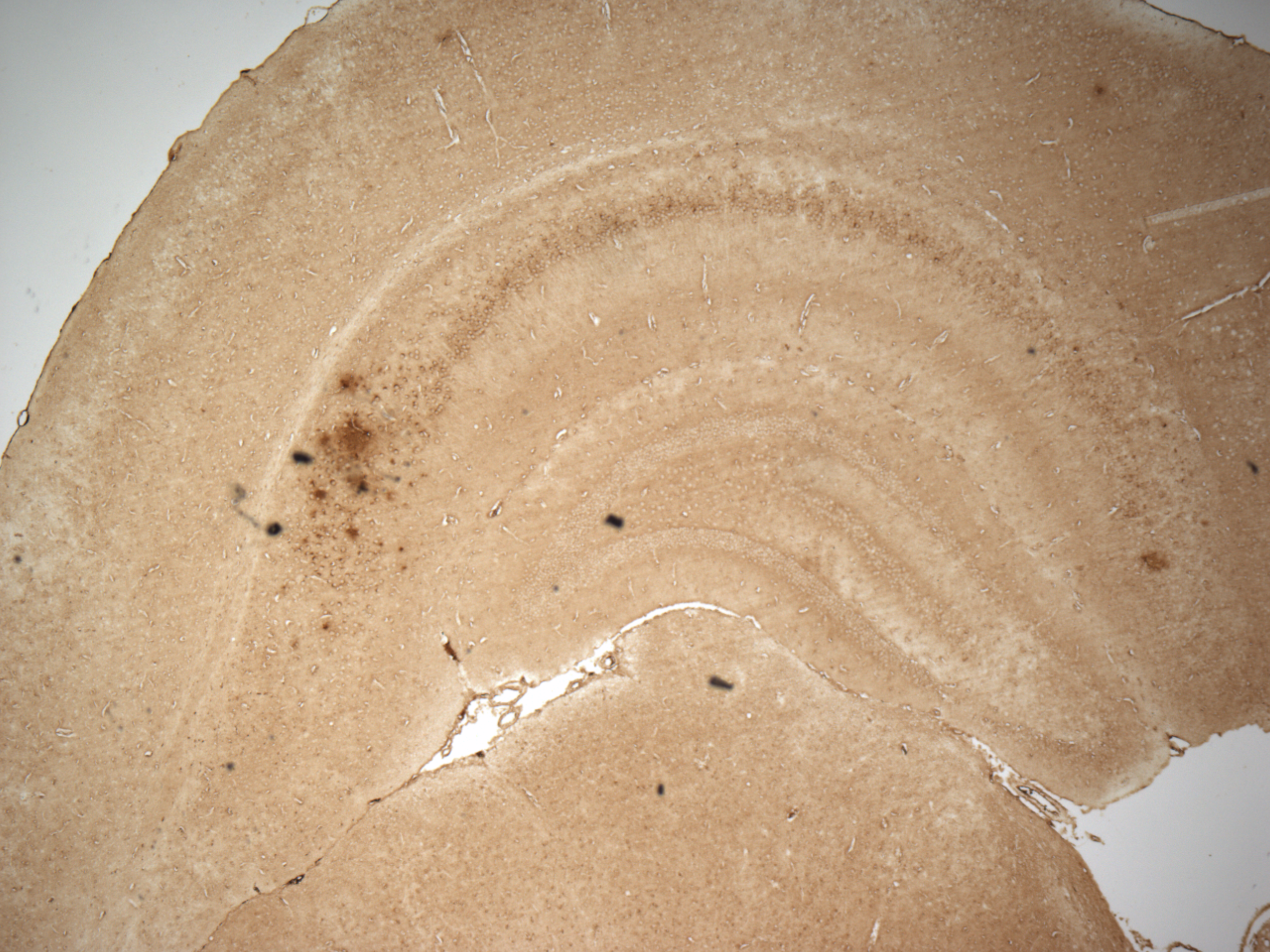

Supplement: Supplementary file 13 — Raw Western Blot and Microscopy Images [file 44318_2026_809_MOESM13_ESM.zip › SD_Images/SD Fig 2E/3xTg-AD x Camk2a-Cre -4x (imaged rotated horizontal).TIF]

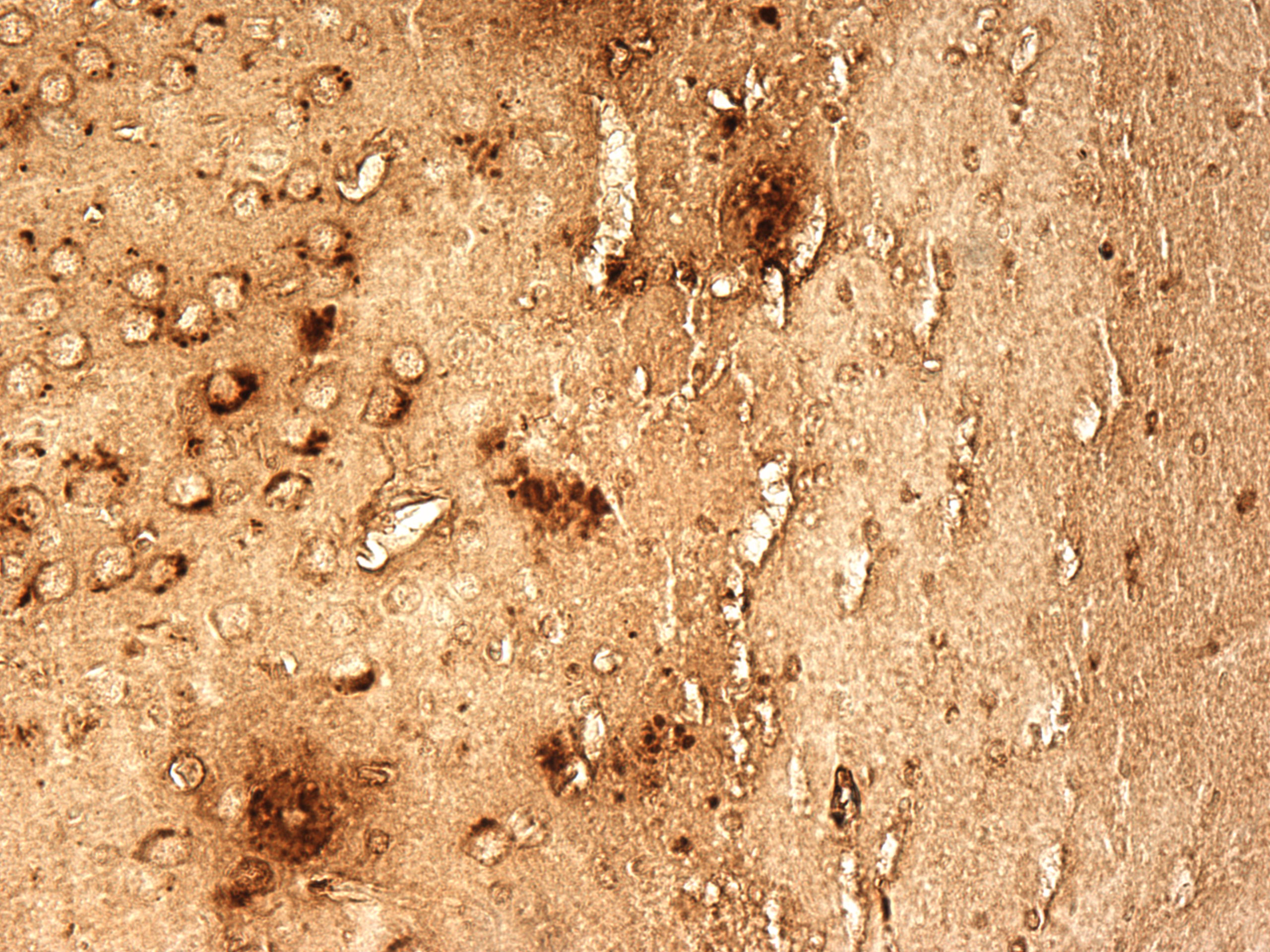

Supplement: Supplementary file 13 — Raw Western Blot and Microscopy Images [file 44318_2026_809_MOESM13_ESM.zip › SD_Images/SD Fig 2E/3xTg-AD x Camk2a-Cre-40x.TIF]

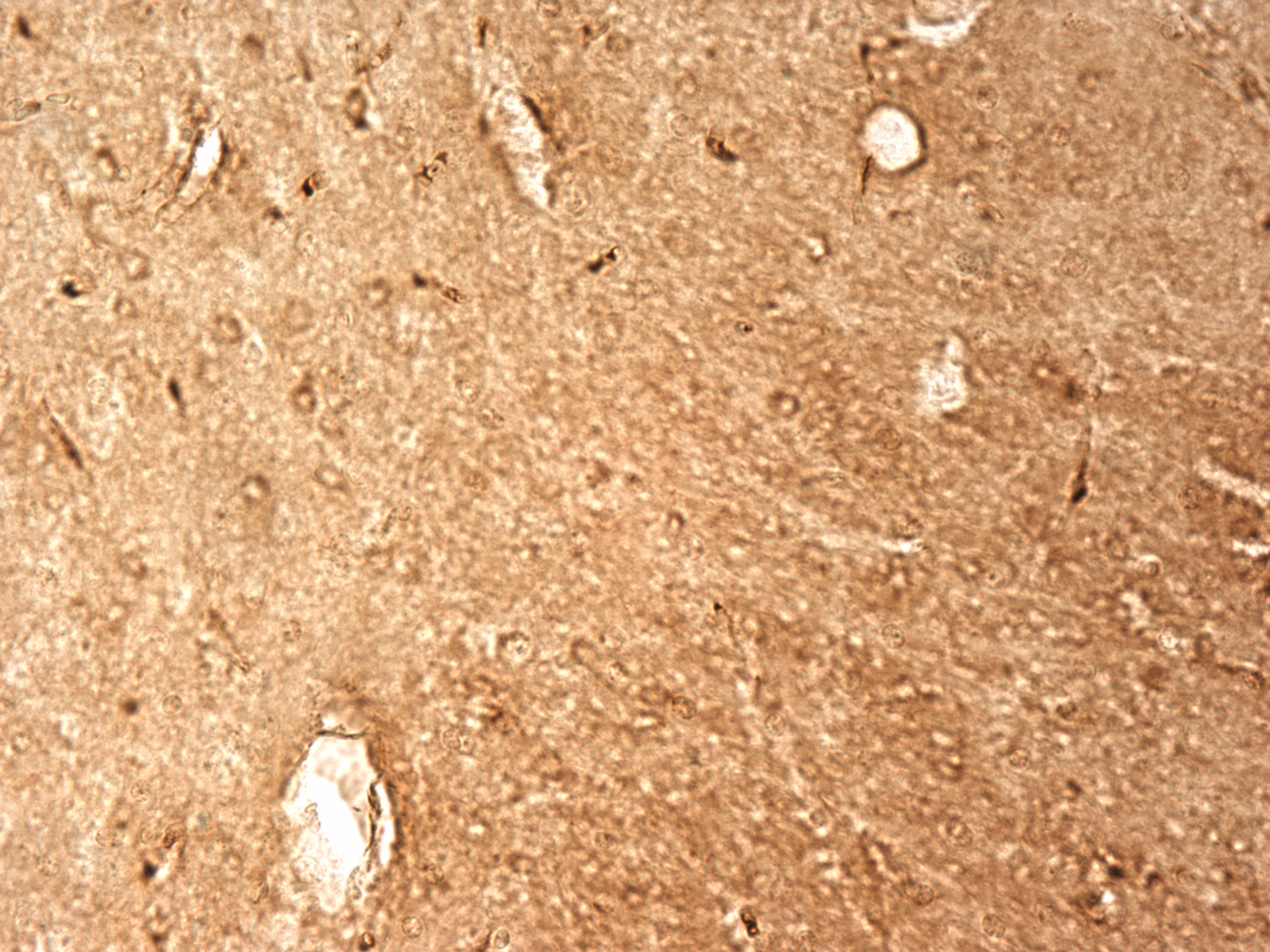

Supplement: Supplementary file 13 — Raw Western Blot and Microscopy Images [file 44318_2026_809_MOESM13_ESM.zip › SD_Images/SD Fig 2E/3xTg-AD x Mcuflfl x Camk2a-Cre- 40x.TIF]

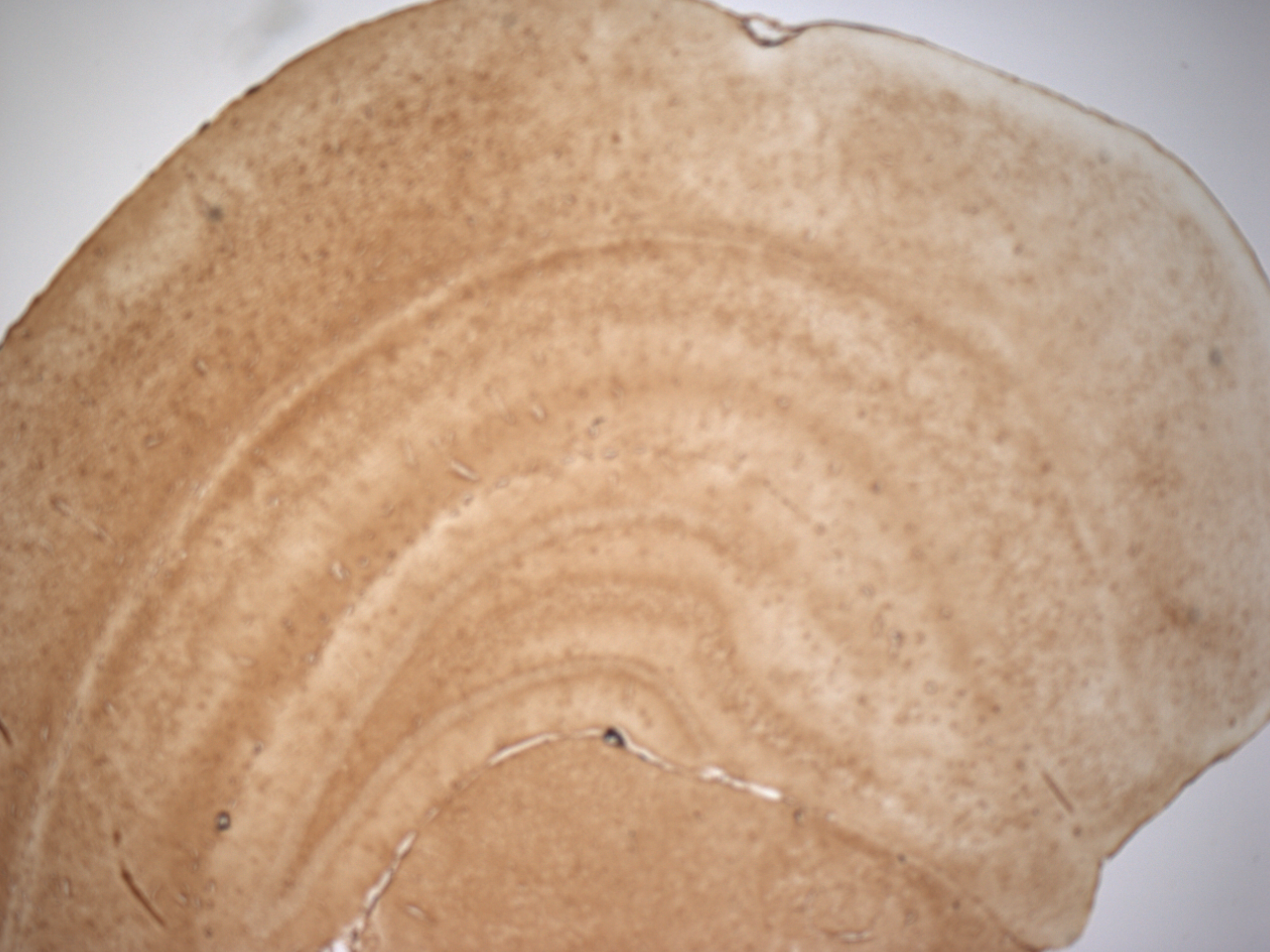

Supplement: Supplementary file 13 — Raw Western Blot and Microscopy Images [file 44318_2026_809_MOESM13_ESM.zip › SD_Images/SD Fig 2E/3xTg-AD x Mcuflfl x Camk2a-Cre- 4x.TIF]

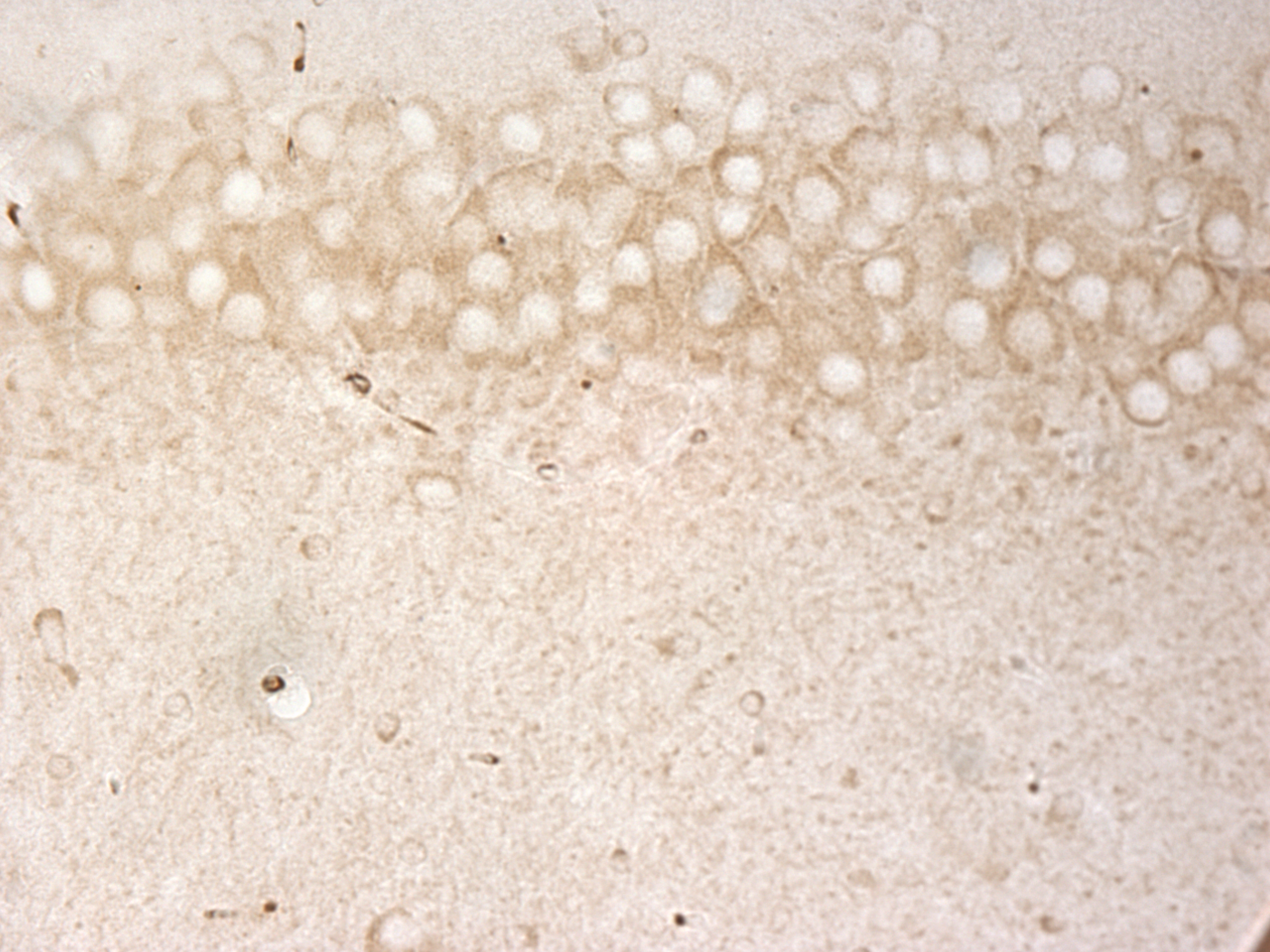

Supplement: Supplementary file 13 — Raw Western Blot and Microscopy Images [file 44318_2026_809_MOESM13_ESM.zip › SD_Images/SD FIG 3G/3xTg-AD x Camk2a-Cre PHF13.TIF]

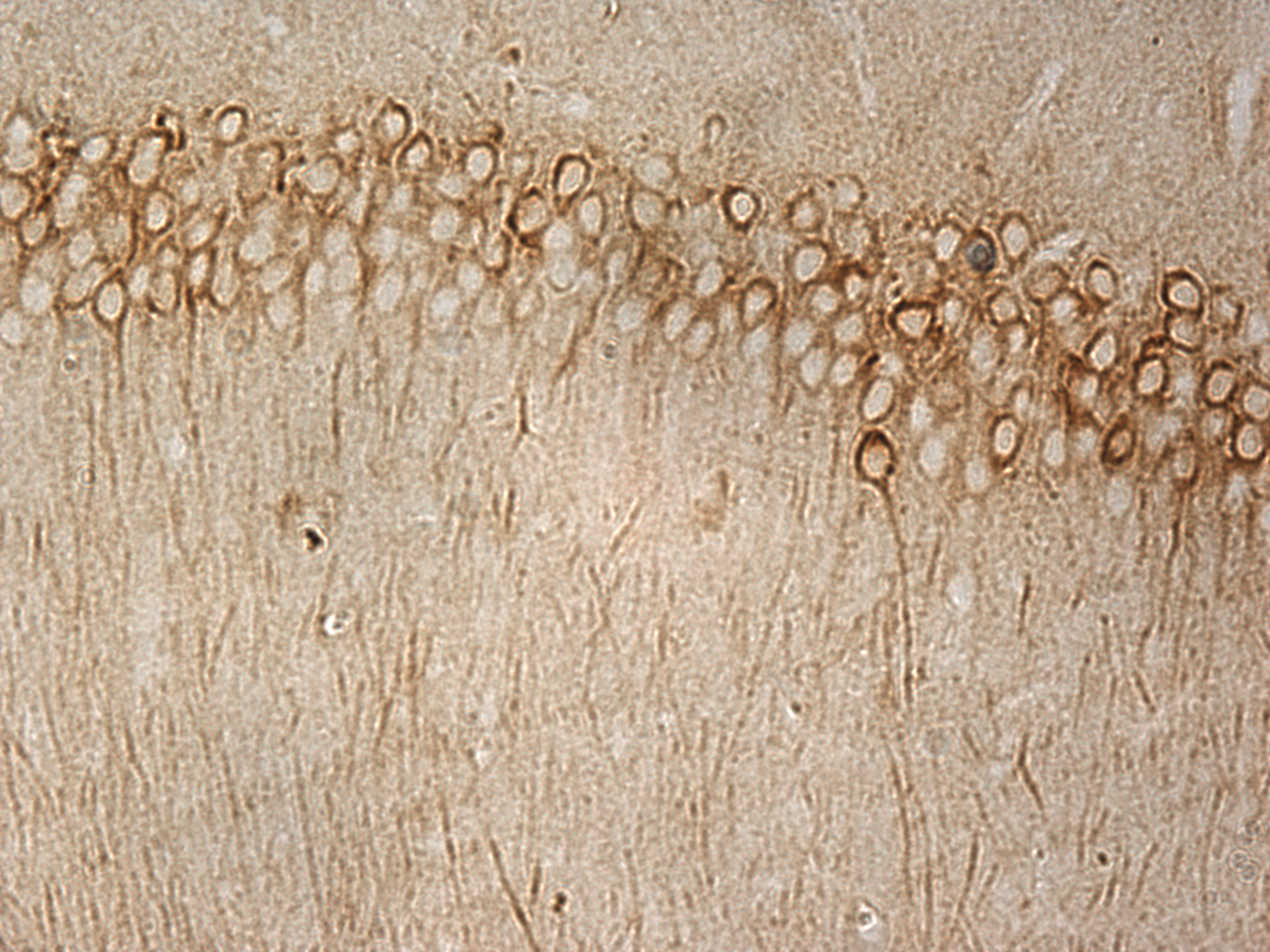

Supplement: Supplementary file 13 — Raw Western Blot and Microscopy Images [file 44318_2026_809_MOESM13_ESM.zip › SD_Images/SD FIG 3G/3xTg-AD x Camk2a-Cre AT180.TIF]

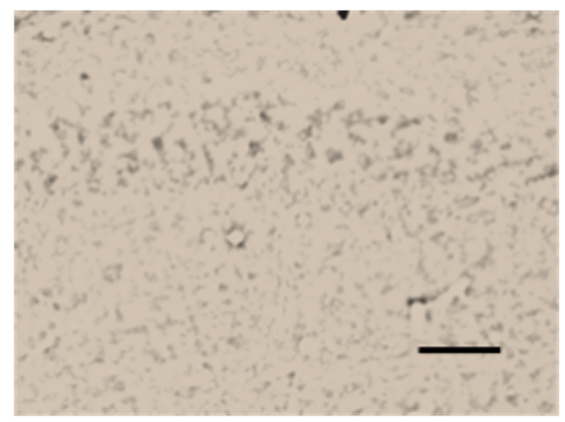

Supplement: Supplementary file 13 — Raw Western Blot and Microscopy Images [file 44318_2026_809_MOESM13_ESM.zip › SD_Images/SD FIG 3G/3xTg-AD x Camk2a-Cre AT270.tif]

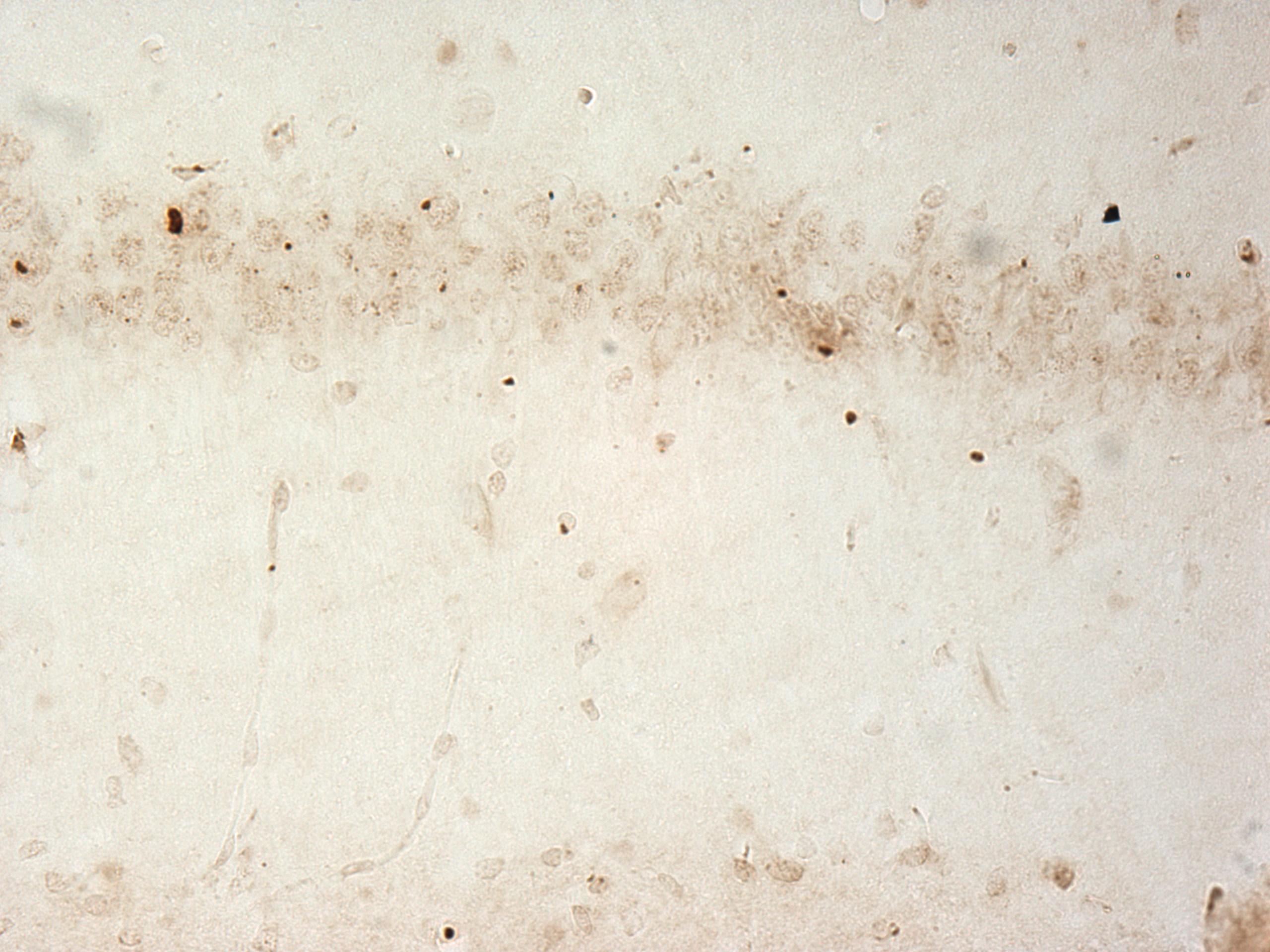

Supplement: Supplementary file 13 — Raw Western Blot and Microscopy Images [file 44318_2026_809_MOESM13_ESM.zip › SD_Images/SD FIG 3G/3xTg-AD x Camk2a-Cre AT8.TIF]

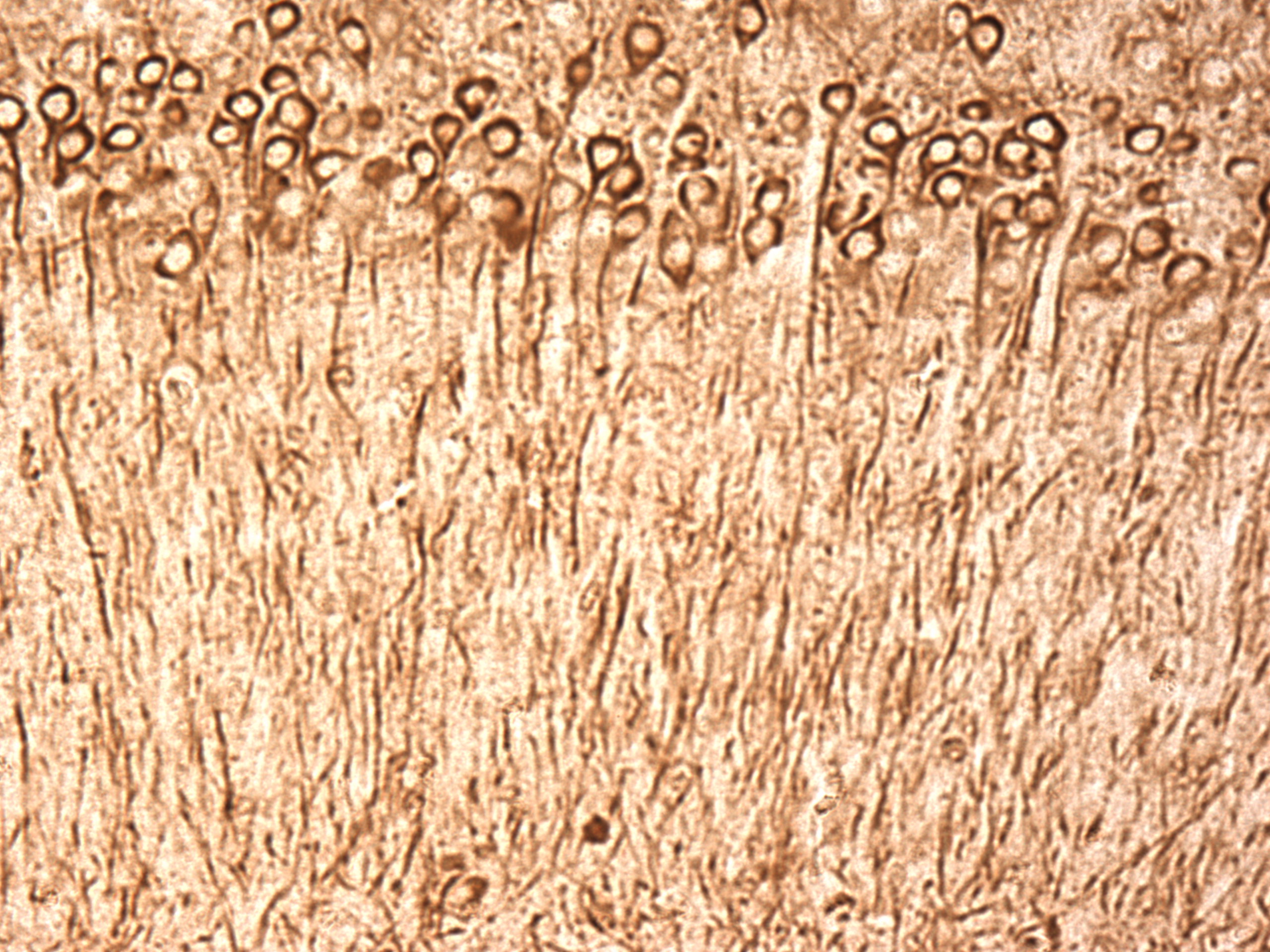

Supplement: Supplementary file 13 — Raw Western Blot and Microscopy Images [file 44318_2026_809_MOESM13_ESM.zip › SD_Images/SD FIG 3G/3xTg-AD x Camk2a-Cre HT7.TIF]

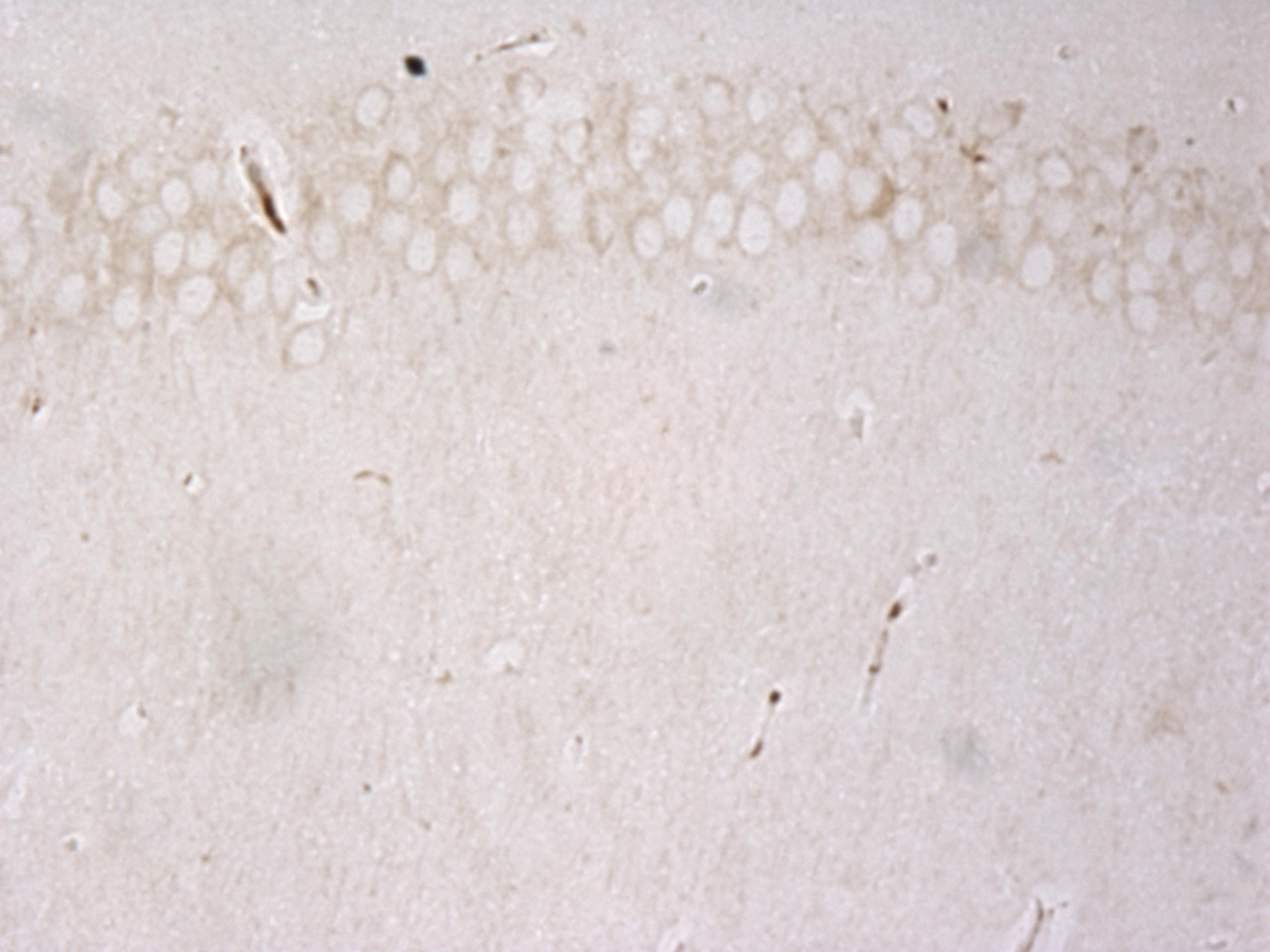

Supplement: Supplementary file 13 — Raw Western Blot and Microscopy Images [file 44318_2026_809_MOESM13_ESM.zip › SD_Images/SD FIG 3G/3xTg-AD x Mcuflfl x Camk2a-Cre PHF13.TIF]

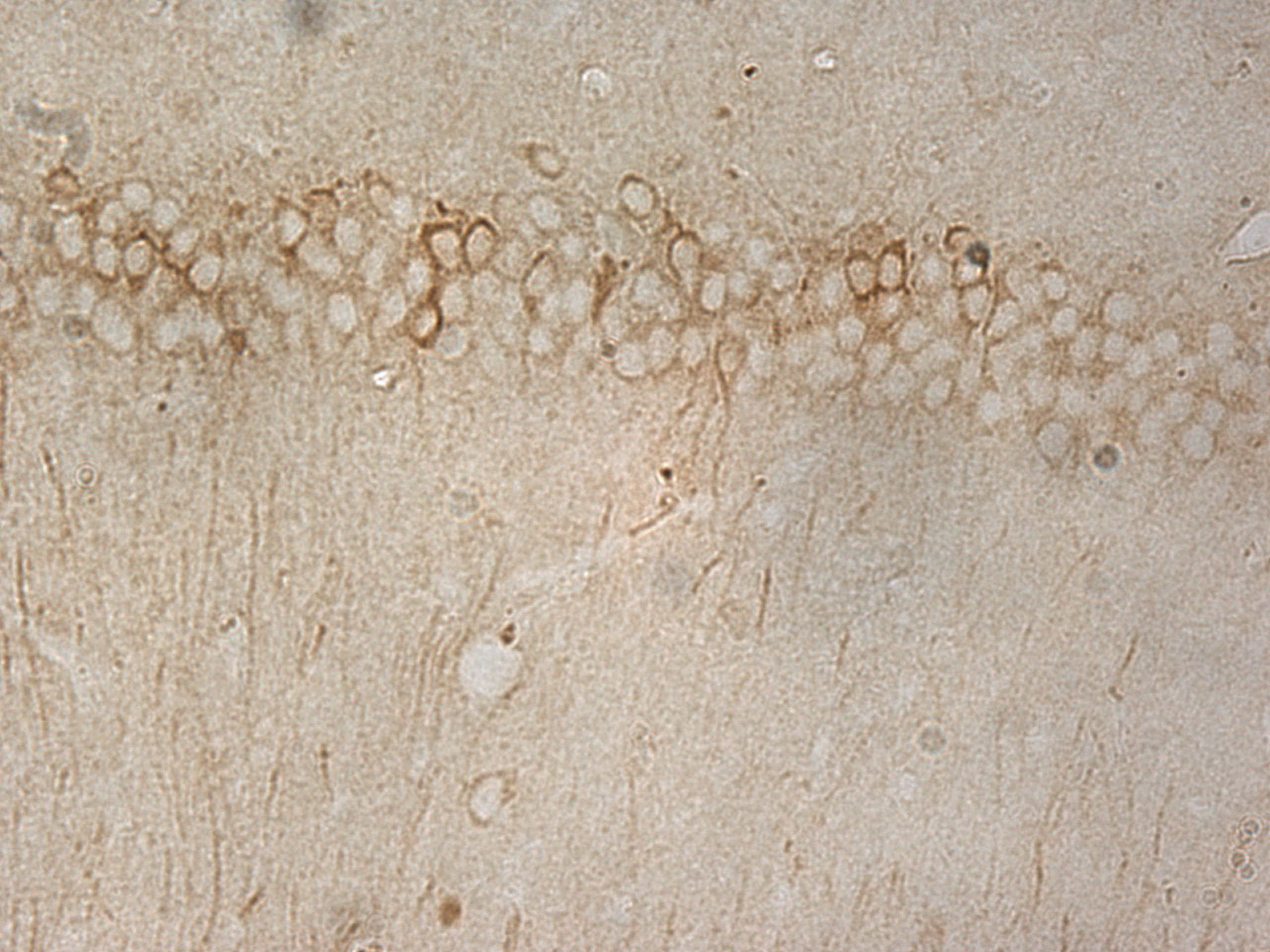

Supplement: Supplementary file 13 — Raw Western Blot and Microscopy Images [file 44318_2026_809_MOESM13_ESM.zip › SD_Images/SD FIG 3G/3xTg-AD x Mcuflfl x Camk2a-Cre- AT180.TIF]

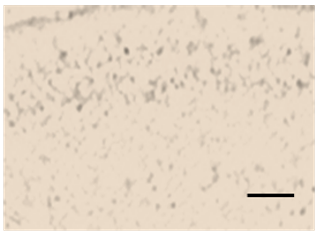

Supplement: Supplementary file 13 — Raw Western Blot and Microscopy Images [file 44318_2026_809_MOESM13_ESM.zip › SD_Images/SD FIG 3G/3xTg-AD x Mcuflfl x Camk2a-Cre- AT270.tif]

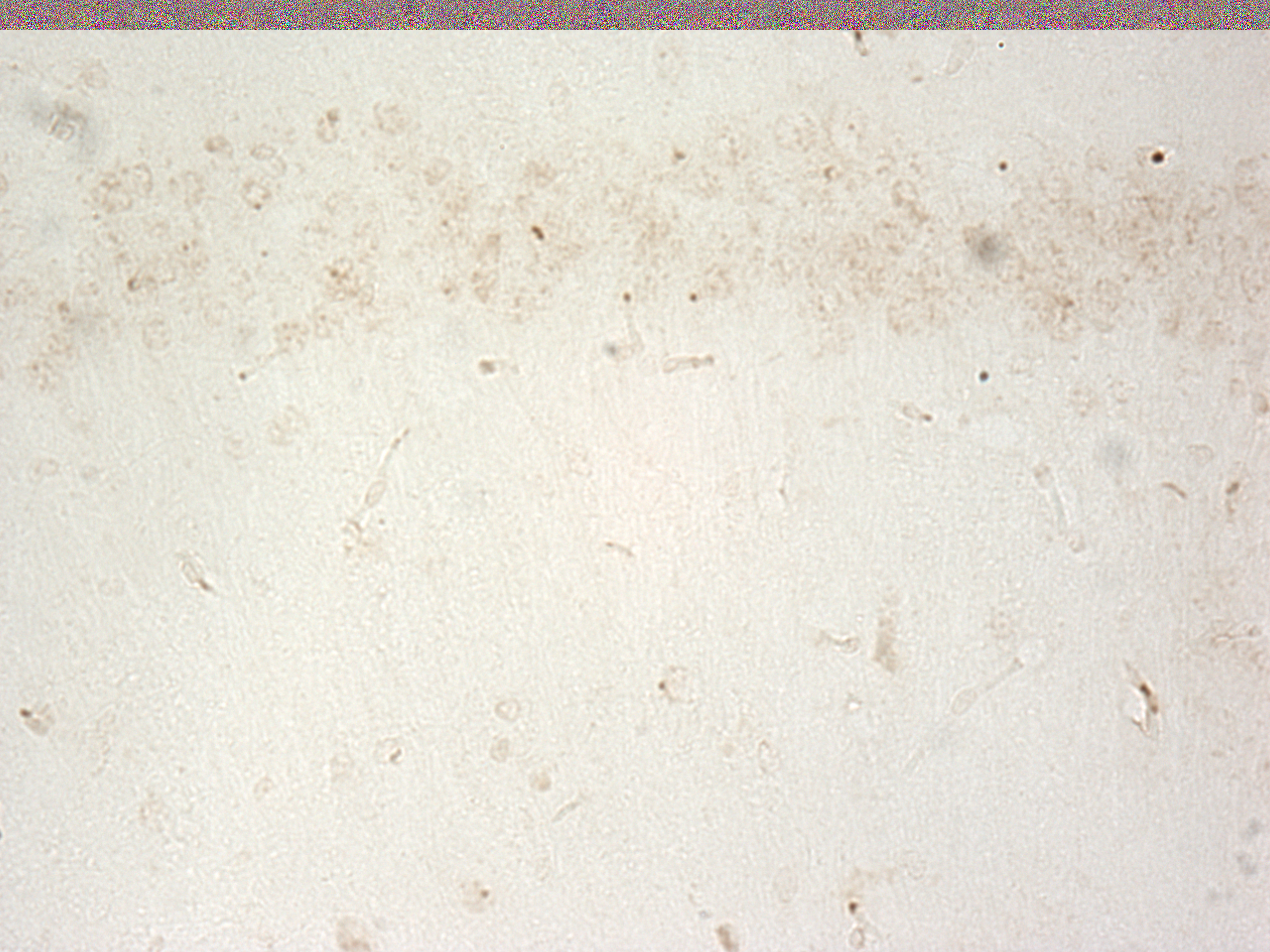

Supplement: Supplementary file 13 — Raw Western Blot and Microscopy Images [file 44318_2026_809_MOESM13_ESM.zip › SD_Images/SD FIG 3G/3xTg-AD x Mcuflfl x Camk2a-Cre- AT8.TIF]

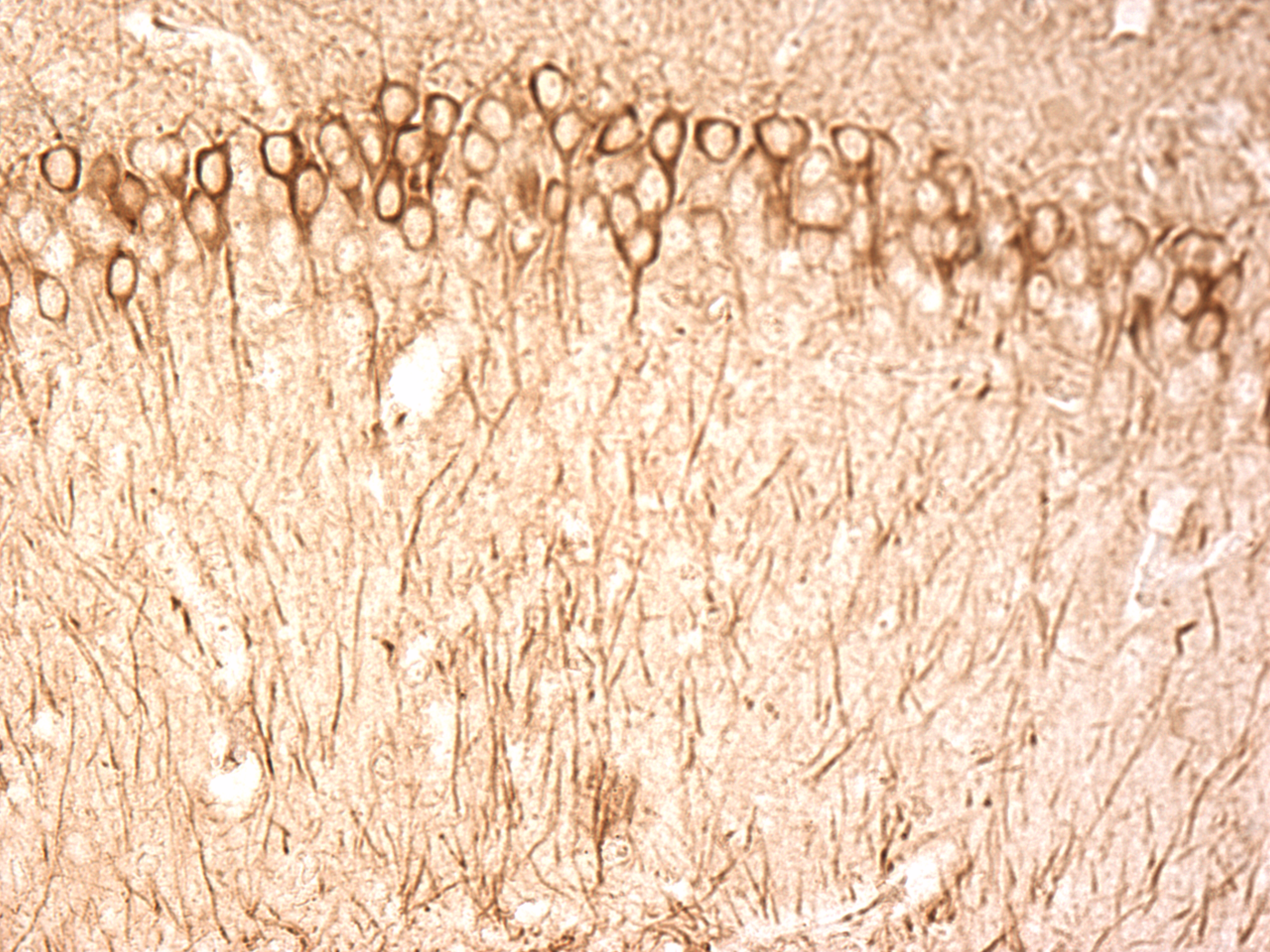

Supplement: Supplementary file 13 — Raw Western Blot and Microscopy Images [file 44318_2026_809_MOESM13_ESM.zip › SD_Images/SD FIG 3G/3xTg-AD x Mcuflfl x Camk2a-Cre- HT7.TIF]

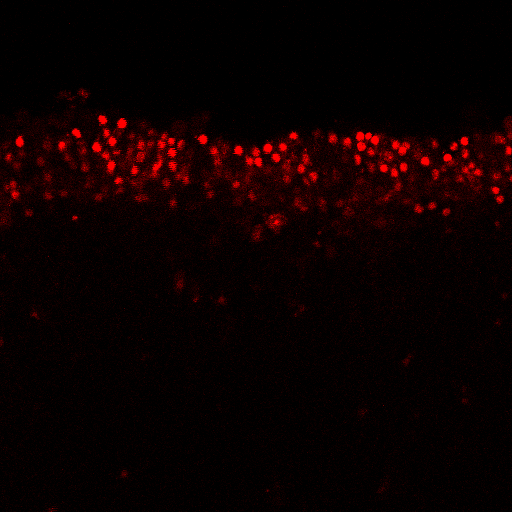

Supplement: Supplementary file 13 — Raw Western Blot and Microscopy Images [file 44318_2026_809_MOESM13_ESM.zip › SD_Images/SD Fig 4A/3xTg-AD x Camk2aCre-Hippo.tif]

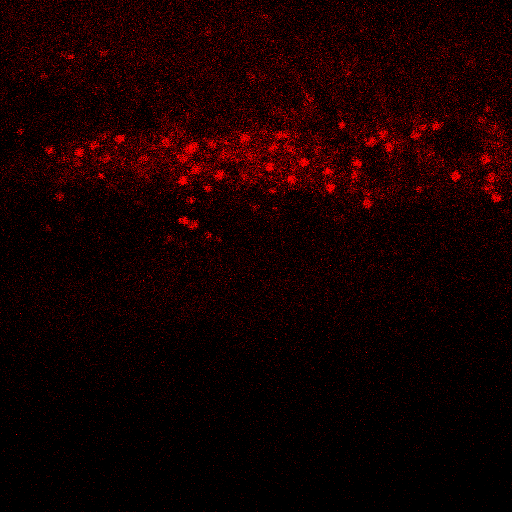

Supplement: Supplementary file 13 — Raw Western Blot and Microscopy Images [file 44318_2026_809_MOESM13_ESM.zip › SD_Images/SD Fig 4A/3xtg-AD x Mcu flfl x Cre- Hippo.tif]

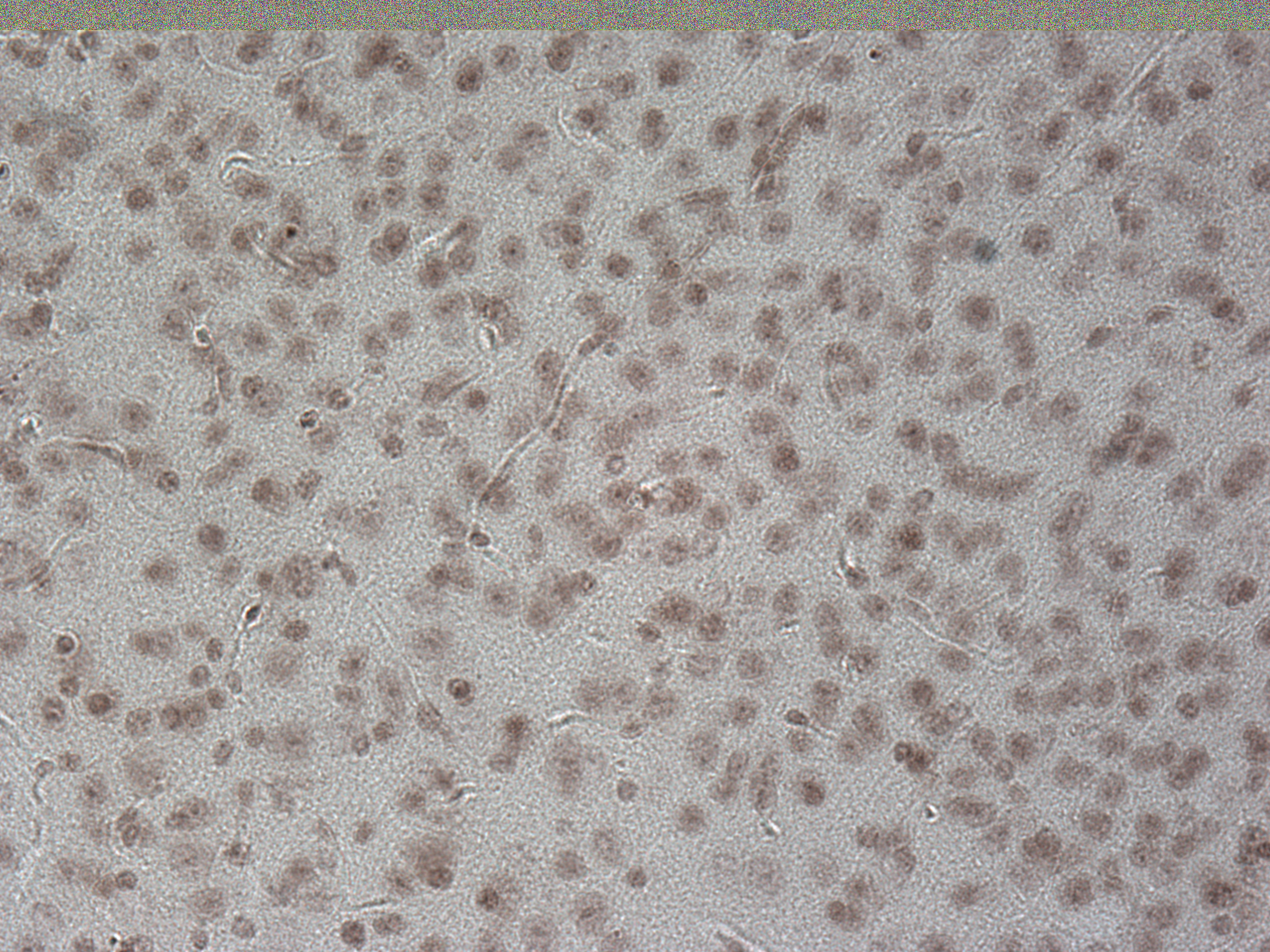

Supplement: Supplementary file 13 — Raw Western Blot and Microscopy Images [file 44318_2026_809_MOESM13_ESM.zip › SD_Images/SD Fig 4D/3xTg-AD x Camk2a-Cre-Cortex.TIF]

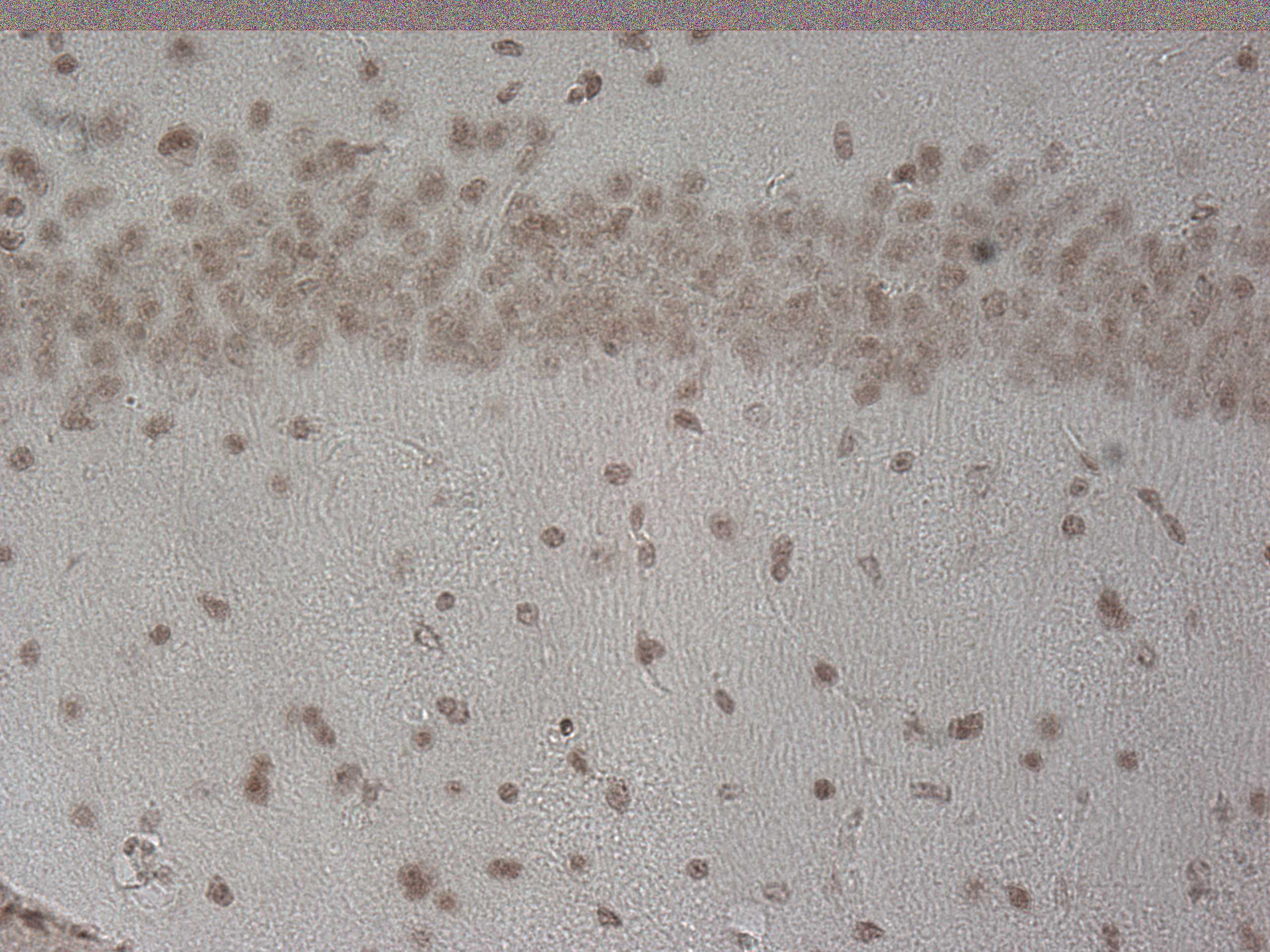

Supplement: Supplementary file 13 — Raw Western Blot and Microscopy Images [file 44318_2026_809_MOESM13_ESM.zip › SD_Images/SD Fig 4D/3xTg-AD x Camk2a-Cre-Hippo.TIF]

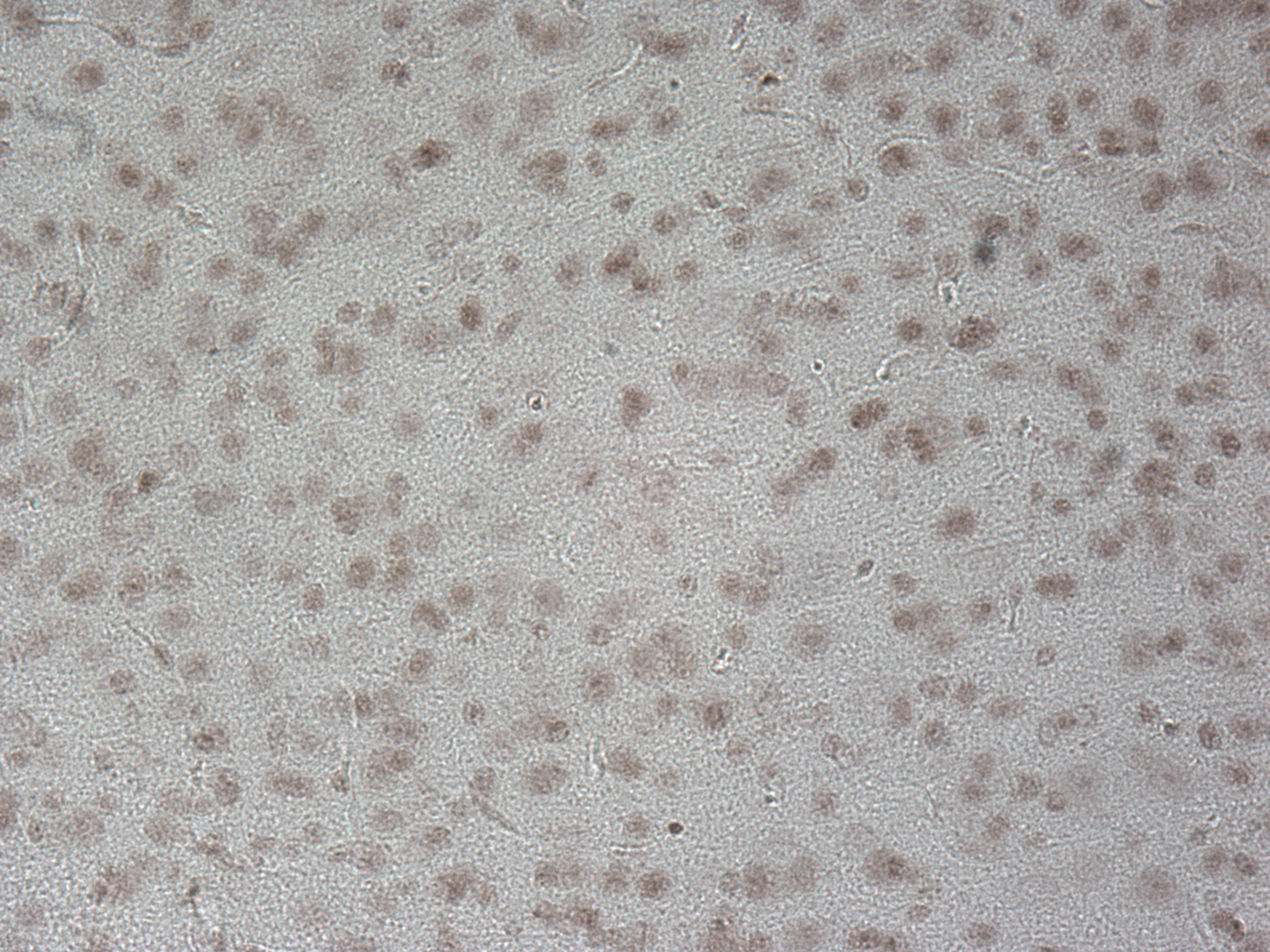

Supplement: Supplementary file 13 — Raw Western Blot and Microscopy Images [file 44318_2026_809_MOESM13_ESM.zip › SD_Images/SD Fig 4D/3xTg-AD x Mcuflfl x Camk2a-Cre-Cortex.TIF]

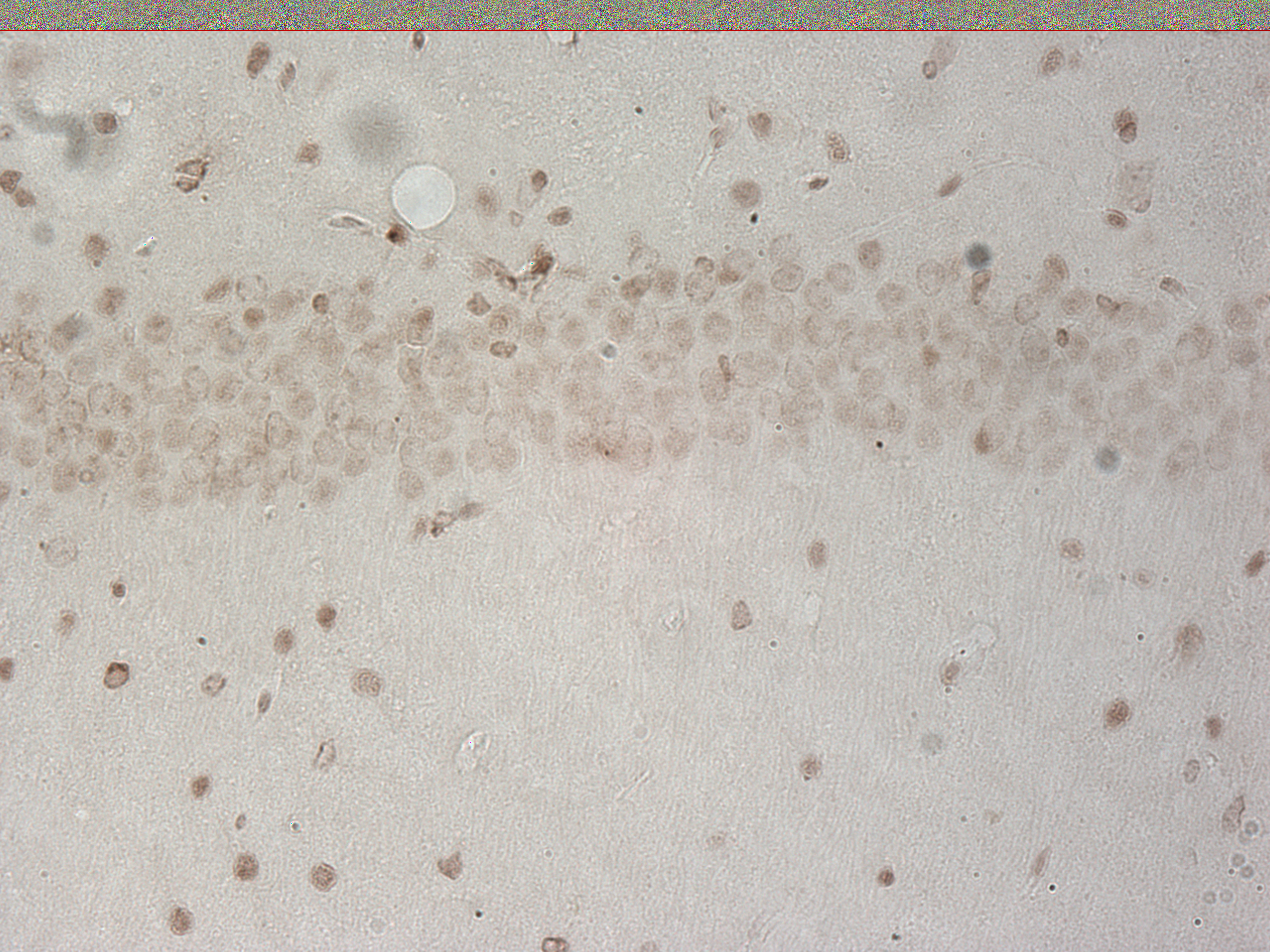

Supplement: Supplementary file 13 — Raw Western Blot and Microscopy Images [file 44318_2026_809_MOESM13_ESM.zip › SD_Images/SD Fig 4D/3xTg-AD x Mcuflfl x Camk2a-Cre-Hippo.TIF]

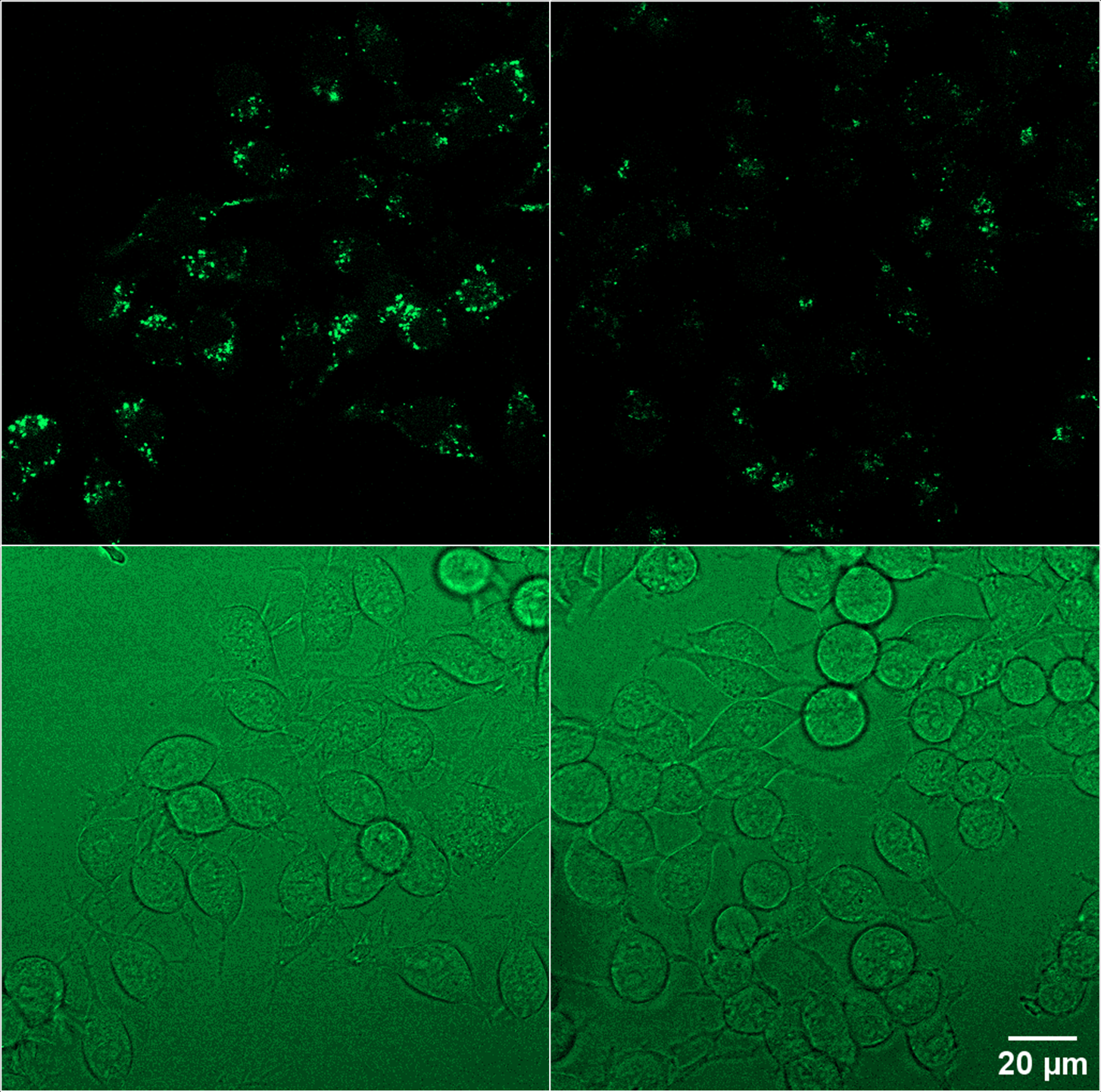

Supplement: Supplementary file 13 — Raw Western Blot and Microscopy Images [file 44318_2026_809_MOESM13_ESM.zip › SD_Images/SD Fig 5J/5J.tif]

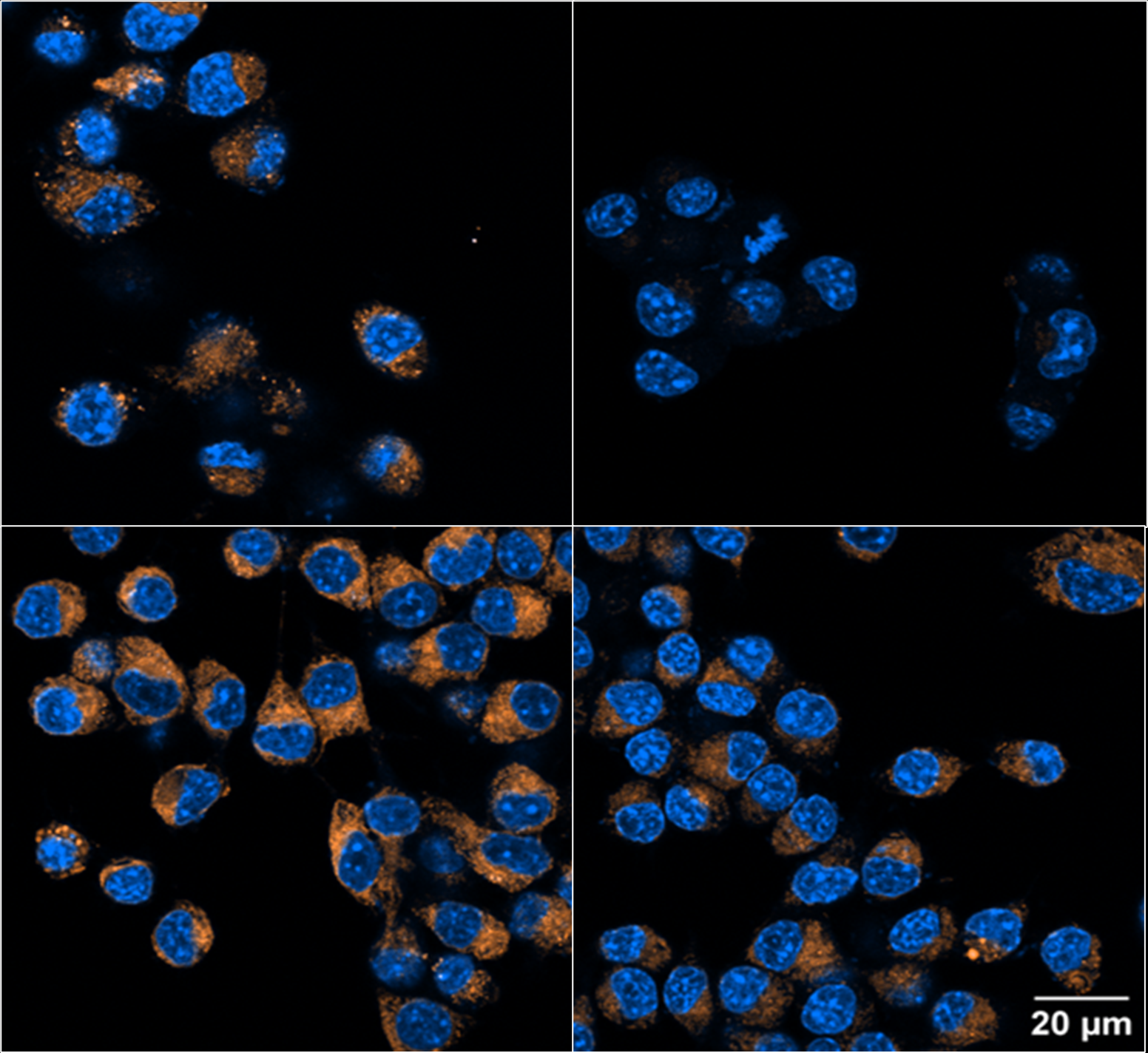

Supplement: Supplementary file 13 — Raw Western Blot and Microscopy Images [file 44318_2026_809_MOESM13_ESM.zip › SD_Images/SD Fig 5L/5L.tif]

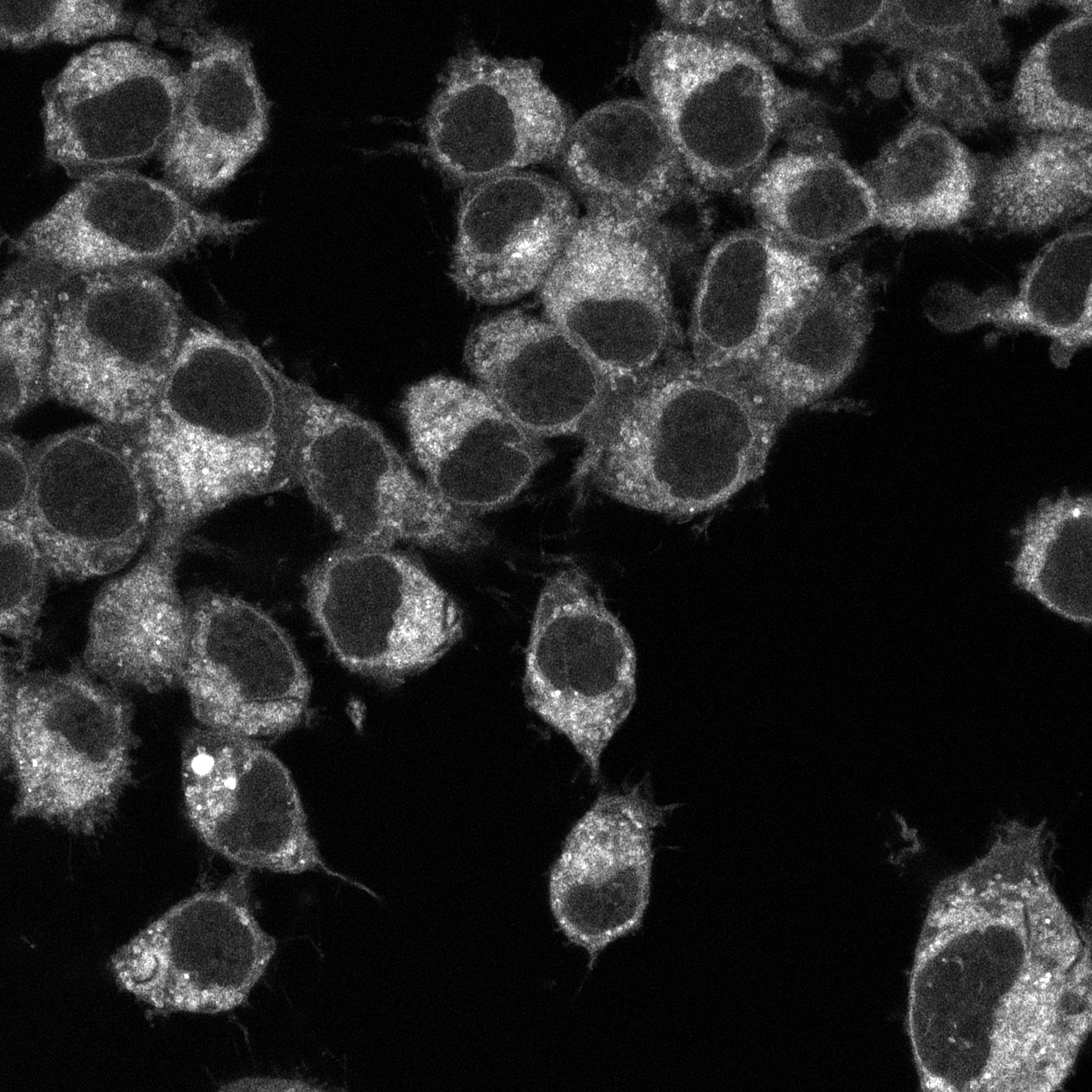

Supplement: Supplementary file 13 — Raw Western Blot and Microscopy Images [file 44318_2026_809_MOESM13_ESM.zip › SD_Images/SD Fig 5L/APPsw MCUshRNA.tif]

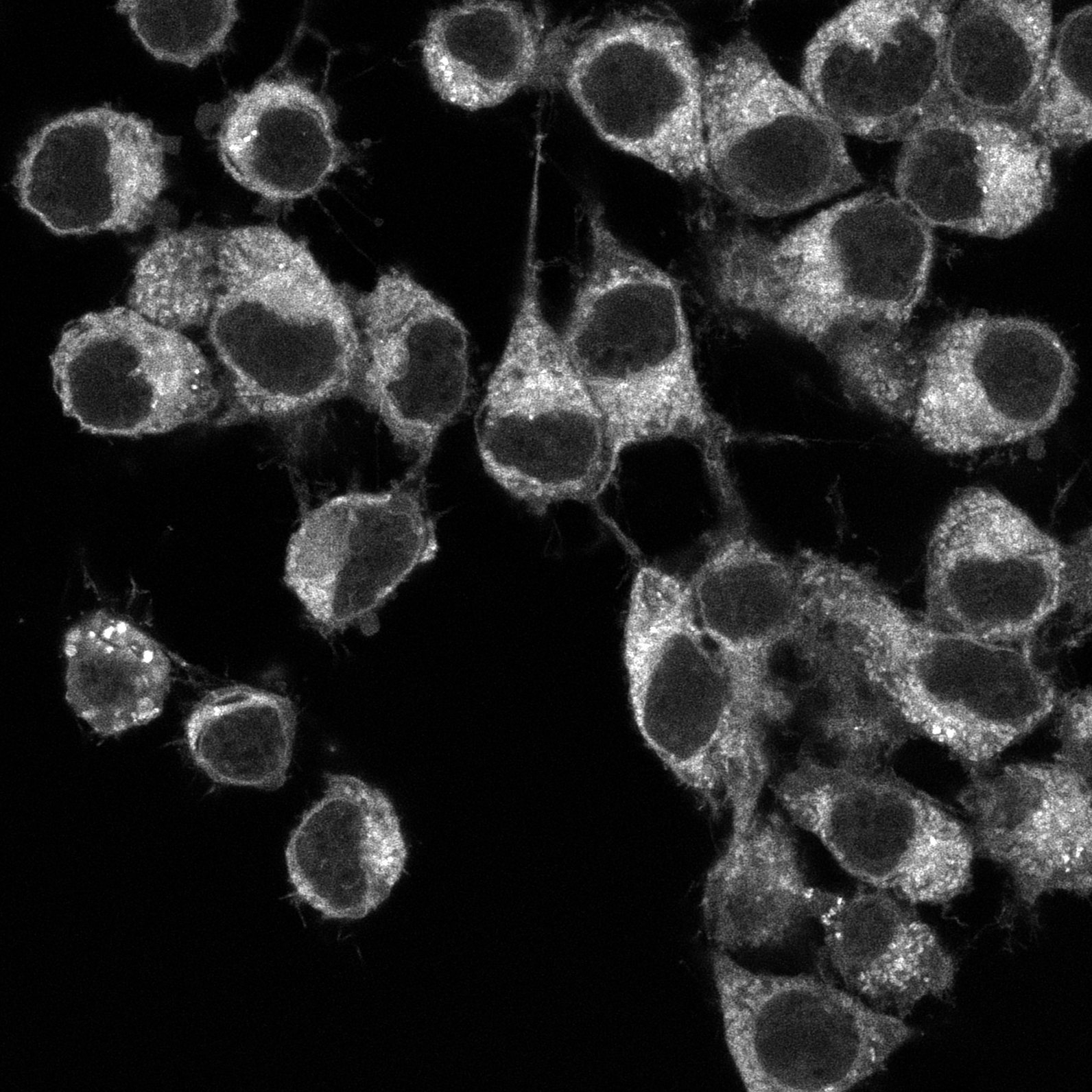

Supplement: Supplementary file 13 — Raw Western Blot and Microscopy Images [file 44318_2026_809_MOESM13_ESM.zip › SD_Images/SD Fig 5L/APPsw scr shRNA.tif]

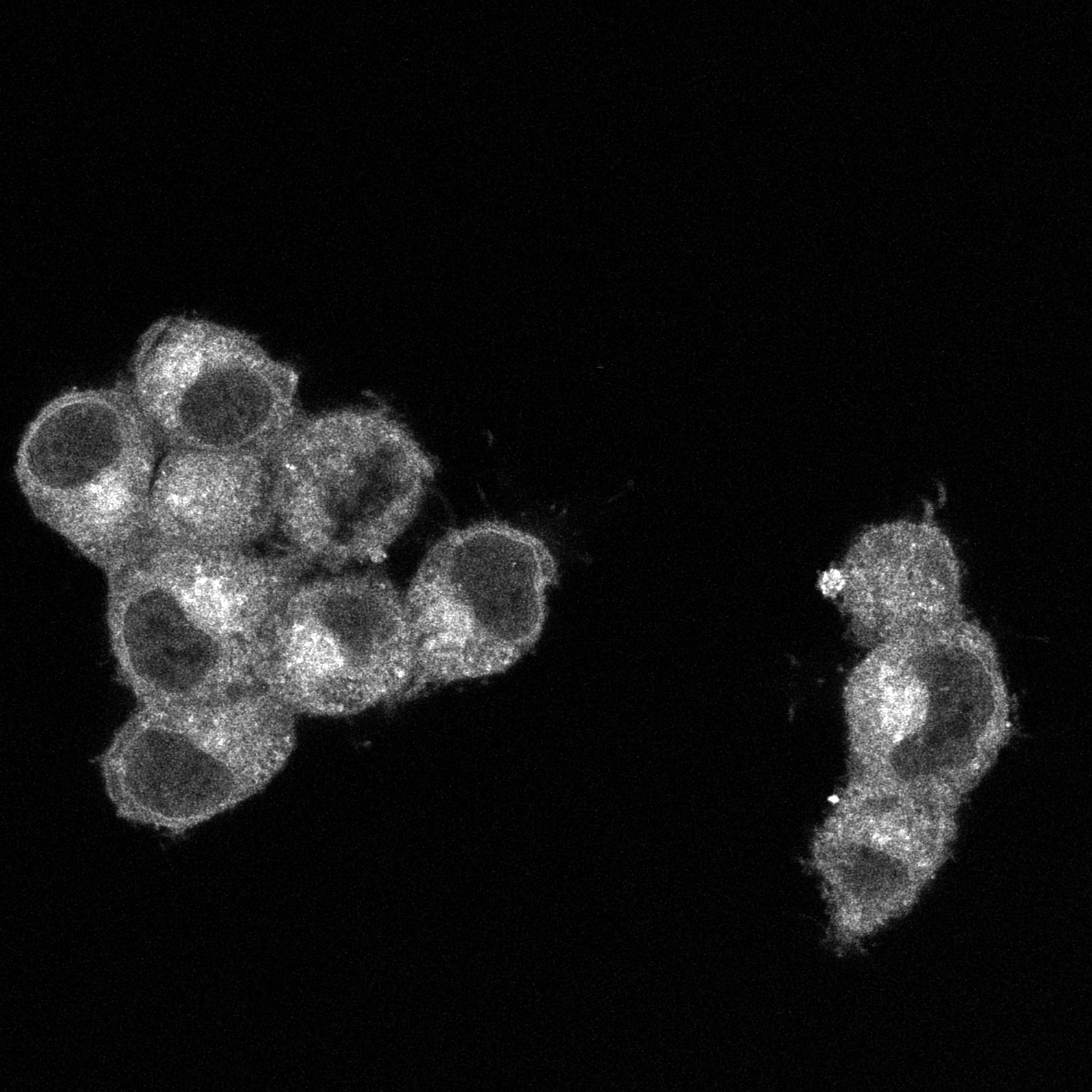

Supplement: Supplementary file 13 — Raw Western Blot and Microscopy Images [file 44318_2026_809_MOESM13_ESM.zip › SD_Images/SD Fig 5L/MCUshRNA.tif]

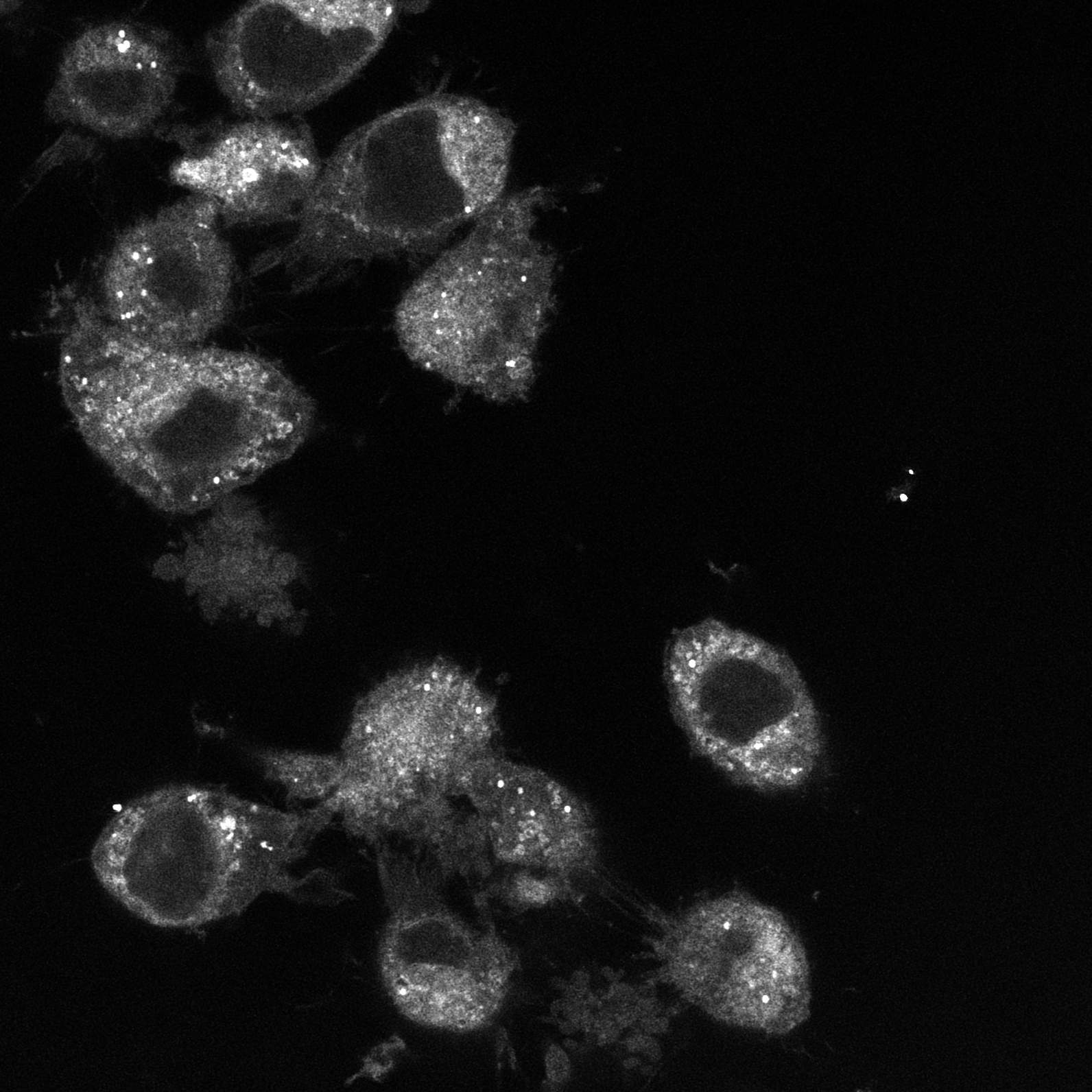

Supplement: Supplementary file 13 — Raw Western Blot and Microscopy Images [file 44318_2026_809_MOESM13_ESM.zip › SD_Images/SD Fig 5L/scrshRNA.tif]

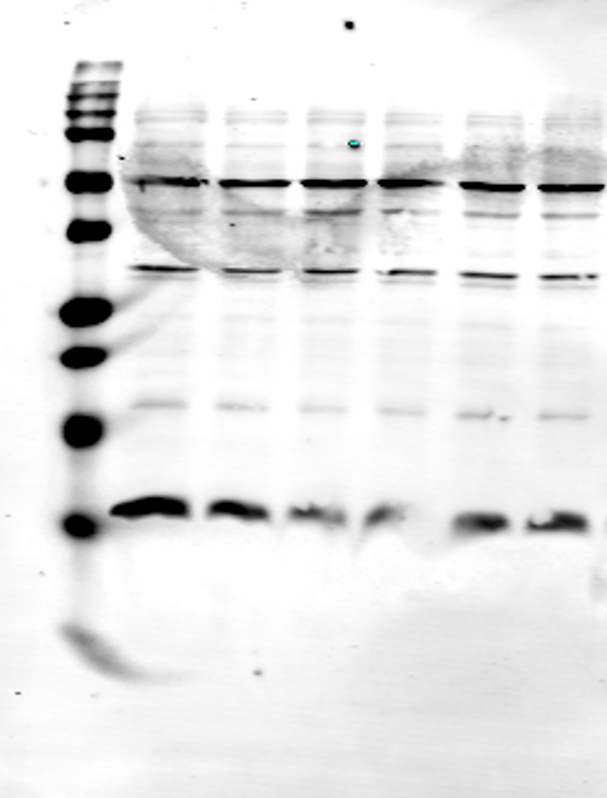

Supplement: Supplementary file 13 — Raw Western Blot and Microscopy Images [file 44318_2026_809_MOESM13_ESM.zip › SD_Blots/SD Figure 1B/1B MICU3.tif]

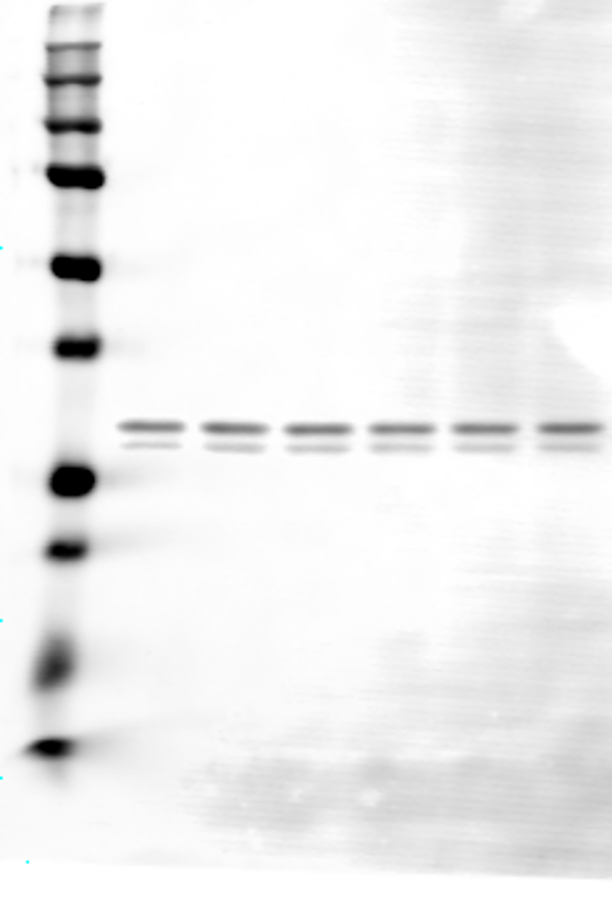

Supplement: Supplementary file 13 — Raw Western Blot and Microscopy Images [file 44318_2026_809_MOESM13_ESM.zip › SD_Blots/SD Figure 1B/1B VDAC.tif]

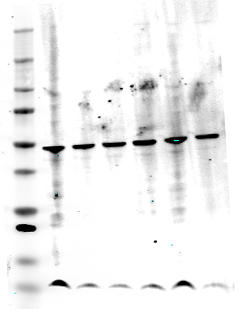

Supplement: Supplementary file 13 — Raw Western Blot and Microscopy Images [file 44318_2026_809_MOESM13_ESM.zip › SD_Blots/SD Figure 1B/1B-EMRE.tif]

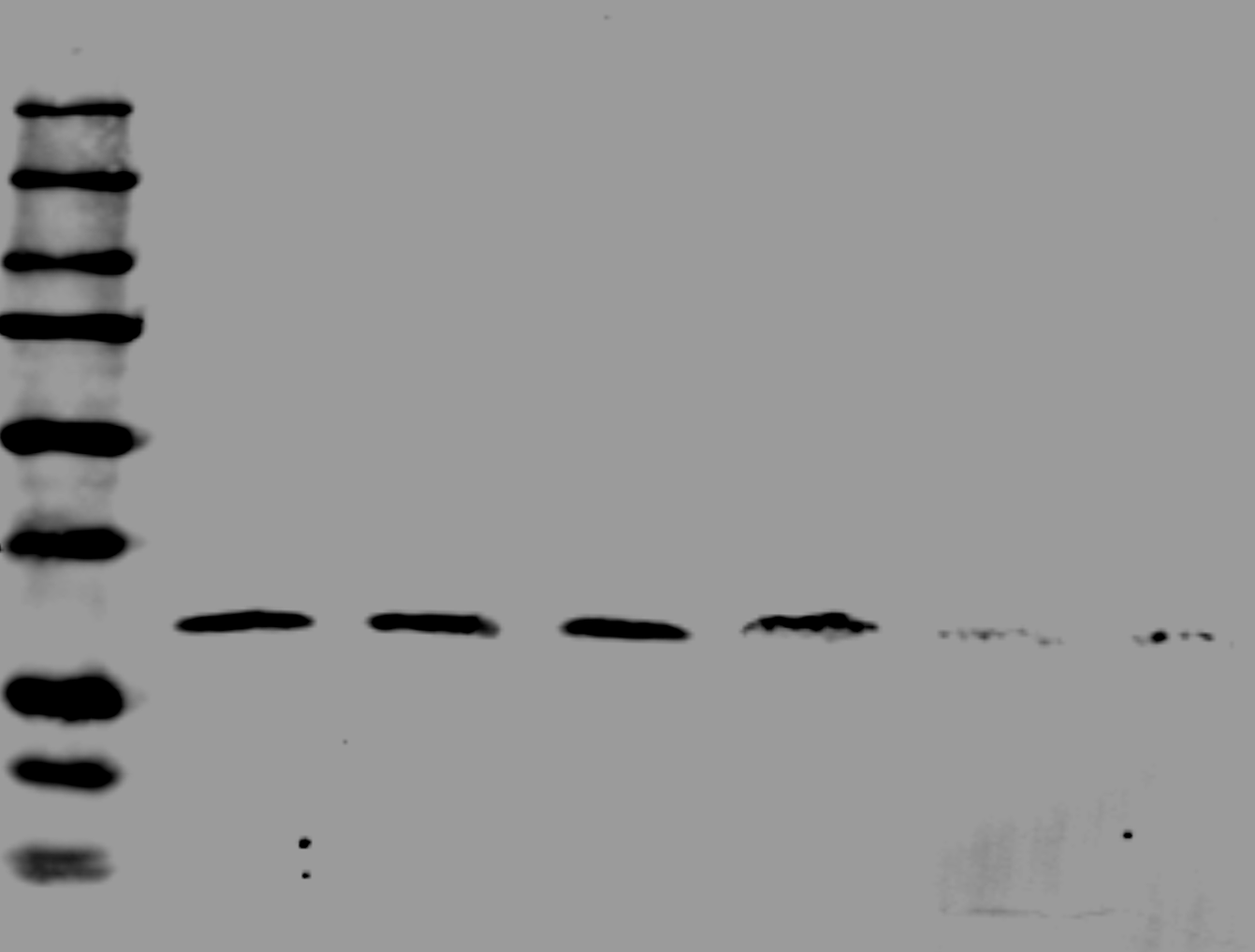

Supplement: Supplementary file 13 — Raw Western Blot and Microscopy Images [file 44318_2026_809_MOESM13_ESM.zip › SD_Blots/SD Figure 1B/1B-MCU.tif]

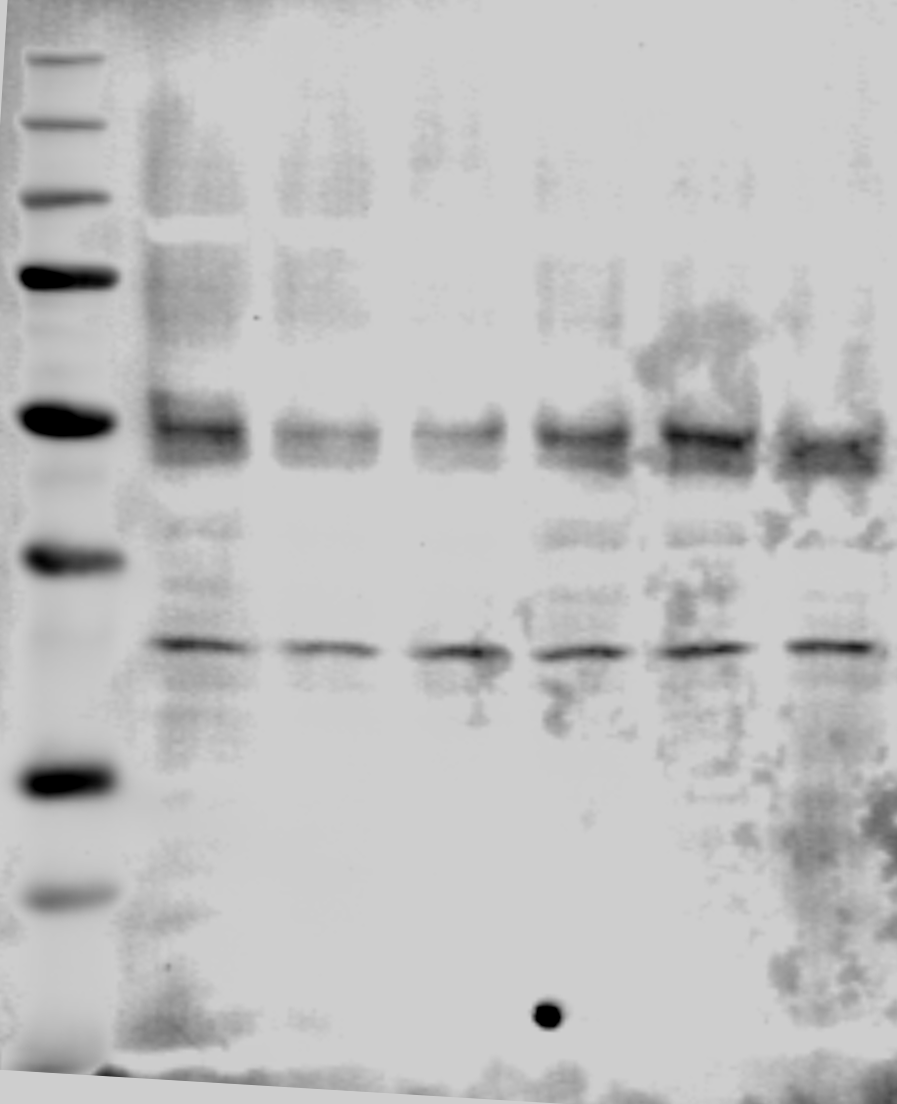

Supplement: Supplementary file 13 — Raw Western Blot and Microscopy Images [file 44318_2026_809_MOESM13_ESM.zip › SD_Blots/SD Figure 1B/1B-MCUB.tif]

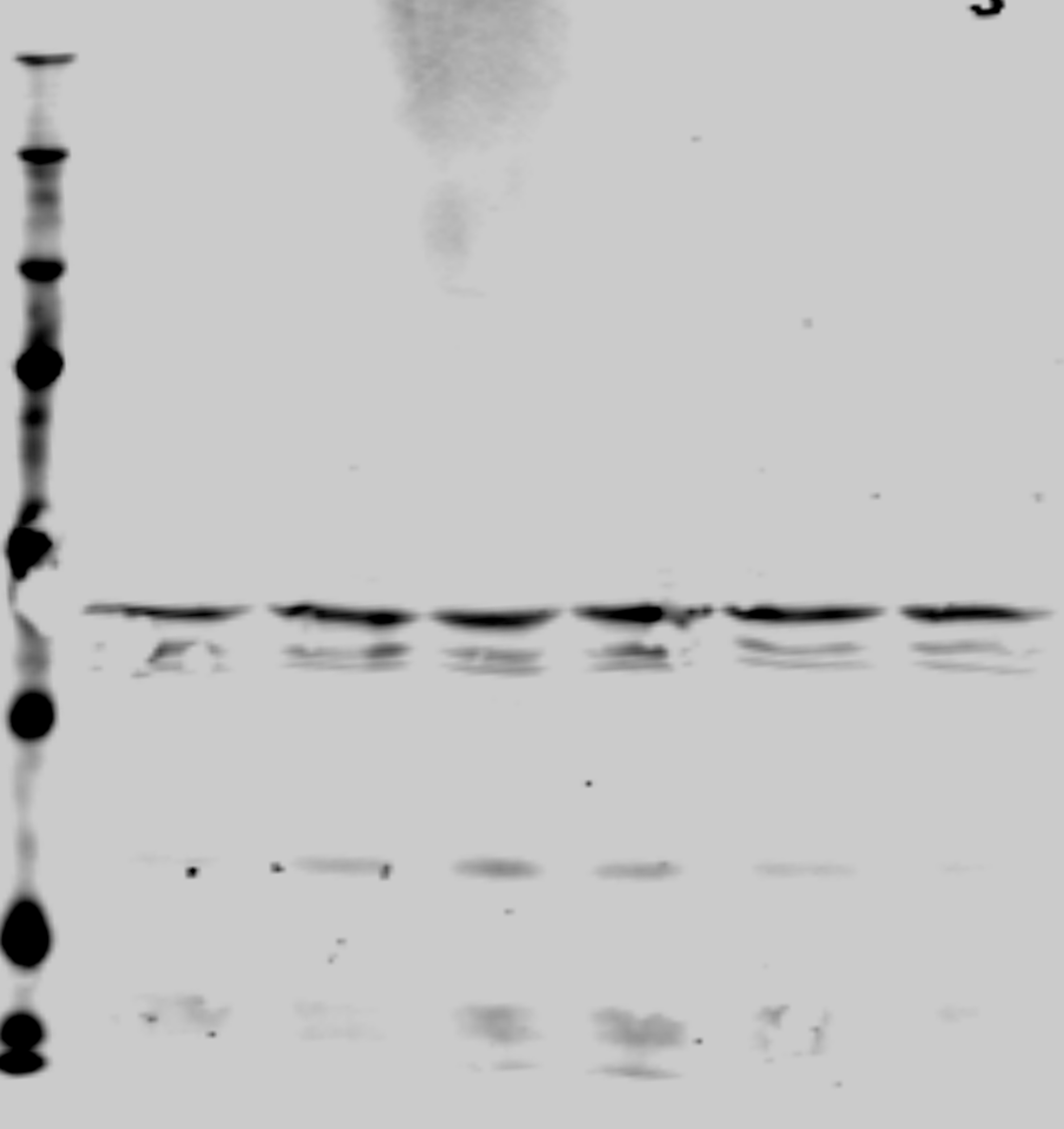

Supplement: Supplementary file 13 — Raw Western Blot and Microscopy Images [file 44318_2026_809_MOESM13_ESM.zip › SD_Blots/SD Figure 1B/1B-MICU1.tif]

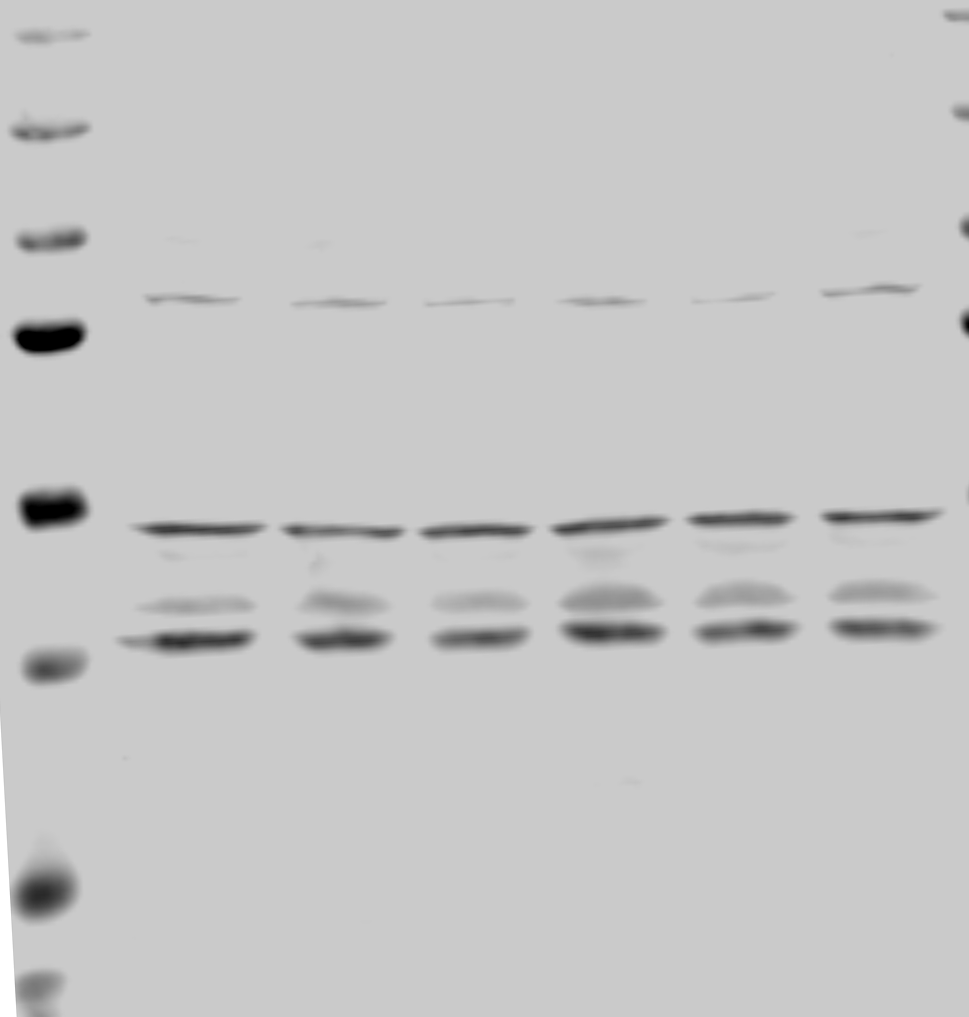

Supplement: Supplementary file 13 — Raw Western Blot and Microscopy Images [file 44318_2026_809_MOESM13_ESM.zip › SD_Blots/SD Figure 1B/1B-MICU2.tif]

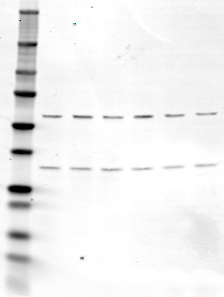

Supplement: Supplementary file 13 — Raw Western Blot and Microscopy Images [file 44318_2026_809_MOESM13_ESM.zip › SD_Blots/SD Figure 1B/1B-NCLX .tif.tif]

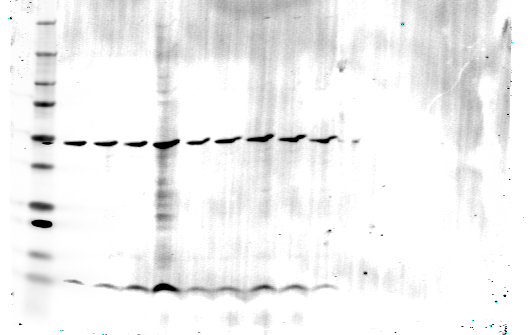

Supplement: Supplementary file 13 — Raw Western Blot and Microscopy Images [file 44318_2026_809_MOESM13_ESM.zip › SD_Blots/SD Figure 1C/1C-EMRE.tif]

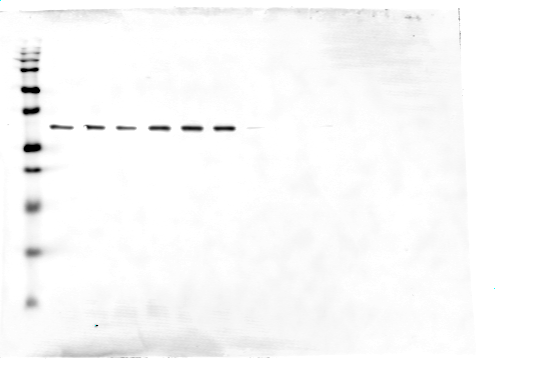

Supplement: Supplementary file 13 — Raw Western Blot and Microscopy Images [file 44318_2026_809_MOESM13_ESM.zip › SD_Blots/SD Figure 1C/1C-MCU.tif.tif]

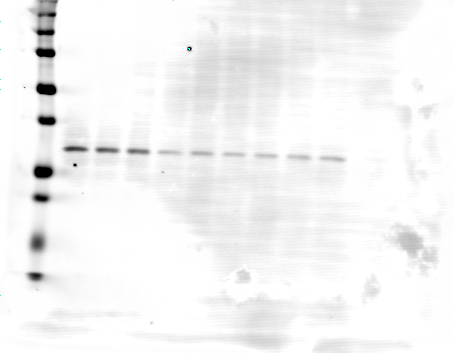

Supplement: Supplementary file 13 — Raw Western Blot and Microscopy Images [file 44318_2026_809_MOESM13_ESM.zip › SD_Blots/SD Figure 1C/1C-MCUB.tif]

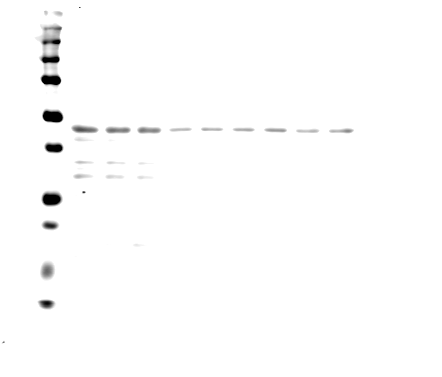

Supplement: Supplementary file 13 — Raw Western Blot and Microscopy Images [file 44318_2026_809_MOESM13_ESM.zip › SD_Blots/SD Figure 1C/1C-MICU1.tif]

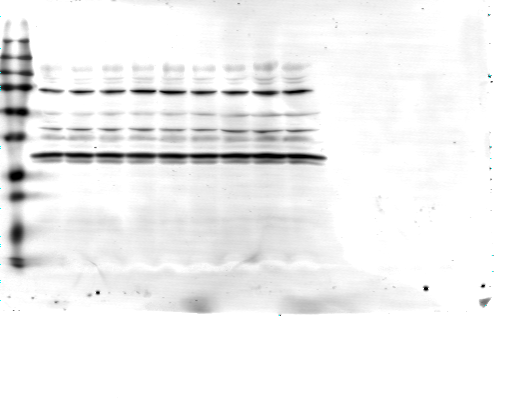

Supplement: Supplementary file 13 — Raw Western Blot and Microscopy Images [file 44318_2026_809_MOESM13_ESM.zip › SD_Blots/SD Figure 1C/1C-MICU2.tif.tif]

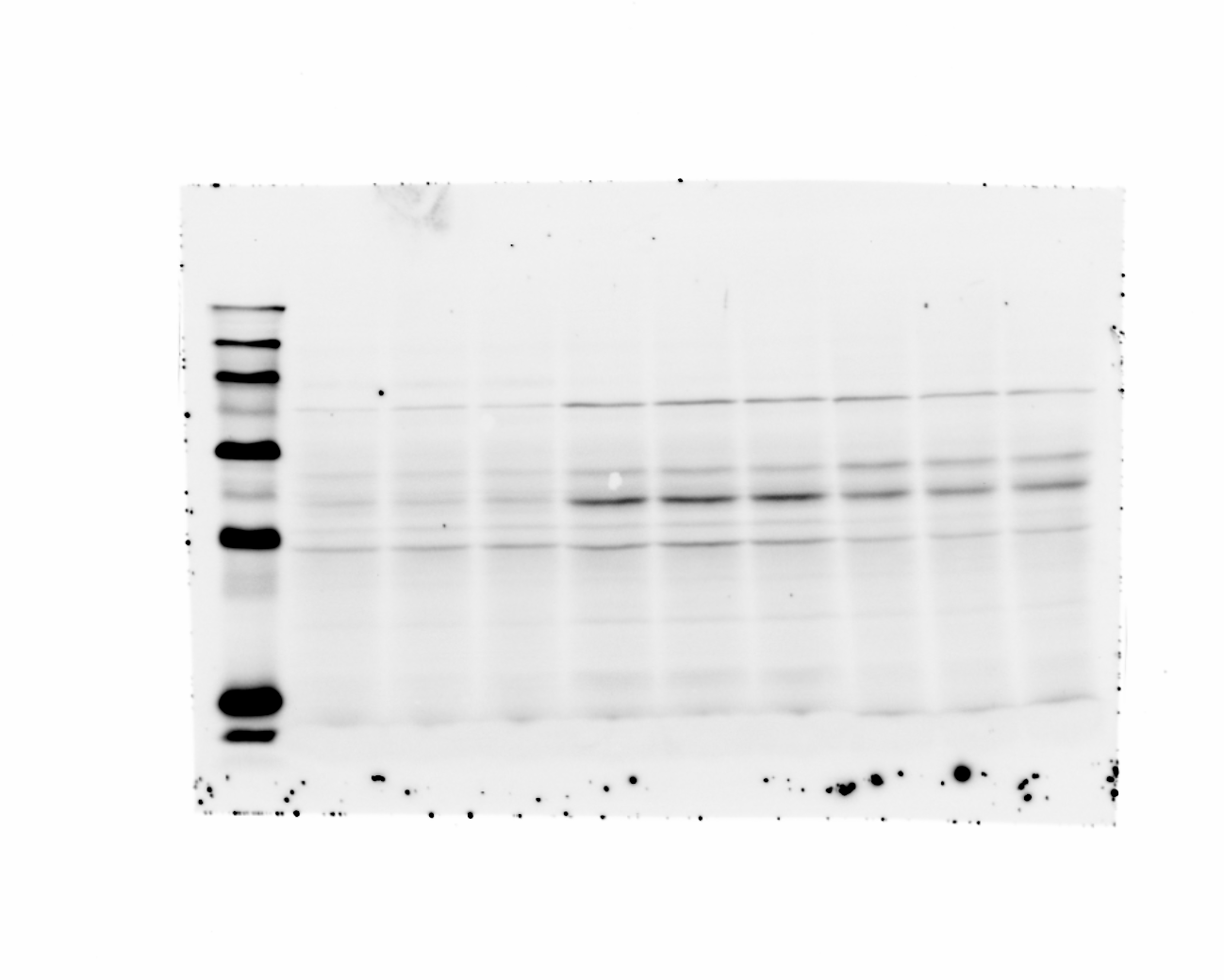

Supplement: Supplementary file 13 — Raw Western Blot and Microscopy Images [file 44318_2026_809_MOESM13_ESM.zip › SD_Blots/SD Figure 1C/1C-MICU3.tif]

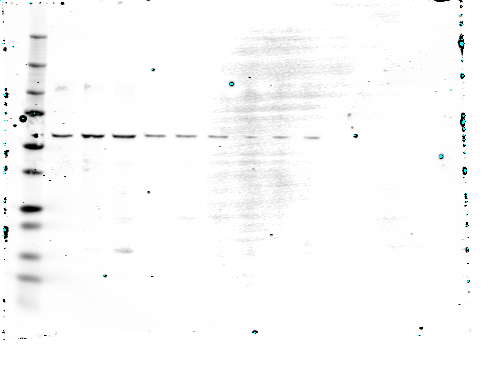

Supplement: Supplementary file 13 — Raw Western Blot and Microscopy Images [file 44318_2026_809_MOESM13_ESM.zip › SD_Blots/SD Figure 1C/1C-NCLX-.tif.tif]

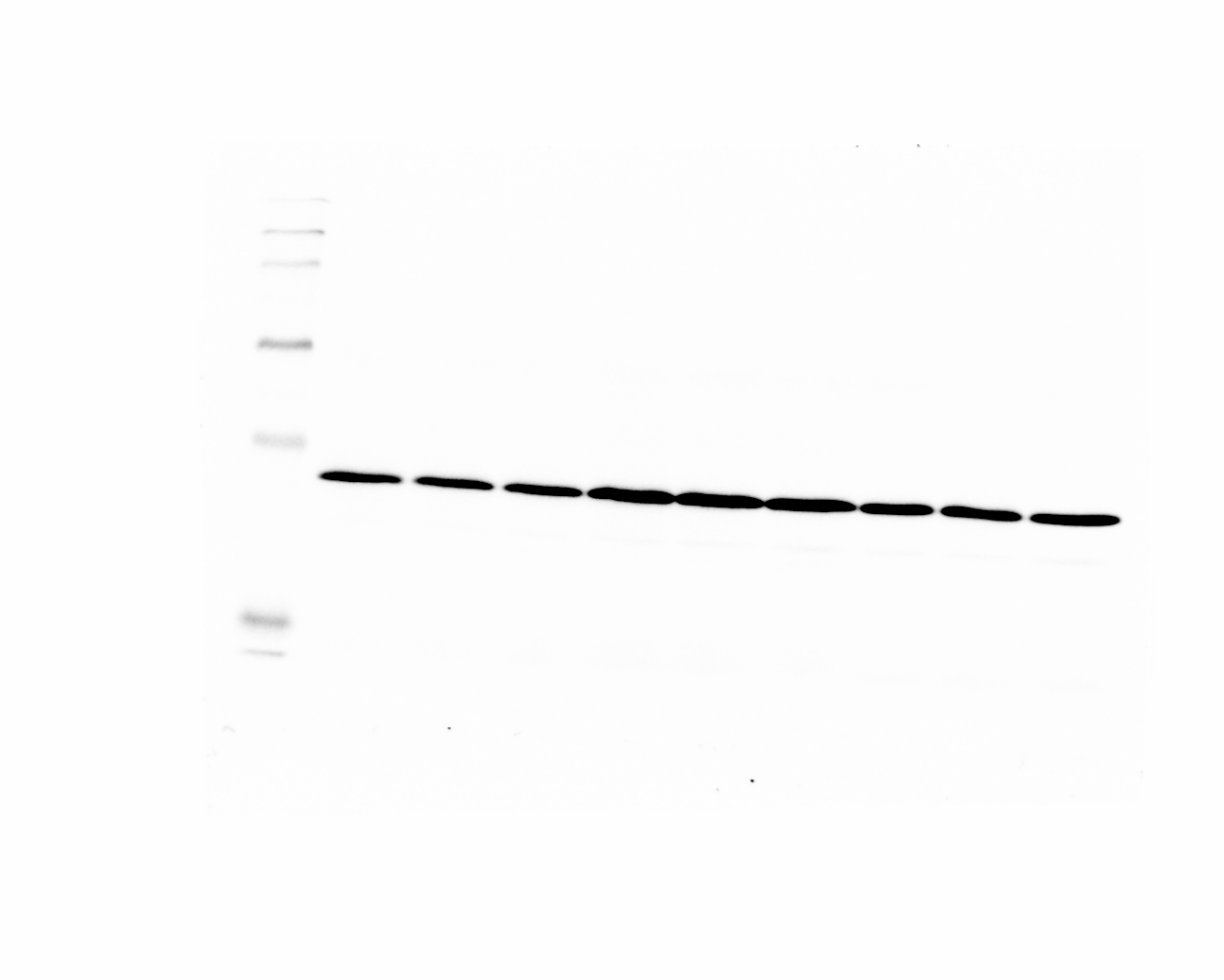

Supplement: Supplementary file 13 — Raw Western Blot and Microscopy Images [file 44318_2026_809_MOESM13_ESM.zip › SD_Blots/SD Figure 1C/1C-VDAC.tif]

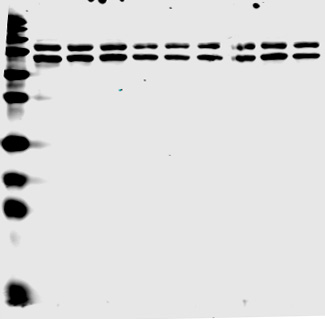

Supplement: Supplementary file 13 — Raw Western Blot and Microscopy Images [file 44318_2026_809_MOESM13_ESM.zip › SD_Blots/SD Figure 2G/2G ADAM-10.tif.jpg]

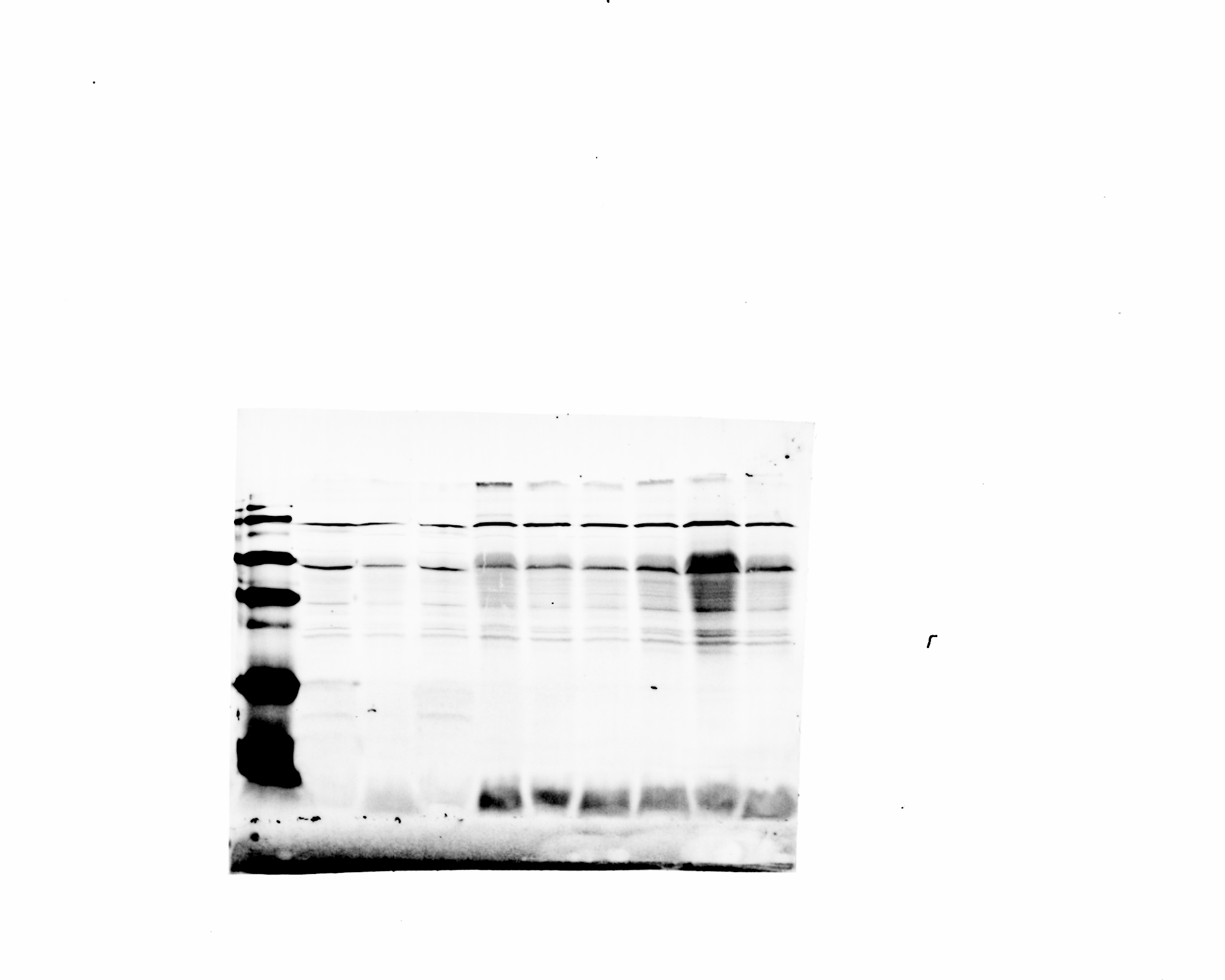

Supplement: Supplementary file 13 — Raw Western Blot and Microscopy Images [file 44318_2026_809_MOESM13_ESM.zip › SD_Blots/SD Figure 2G/2G AICD.tif]

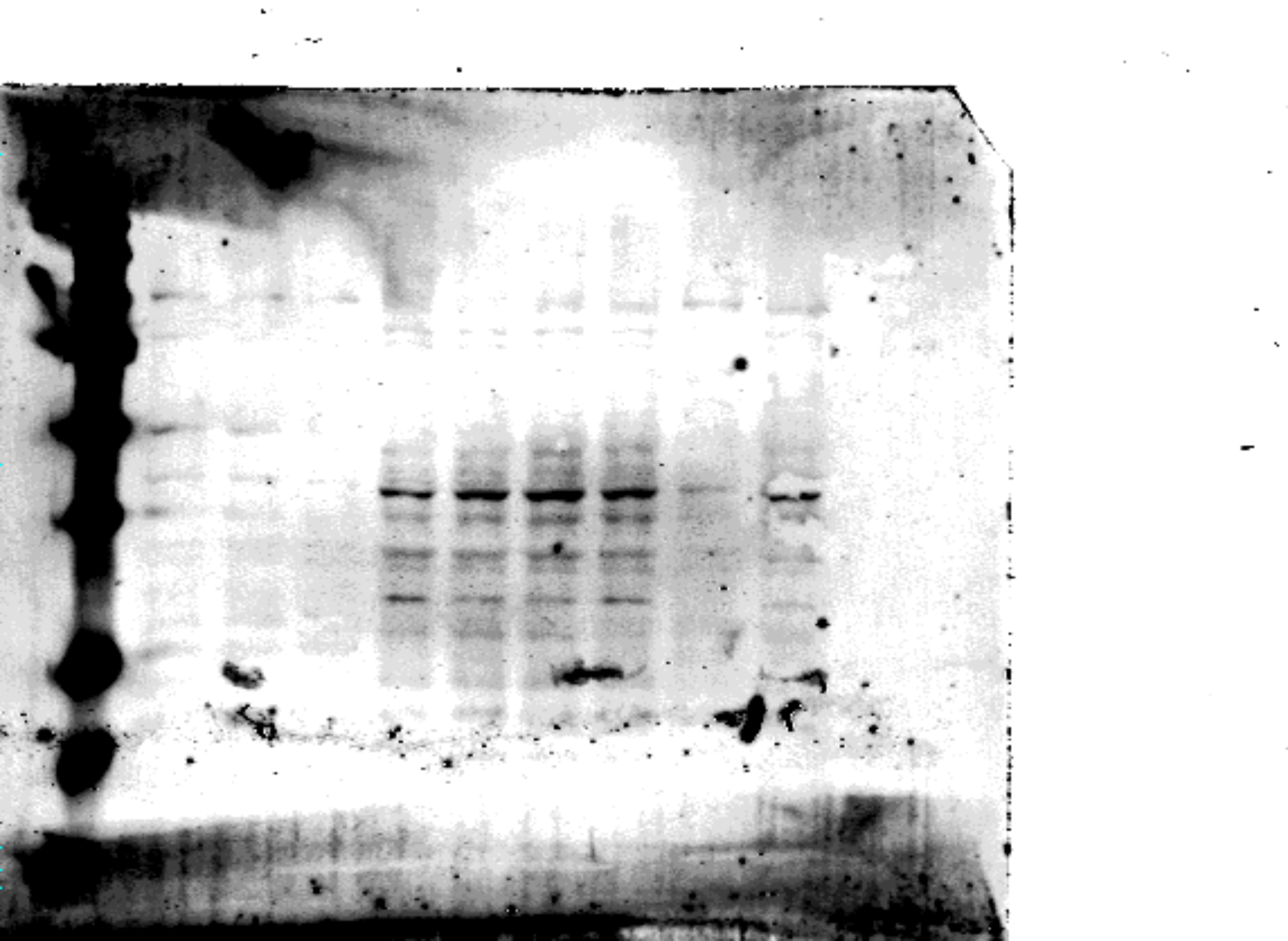

Supplement: Supplementary file 13 — Raw Western Blot and Microscopy Images [file 44318_2026_809_MOESM13_ESM.zip › SD_Blots/SD Figure 2G/2G APH.tif]

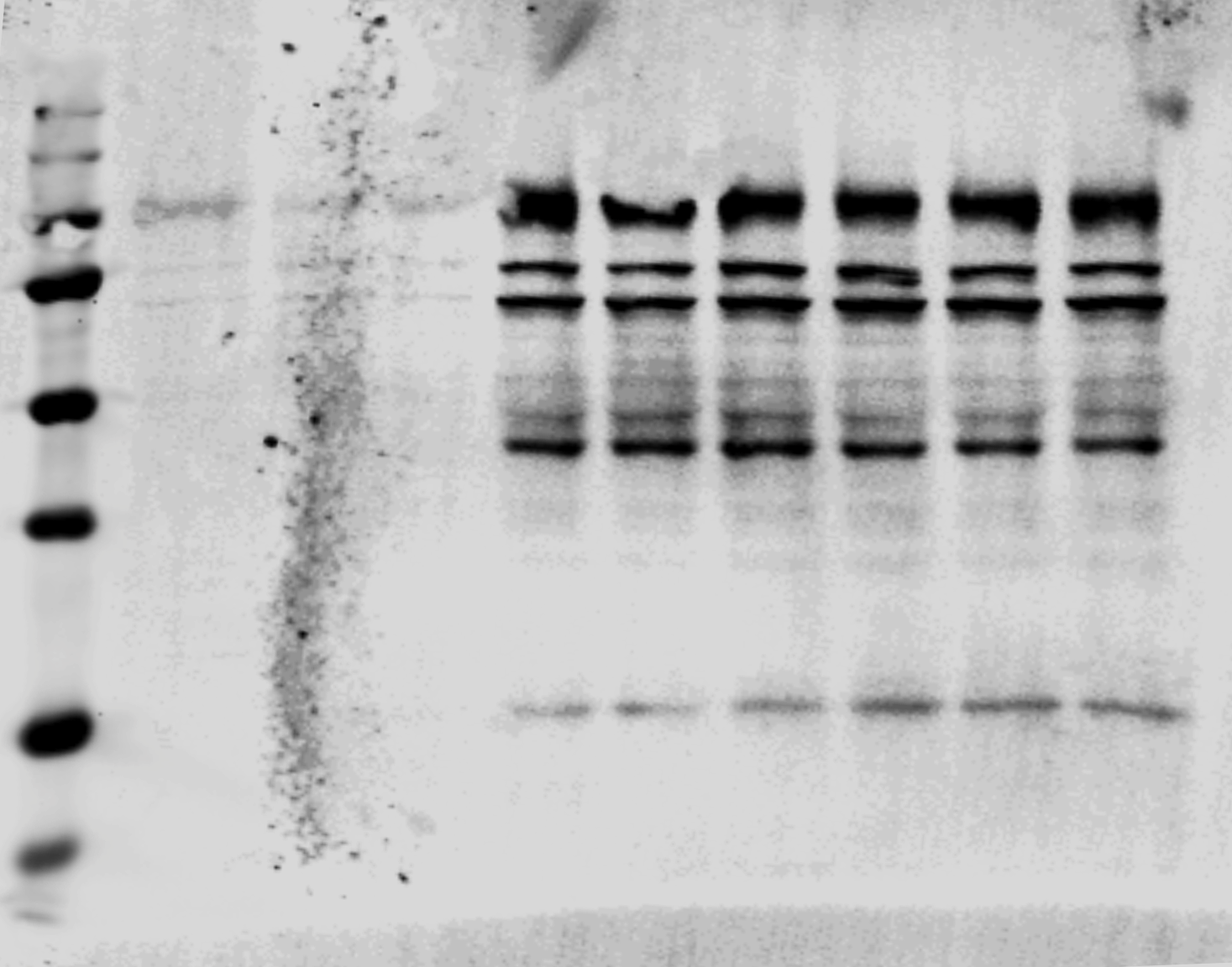

Supplement: Supplementary file 13 — Raw Western Blot and Microscopy Images [file 44318_2026_809_MOESM13_ESM.zip › SD_Blots/SD Figure 2G/2G APP.tif]

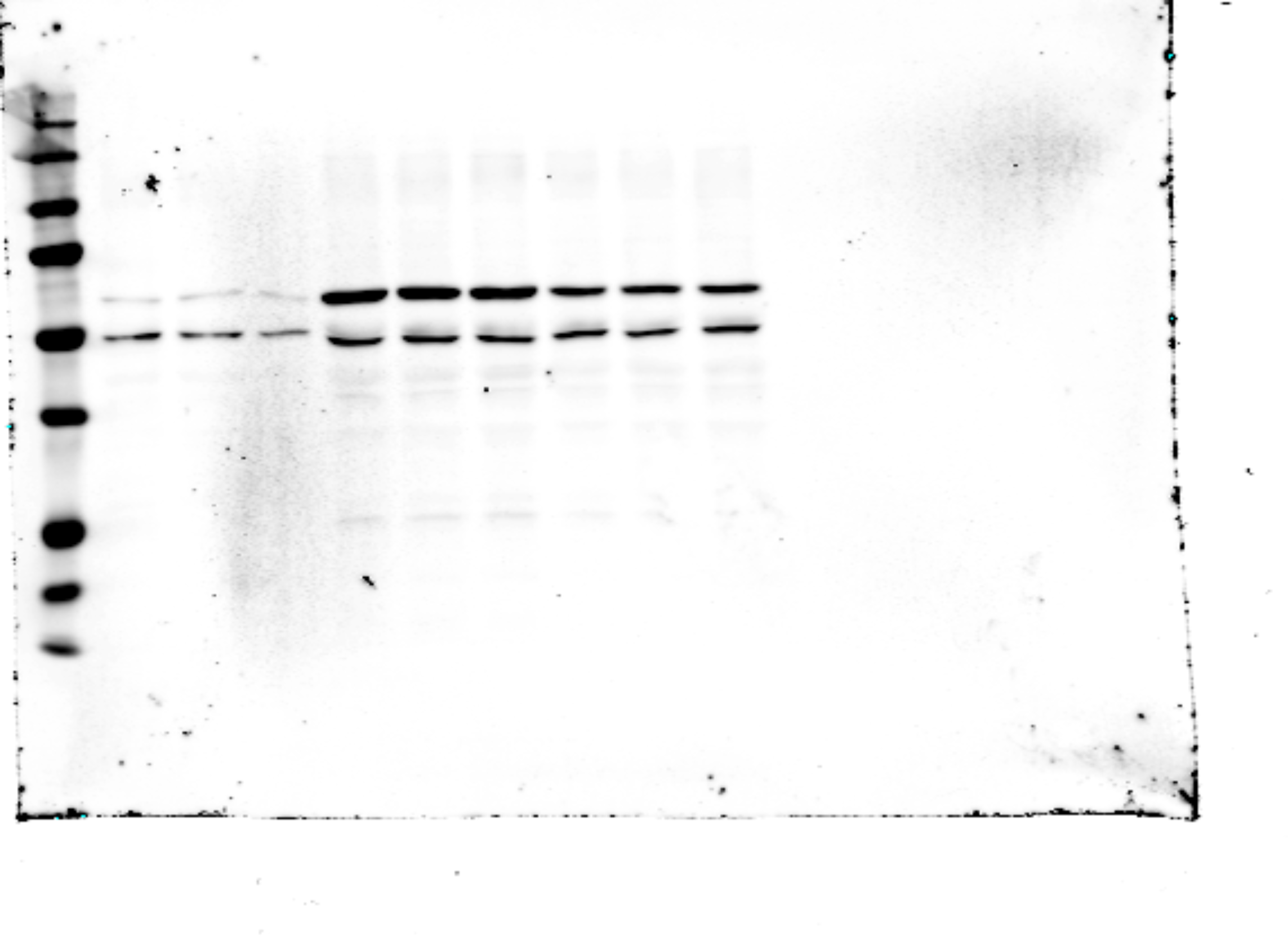

Supplement: Supplementary file 13 — Raw Western Blot and Microscopy Images [file 44318_2026_809_MOESM13_ESM.zip › SD_Blots/SD Figure 2G/2G BACE1.tif]

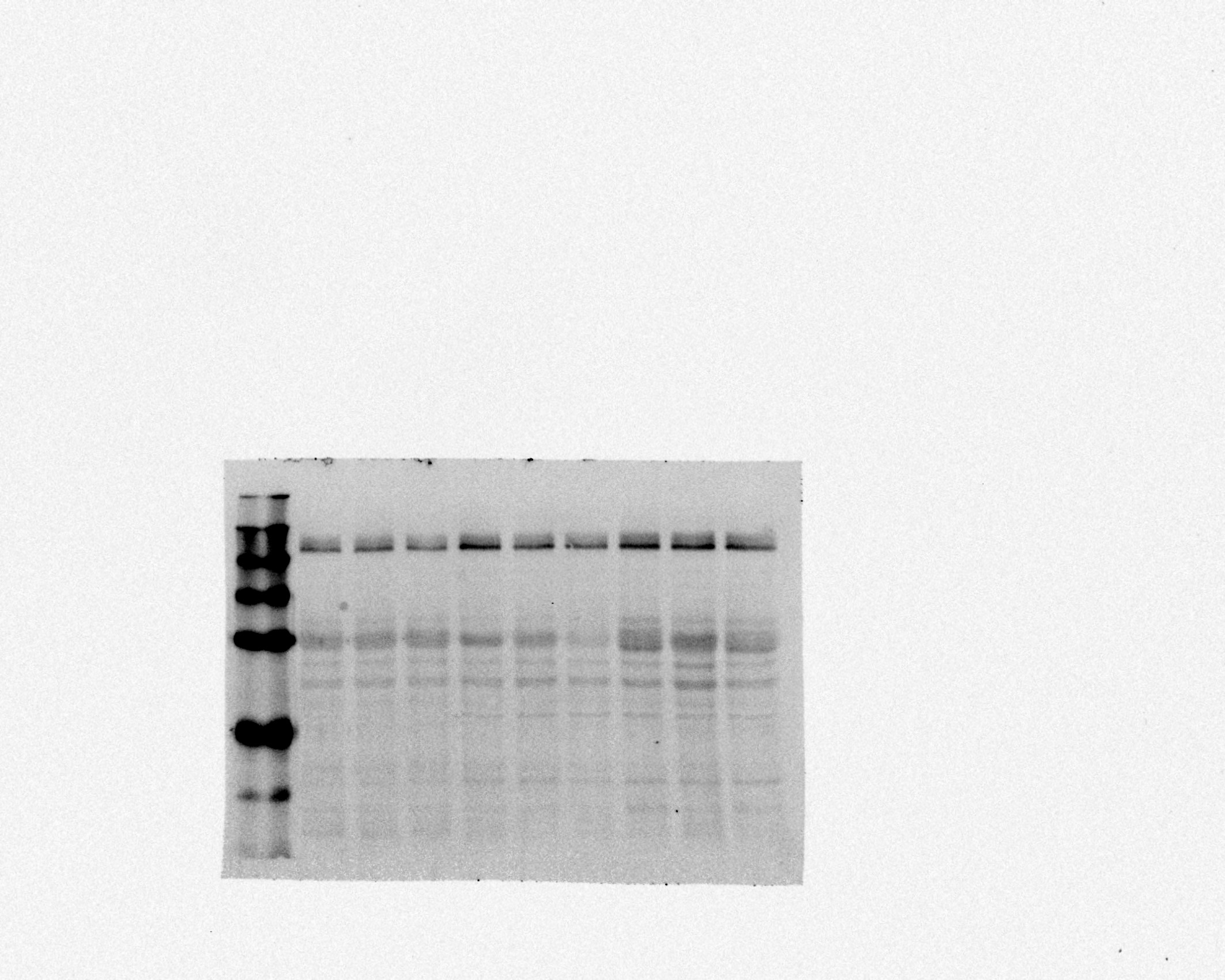

Supplement: Supplementary file 13 — Raw Western Blot and Microscopy Images [file 44318_2026_809_MOESM13_ESM.zip › SD_Blots/SD Figure 2G/2G C99.jpg]

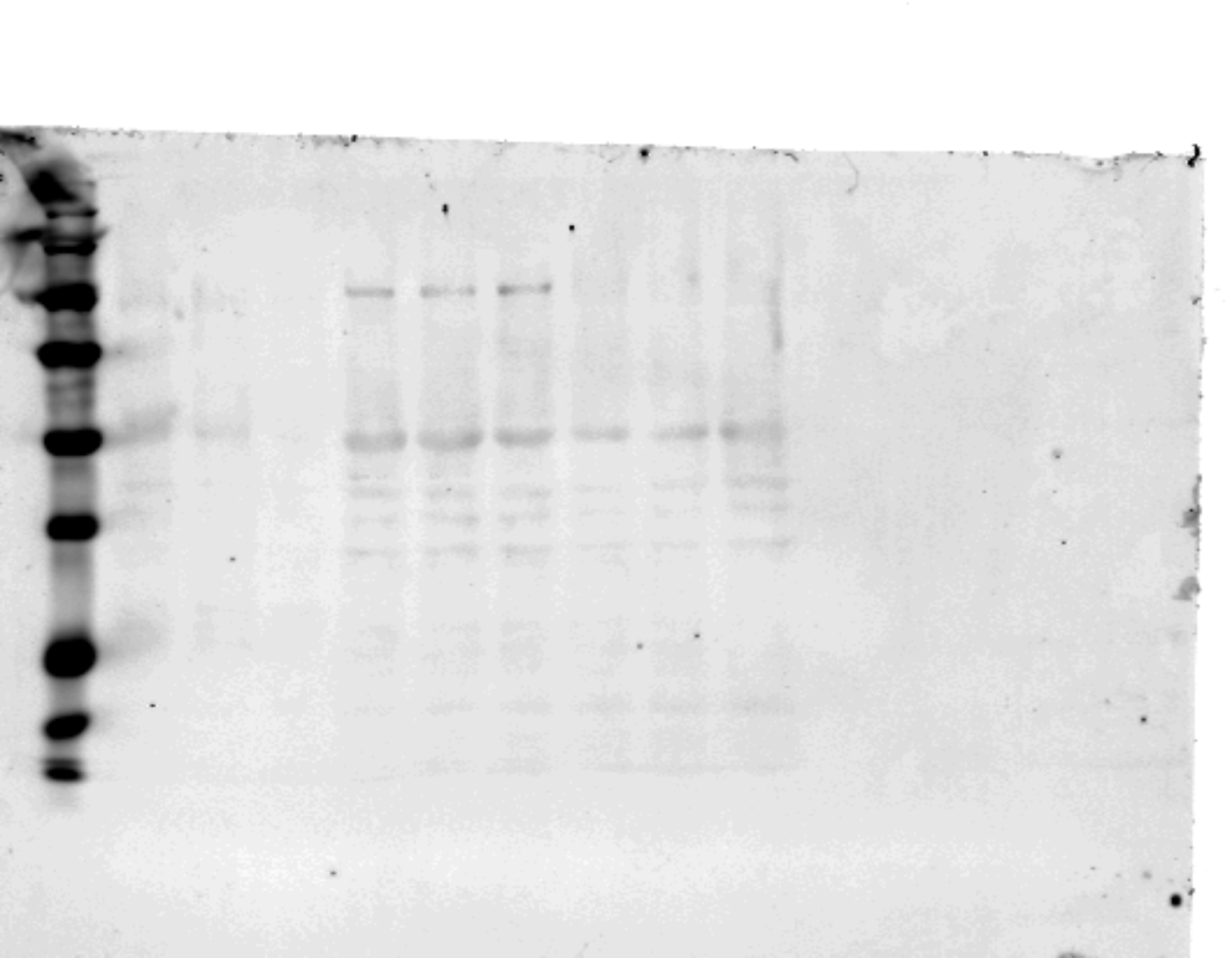

Supplement: Supplementary file 13 — Raw Western Blot and Microscopy Images [file 44318_2026_809_MOESM13_ESM.zip › SD_Blots/SD Figure 2G/2G NCT.tif]

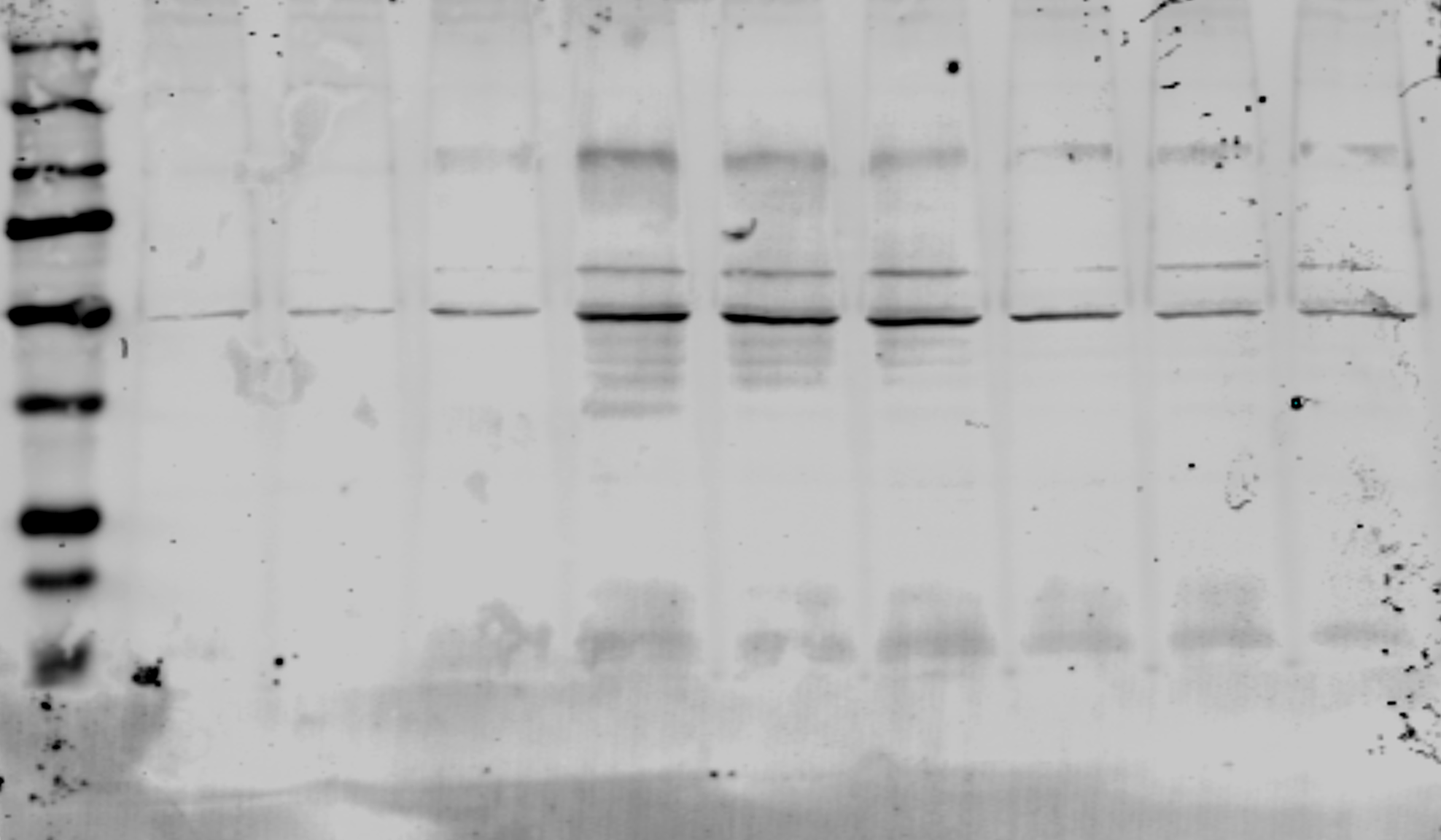

Supplement: Supplementary file 13 — Raw Western Blot and Microscopy Images [file 44318_2026_809_MOESM13_ESM.zip › SD_Blots/SD Figure 2G/2G PS1.tif]

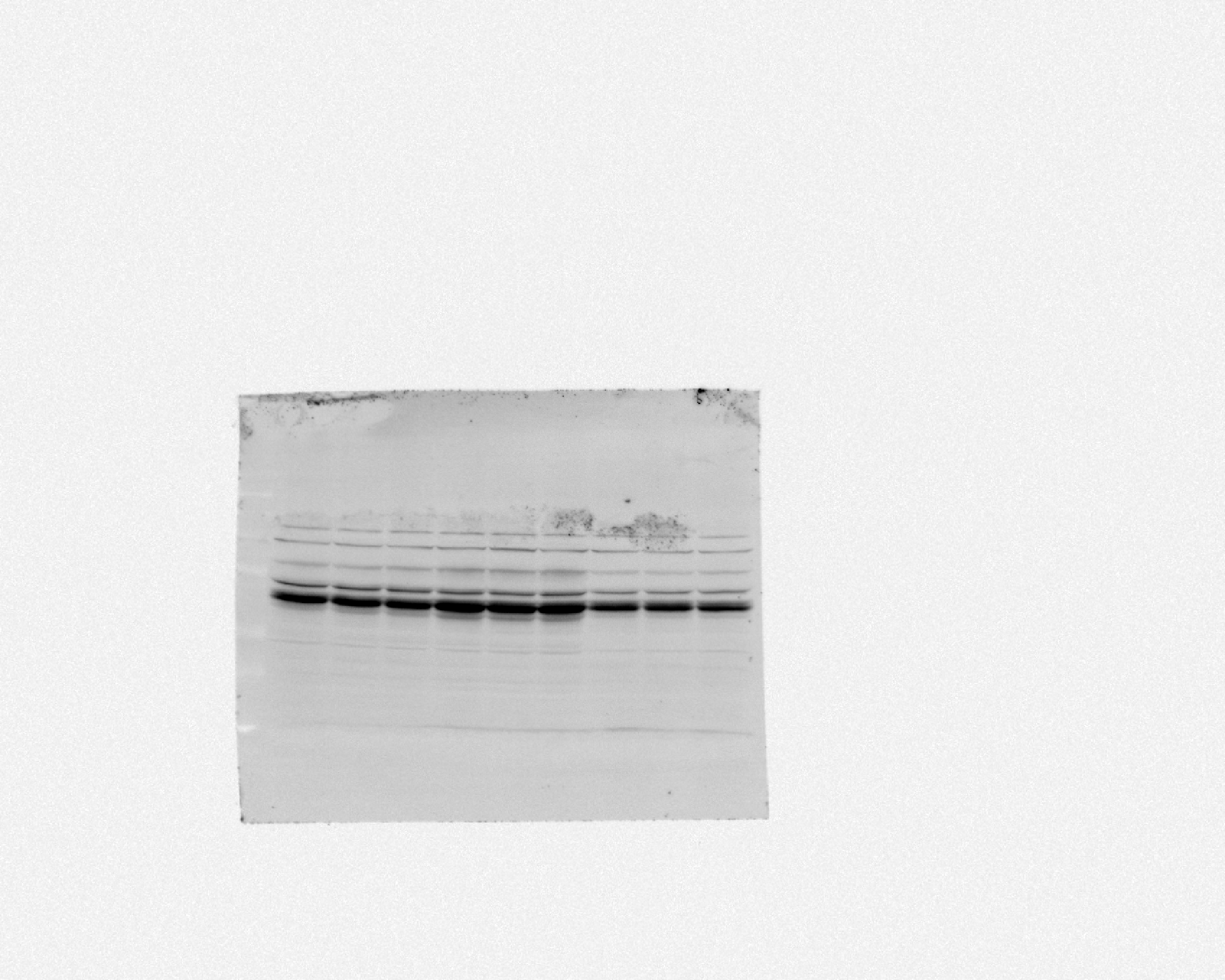

Supplement: Supplementary file 13 — Raw Western Blot and Microscopy Images [file 44318_2026_809_MOESM13_ESM.zip › SD_Blots/SD Figure 2G/2G sAPP B.tif]

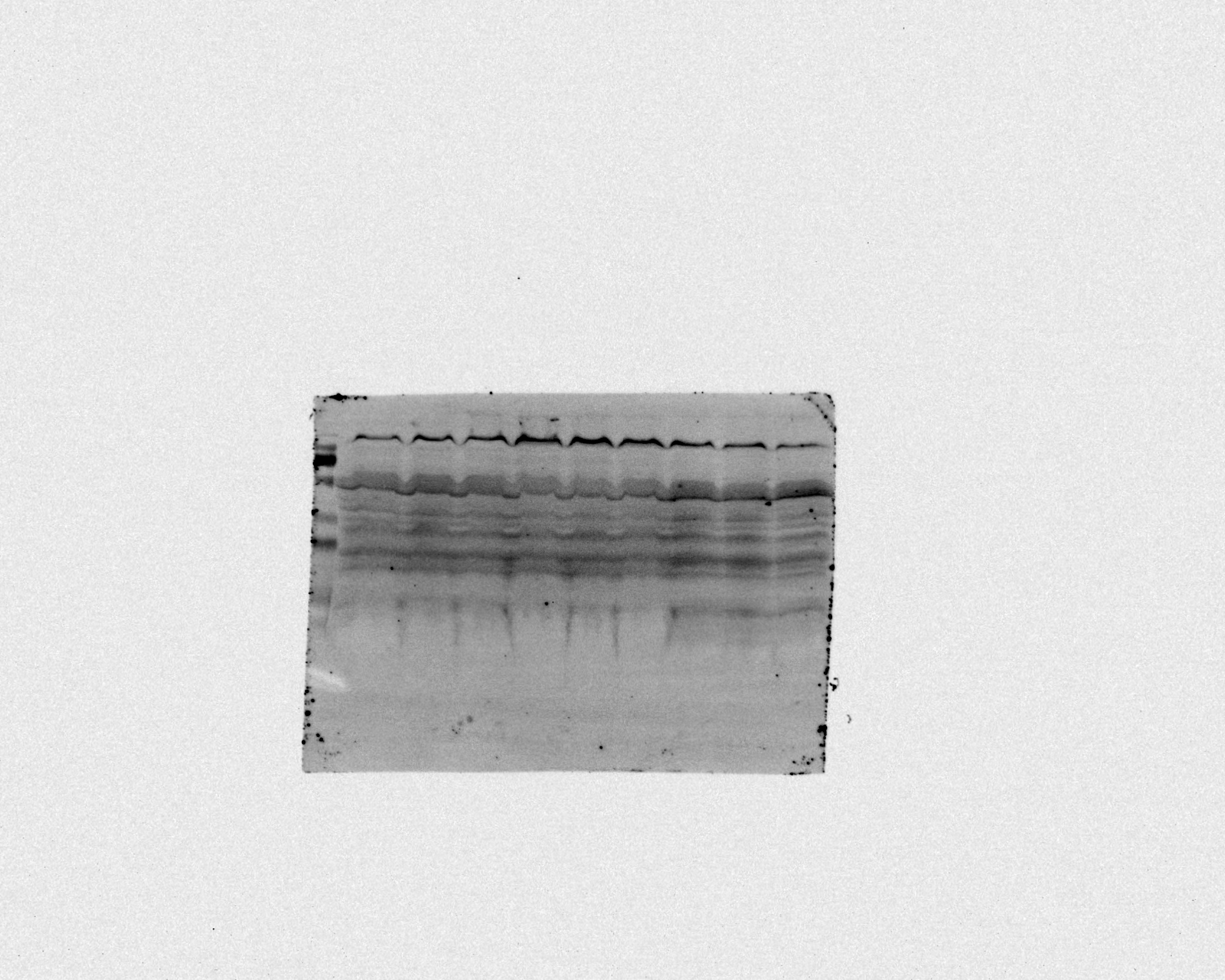

Supplement: Supplementary file 13 — Raw Western Blot and Microscopy Images [file 44318_2026_809_MOESM13_ESM.zip › SD_Blots/SD Figure 2G/2G sAPPa.tif]

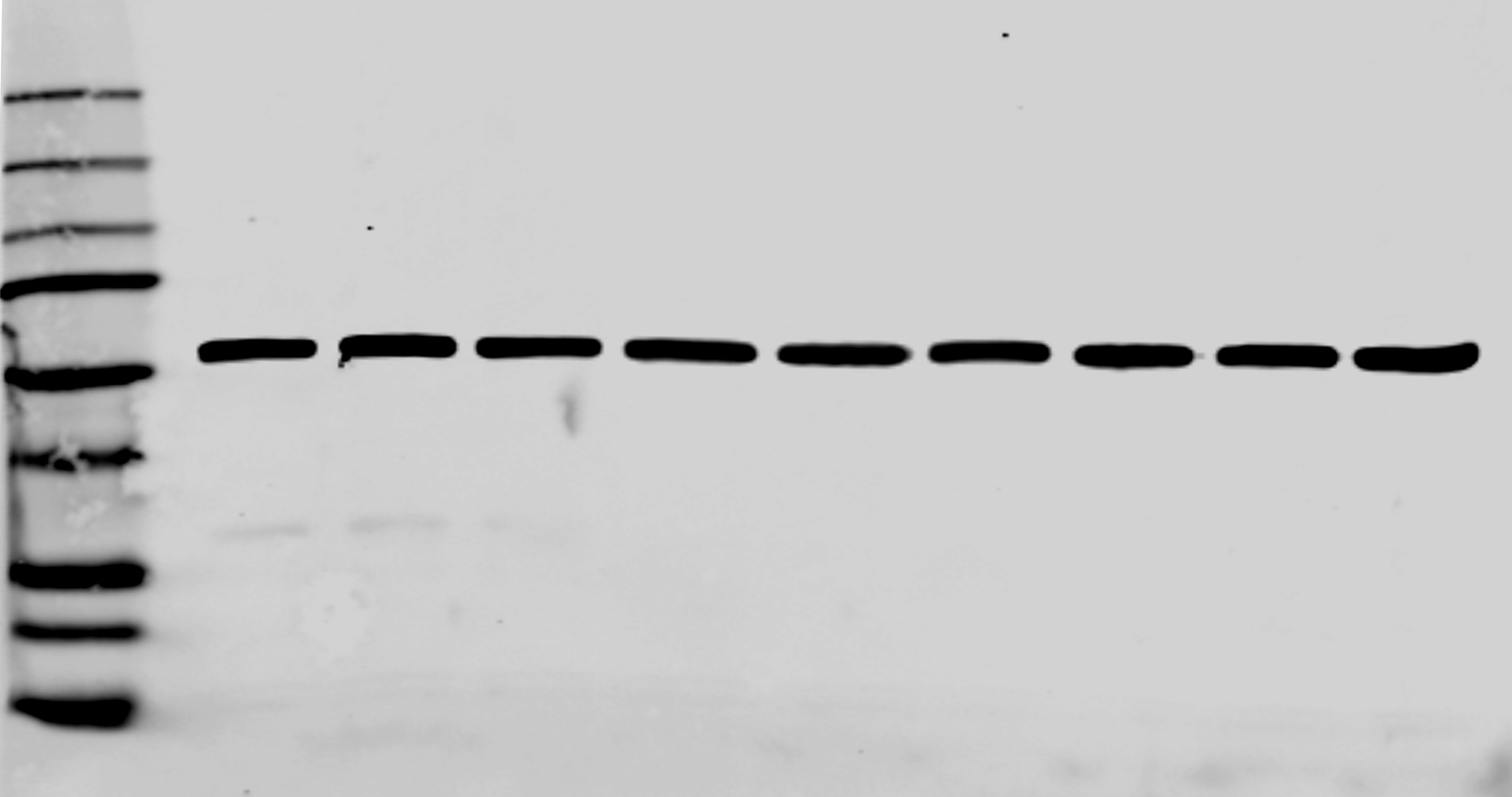

Supplement: Supplementary file 13 — Raw Western Blot and Microscopy Images [file 44318_2026_809_MOESM13_ESM.zip › SD_Blots/SD Figure 2G/2G Tubulin.tif]

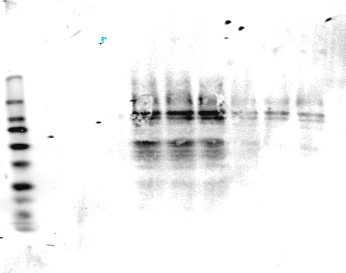

Supplement: Supplementary file 13 — Raw Western Blot and Microscopy Images [file 44318_2026_809_MOESM13_ESM.zip › SD_Blots/SD Figure 3A/3A AT180.jpg]

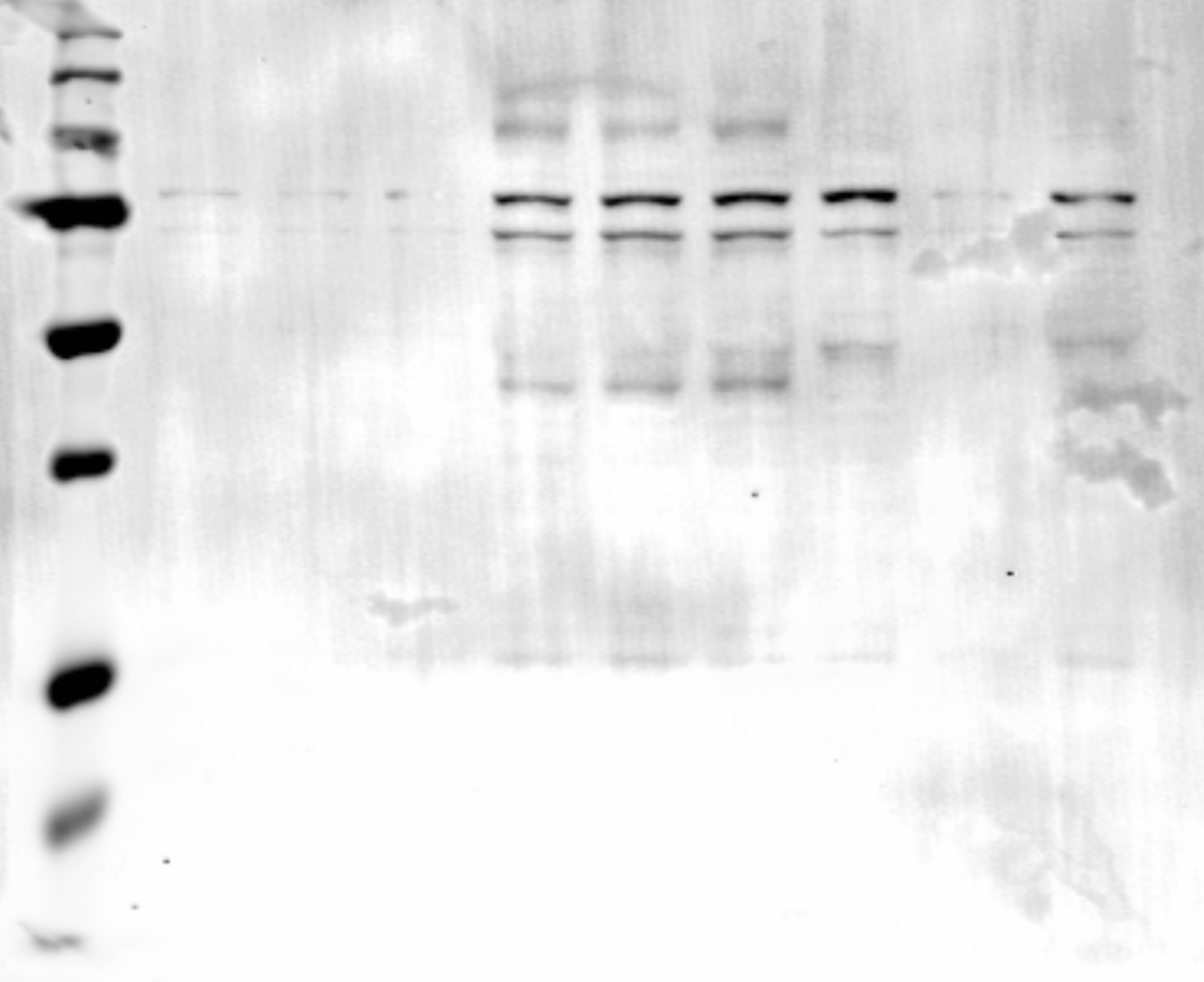

Supplement: Supplementary file 13 — Raw Western Blot and Microscopy Images [file 44318_2026_809_MOESM13_ESM.zip › SD_Blots/SD Figure 3A/3A AT270.tif.tif]

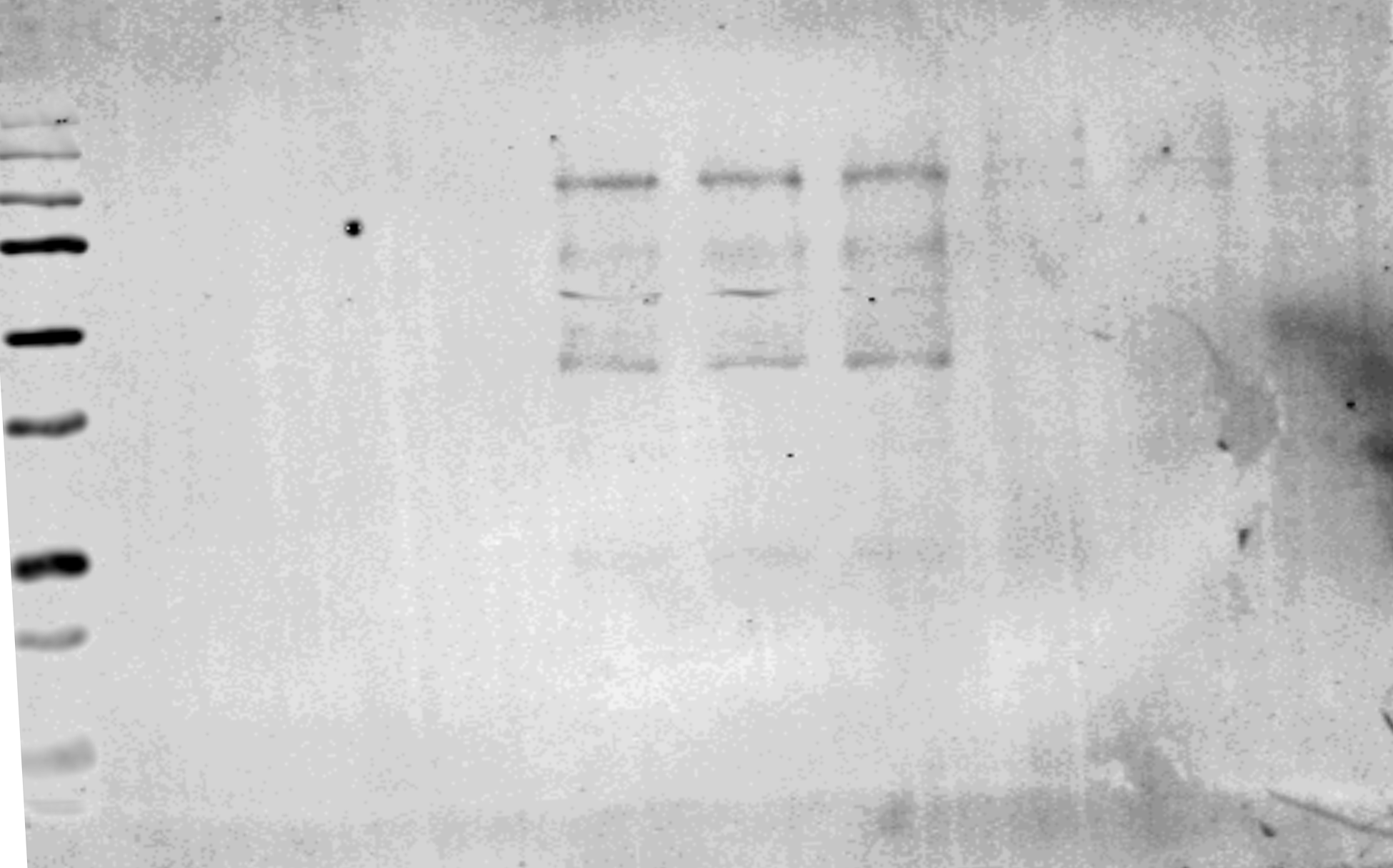

Supplement: Supplementary file 13 — Raw Western Blot and Microscopy Images [file 44318_2026_809_MOESM13_ESM.zip › SD_Blots/SD Figure 3A/3A AT8.tif]

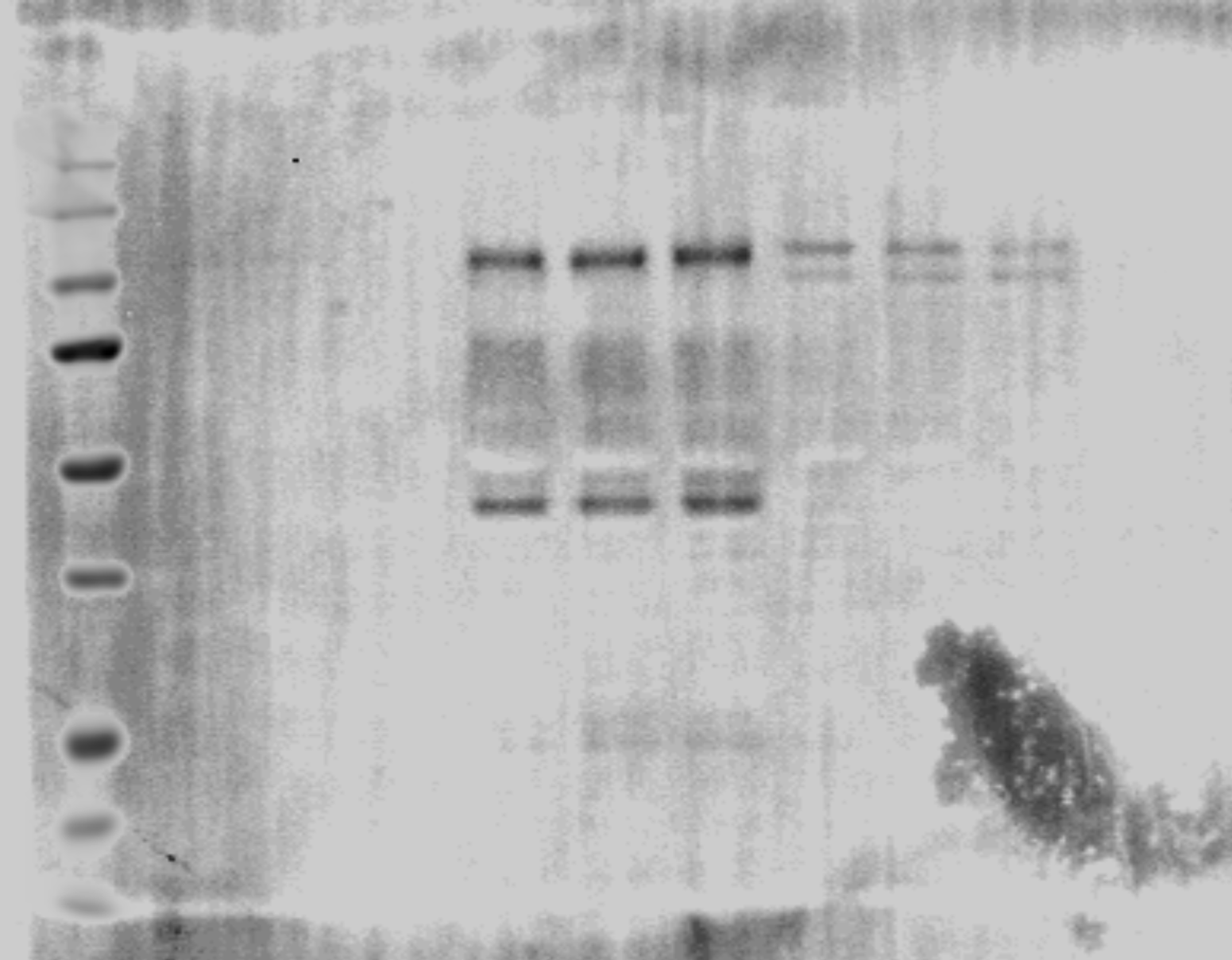

Supplement: Supplementary file 13 — Raw Western Blot and Microscopy Images [file 44318_2026_809_MOESM13_ESM.zip › SD_Blots/SD Figure 3A/3A HT7 Insoluble.tif]

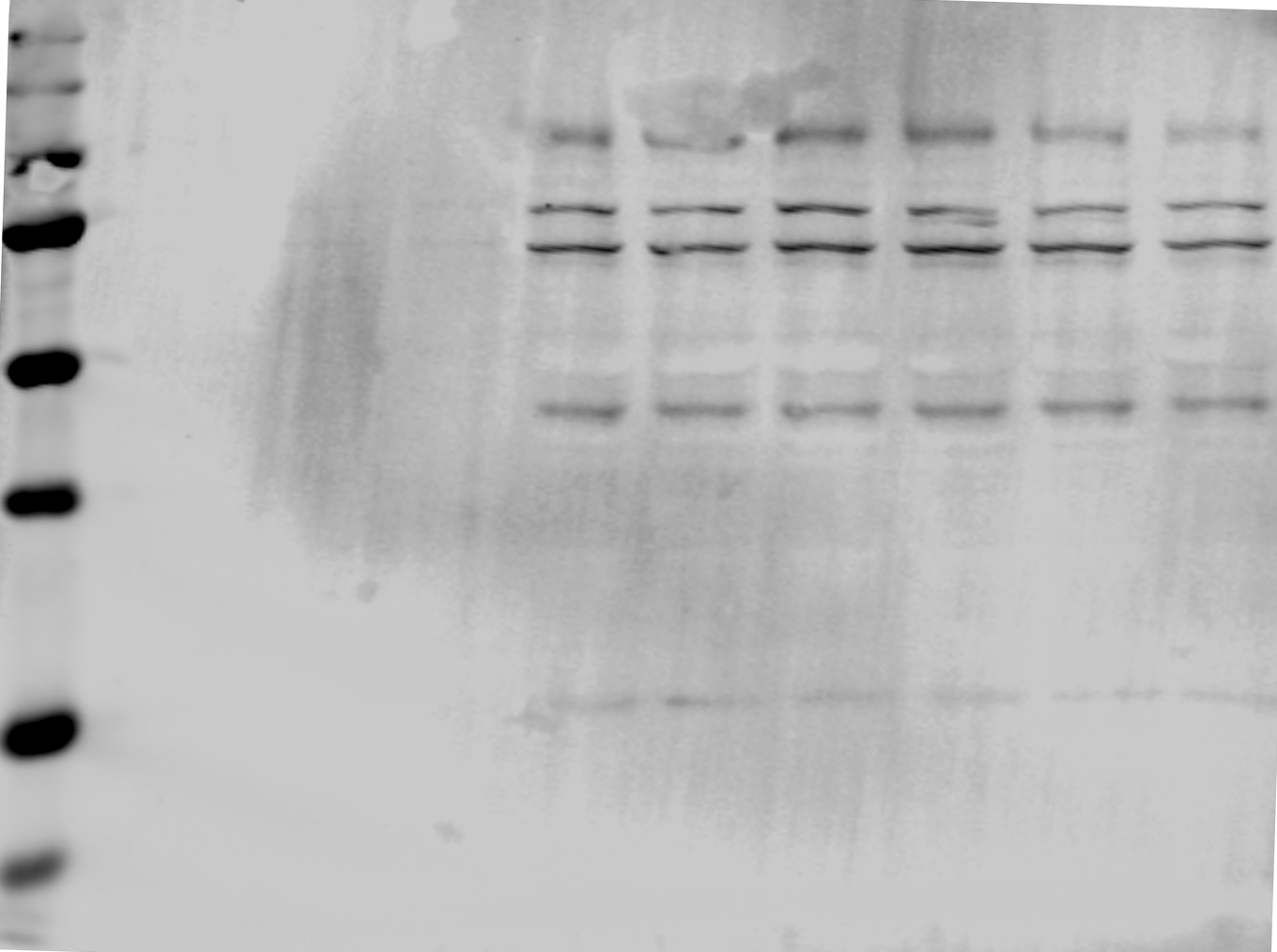

Supplement: Supplementary file 13 — Raw Western Blot and Microscopy Images [file 44318_2026_809_MOESM13_ESM.zip › SD_Blots/SD Figure 3A/3A HT7 Soluble.tif]

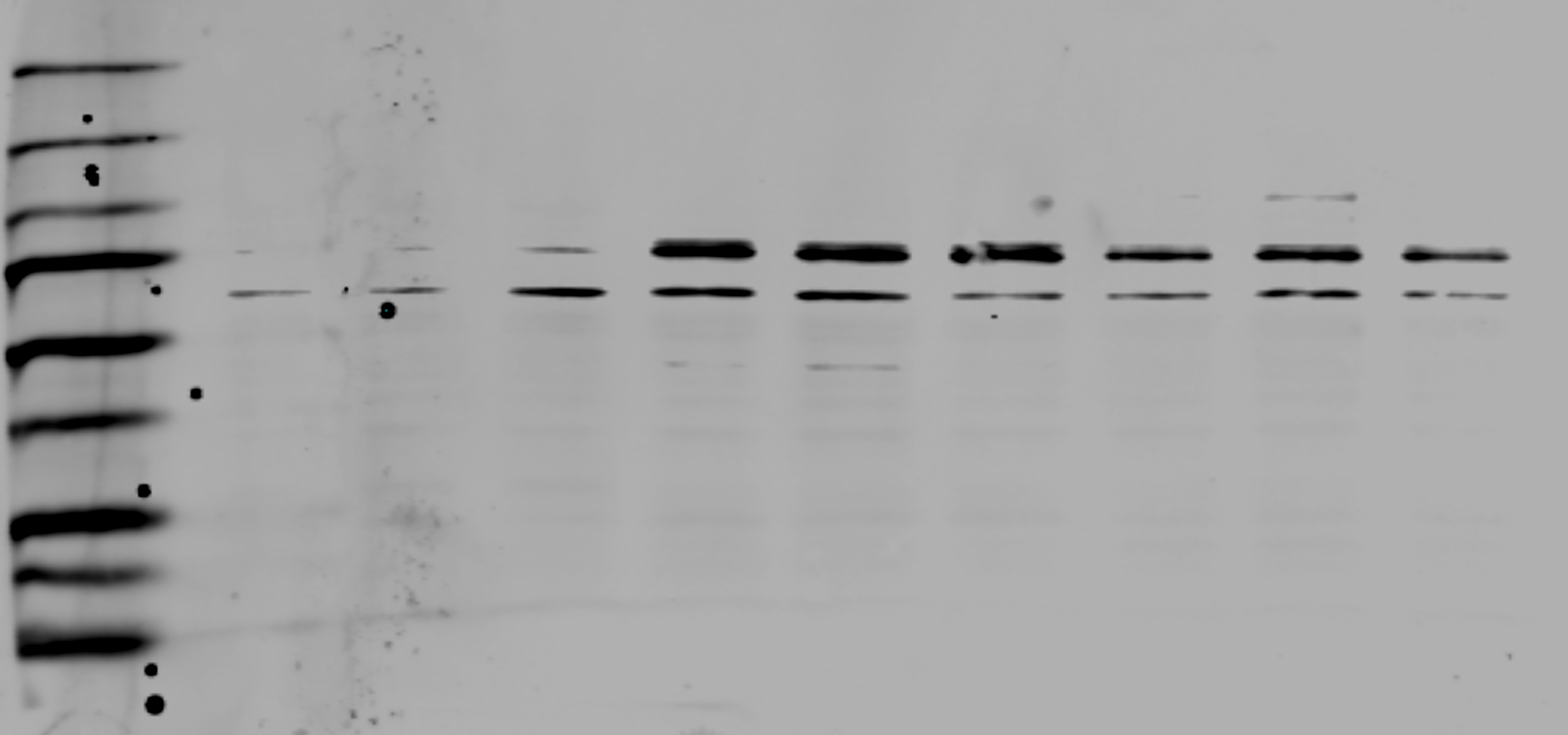

Supplement: Supplementary file 13 — Raw Western Blot and Microscopy Images [file 44318_2026_809_MOESM13_ESM.zip › SD_Blots/SD Figure 3A/3A PHF13.tif]

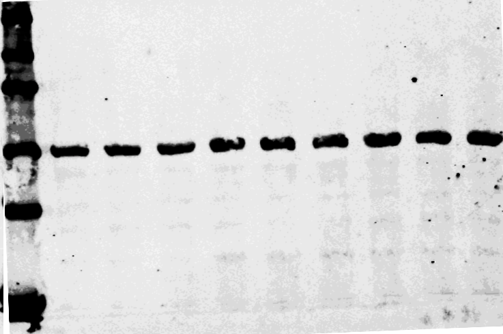

Supplement: Supplementary file 13 — Raw Western Blot and Microscopy Images [file 44318_2026_809_MOESM13_ESM.zip › SD_Blots/SD Figure 3A/3A tubulin.png]

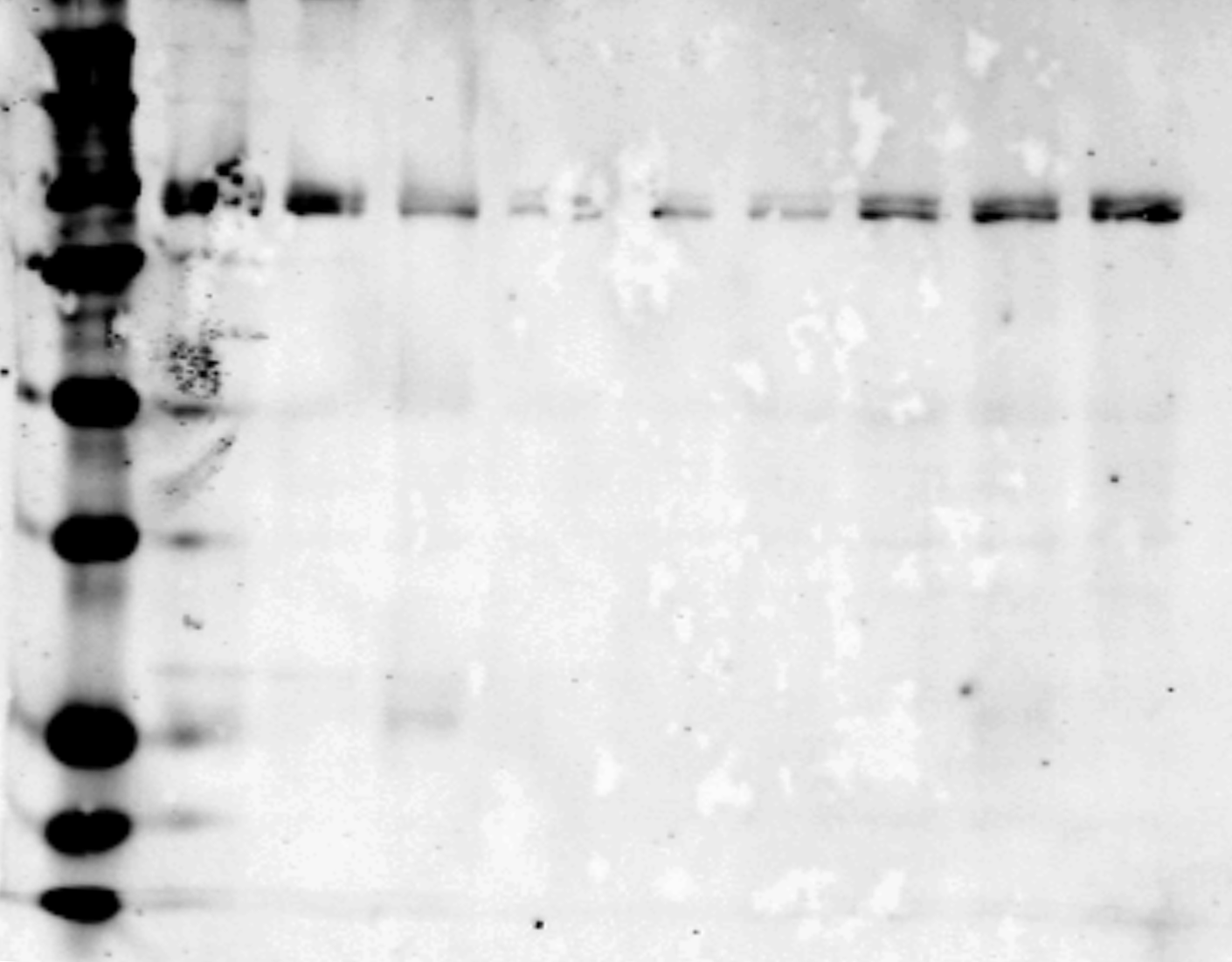

Supplement: Supplementary file 13 — Raw Western Blot and Microscopy Images [file 44318_2026_809_MOESM13_ESM.zip › SD_Blots/SD Figure 4G/4G PSD-95.tif.tif]

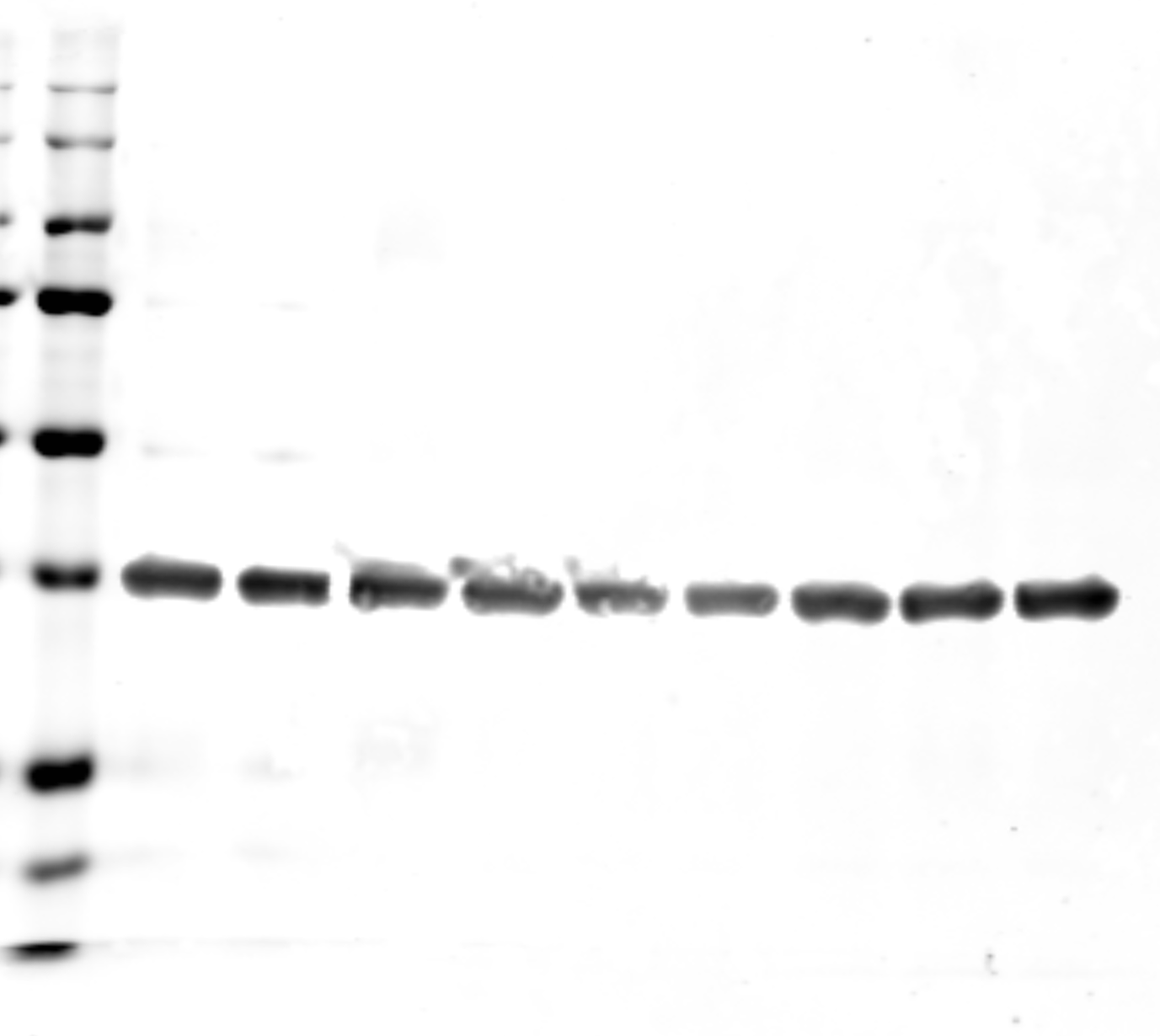

Supplement: Supplementary file 13 — Raw Western Blot and Microscopy Images [file 44318_2026_809_MOESM13_ESM.zip › SD_Blots/SD Figure 4G/4G SYP.tif]

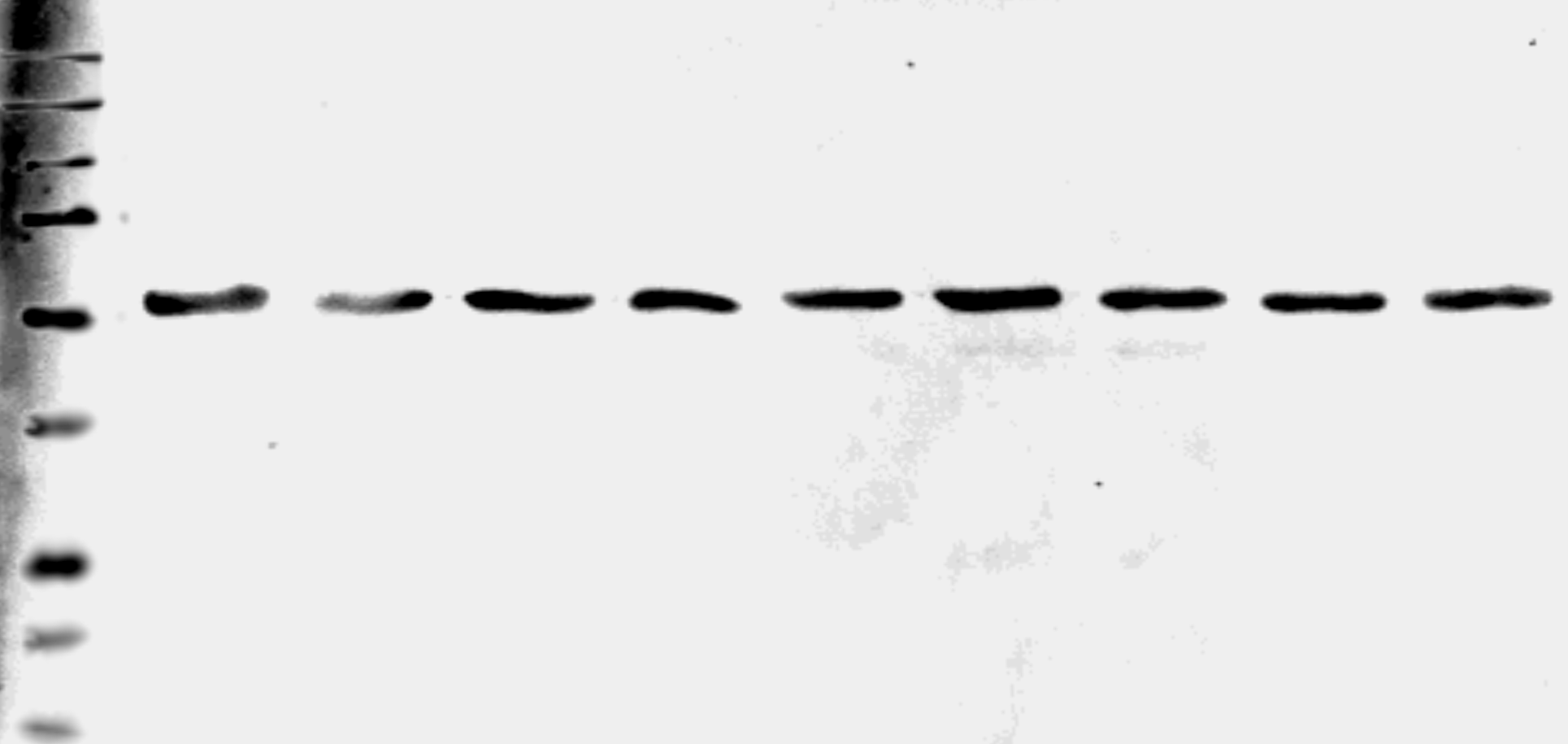

Supplement: Supplementary file 13 — Raw Western Blot and Microscopy Images [file 44318_2026_809_MOESM13_ESM.zip › SD_Blots/SD Figure 4G/4G Tubulin.tif]

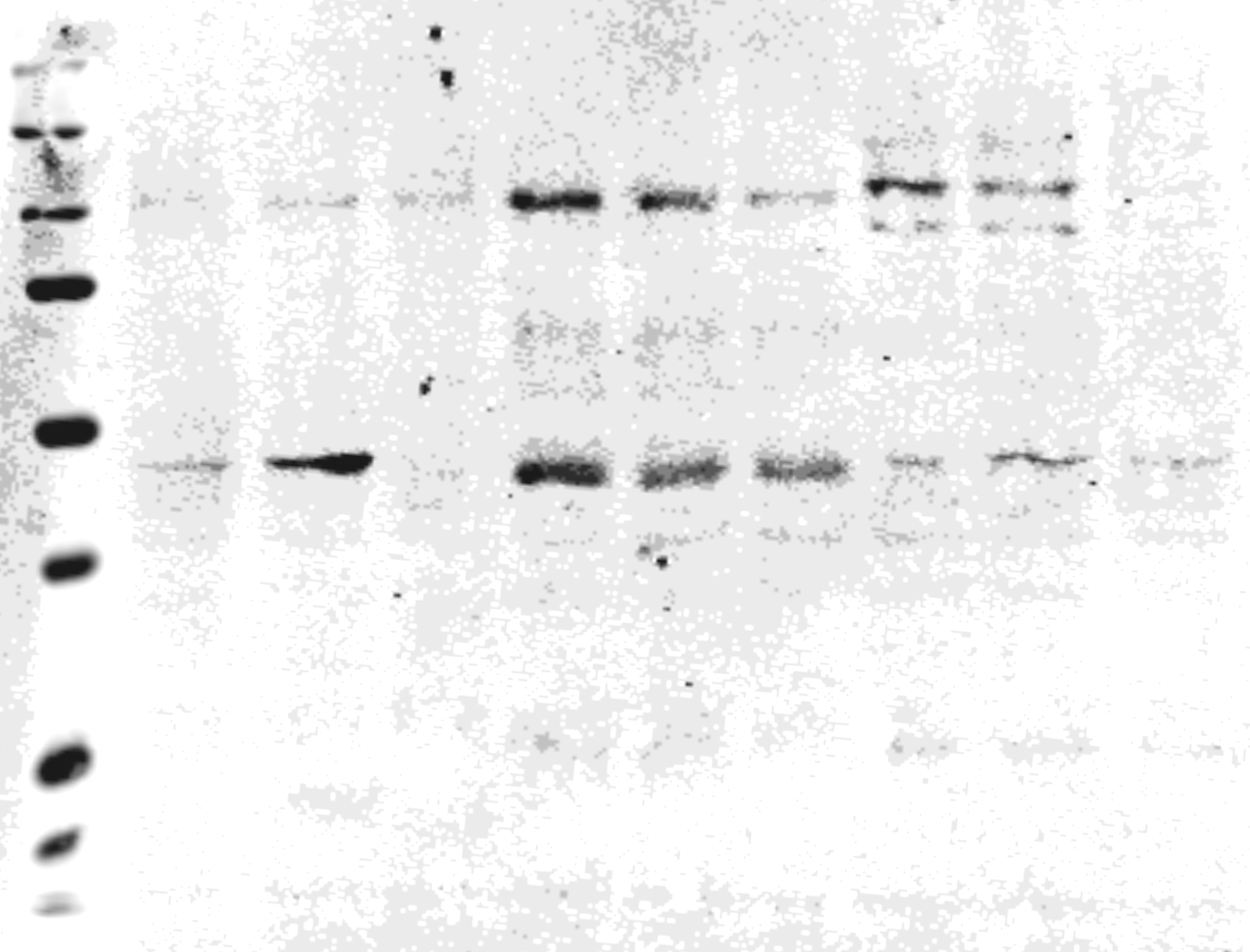

Supplement: Supplementary file 13 — Raw Western Blot and Microscopy Images [file 44318_2026_809_MOESM13_ESM.zip › SD_Blots/SD Figure 4H/4H GFAP.tif]

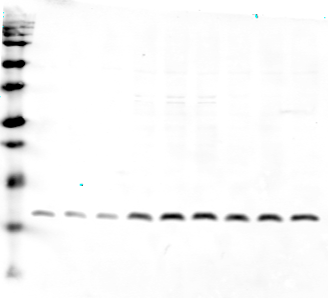

Supplement: Supplementary file 13 — Raw Western Blot and Microscopy Images [file 44318_2026_809_MOESM13_ESM.zip › SD_Blots/SD Figure 4H/4H iba-1 .tif.tif]

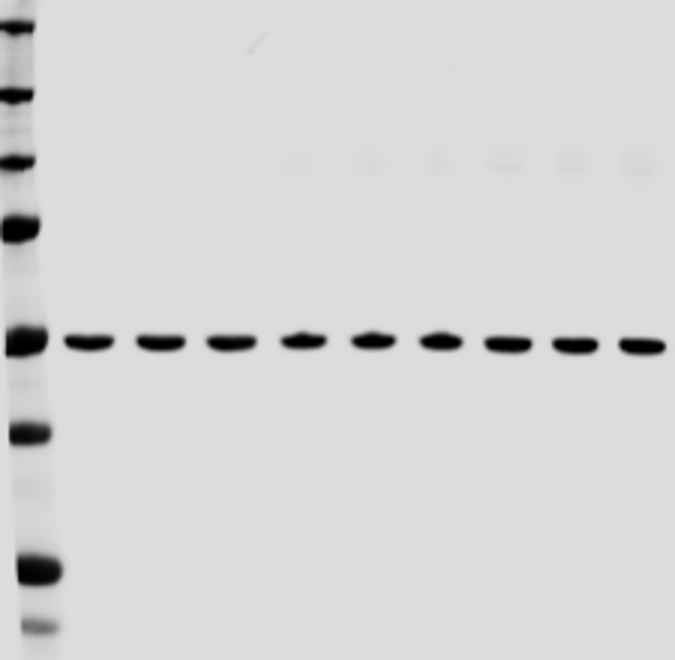

Supplement: Supplementary file 13 — Raw Western Blot and Microscopy Images [file 44318_2026_809_MOESM13_ESM.zip › SD_Blots/SD Figure 4H/4H tubulin.tif]

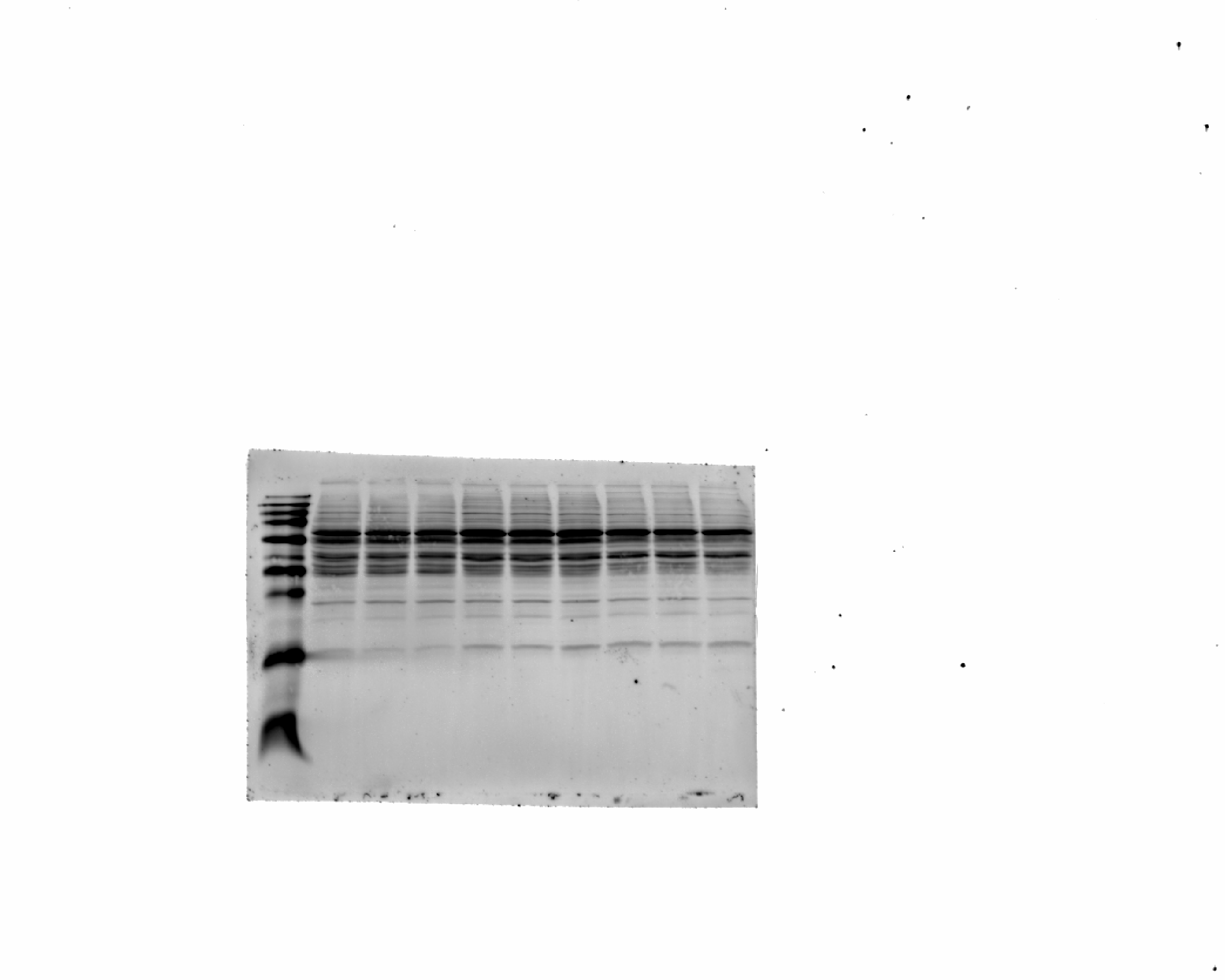

Supplement: Supplementary file 13 — Raw Western Blot and Microscopy Images [file 44318_2026_809_MOESM13_ESM.zip › SD_Blots/SD Figure 4I/4I -IBA-1.tif]

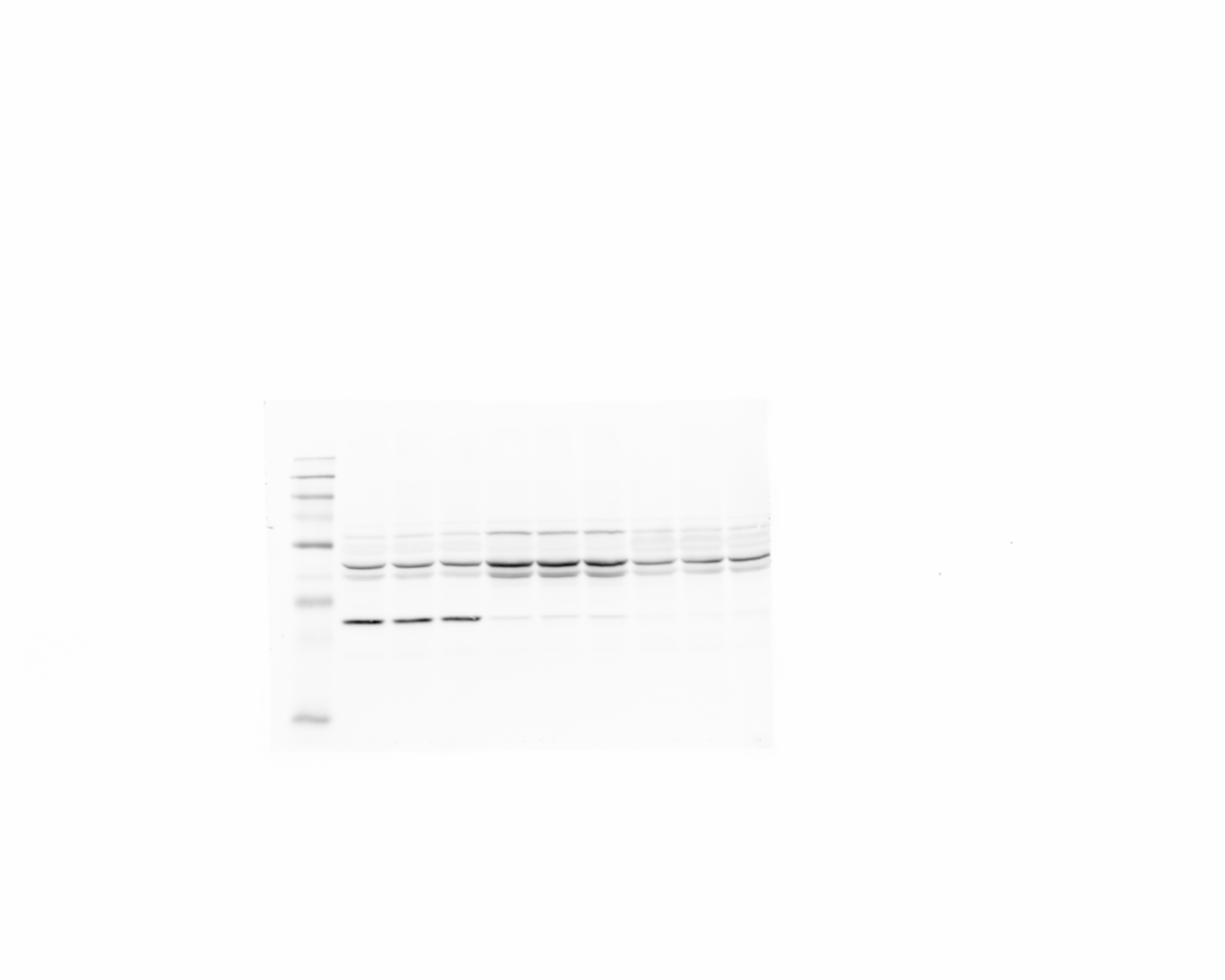

Supplement: Supplementary file 13 — Raw Western Blot and Microscopy Images [file 44318_2026_809_MOESM13_ESM.zip › SD_Blots/SD Figure 4I/4I GFAP.tif]

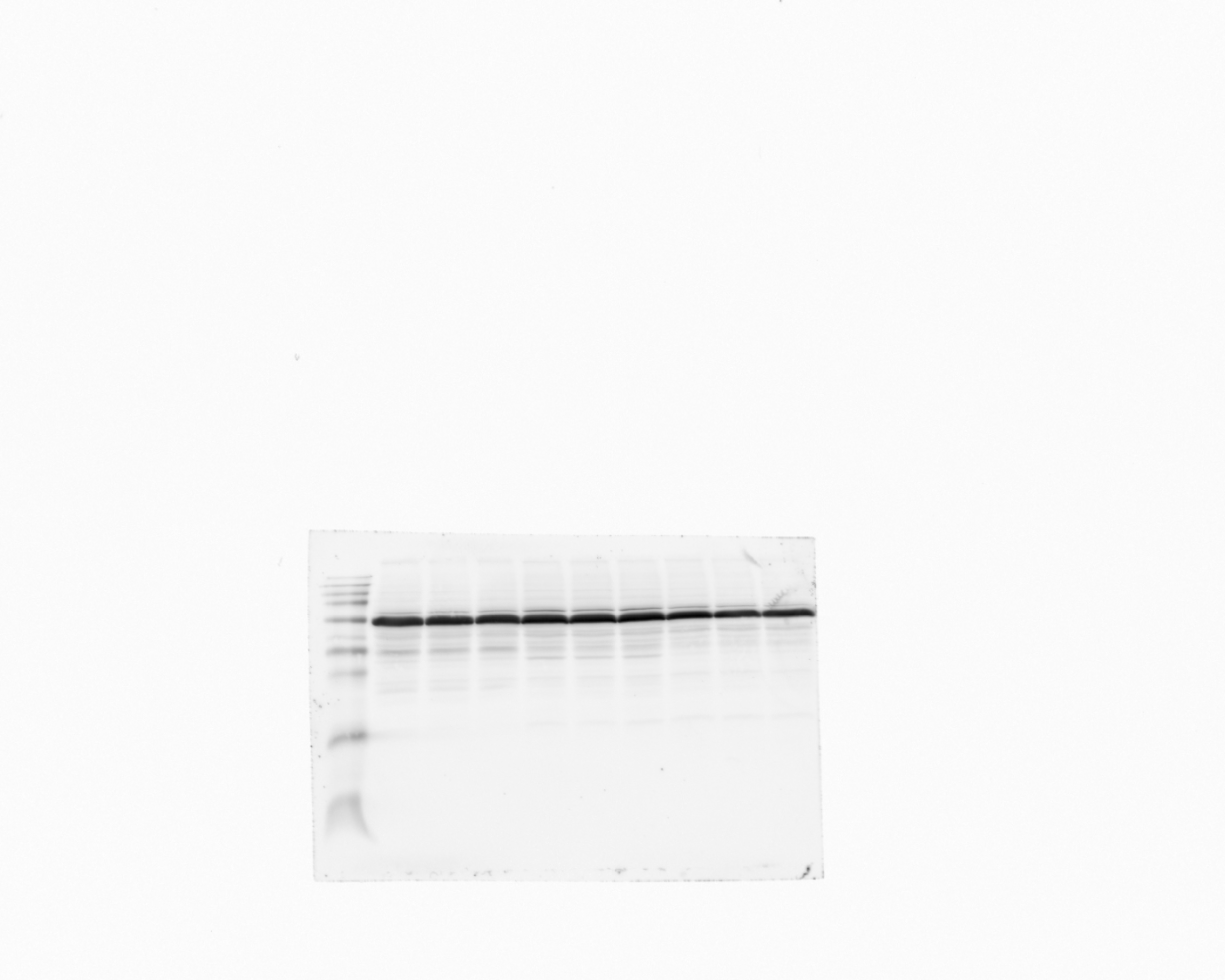

Supplement: Supplementary file 13 — Raw Western Blot and Microscopy Images [file 44318_2026_809_MOESM13_ESM.zip › SD_Blots/SD Figure 4I/4I Tubulin.tif]

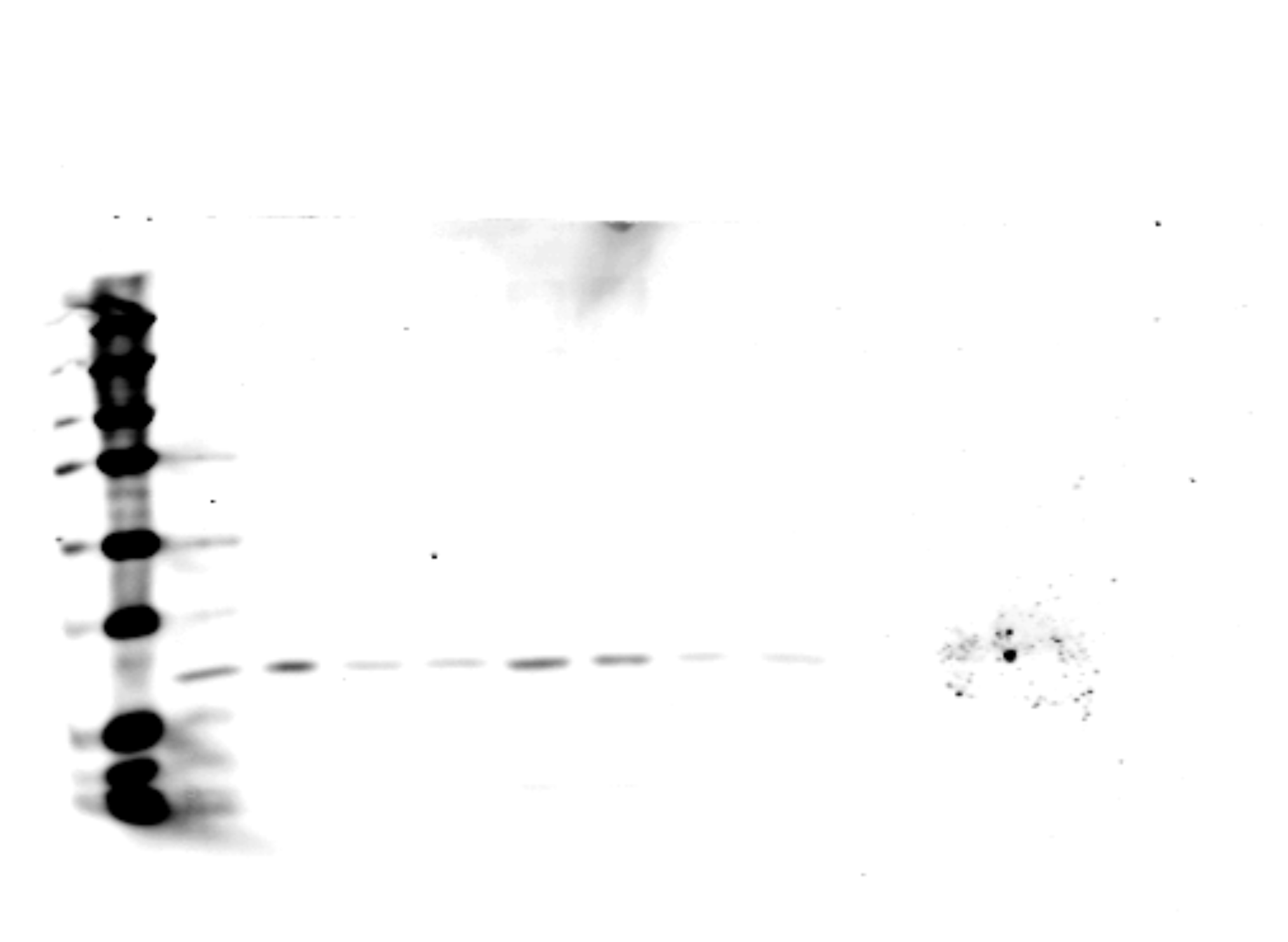

Supplement: Supplementary file 13 — Raw Western Blot and Microscopy Images [file 44318_2026_809_MOESM13_ESM.zip › SD_Blots/SD Figure 5B/5B MCU.tif]

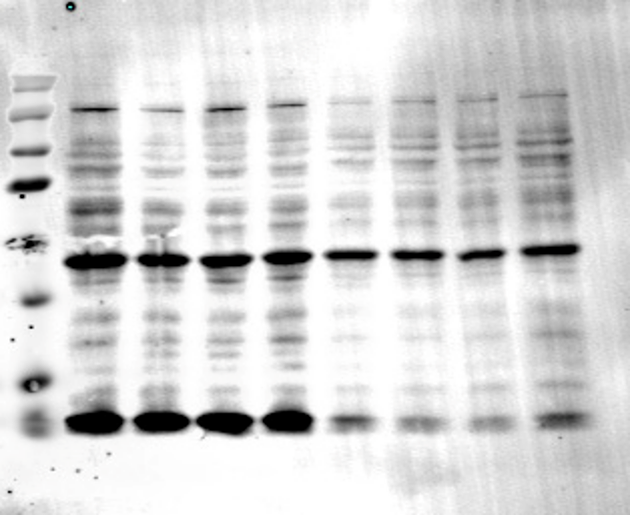

Supplement: Supplementary file 13 — Raw Western Blot and Microscopy Images [file 44318_2026_809_MOESM13_ESM.zip › SD_Blots/SD Figure 5B/5B MCUB.tif]

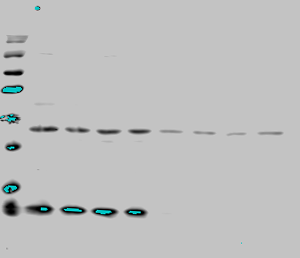

Supplement: Supplementary file 13 — Raw Western Blot and Microscopy Images [file 44318_2026_809_MOESM13_ESM.zip › SD_Blots/SD Figure 5B/5B MICU1.tif]

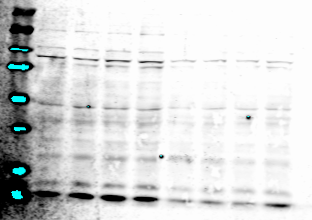

Supplement: Supplementary file 13 — Raw Western Blot and Microscopy Images [file 44318_2026_809_MOESM13_ESM.zip › SD_Blots/SD Figure 5B/5B MICU2.tif.tif]

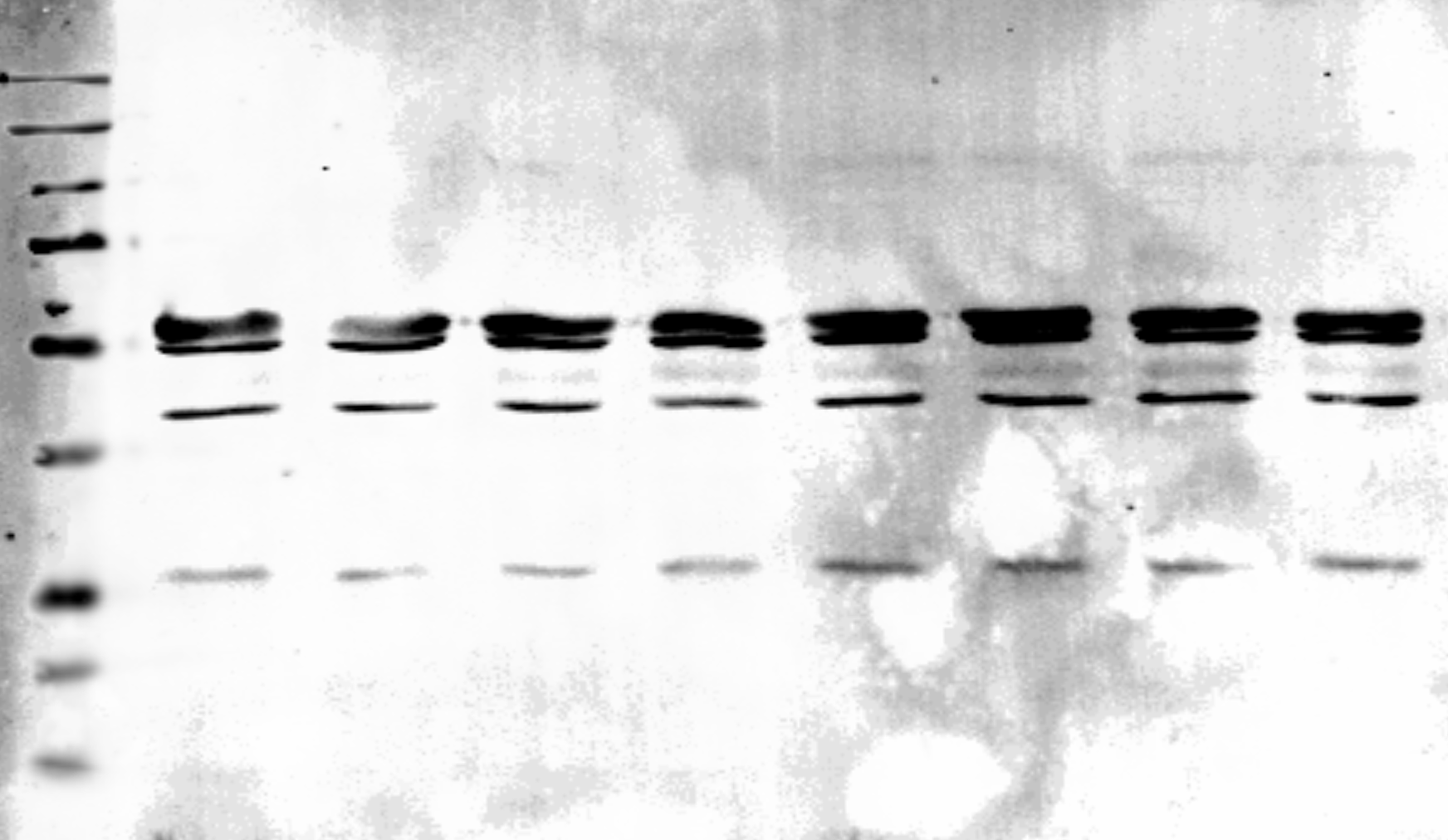

Supplement: Supplementary file 13 — Raw Western Blot and Microscopy Images [file 44318_2026_809_MOESM13_ESM.zip › SD_Blots/SD Figure 5B/5B Oxphos.tif.tif]

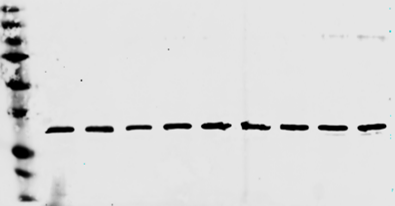

Supplement: Supplementary file 13 — Raw Western Blot and Microscopy Images [file 44318_2026_809_MOESM13_ESM.zip › SD_Blots/SD Figure 5B/5B VDAC.tif]

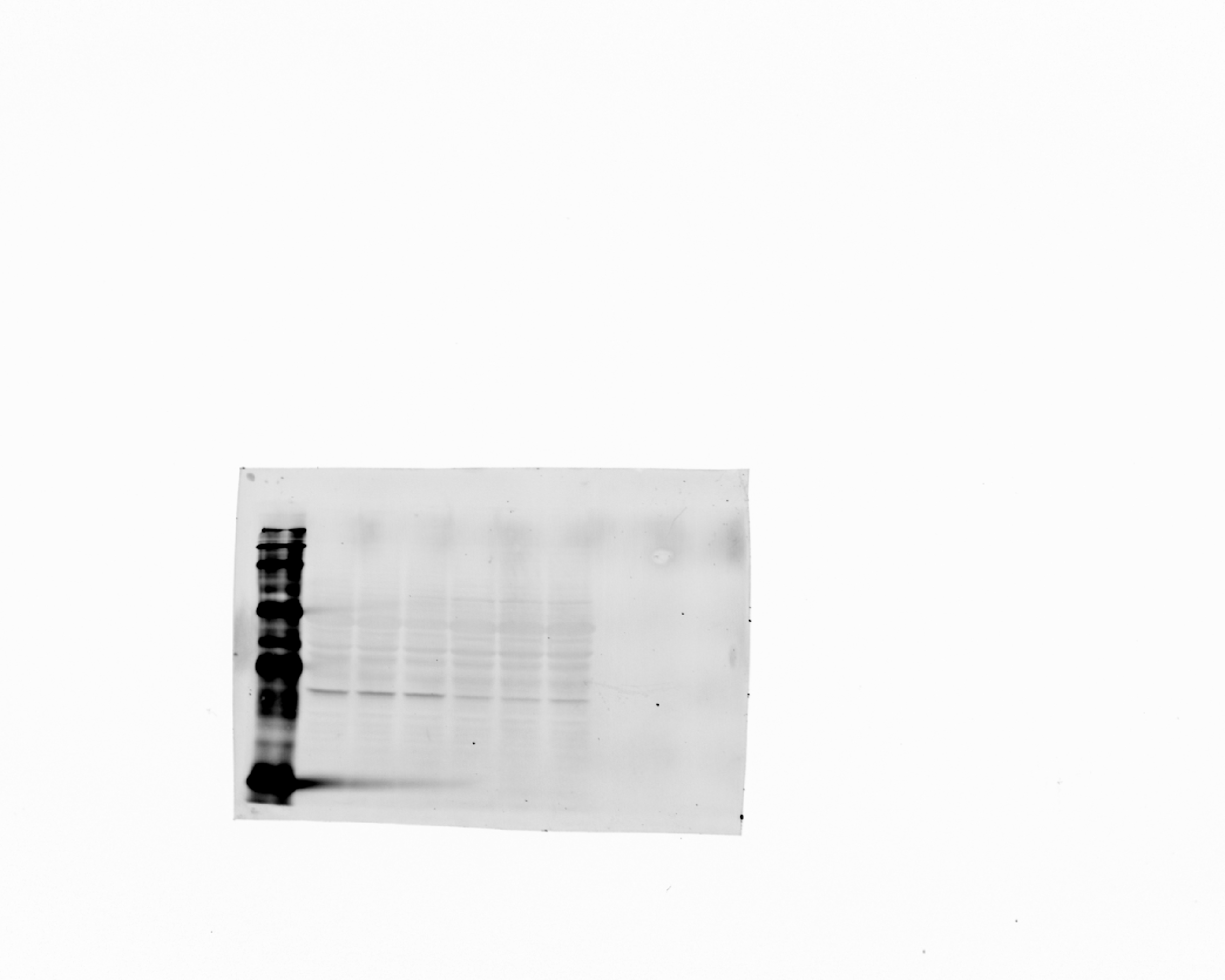

Supplement: Supplementary file 13 — Raw Western Blot and Microscopy Images [file 44318_2026_809_MOESM13_ESM.zip › SD_Blots/SD S1 O/S10-MCU.jpg]

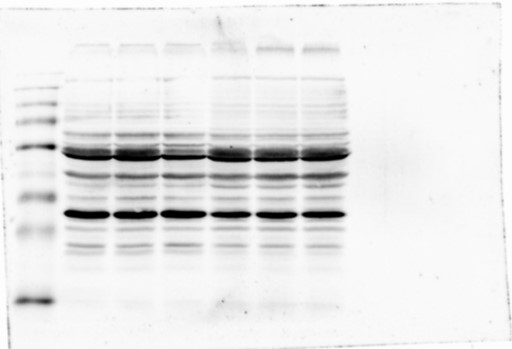

Supplement: Supplementary file 13 — Raw Western Blot and Microscopy Images [file 44318_2026_809_MOESM13_ESM.zip › SD_Blots/SD S1 O/S1O VDAC.jpg]

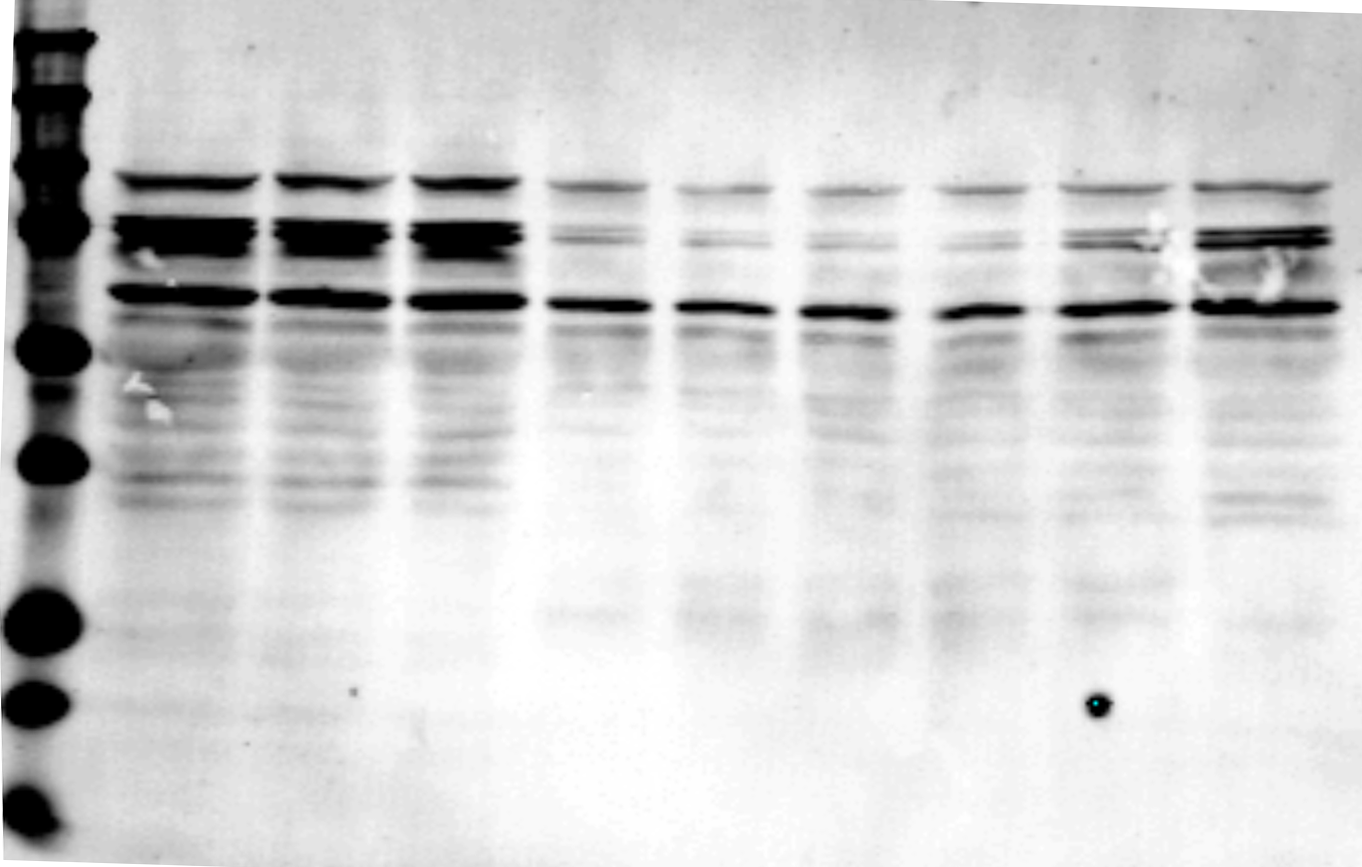

Supplement: Supplementary file 13 — Raw Western Blot and Microscopy Images [file 44318_2026_809_MOESM13_ESM.zip › SD_Blots/SD S2C/S2C ADAM10.tif]

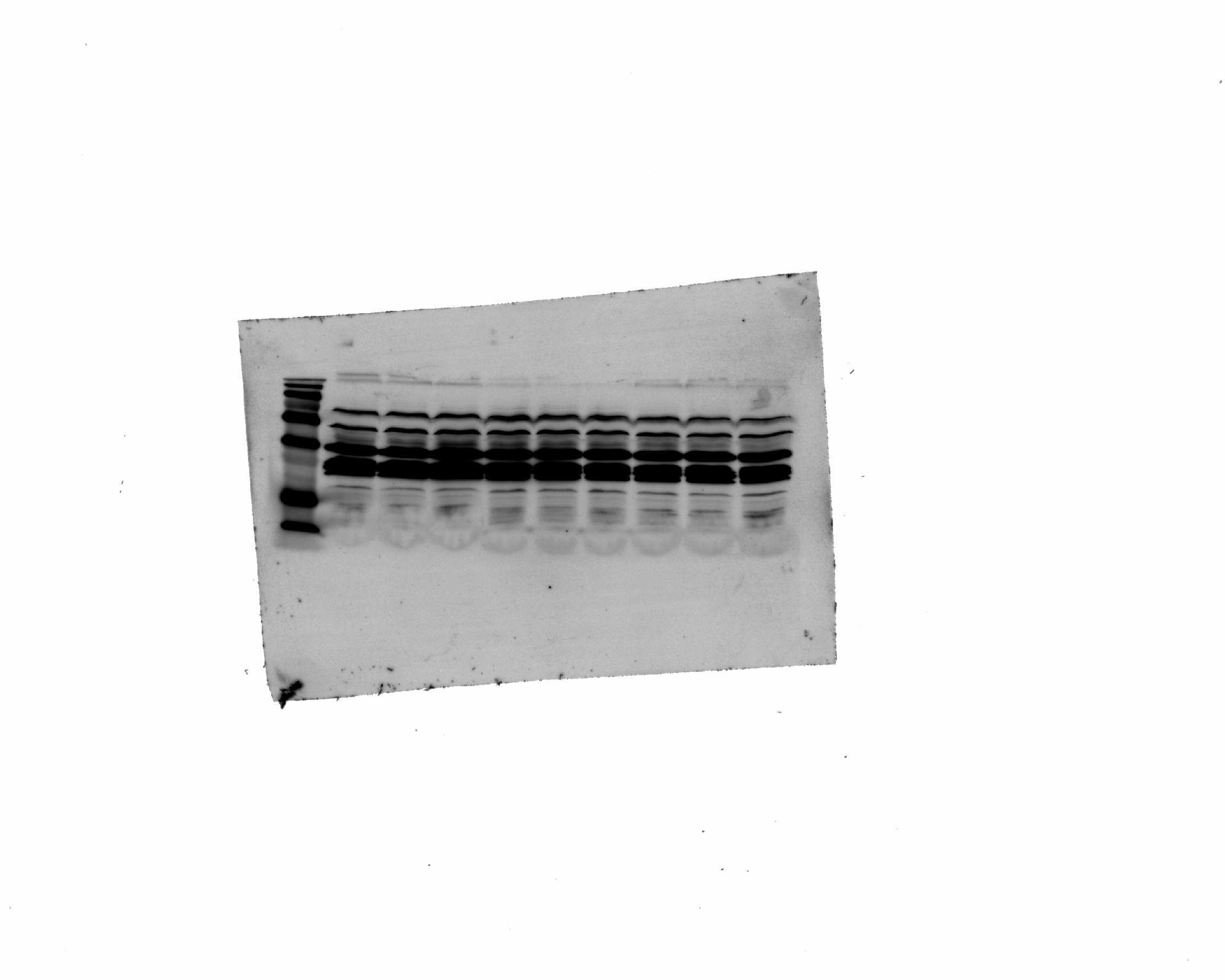

Supplement: Supplementary file 13 — Raw Western Blot and Microscopy Images [file 44318_2026_809_MOESM13_ESM.zip › SD_Blots/SD S2C/S2C AICD.tif]

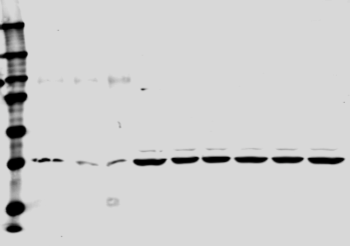

Supplement: Supplementary file 13 — Raw Western Blot and Microscopy Images [file 44318_2026_809_MOESM13_ESM.zip › SD_Blots/SD S2C/S2C APH-1.png]

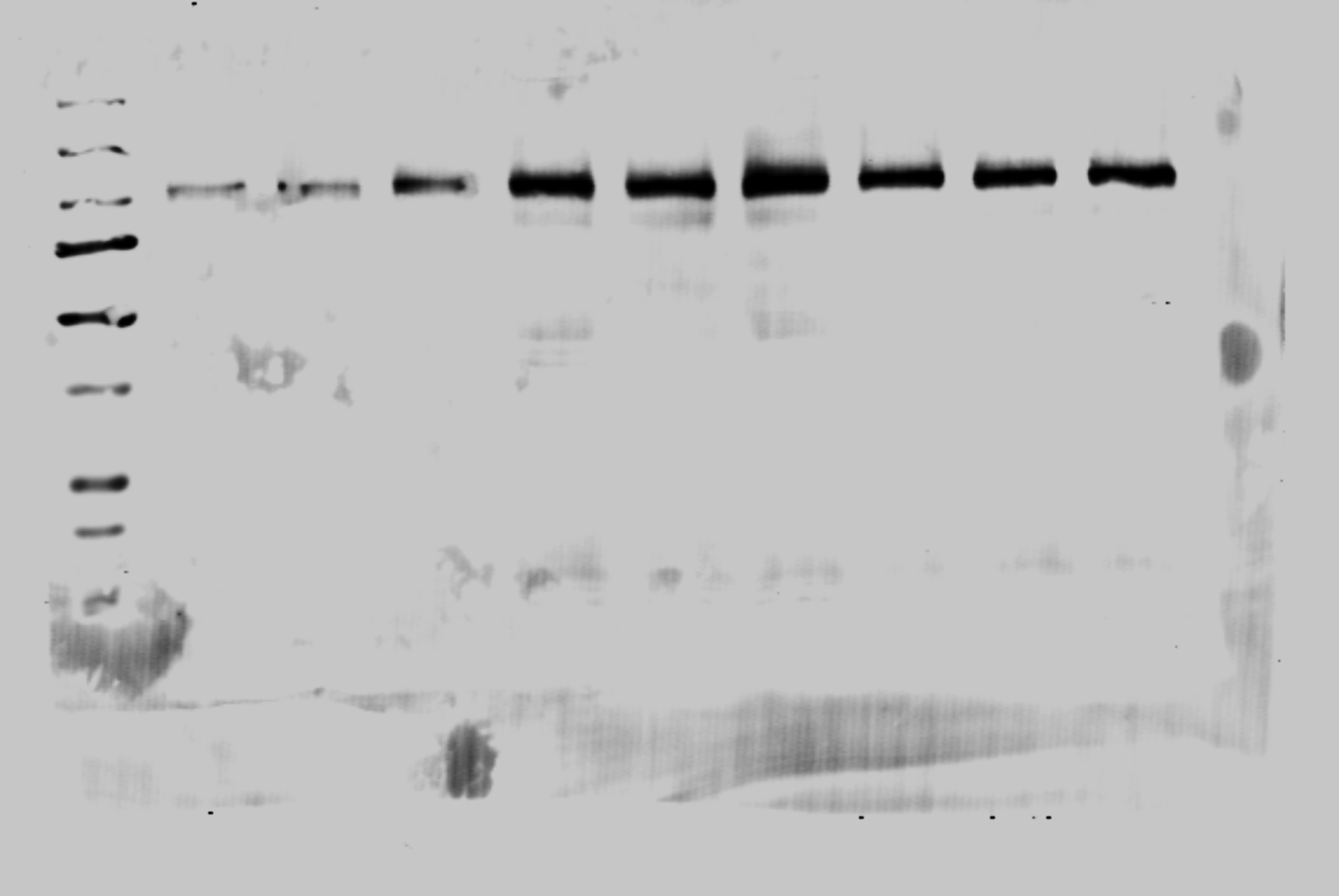

Supplement: Supplementary file 13 — Raw Western Blot and Microscopy Images [file 44318_2026_809_MOESM13_ESM.zip › SD_Blots/SD S2C/S2C APP.tif]

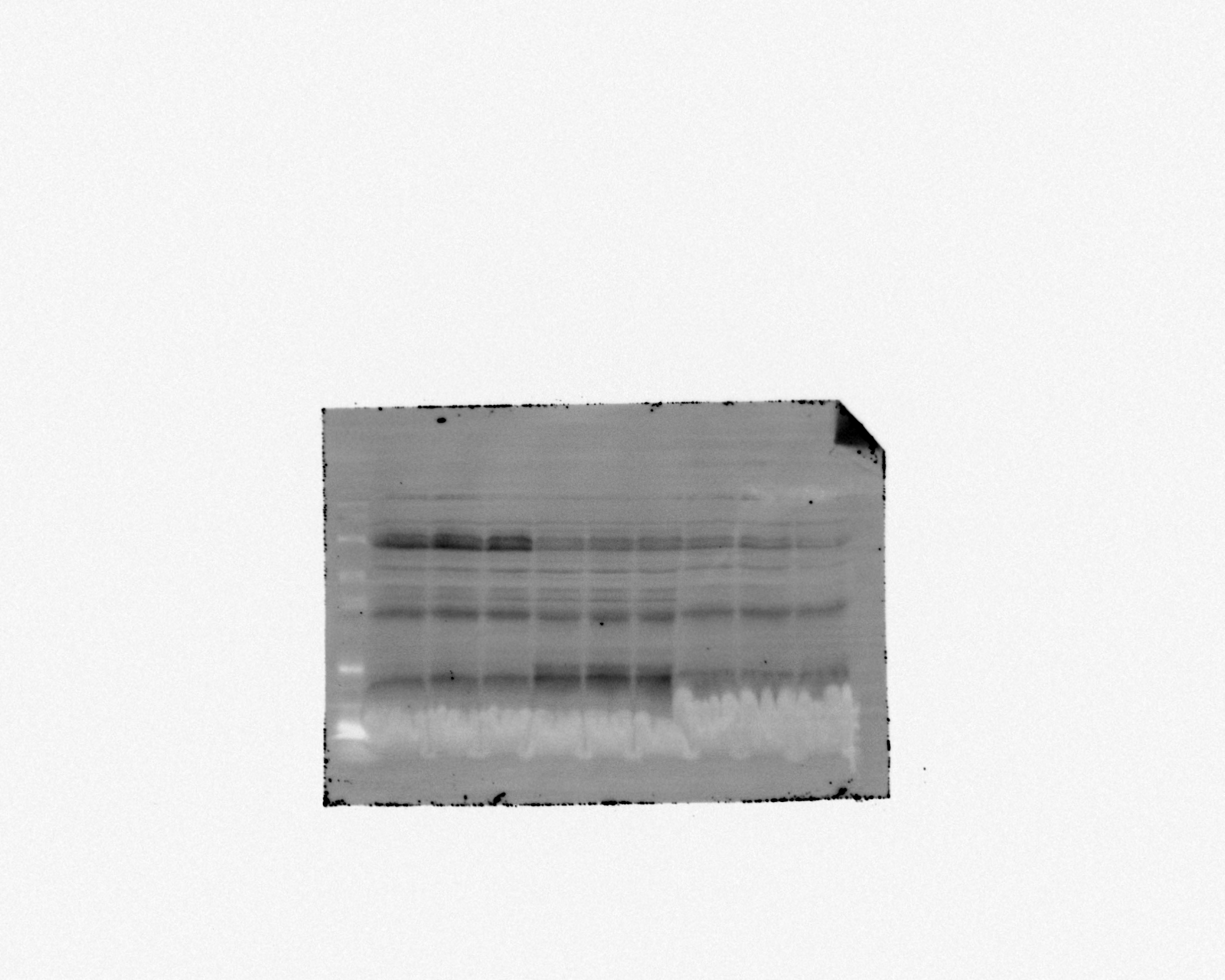

Supplement: Supplementary file 13 — Raw Western Blot and Microscopy Images [file 44318_2026_809_MOESM13_ESM.zip › SD_Blots/SD S2C/S2C C99.tif]

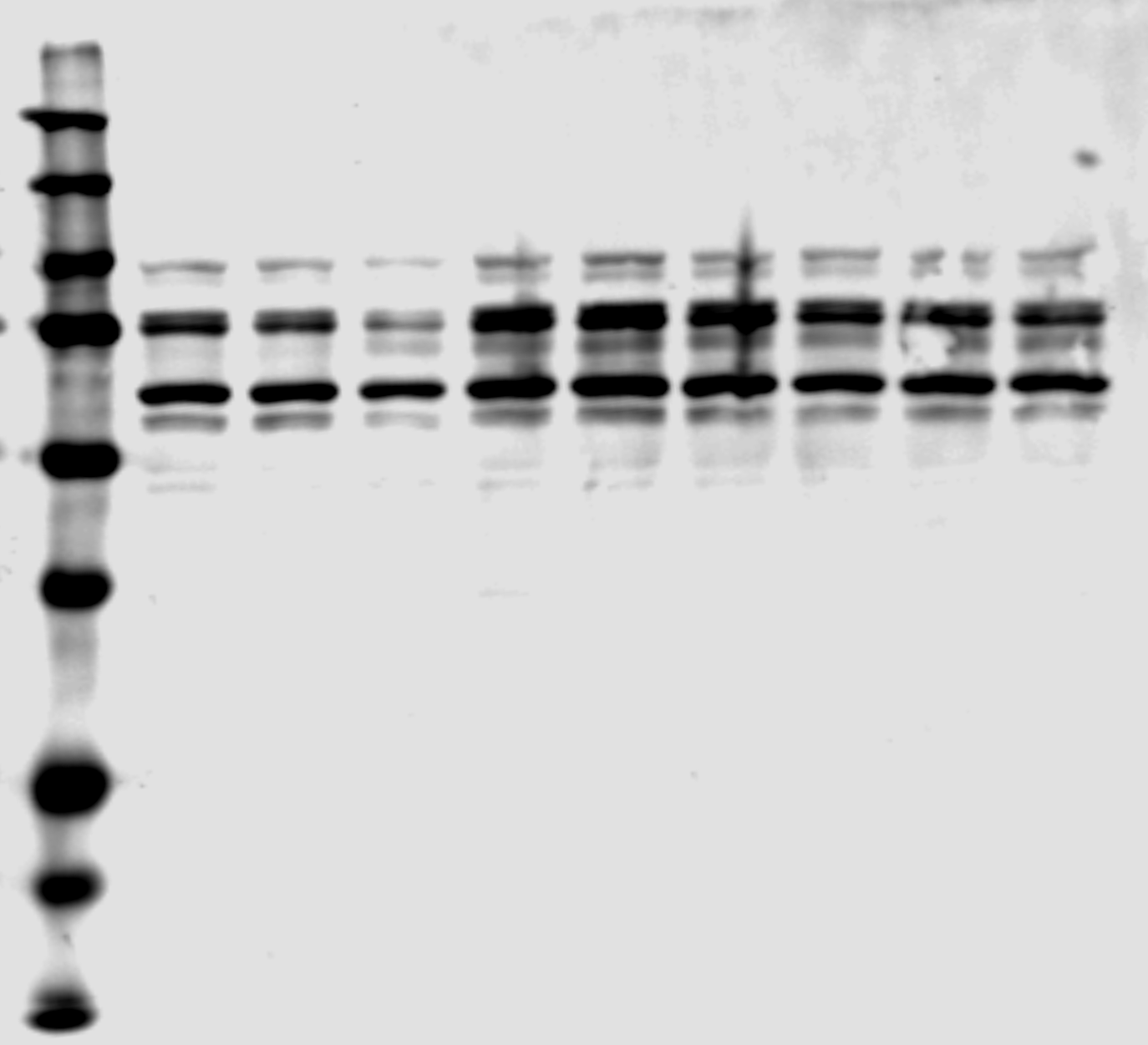

Supplement: Supplementary file 13 — Raw Western Blot and Microscopy Images [file 44318_2026_809_MOESM13_ESM.zip › SD_Blots/SD S2C/S2C NCT.tif]

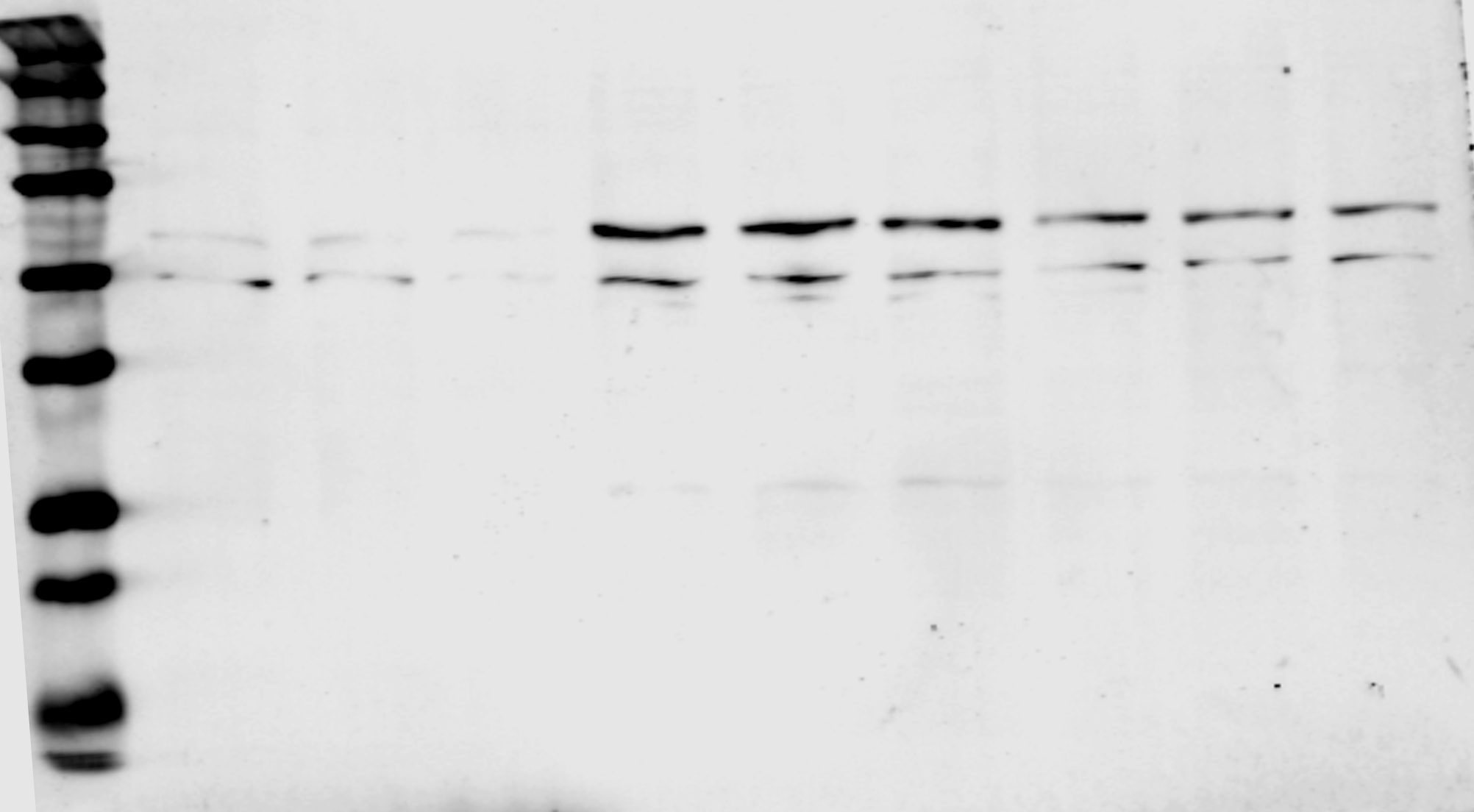

Supplement: Supplementary file 13 — Raw Western Blot and Microscopy Images [file 44318_2026_809_MOESM13_ESM.zip › SD_Blots/SD S2C/S2C PS1.tif]

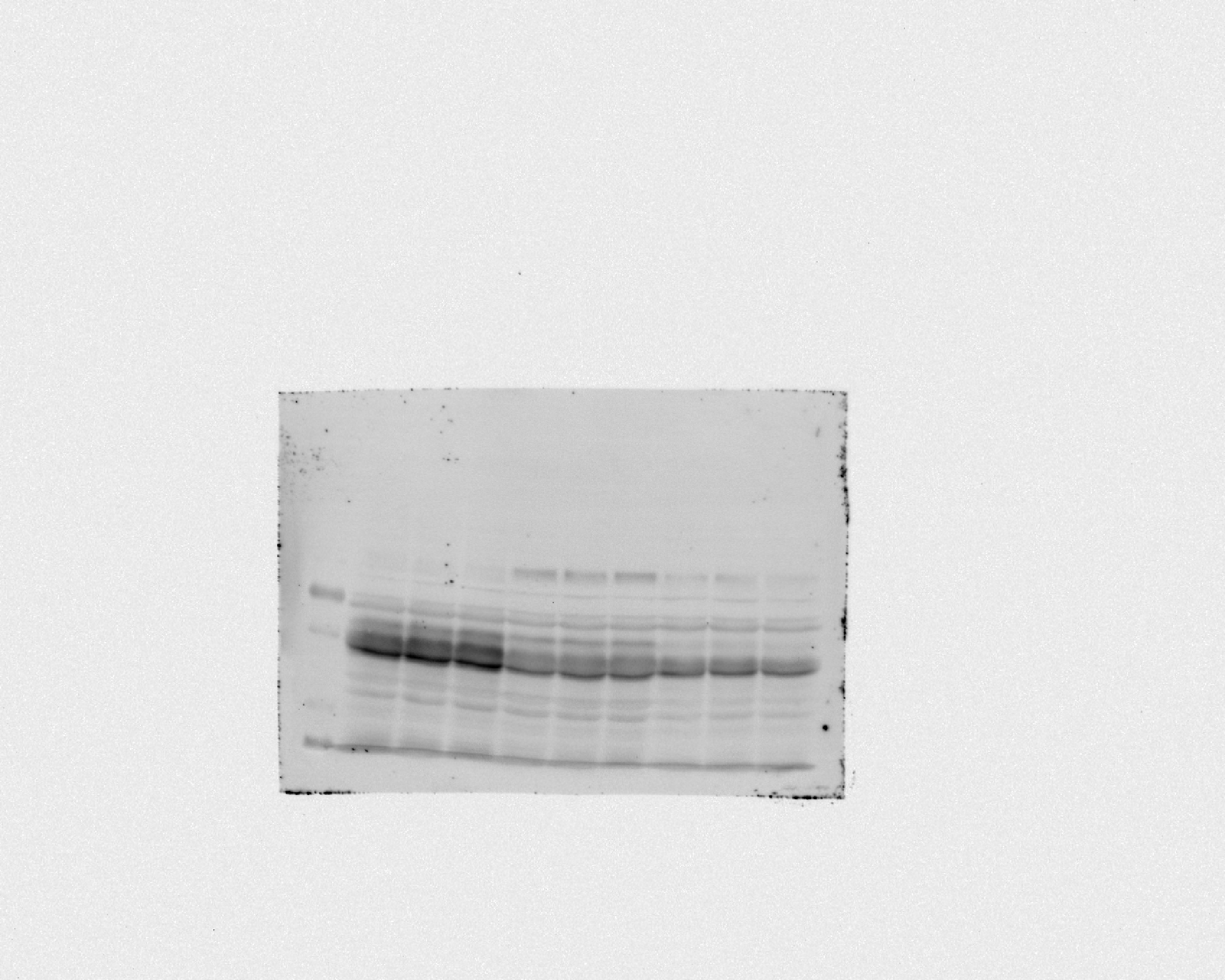

Supplement: Supplementary file 13 — Raw Western Blot and Microscopy Images [file 44318_2026_809_MOESM13_ESM.zip › SD_Blots/SD S2C/S2C sAPPa.tif]

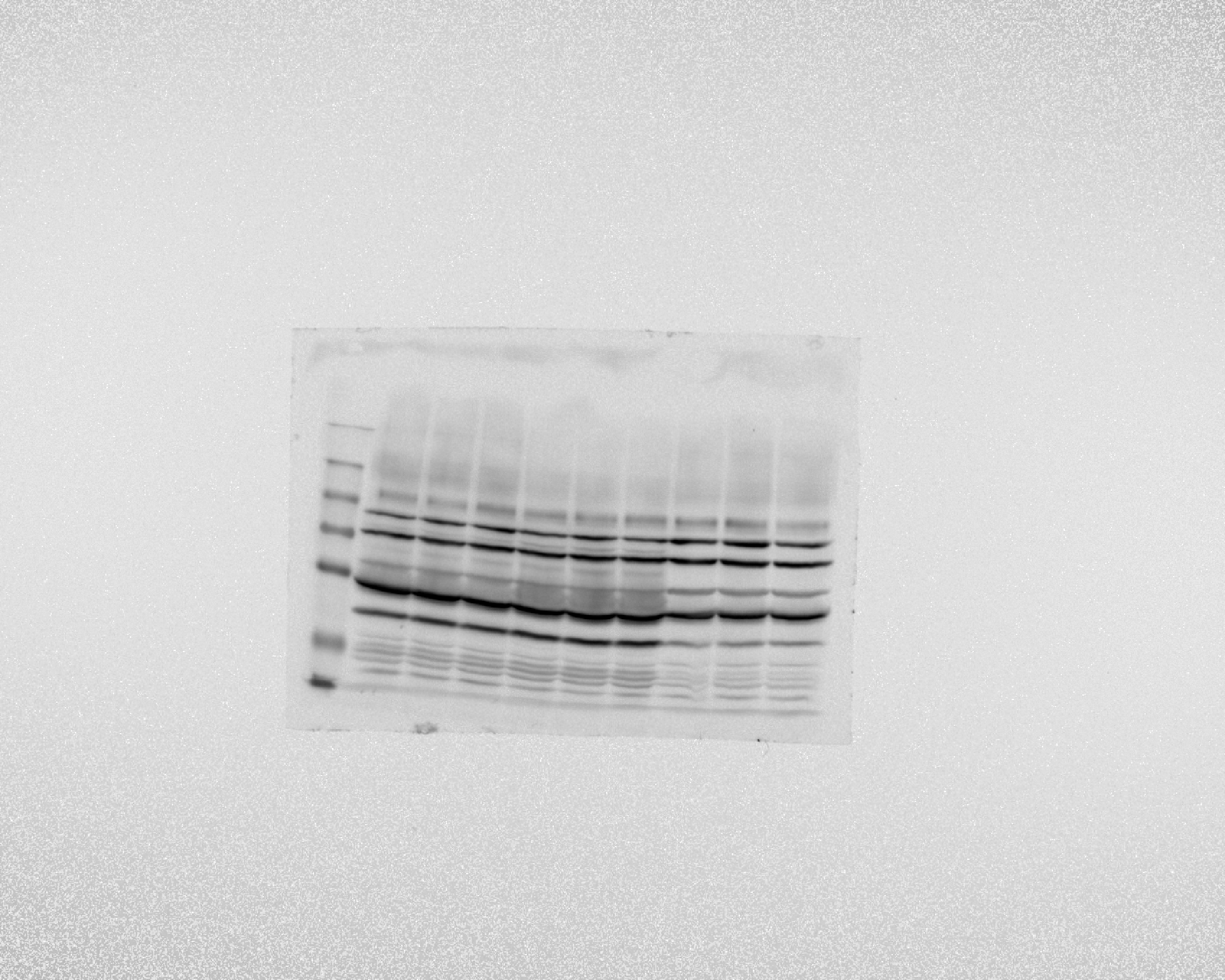

Supplement: Supplementary file 13 — Raw Western Blot and Microscopy Images [file 44318_2026_809_MOESM13_ESM.zip › SD_Blots/SD S2C/S2C sAPPb.tif]

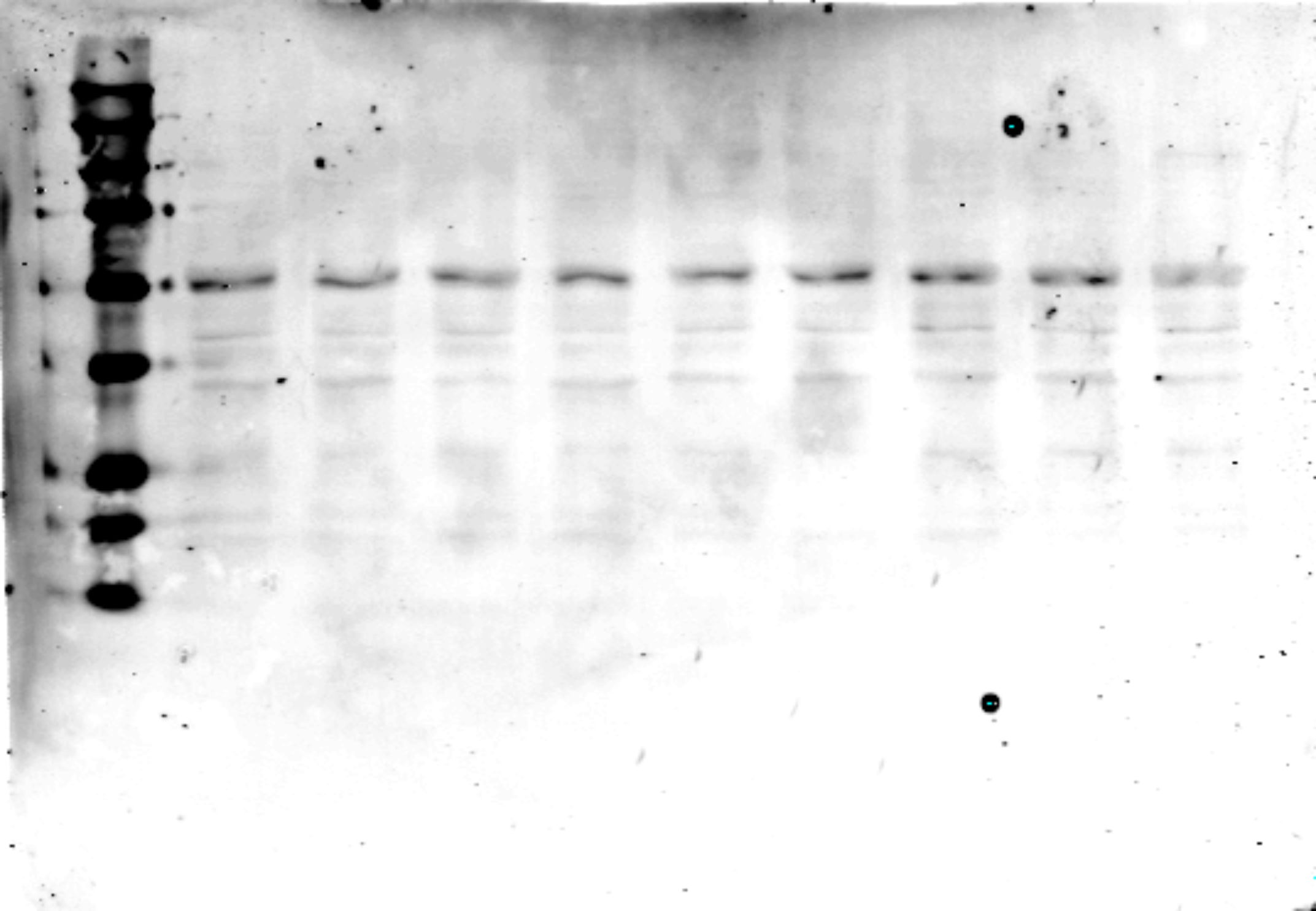

Supplement: Supplementary file 13 — Raw Western Blot and Microscopy Images [file 44318_2026_809_MOESM13_ESM.zip › SD_Blots/SD S2C/S2C TUBULIN.tif]

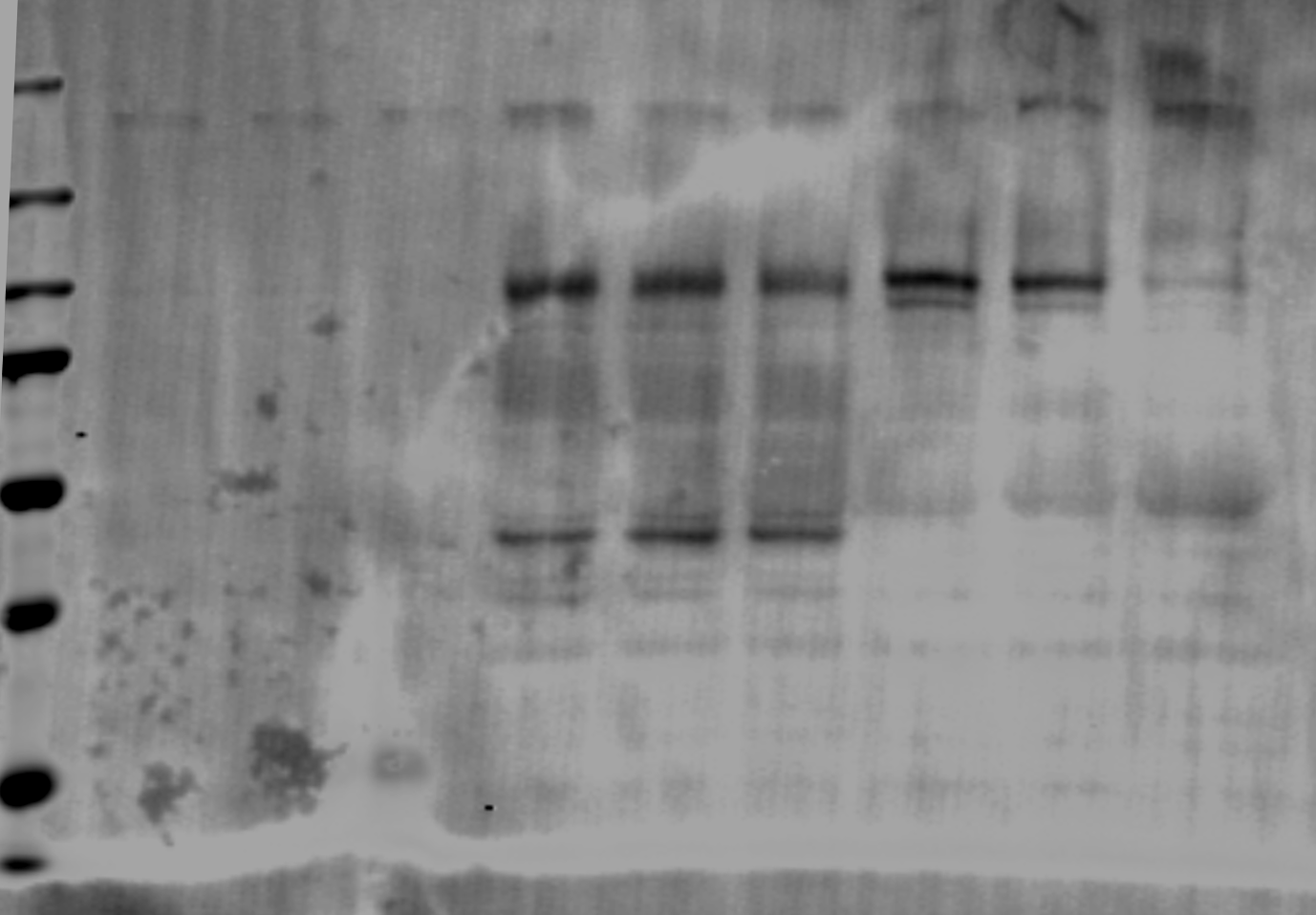

Supplement: Supplementary file 13 — Raw Western Blot and Microscopy Images [file 44318_2026_809_MOESM13_ESM.zip › SD_Blots/SD S3A/S3A AT180.tif]

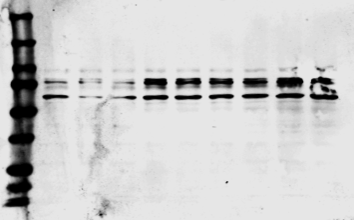

Supplement: Supplementary file 13 — Raw Western Blot and Microscopy Images [file 44318_2026_809_MOESM13_ESM.zip › SD_Blots/SD S3A/S3A AT270.tif]

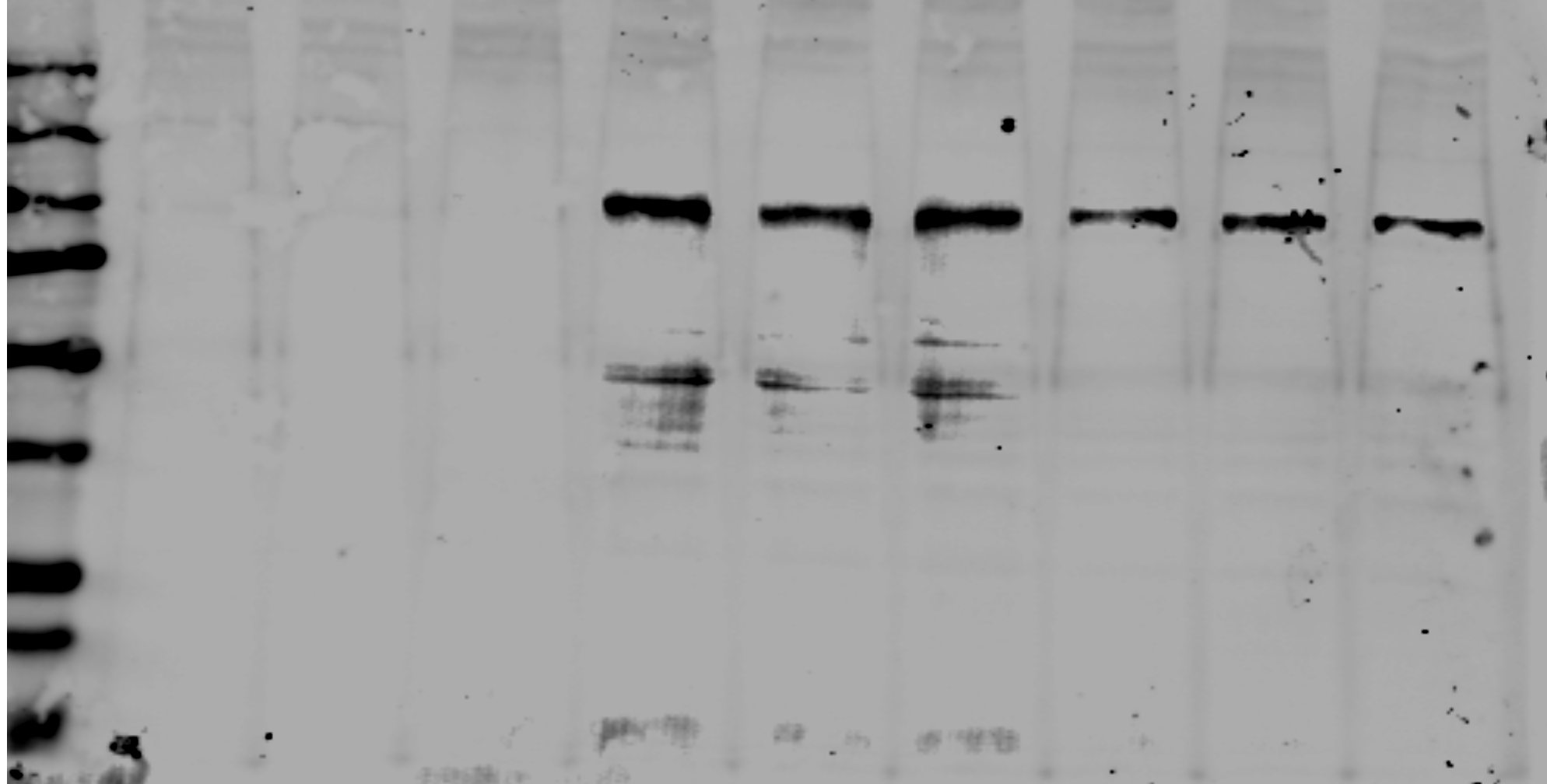

Supplement: Supplementary file 13 — Raw Western Blot and Microscopy Images [file 44318_2026_809_MOESM13_ESM.zip › SD_Blots/SD S3A/S3A AT8.tif]

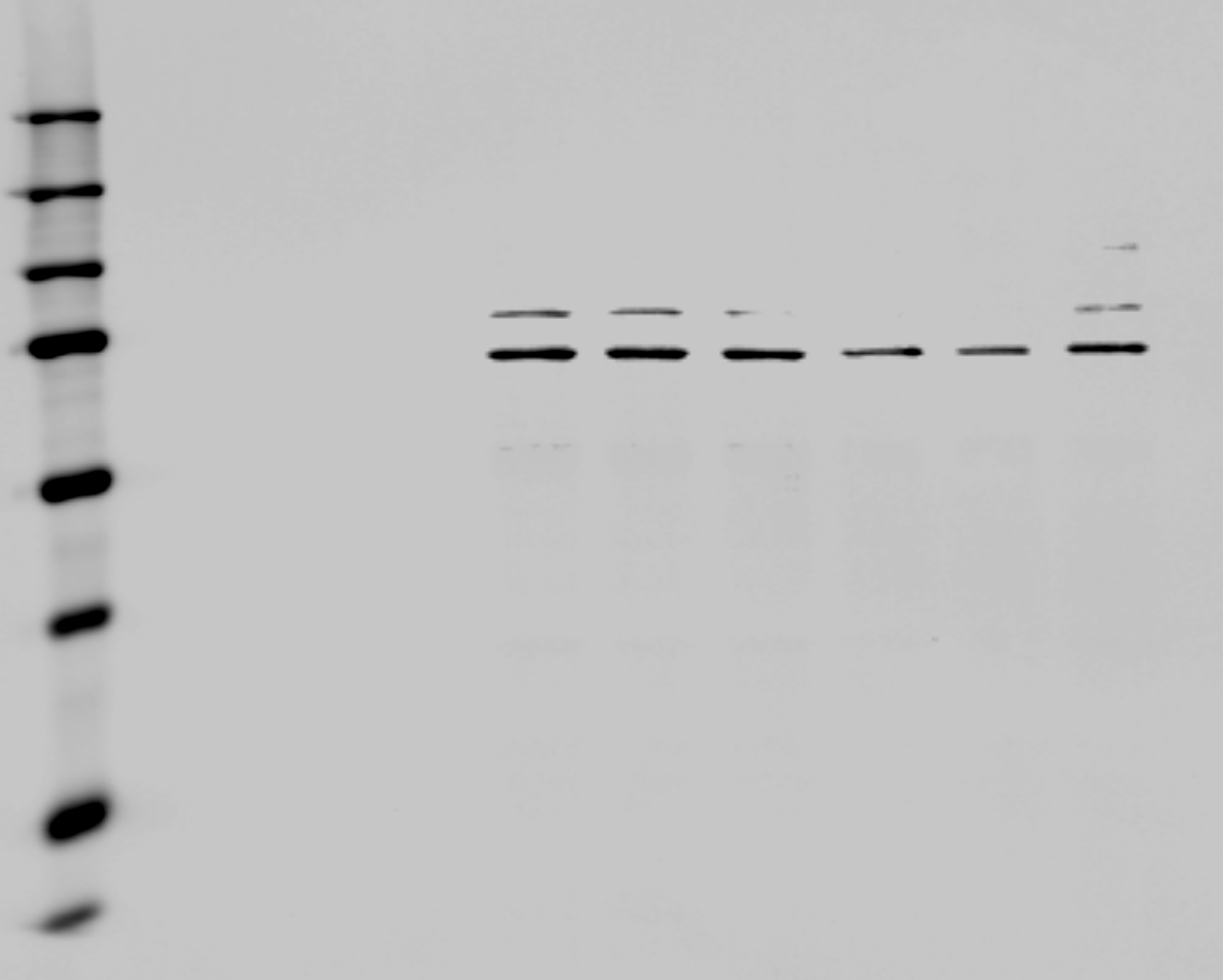

Supplement: Supplementary file 13 — Raw Western Blot and Microscopy Images [file 44318_2026_809_MOESM13_ESM.zip › SD_Blots/SD S3A/S3A HT7 Insoluble.tif]

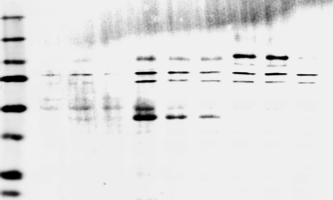

Supplement: Supplementary file 13 — Raw Western Blot and Microscopy Images [file 44318_2026_809_MOESM13_ESM.zip › SD_Blots/SD S3A/S3A HT7 SOLUBLE.tif]

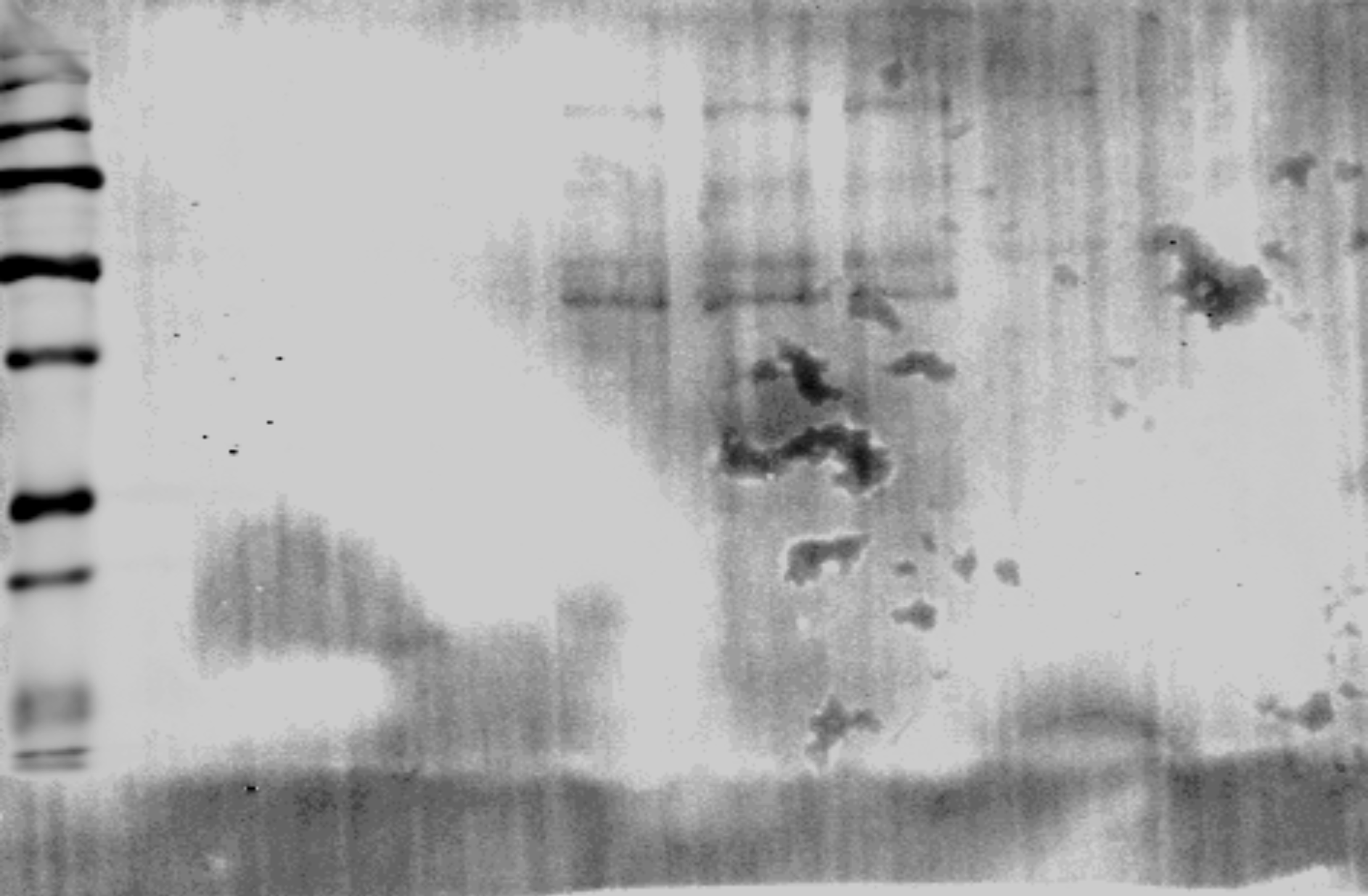

Supplement: Supplementary file 13 — Raw Western Blot and Microscopy Images [file 44318_2026_809_MOESM13_ESM.zip › SD_Blots/SD S3A/S3A PHF13.tif]

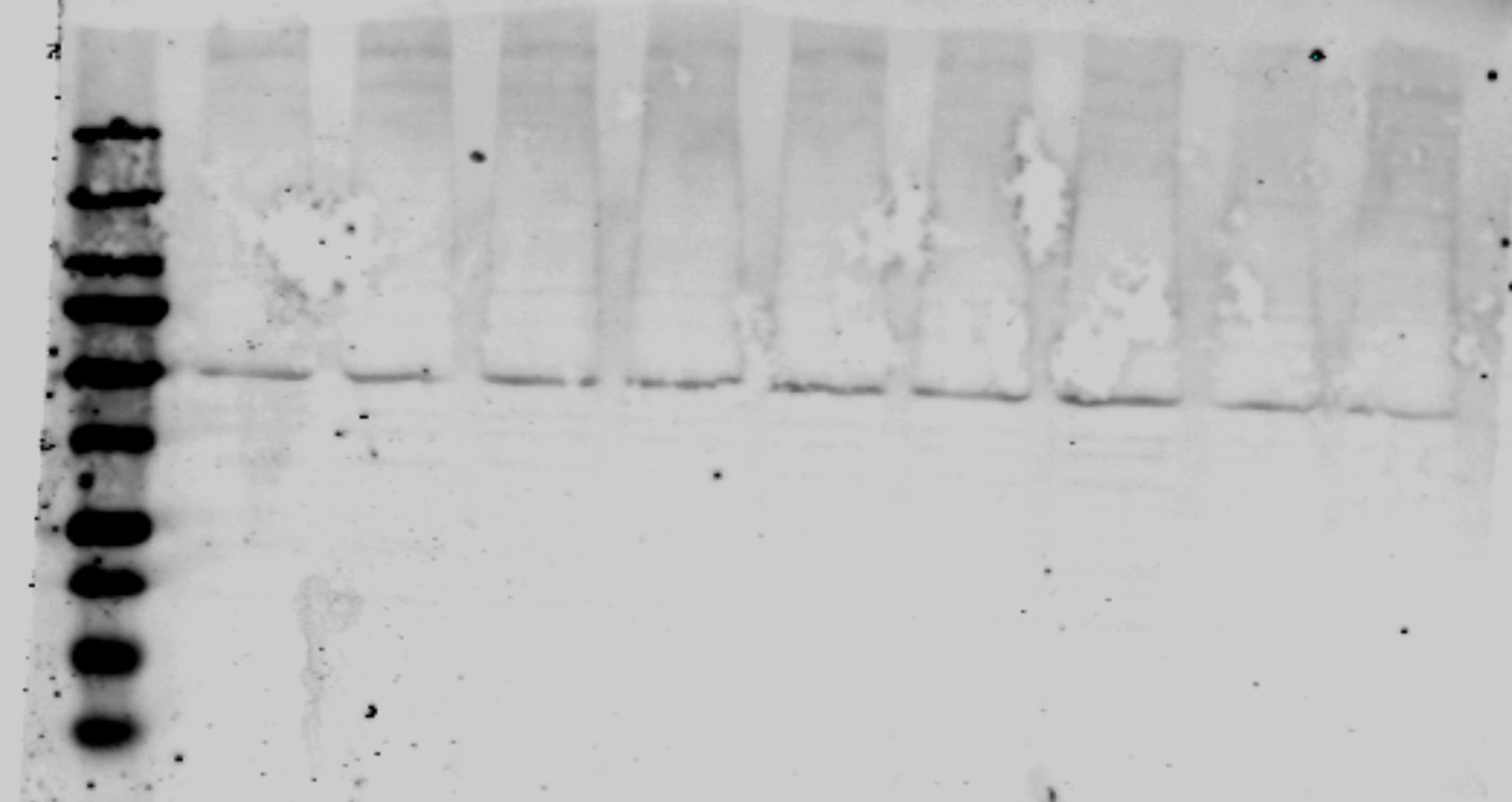

Supplement: Supplementary file 13 — Raw Western Blot and Microscopy Images [file 44318_2026_809_MOESM13_ESM.zip › SD_Blots/SD S3A/S3A Tubulin2.tif]

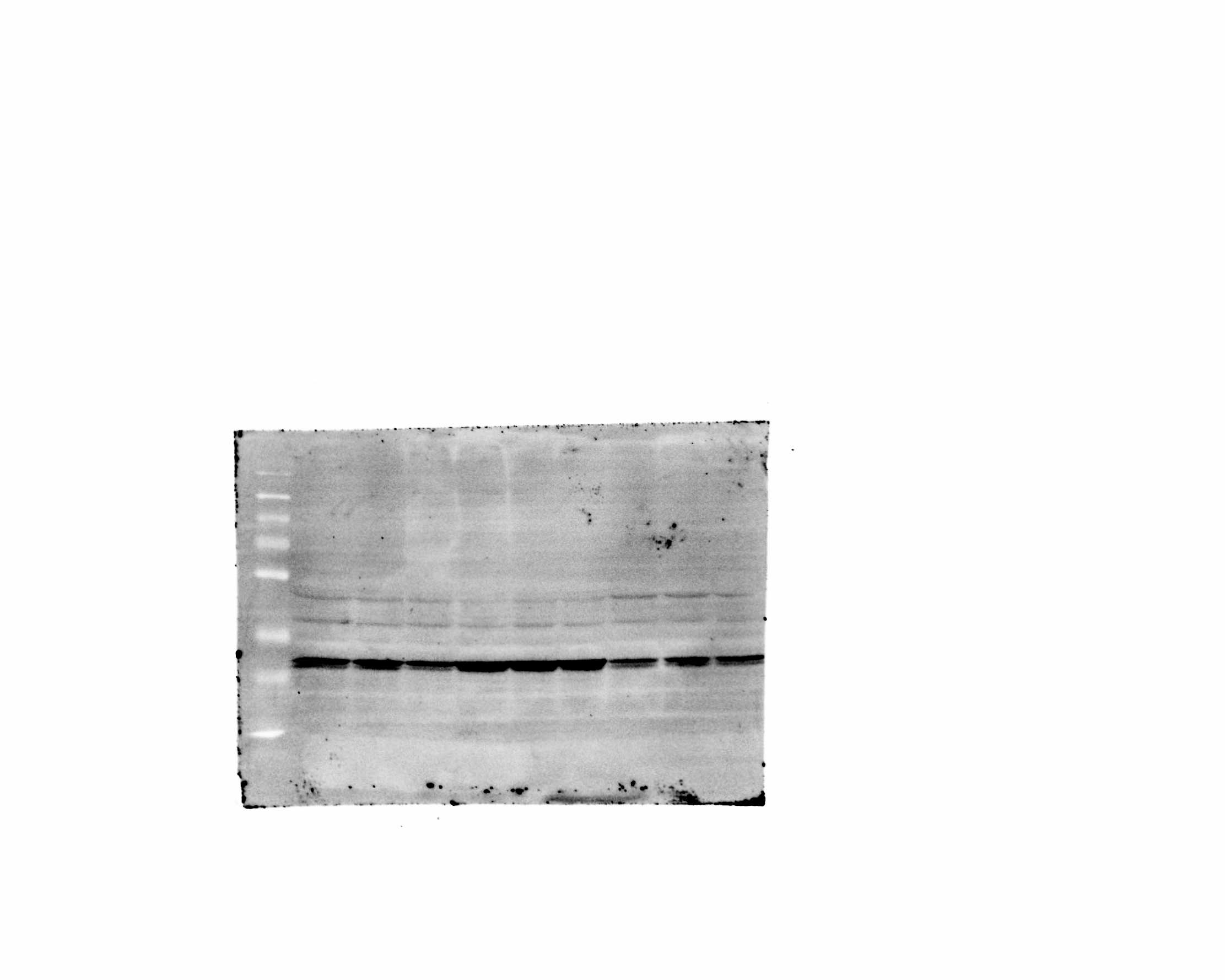

Supplement: Supplementary file 13 — Raw Western Blot and Microscopy Images [file 44318_2026_809_MOESM13_ESM.zip › SD_Blots/SD S3E/3E CamkIIa.tif]

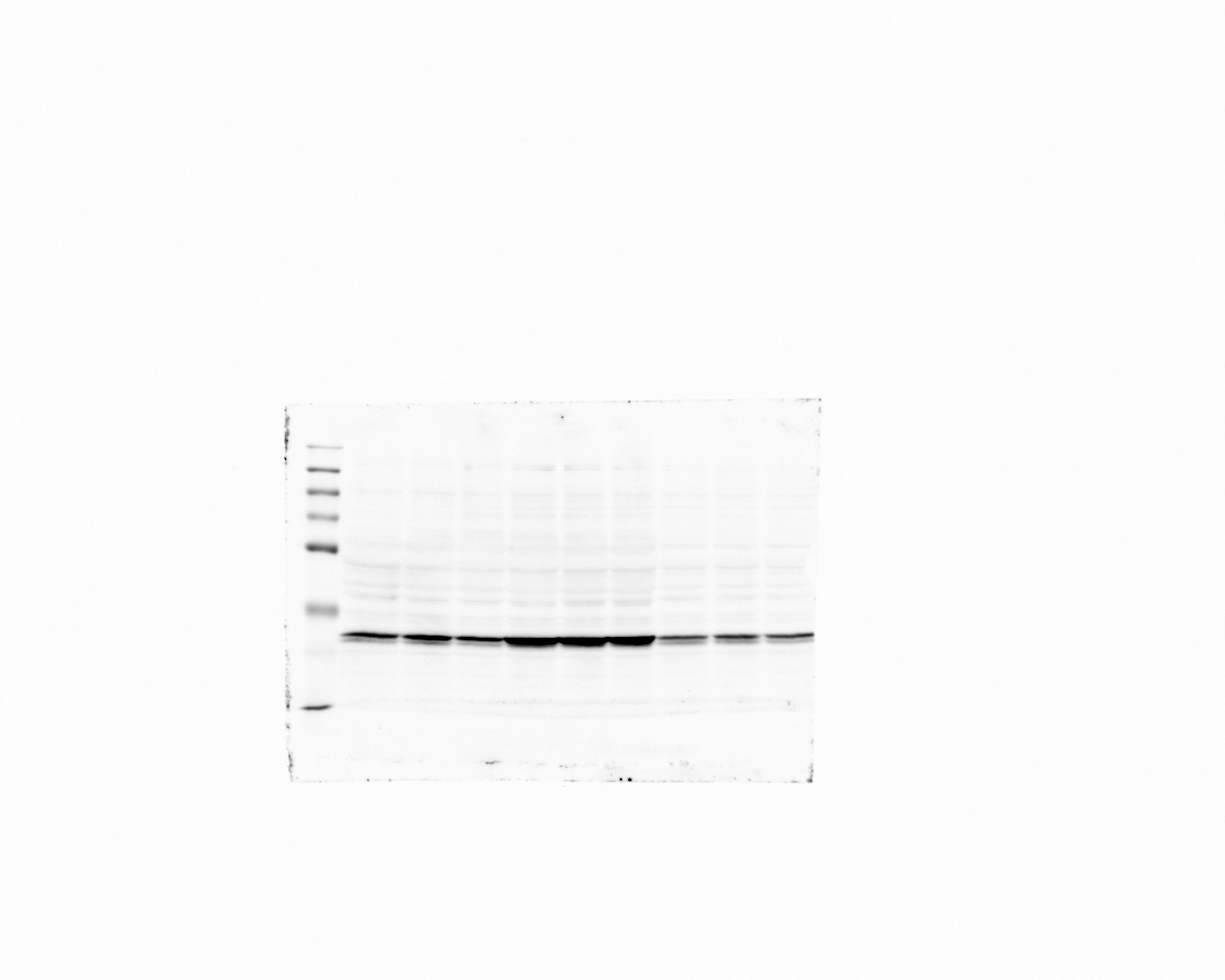

Supplement: Supplementary file 13 — Raw Western Blot and Microscopy Images [file 44318_2026_809_MOESM13_ESM.zip › SD_Blots/SD S3E/3E CDK5.tif]

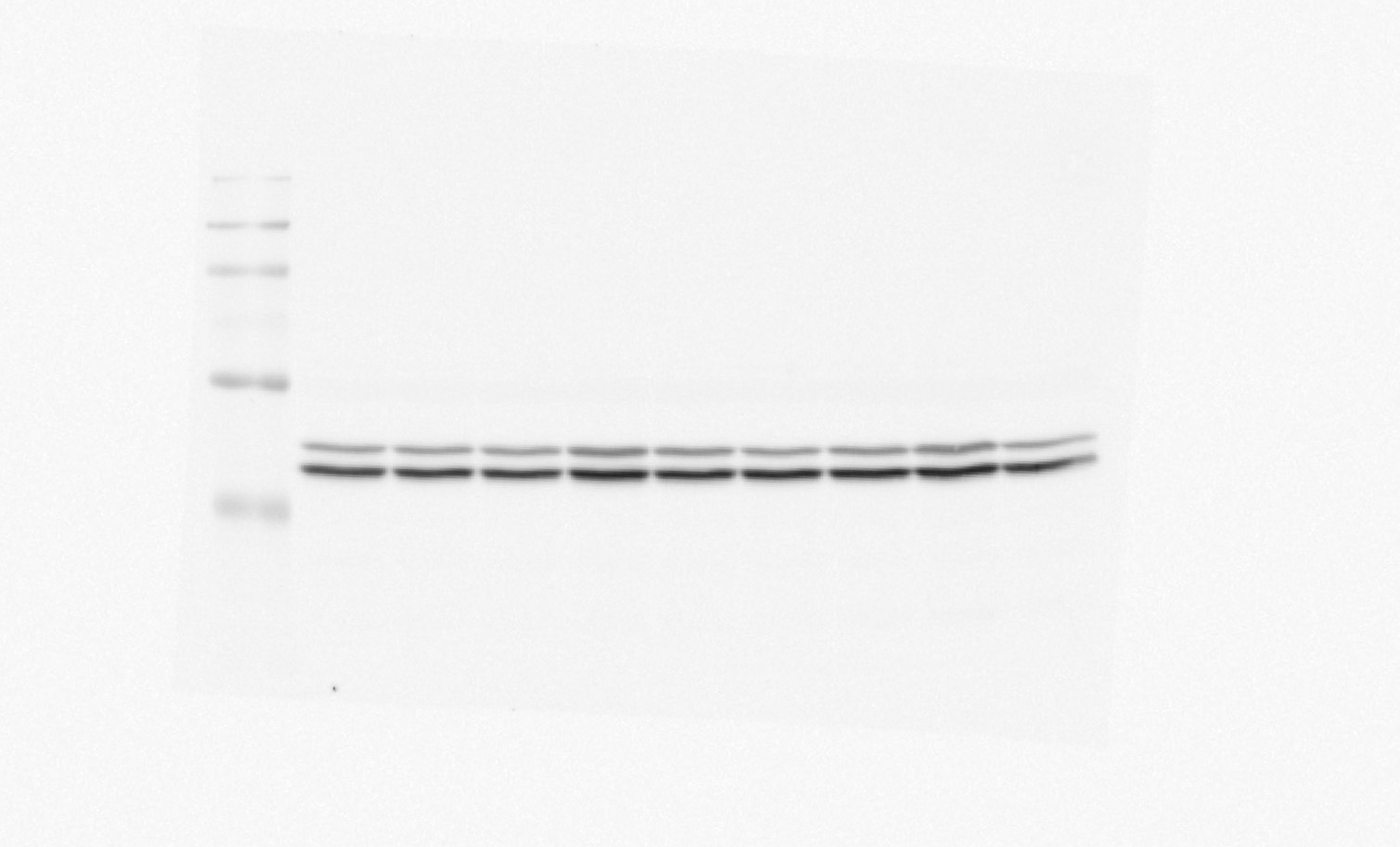

Supplement: Supplementary file 13 — Raw Western Blot and Microscopy Images [file 44318_2026_809_MOESM13_ESM.zip › SD_Blots/SD S3E/3E ERK2.jpg]
