# Supplementary material for: Genetic ablation of neuronal mitochondrial calcium uptake impedes Alzheimer’s disease progression
Source: EMBO J. 2026 May 22;45(13):4469–91. doi: 10.1038/s44318-026-00809-w (PMC13324160; doi:10.1038/s44318-026-00809-w)
Supplement: Supplementary file 9 — Figure EV2 Source Data [file 44318_2026_809_MOESM9_ESM.zip › Source data for Figure EV2/EV2C.pptx]

## Slide 1
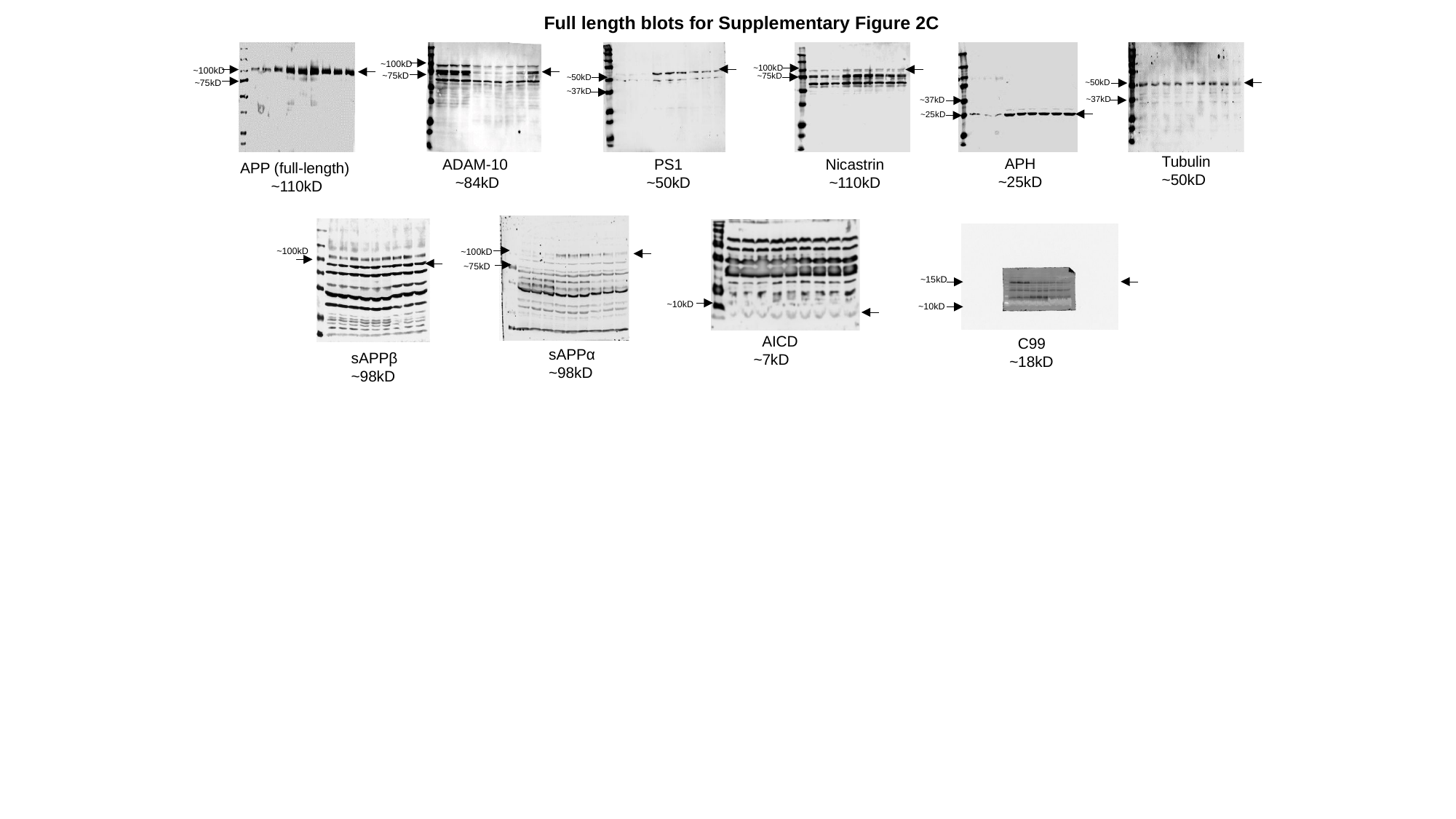

Full length blots for Supplementary Figure 2C
~100kD
~75kD
~100kD
~75kD
~100kD
~75kD
~50kD
~37kD
~50kD
~37kD
~37kD
~25kD
Tubulin
~50kD
APH
~25kD
Nicastrin
~110kD
ADAM-10
~84kD
PS1
~50kD
APP (full-length)
~110kD
~15kD
~10kD
~100kD
~100kD
~75kD
~10kD
 AICD
~7kD
 C99
~18kD
sAPPα
~98kD
sAPPβ
~98kD
