# Supplementary material for: Genetic ablation of neuronal mitochondrial calcium uptake impedes Alzheimer’s disease progression
Source: EMBO J. 2026 May 22;45(13):4469–91. doi: 10.1038/s44318-026-00809-w (PMC13324160; doi:10.1038/s44318-026-00809-w)
Supplement: Supplementary file 10 — Figure EV3 Source Data [file 44318_2026_809_MOESM10_ESM.zip › Source data for Figure EV3/EV3A.pptx]

## Slide 1
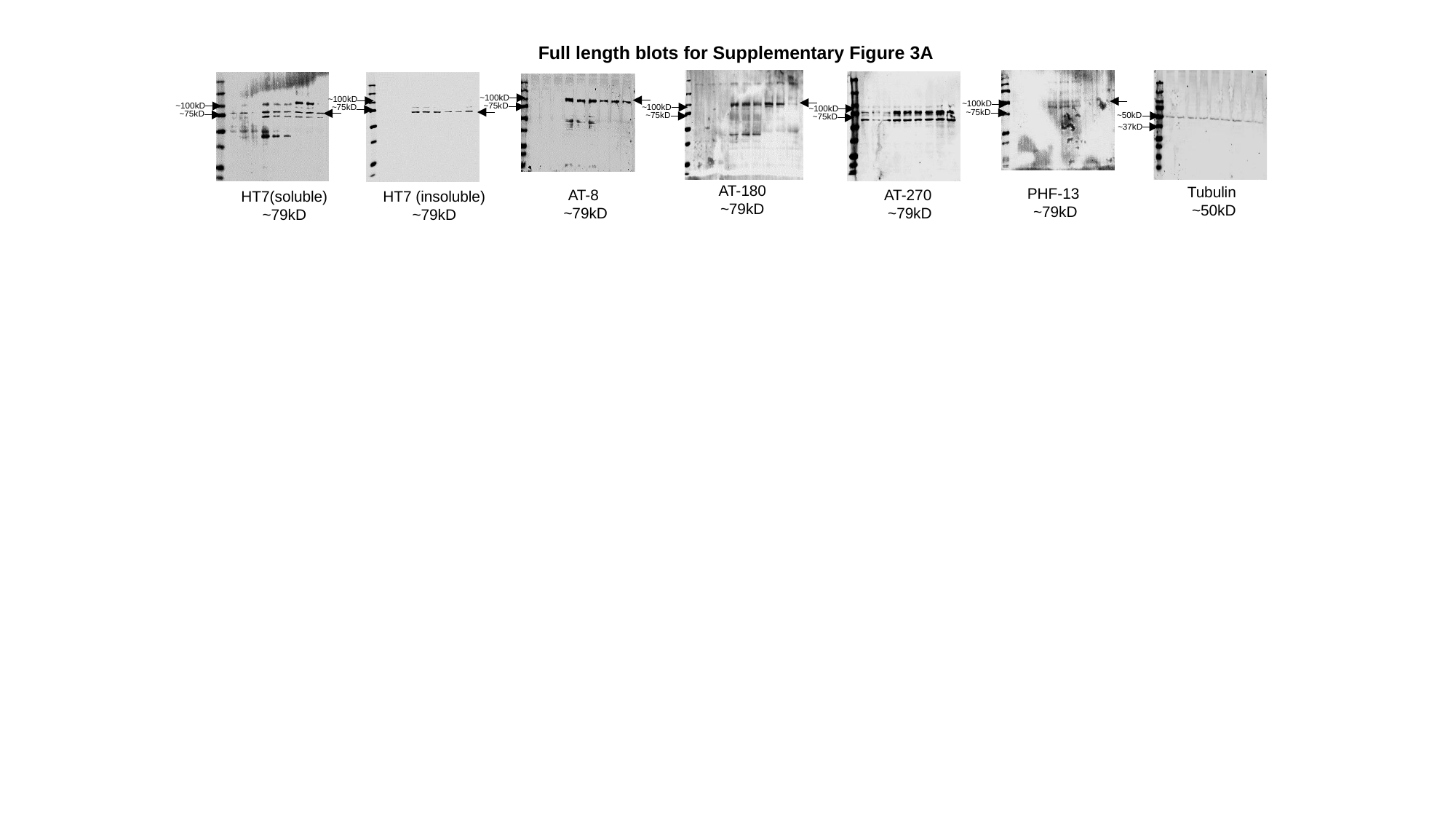

Full length blots for Supplementary Figure 3A
PHF-13
~79kD
~100kD
~75kD
~100kD
~75kD
~100kD
~75kD
~100kD
~75kD
~100kD
~75kD
~100kD
~75kD
~50kD
~37kD
AT-180
~79kD
Tubulin
~50kD
AT-8
~79kD
AT-270
~79kD
HT7 (insoluble) ~79kD
HT7(soluble) ~79kD
