# Supplementary material for: Genetic ablation of neuronal mitochondrial calcium uptake impedes Alzheimer’s disease progression
Source: EMBO J. 2026 May 22;45(13):4469–91. doi: 10.1038/s44318-026-00809-w (PMC13324160; doi:10.1038/s44318-026-00809-w)
Supplement: Supplementary file 10 — Figure EV3 Source Data [file 44318_2026_809_MOESM10_ESM.zip › Source data for Figure EV3/EV3E.pptx]

## Slide 1
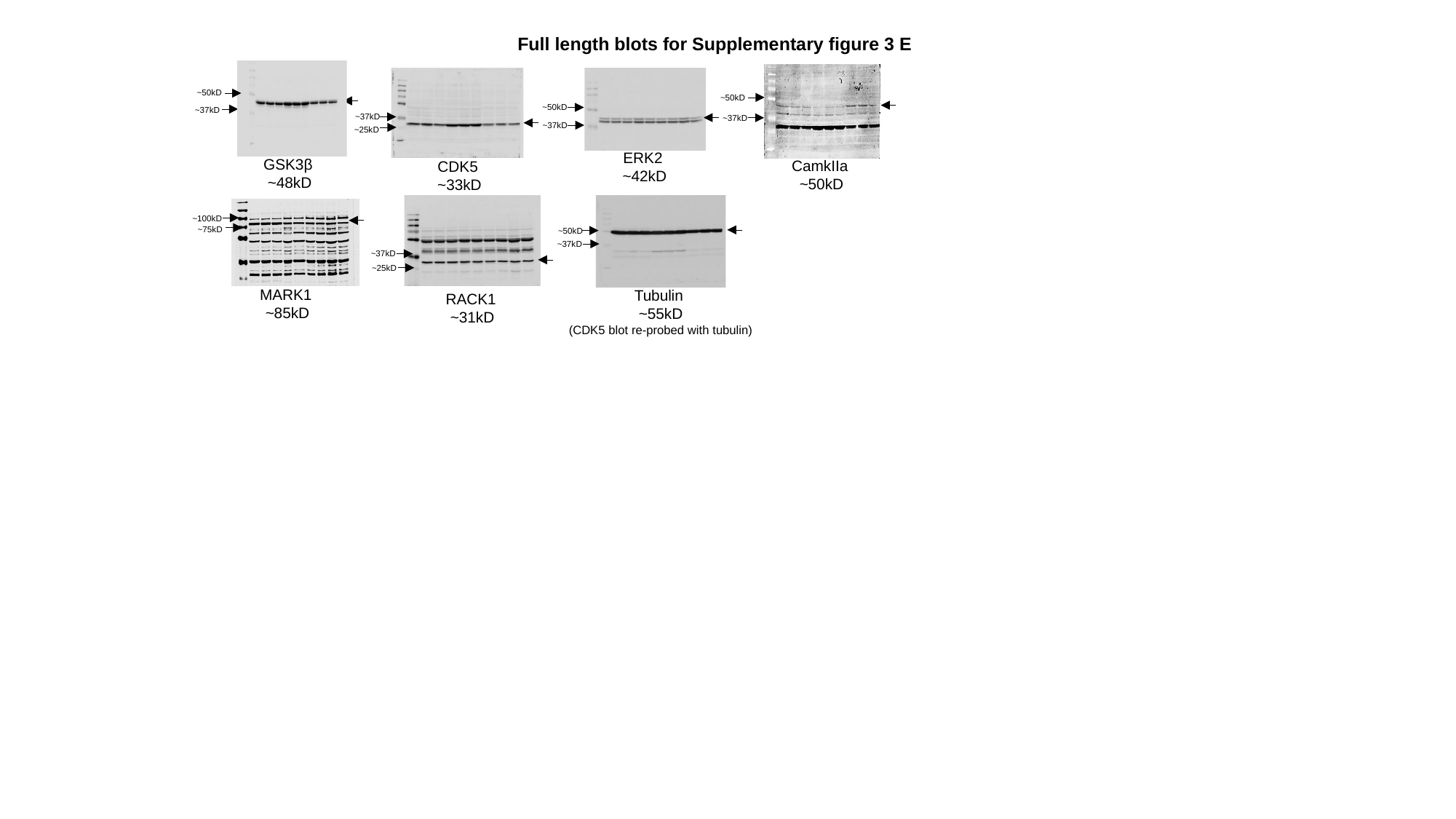

Full length blots for Supplementary figure 3 E
CDK5
~33kD
~50kD
~37kD
GSK3β
~48kD
~50kD
~37kD
~50kD
~37kD
~37kD
~25kD
ERK2
~42kD
CamkIIa
~50kD
~100kD
~75kD
~50kD
~37kD
~37kD
~25kD
MARK1
~85kD
Tubulin
~55kD
(CDK5 blot re-probed with tubulin)
RACK1
~31kD
