# Supplementary material for: Genetic ablation of neuronal mitochondrial calcium uptake impedes Alzheimer’s disease progression
Source: EMBO J. 2026 May 22;45(13):4469–91. doi: 10.1038/s44318-026-00809-w (PMC13324160; doi:10.1038/s44318-026-00809-w)
Supplement: Supplementary file 10 — Figure EV3 Source Data [file 44318_2026_809_MOESM10_ESM.zip › Source data for Figure EV3/EV3L.pptx]

## Slide 1
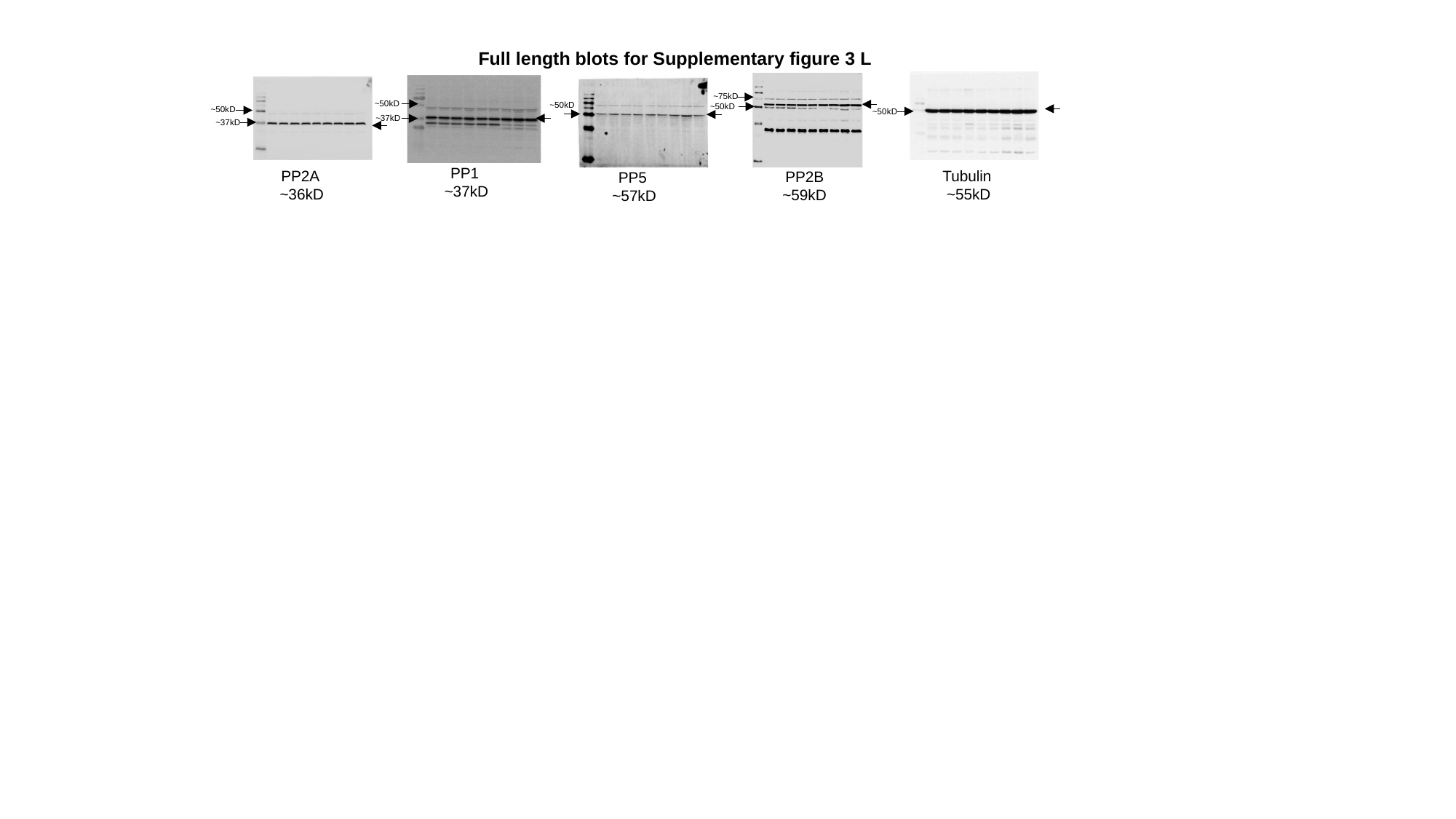

Full length blots for Supplementary figure 3 L
~75kD
~50kD
~50kD
~50kD
~50kD
~50kD
~37kD
~37kD
PP1
~37kD
PP2A
~36kD
Tubulin
~55kD
PP2B
~59kD
PP5
~57kD
