# Supplementary material for: Genetic ablation of neuronal mitochondrial calcium uptake impedes Alzheimer’s disease progression
Source: EMBO J. 2026 May 22;45(13):4469–91. doi: 10.1038/s44318-026-00809-w (PMC13324160; doi:10.1038/s44318-026-00809-w)
Supplement: Supplementary file 12 — Figure EV5 Source Data [file 44318_2026_809_MOESM12_ESM.zip › Source data for Figure EV5/EV5P.pptx]

## Slide 1
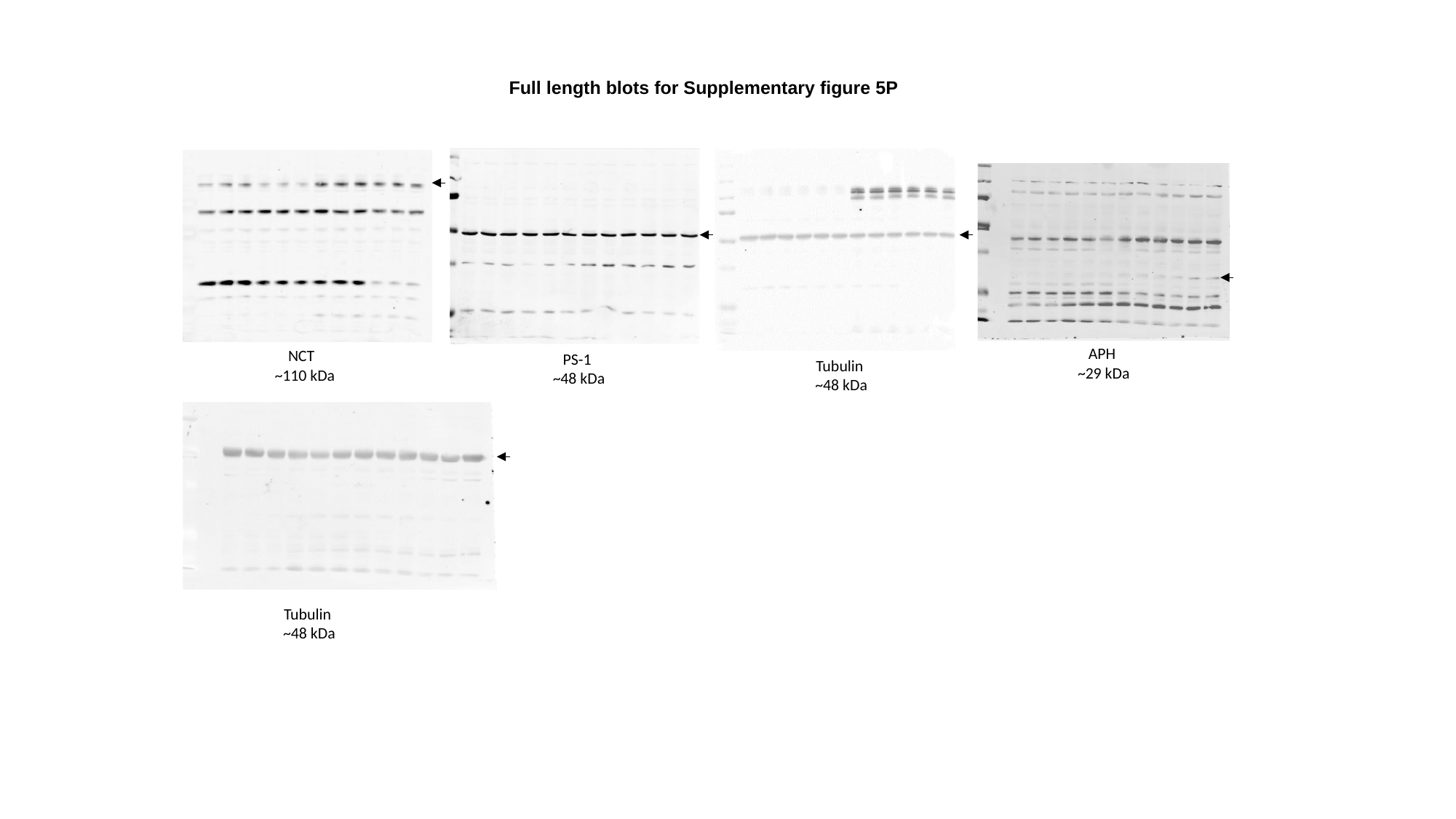

Full length blots for Supplementary figure 5P
APH
 ~29 kDa
NCT
 ~110 kDa
PS-1
 ~48 kDa
Tubulin
 ~48 kDa
Tubulin
 ~48 kDa
