# Supplementary figures and images for: Genetic ablation of neuronal mitochondrial calcium uptake impedes Alzheimer’s disease progression (part 2 of 2)
Source: EMBO J. 2026 May 22;45(13):4469–91. doi: 10.1038/s44318-026-00809-w (PMC13324160; doi:10.1038/s44318-026-00809-w)

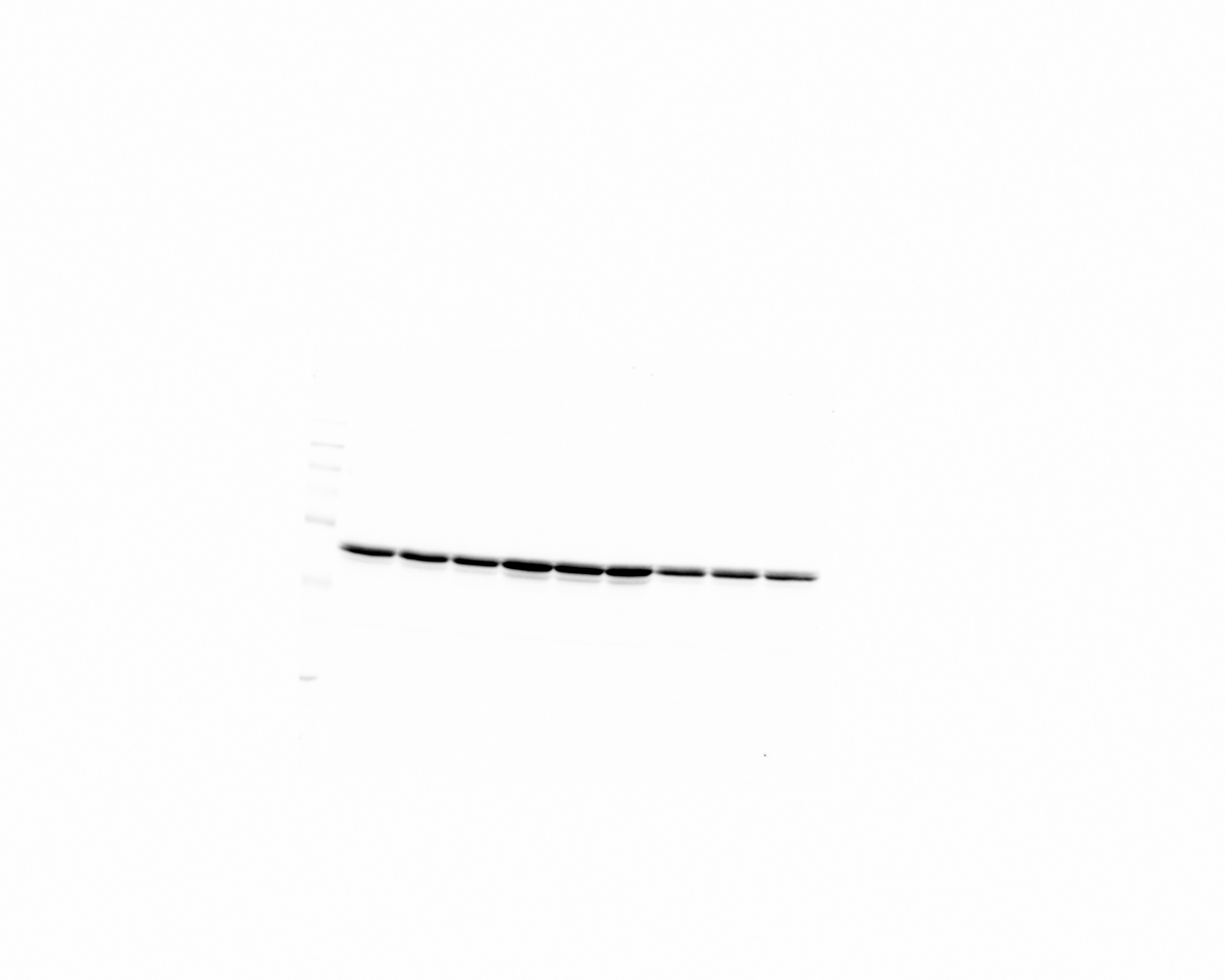

Supplement: Supplementary file 13 — Raw Western Blot and Microscopy Images [file 44318_2026_809_MOESM13_ESM.zip › SD_Blots/SD S3E/3E GSK3b.tif]

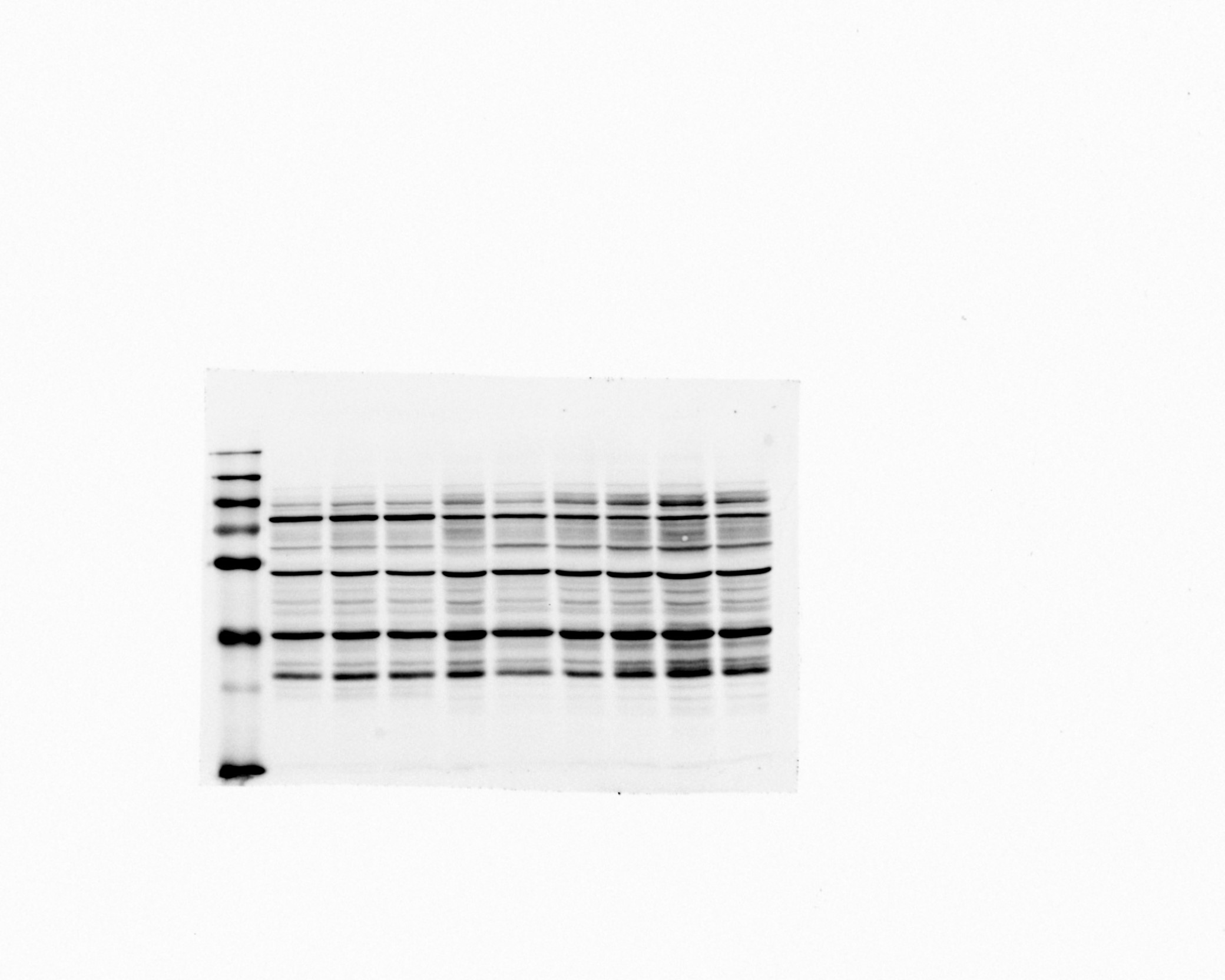

Supplement: Supplementary file 13 — Raw Western Blot and Microscopy Images [file 44318_2026_809_MOESM13_ESM.zip › SD_Blots/SD S3E/3E MARK 1.jpg]

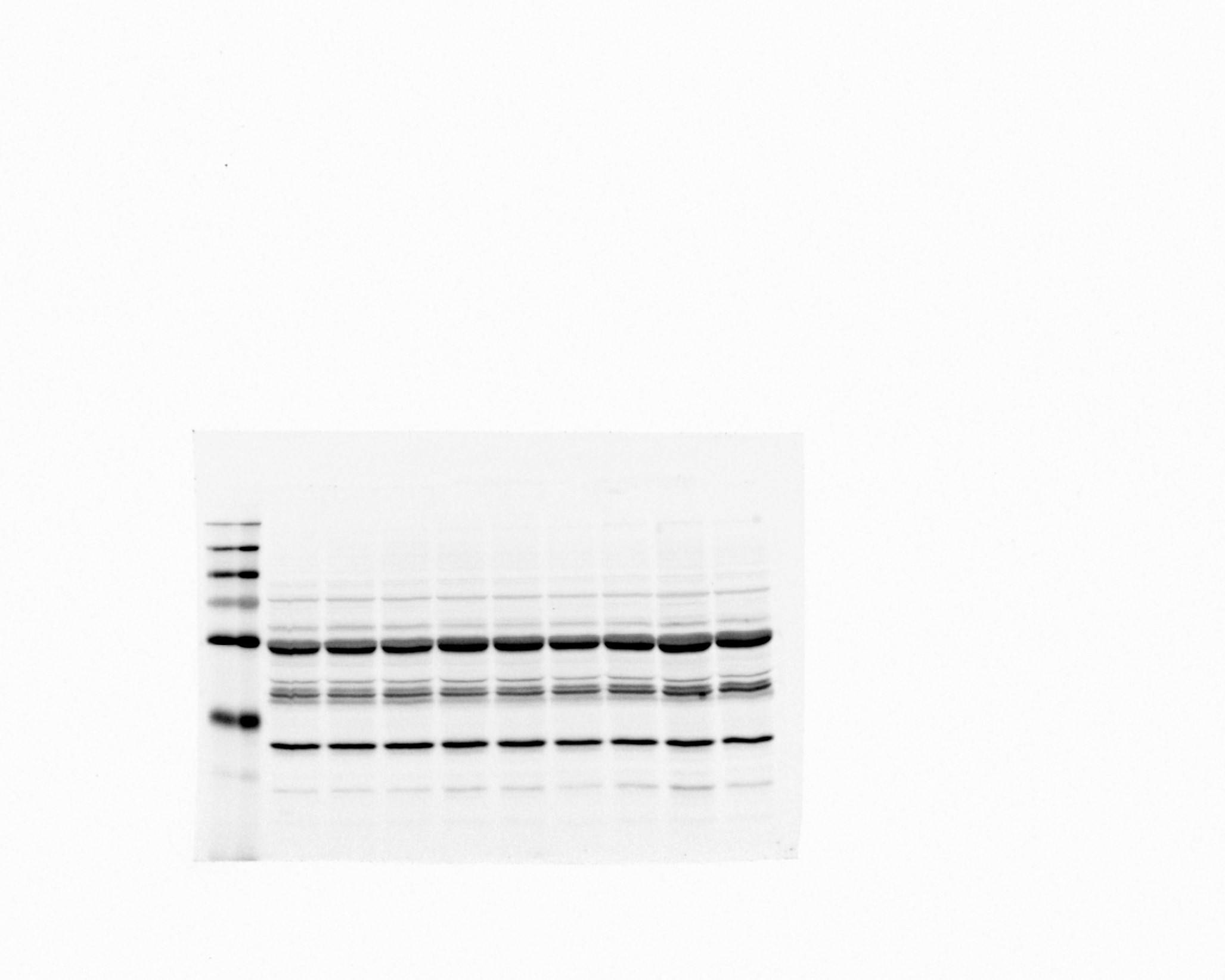

Supplement: Supplementary file 13 — Raw Western Blot and Microscopy Images [file 44318_2026_809_MOESM13_ESM.zip › SD_Blots/SD S3E/3E RACK1.jpg]

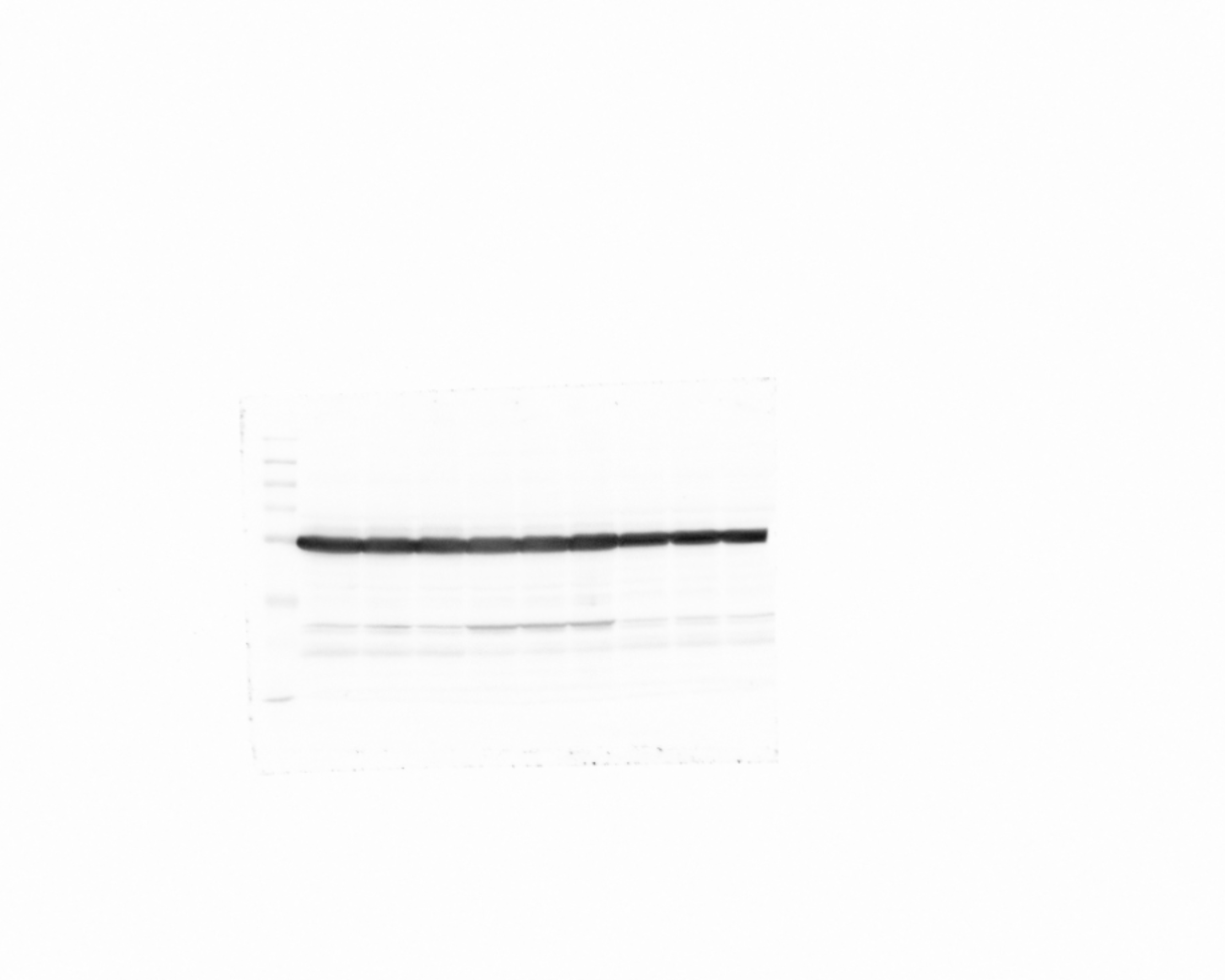

Supplement: Supplementary file 13 — Raw Western Blot and Microscopy Images [file 44318_2026_809_MOESM13_ESM.zip › SD_Blots/SD S3E/3E tubulin.tif]

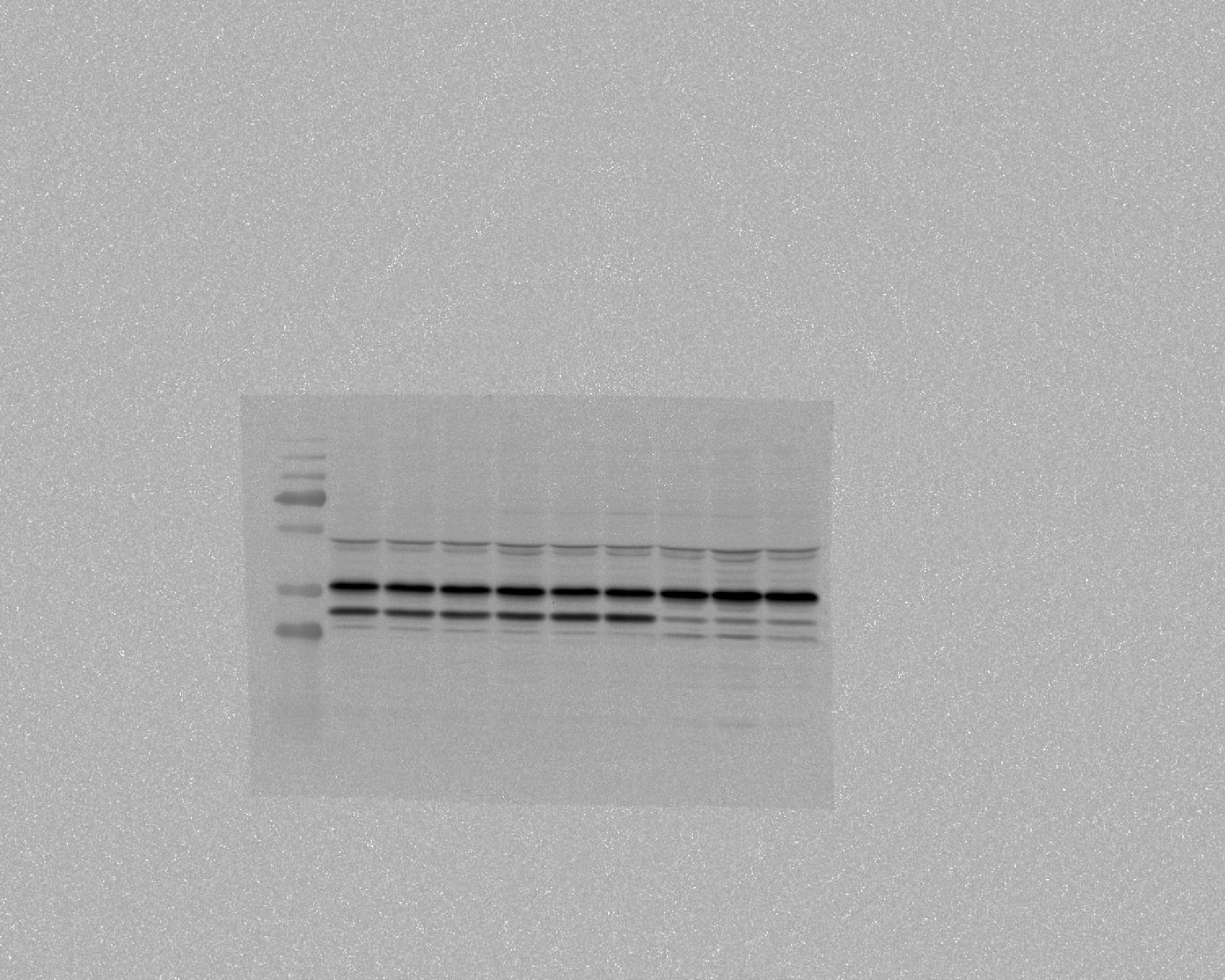

Supplement: Supplementary file 13 — Raw Western Blot and Microscopy Images [file 44318_2026_809_MOESM13_ESM.zip › SD_Blots/SD S3L/3L PP1.jpg]

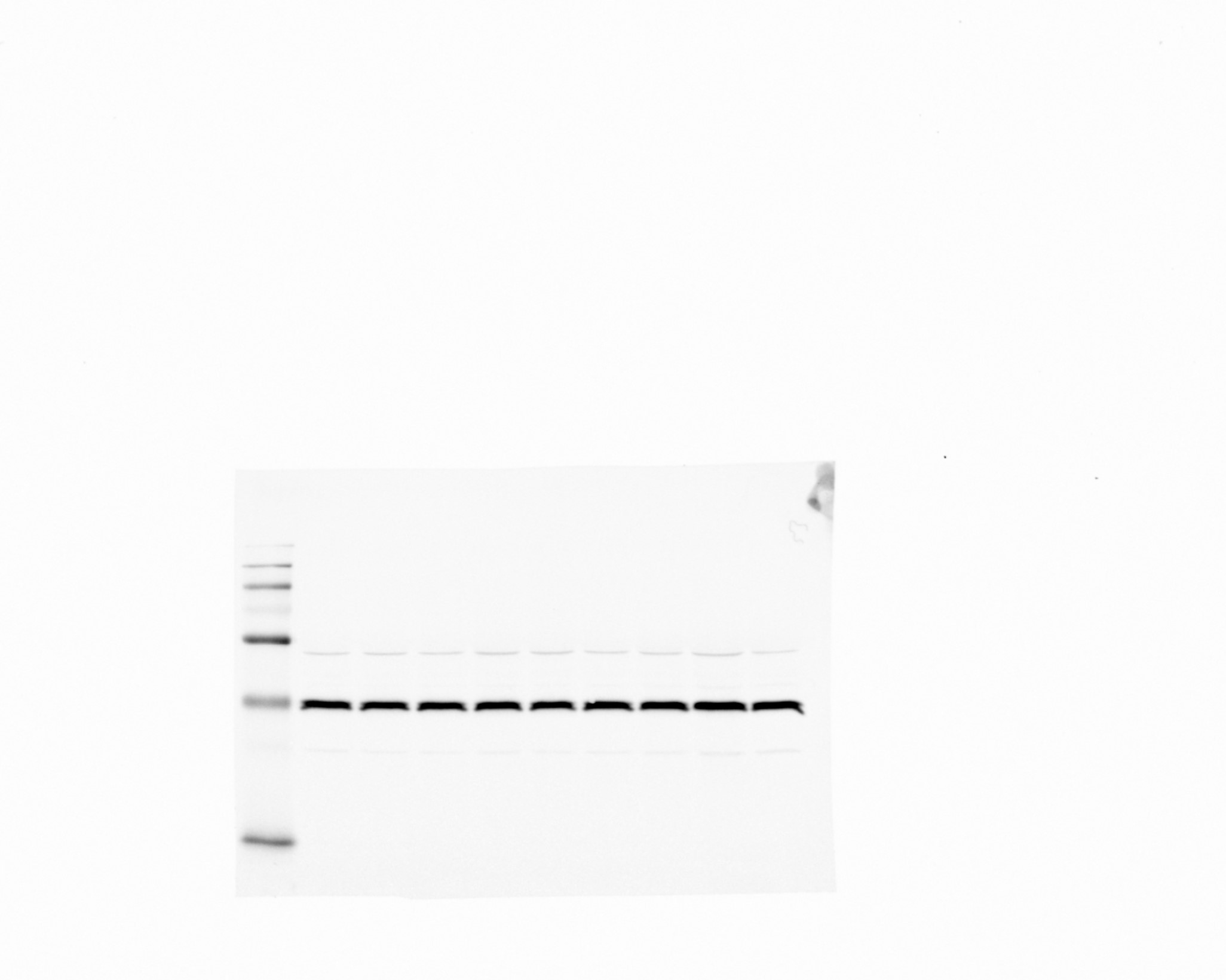

Supplement: Supplementary file 13 — Raw Western Blot and Microscopy Images [file 44318_2026_809_MOESM13_ESM.zip › SD_Blots/SD S3L/3L PP2A.jpg]

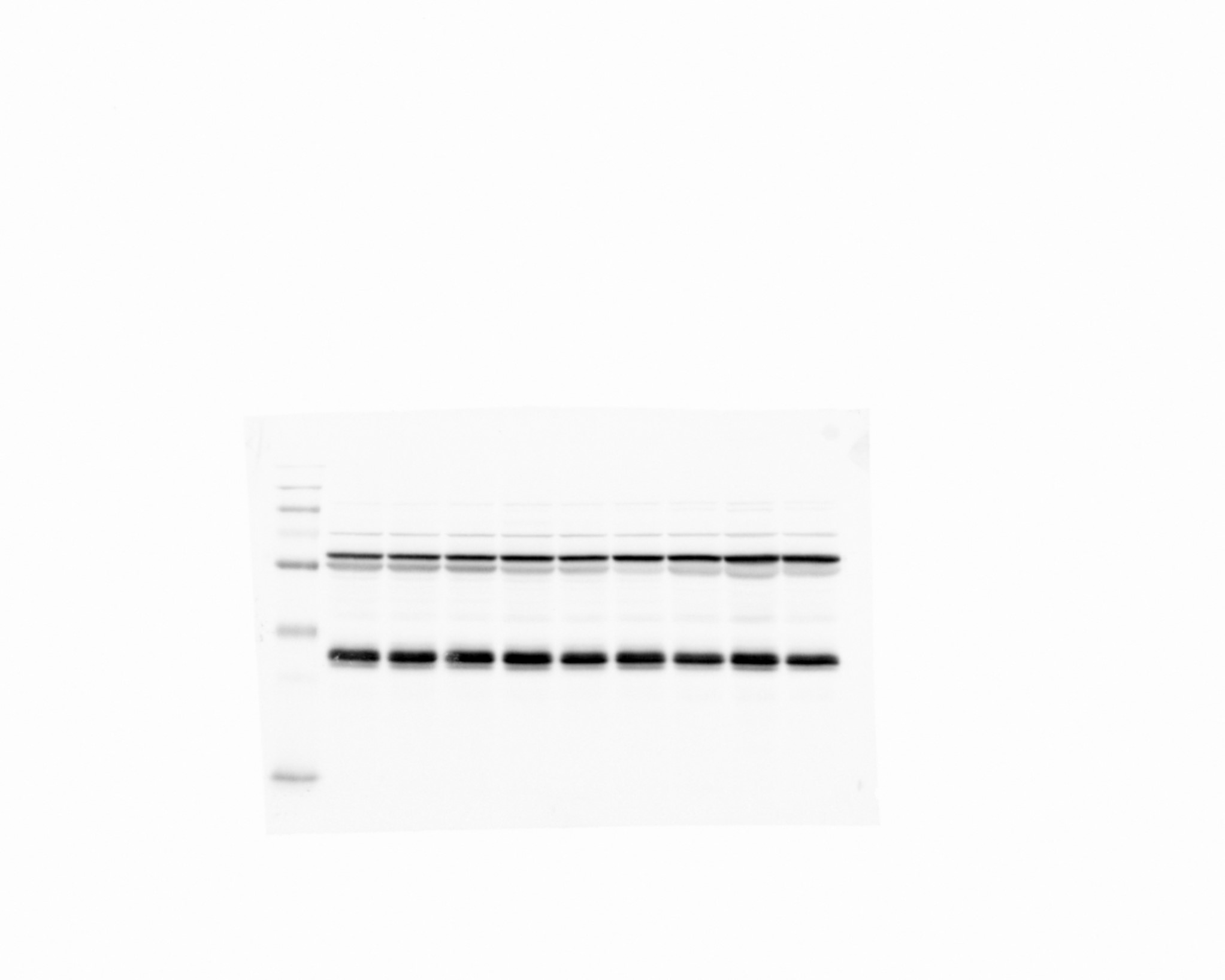

Supplement: Supplementary file 13 — Raw Western Blot and Microscopy Images [file 44318_2026_809_MOESM13_ESM.zip › SD_Blots/SD S3L/3L PP2B.jpg]

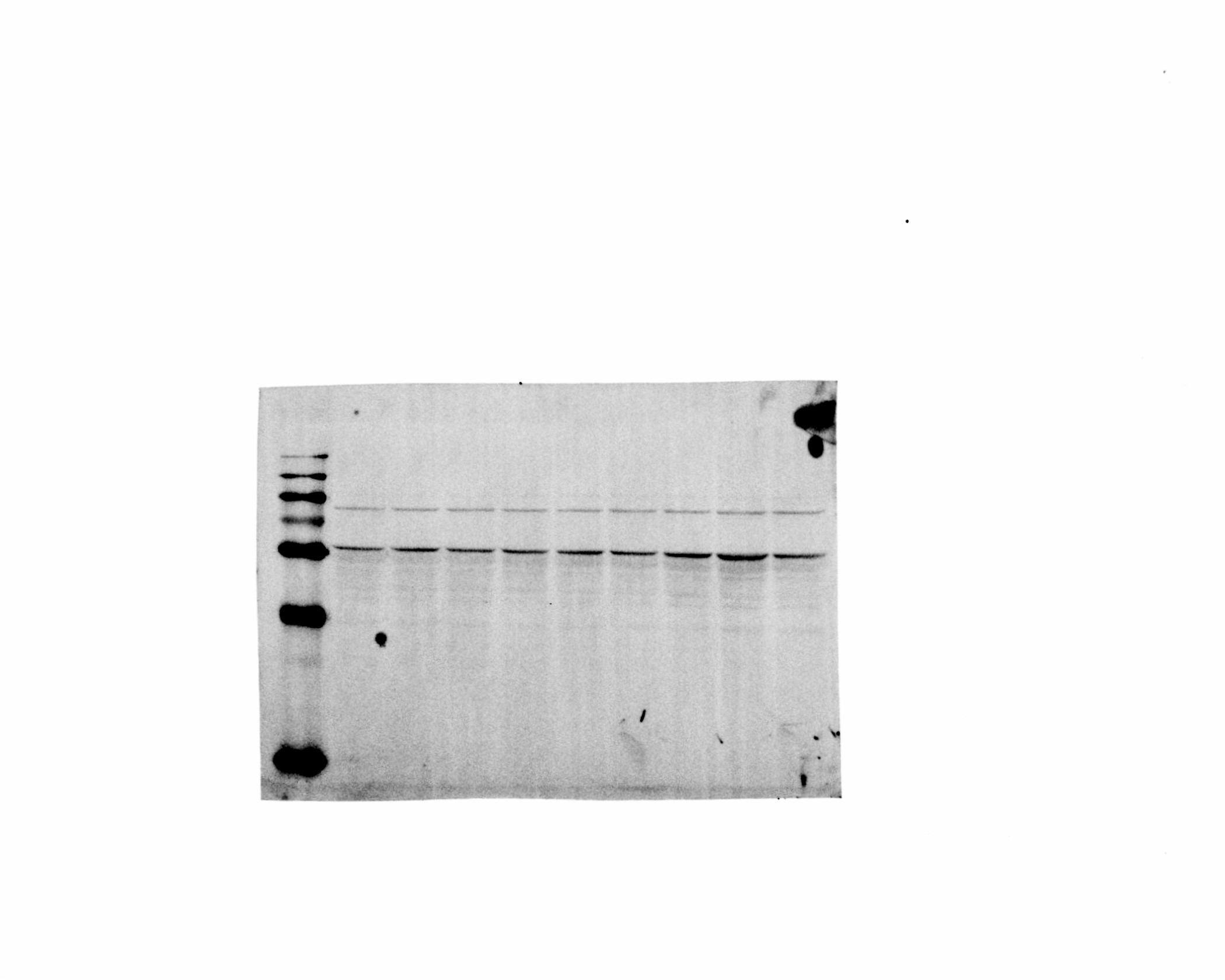

Supplement: Supplementary file 13 — Raw Western Blot and Microscopy Images [file 44318_2026_809_MOESM13_ESM.zip › SD_Blots/SD S3L/3L PP5.jpg]

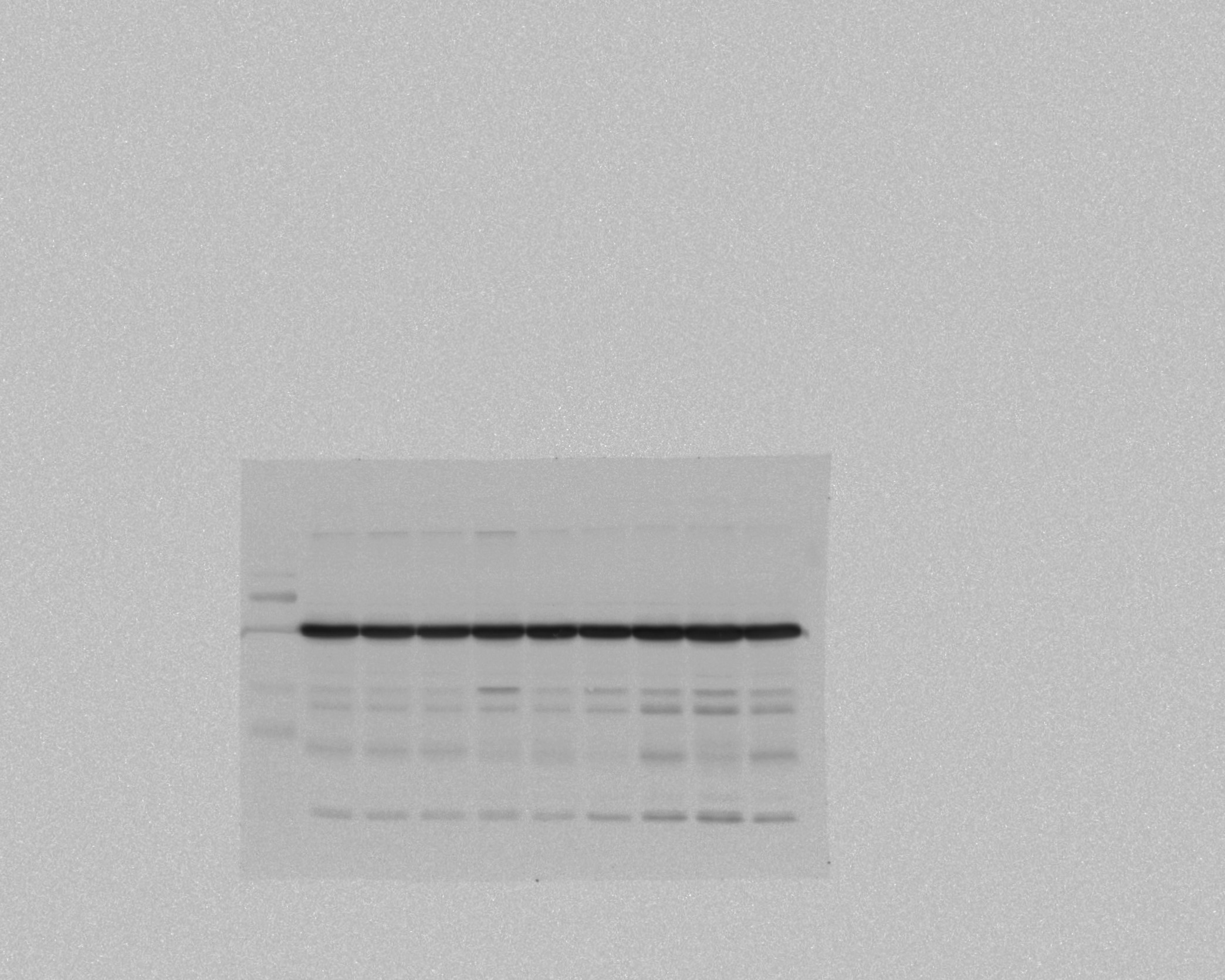

Supplement: Supplementary file 13 — Raw Western Blot and Microscopy Images [file 44318_2026_809_MOESM13_ESM.zip › SD_Blots/SD S3L/3L Tubulin.jpg]

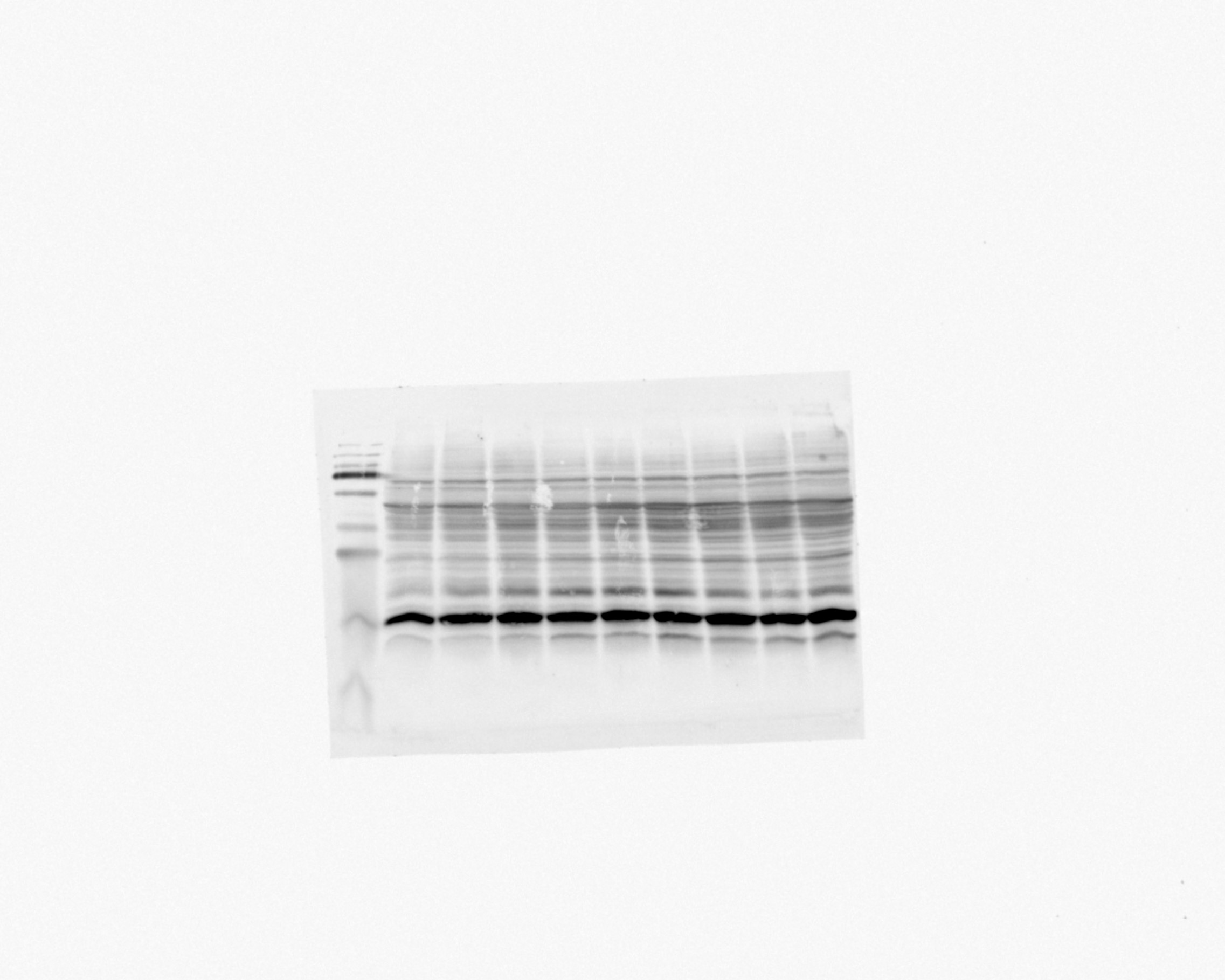

Supplement: Supplementary file 13 — Raw Western Blot and Microscopy Images [file 44318_2026_809_MOESM13_ESM.zip › SD_Blots/SD S5M/5M LC3 Iand II.jpg]

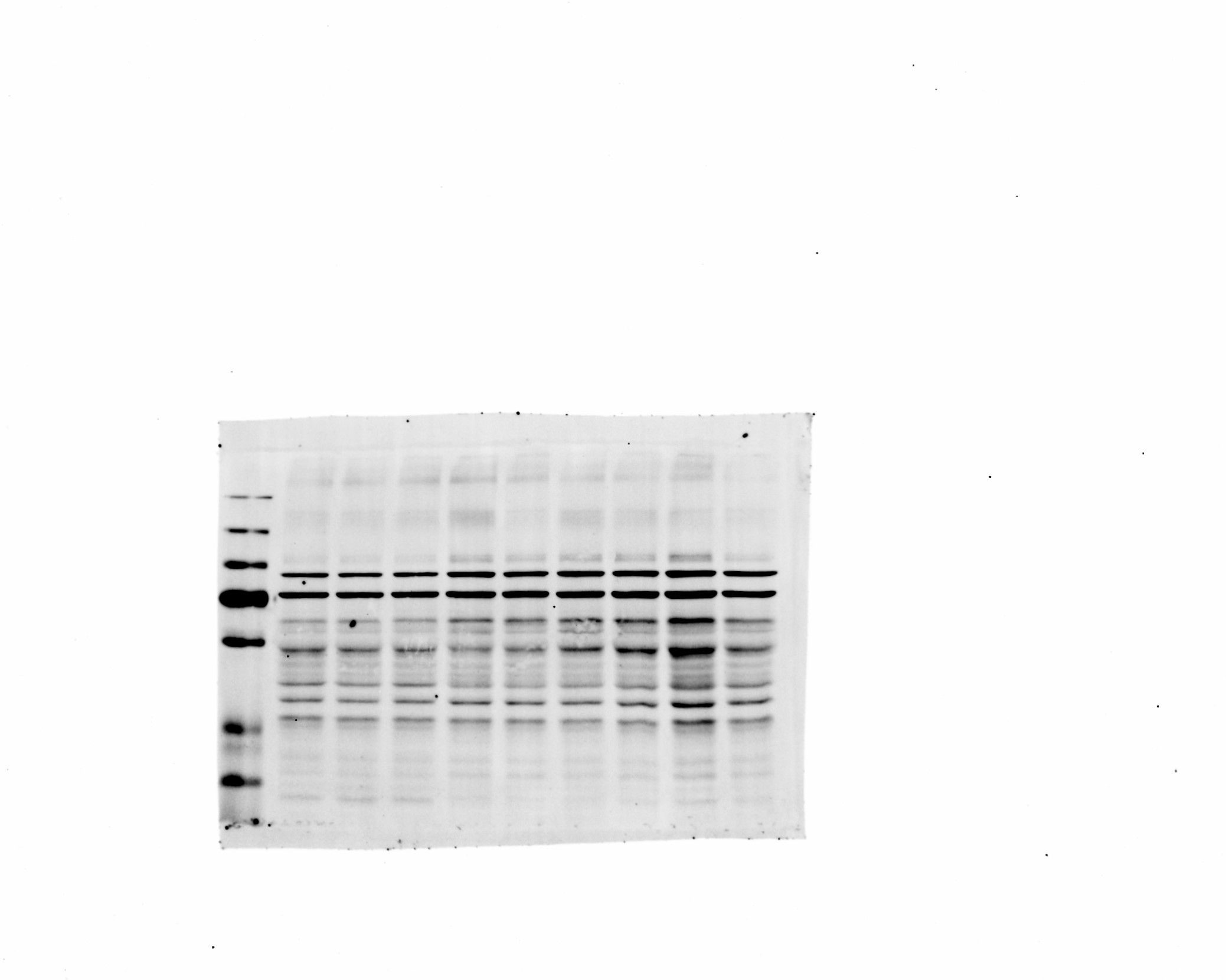

Supplement: Supplementary file 13 — Raw Western Blot and Microscopy Images [file 44318_2026_809_MOESM13_ESM.zip › SD_Blots/SD S5M/5M p62 blot.jpg]

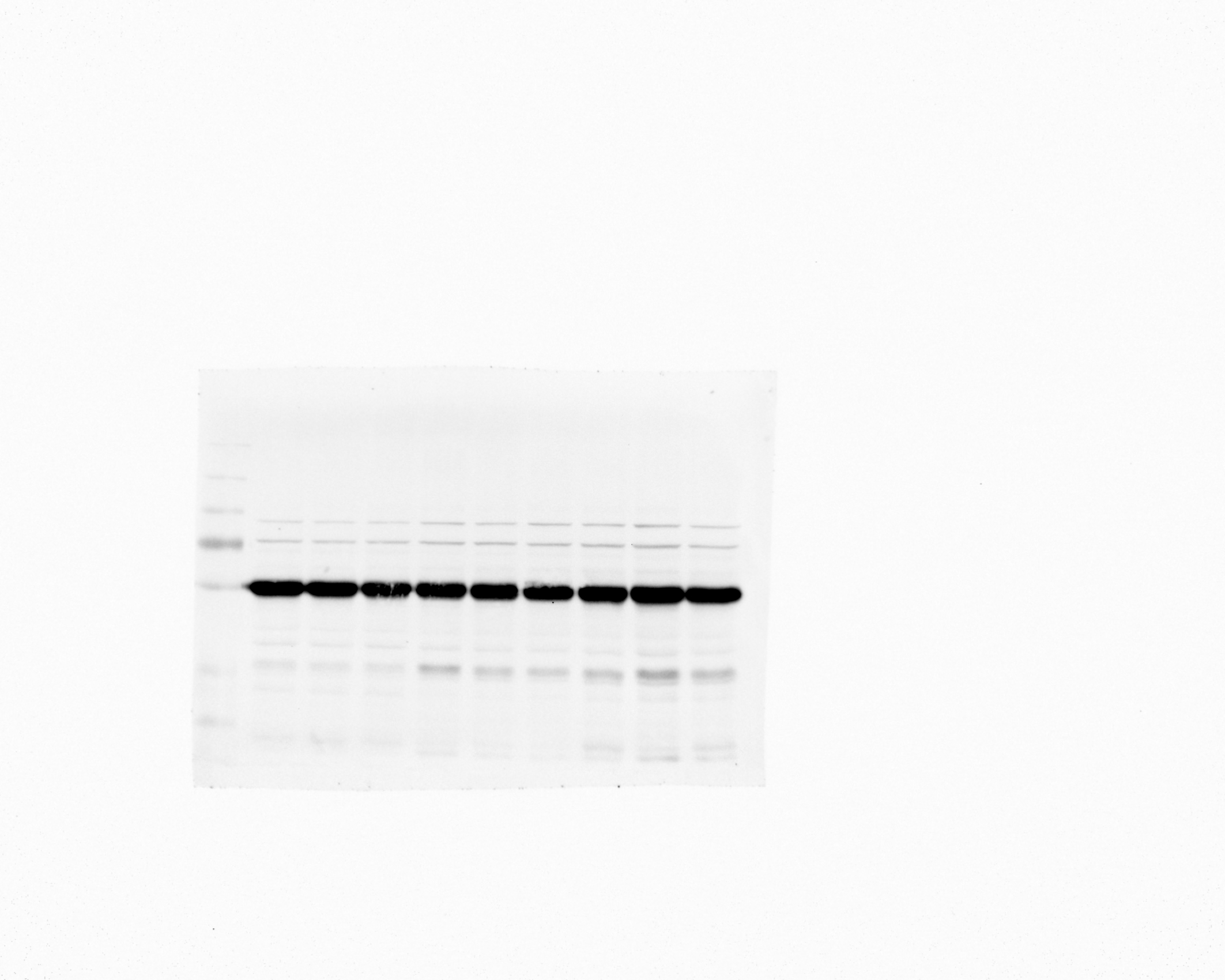

Supplement: Supplementary file 13 — Raw Western Blot and Microscopy Images [file 44318_2026_809_MOESM13_ESM.zip › SD_Blots/SD S5M/5M Tubulin.jpg]

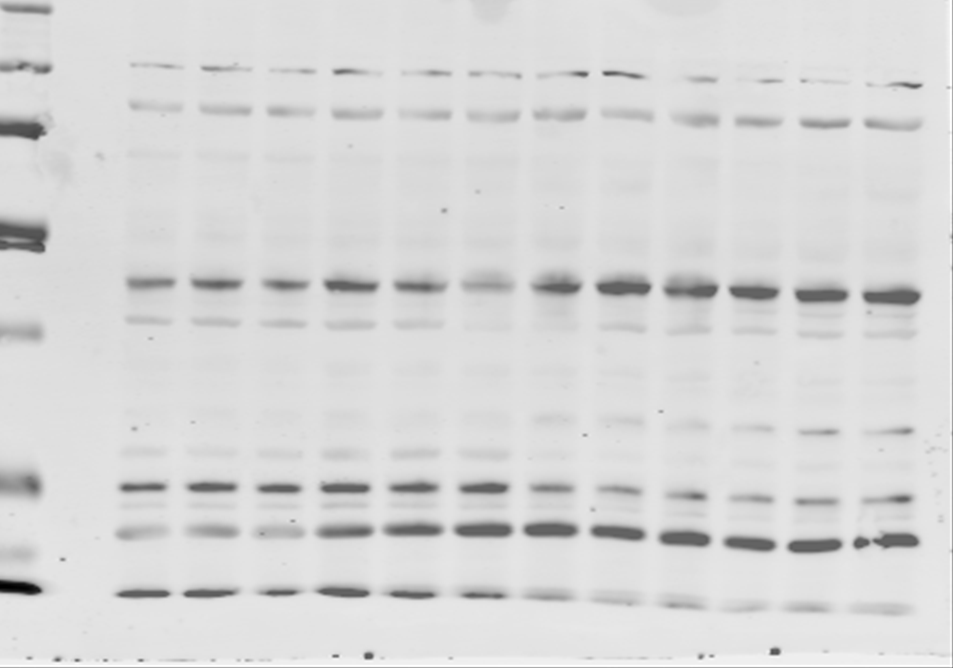

Supplement: Supplementary file 13 — Raw Western Blot and Microscopy Images [file 44318_2026_809_MOESM13_ESM.zip › SD_Blots/SD S5P/APH.tif]

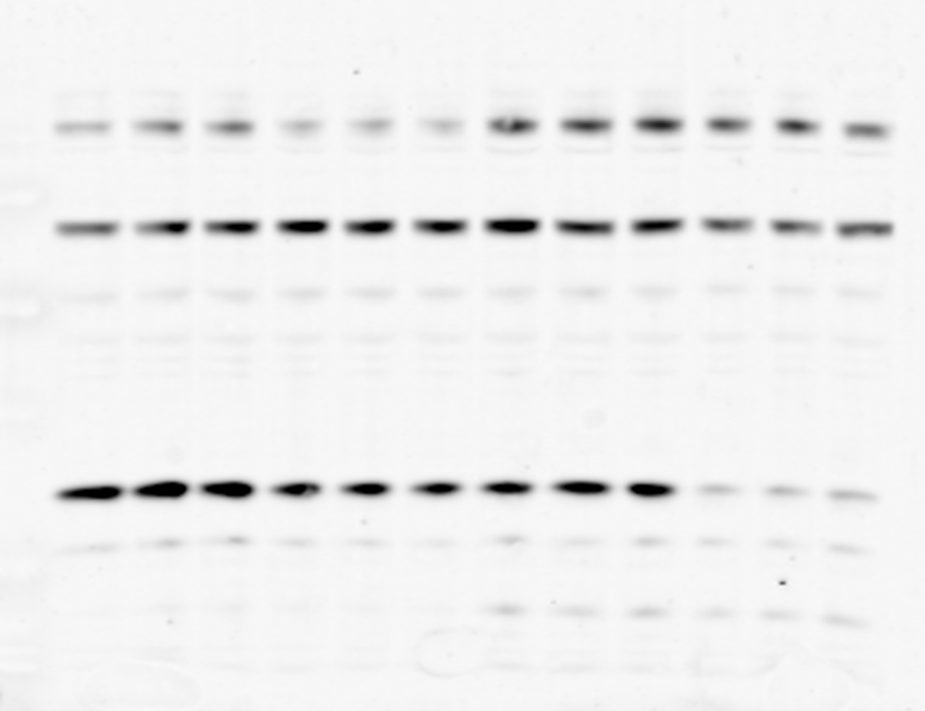

Supplement: Supplementary file 13 — Raw Western Blot and Microscopy Images [file 44318_2026_809_MOESM13_ESM.zip › SD_Blots/SD S5P/NCT.tif]

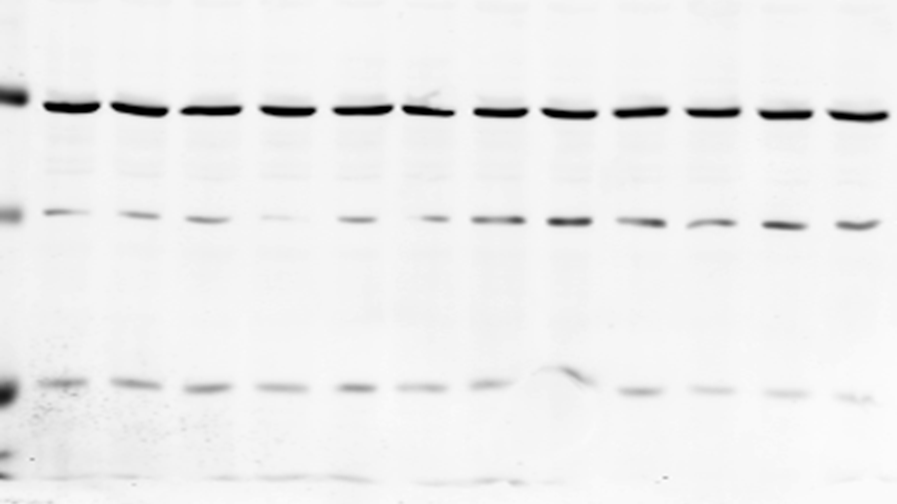

Supplement: Supplementary file 13 — Raw Western Blot and Microscopy Images [file 44318_2026_809_MOESM13_ESM.zip › SD_Blots/SD S5P/PS-1.tif]

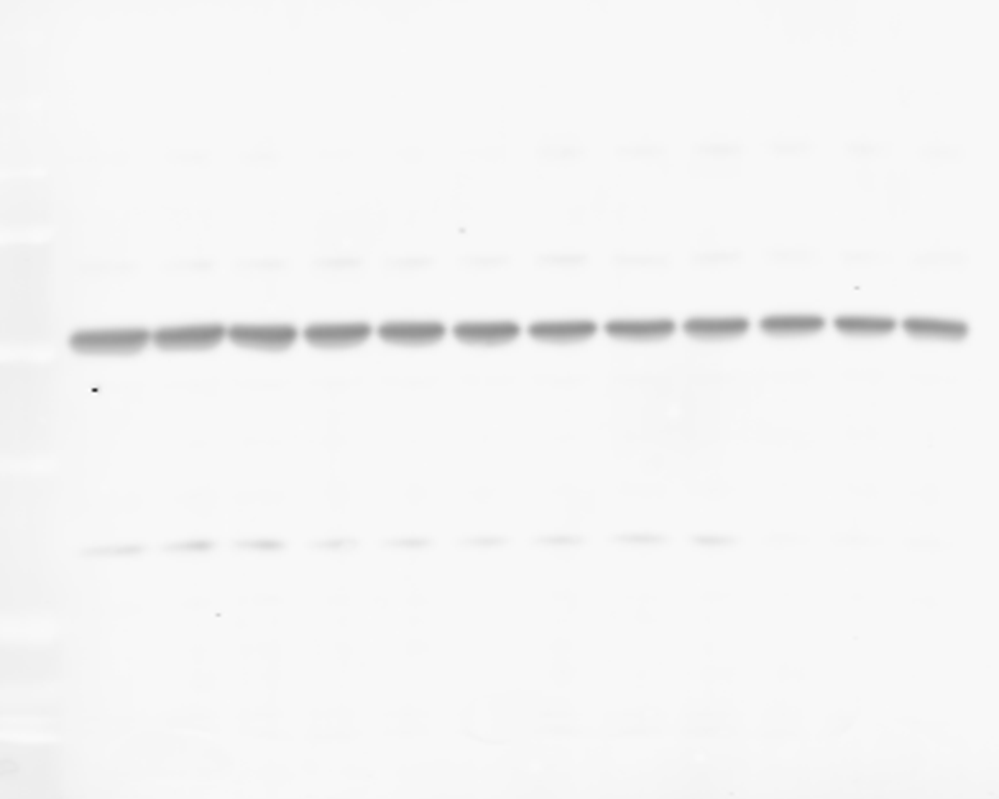

Supplement: Supplementary file 13 — Raw Western Blot and Microscopy Images [file 44318_2026_809_MOESM13_ESM.zip › SD_Blots/SD S5P/tubulin-1.tif]

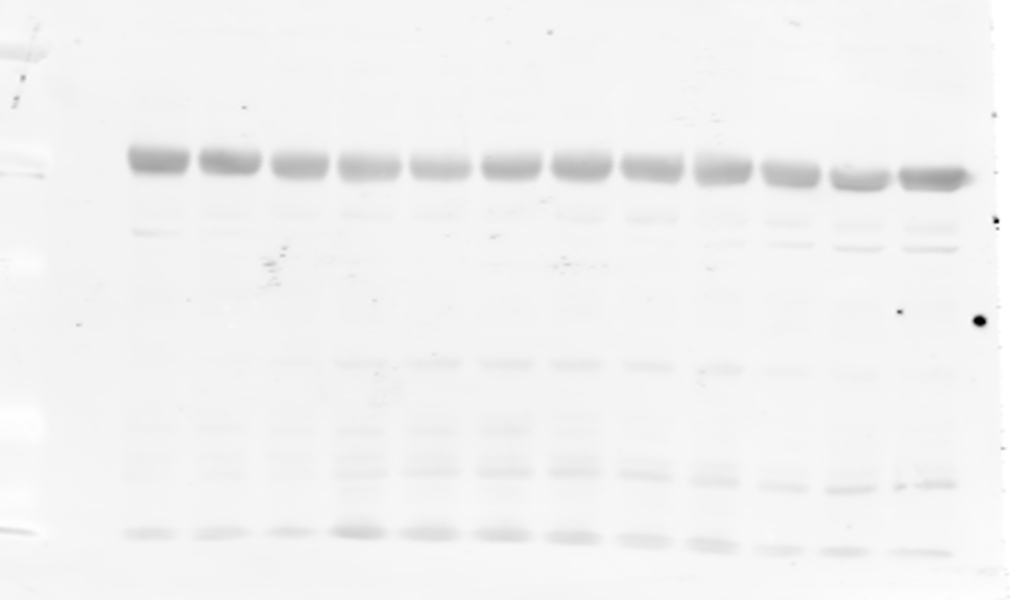

Supplement: Supplementary file 13 — Raw Western Blot and Microscopy Images [file 44318_2026_809_MOESM13_ESM.zip › SD_Blots/SD S5P/tubulin-2.tif]
