# Supplementary material for: Genetic ablation of neuronal mitochondrial calcium uptake impedes Alzheimer’s disease progression
Source: EMBO J. 2026 May 22;45(13):4469–91. doi: 10.1038/s44318-026-00809-w (PMC13324160; doi:10.1038/s44318-026-00809-w)
Supplement: Supplementary file 14 — Expanded View Figures [file 44318_2026_809_MOESM14_ESM.pdf]

Expanded View Figures

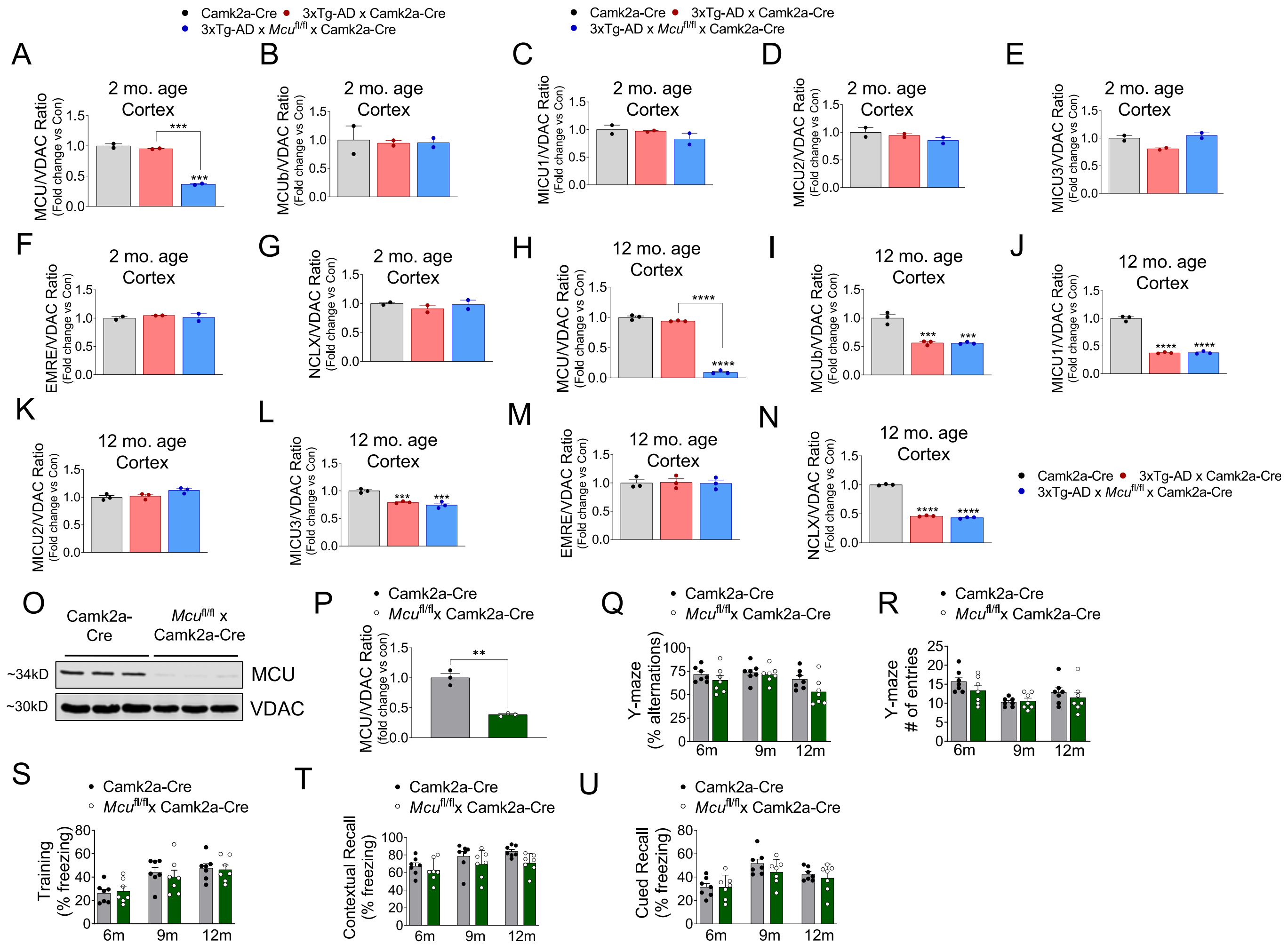

Figure EV1. $_m$**Ca**$^{2+}$ **exchanger expression and cognitive assay.**

(**A–N**) Quantification of protein expression associated with $_m$Ca^{2+} exchange expressed as fold-change vs. Camk2a-Cre con. corrected to a mitochondrial loading control VDAC, in tissue isolated from the brain cortex of 2- and 12-month-old mice, $n = 2$ per group in (**A–G**), $n = 3$ per group in (**H–N**). (**O, P**) Western blot validation and densitometry analysis for the expression of MCU protein in tissue isolated from the cortex of *Mcu*$^{fl/fl}$ x Camk2a-Cre mice compared to age-matched control corrected to VDAC, $n = 3$. (**Q, R**) Y-maze spontaneous alternation test, $n = 7$ per group. (**Q**) Percentage of spontaneous alternation. (**R**) Total number of arm entries. (**S–U**) Fear-conditioning test, $n = 7$ per group. (**S**) Freezing responses in the training phase. (**T**) Contextual recall freezing responses, (**U**) Cued recall freezing responses. n = individual dots shown for each group in all graphs. All data presented as mean ± SEM, ****$P < 0.0001$, ***$P < 0.001$, **$P < 0.01$, *$P < 0.05$. One-way ANOVA with Sidak's multiple comparisons test with adjusted P values shown in the graphs left to right: (**A**) $P = 0.0006$, $P = 0.0008$; (**H**) $P = 0.00000006$, $P = 0.00000009$; (**I**) $P = 0.00052$, $P = 0.00049$; (**J**) $P = 0.000001$, $P = 0.\ 000001$; (**L**) $P = 0.0000002$, $P = 0.0000002$; (**N**) $P = 0.000000007$, $P = 0.000000005$. To compare data in (**P**) t test was used with P value of 0.0011. Two-way ANOVA with Sidak's multiple comparisons test was performed to compare data in (**Q–S**). Source data are available online for this figure.

© The Author(s)

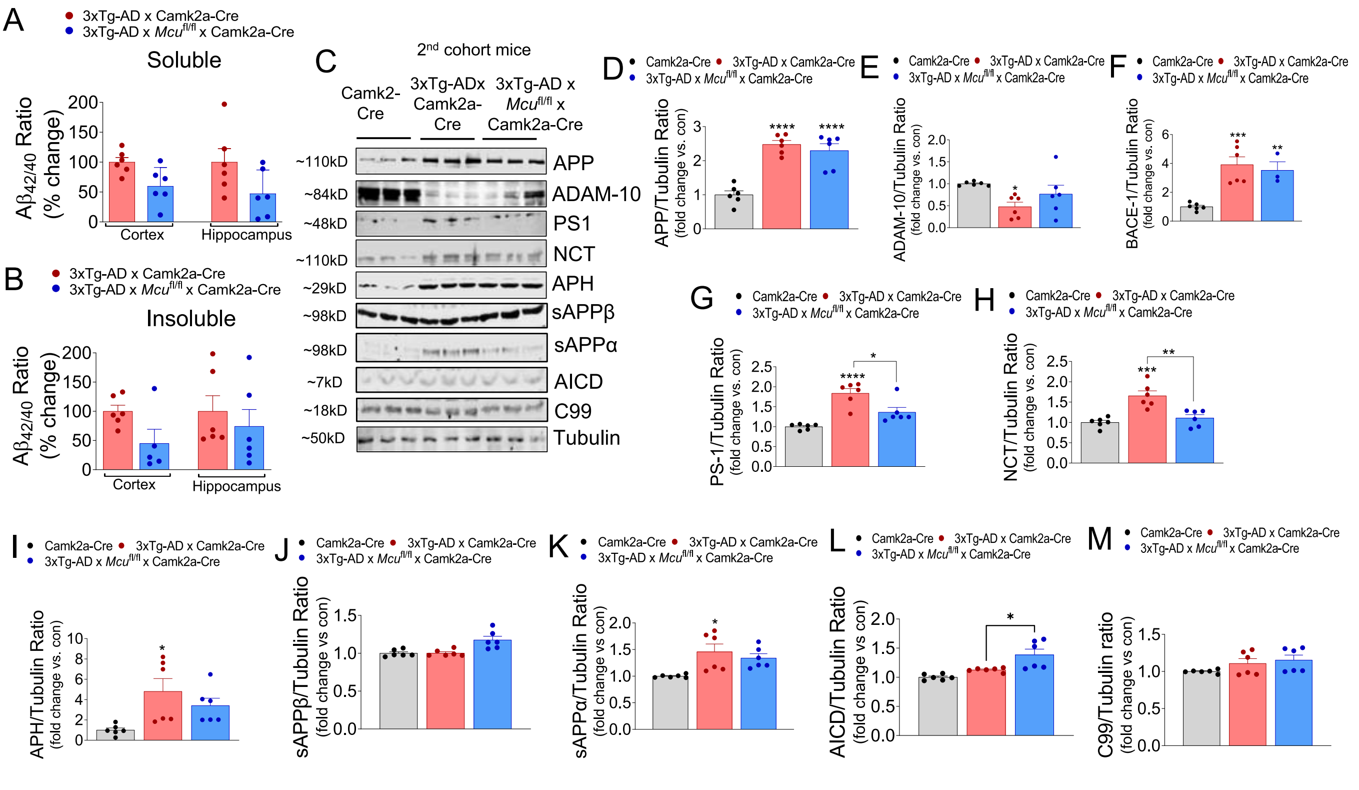

Figure EV2. Effect of genetic ablation of neuronal $_mCa^{2+}$ uptake on Aβ pathway.

(**A**) Soluble $Aβ_{1-42}/Aβ_{1-40}$ ratio in cortex and hippocampus of 15-month-old mice, measured by sandwich ELISA, $n = 6$ per group. (**B**) Insoluble $Aβ_{1-42}/Aβ_{1-40}$ ratio in cortex and hippocampus of 15-month-old mice, measured by sandwich ELISA, n(3xTg-AD × *Mcu*$^{fl/fl}$ × Camk2a-Cre, cortex) = 5, for other groups $n = 6$. (**C**) Western blots of full-length APP, ADAM-10, PS1, nicastrin, APH, sAPPα, sAPPβ, AICD, C99 and tubulin (loading control) for cortex homogenate of 15-month-old mice (second cohort mice), $n = 3$ for all groups. (**D–M**) Densitometry analysis of Western blots shown in Fig. 2G and (**C**), expressed as fold-change vs. Camk2a-Cre con. corrected to a loading control tubulin. $n = 6$ individual dots shown for each group in all graphs except for (**F**) where n(3xTg-AD × *Mcu*$^{fl/fl}$ × Camk2a-Cre) = 3. All data presented as mean ± SEM, ****$P < 0.0001$, ***$P < 0.001$, **$P < 0.01$, *$P < 0.05$. One-way ANOVA with Sidak's multiple comparisons test was performed with adjusted *P* values shown in the graph left to right: (**D**) $P = 0.000012$, $P = 0.000053$; (**E**) $P = 0.0384$; (**F**) $P = 0.0006$, $P = 0.009$; (**G**) $P = 0.000064$, $P = 0.01$; (**H**) $P = 0.0003$, $P = 0.0017$; (**I**) $P = 0.0186$; (**K**) $P = 0.0127$. One-way ANOVA with Tukey's multiple comparisons test was performed in (**L**) with adjusted *P* value shown, $P = 0.0176$. Source data are available online for this figure.

 © The Author(s)

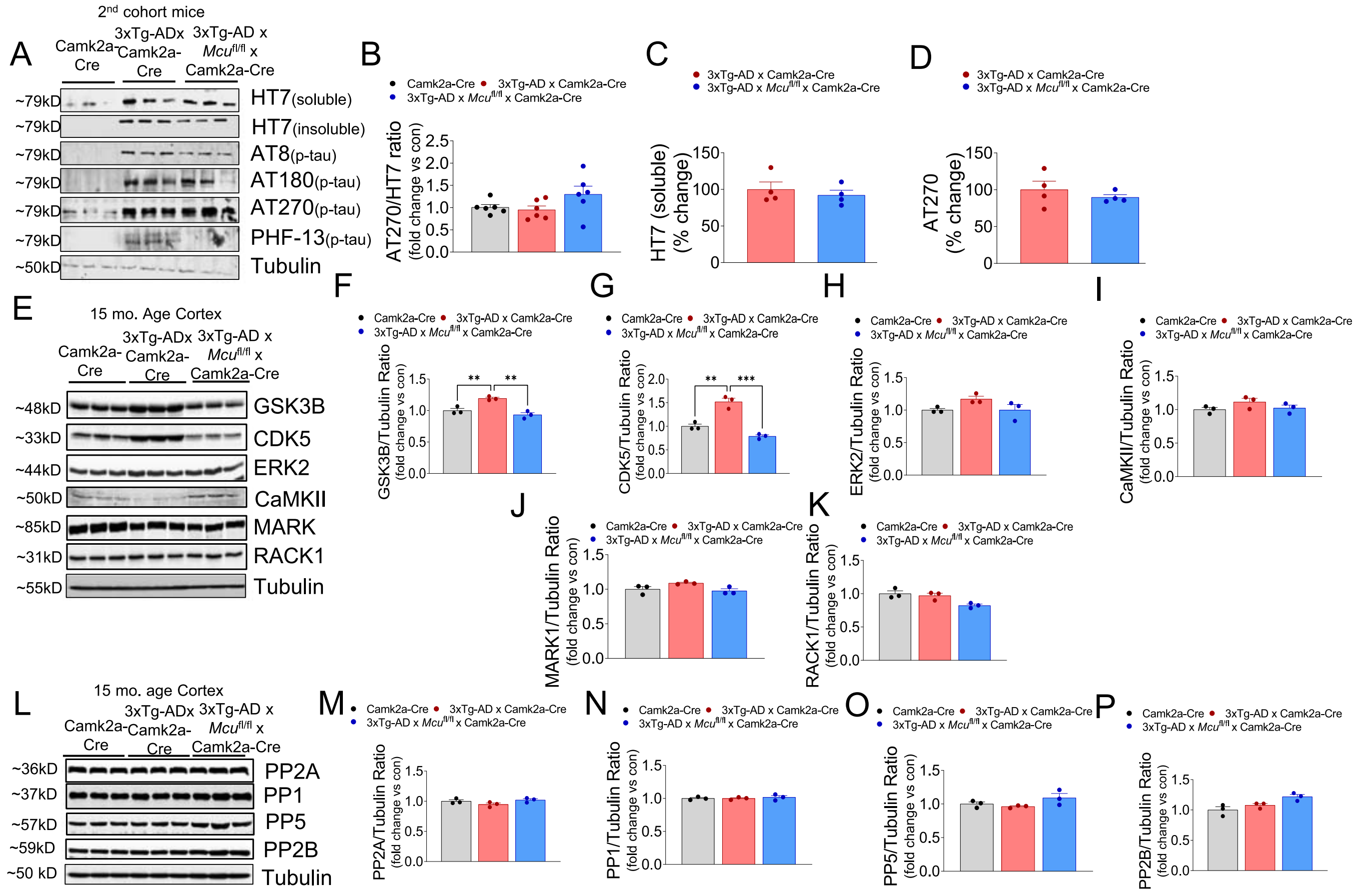

Figure EV3. Effect of loss of neuronal MCU expression on tau pathology.

(**A**) Western blots (2nd cohort mice) of soluble and insoluble total tau (HT7), phosphorylated tau at residues S202/T205 (AT8), T231/S235 (AT180), T181 (AT270), and S396 (PHF13) in cortex homogenate of 15-month-old mice, $n = 3$ for all groups. (**B–D**) Densitometric analysis of Western blots shown in Fig. 3A, and (**A**), expressed as fold-change vs. Camk2a-Cre con. corrected to a loading control, tubulin. Quantification is the sum of all replicates, $n = 6$. (**E**) Western blots for protein expression of GSK3β, CDK5, ERK2, CamkII, MARK, RACK1 and tubulin (loading control) for cortex homogenate of 15-month-old mice, $n = 3$ for all groups. (**F–K**) Densitometric analysis of western blots shown in (**E**), expressed as fold-change vs. Camk2a-Cre con. corrected to a loading control, tubulin, $n = 3$ for all groups. (**L**) Western blots for protein expression of PP2A, PP1, PP5, PP2B and tubulin (loading control) for cortex homogenate of 15-month-old mice, $n = 3$ for all groups. (**M–P**) Densitometric analysis of western blots shown in (**L**), expressed as fold-change vs. Camk2a-Cre con. corrected to a loading control, tubulin, $n = 3$ per group. All data presented as mean ± SEM, $^{****}P < 0.0001$, $^{***}P < 0.001$, $^{**}P < 0.01$, $^{*}P < 0.05$. To compare data in (**C**, **D**) t test was used. One-way ANOVA with Tukey's multiple comparisons test was performed for other panels with adjusted P value shown from right to left: (**F**) $P = 0.009$, $P = 0.002$; (**G**) $P = 0.0012$, $P = 0.0002$. Source data are available online for this figure.

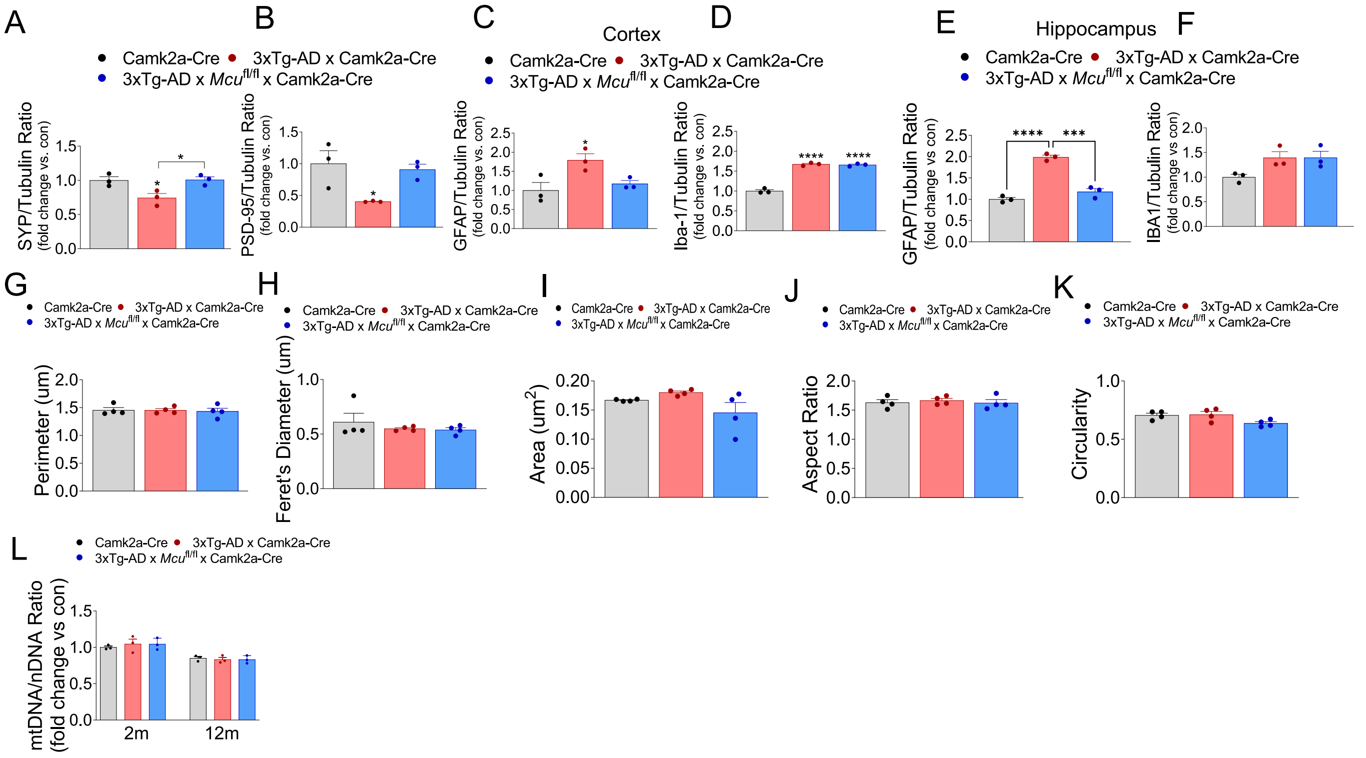

Figure EV4. Loss of neuronal MCU preserves synaptic integrity and mitochondrial ultrastructure in AD mice.

(A–F) Densitometric analysis of Western blots shown in Fig. 4G–I for SYP, PSD-95, GFAP and IBA1 expression. $n = 3$ for all groups. **(G, K)** Quantification of the shape descriptors and morphological parameters for mitochondria. $n = 4$ for all groups. **(G)** Perimeter (μM). **(H)** Feret's diameter (μM). **(I)** Area (μM^2). **(J)** Aspect ratio. **(K)** Circularity. **(L)** Mitochondrial DNA (mtDNA)/nuclear DNA (nDNA) ratio in tissue isolated from the cortex of 2- and 12-months old mice, fold change vs. 2-month-old Camk2a-Cre controls, $n = 3$ for all groups. All data presented as mean ± SEM, $^{****}P < 0.0001$, $^{***}P < 0.001$, $^{**}P < 0.01$, $^{*}P < 0.05$. One-way ANOVA with Sidak's multiple comparisons test was performed in (**A**, **G–K**) with adjusted P values in (**A**), $P = 0.046$ and $P = 0.0409$. One-way ANOVA with Tukey's multiple comparisons test was performed in (**B–F**) with adjusted P values shown in the graphs left to right: **(B)** $P = 0.0379$; **(C)** $P = 0.031$; **(D)** $P = 0.000005$, $P = 0.\ 000005$; **(E)** $P = 0.000045$, $P = 0.\ 000135$. Two-way ANOVA with Sidak's multiple comparisons test was performed in (**L**). Source data are available online for this figure.

 © The Author(s)

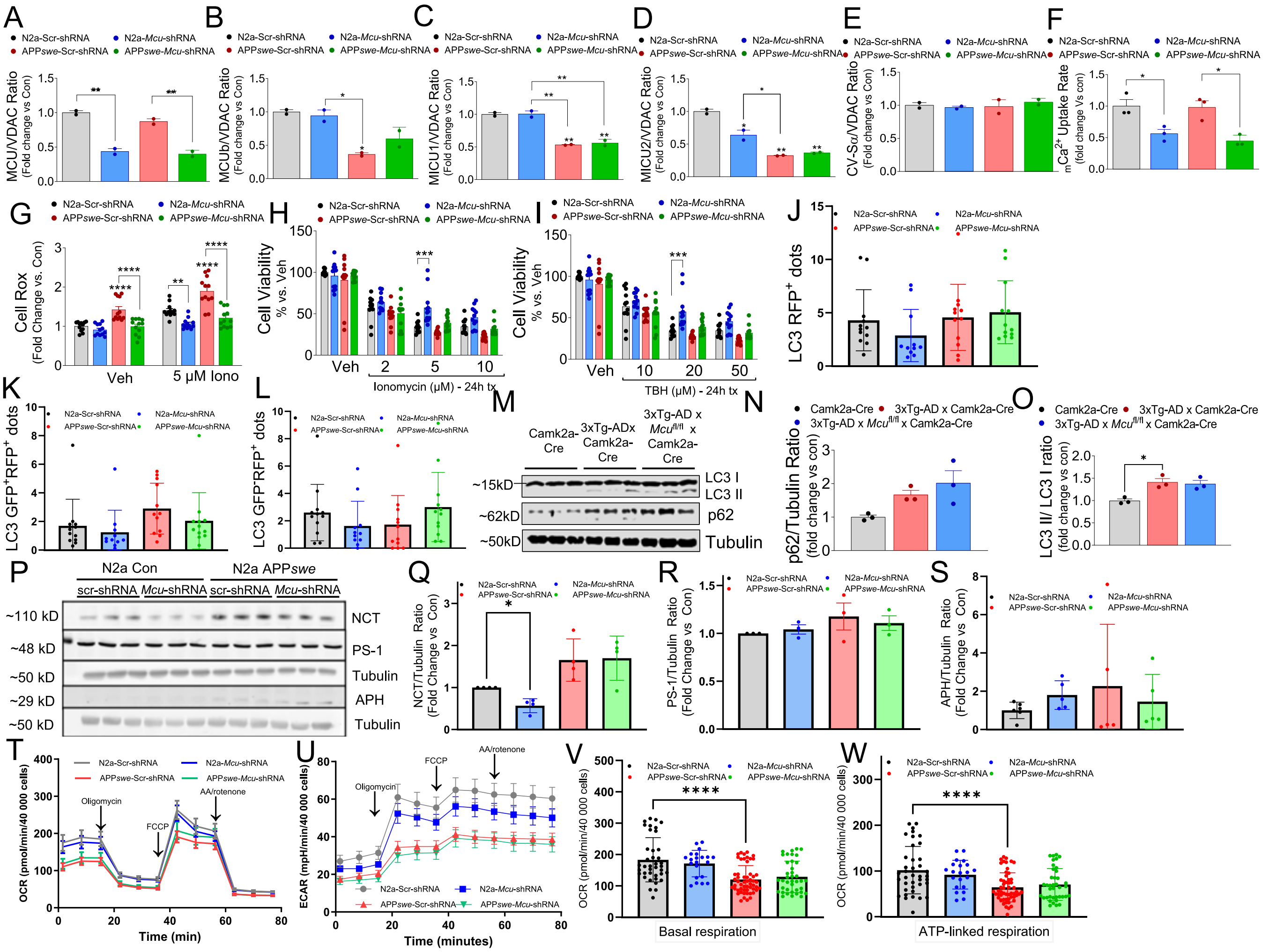

Figure EV5. Loss of MCU reduces mitochondrial Ca^{2+} uptake, oxidative stress, and protein aggregation and restores autophagic flux in APPswe cells.

(**A–E**) Quantification of protein expression associated with $_mCa^{2+}$ exchange expressed as fold-change vs. N2a Scr-shRNA con. and corrected to a mitochondrial loading control, VDAC, $n = 2$ for each group. (**F**) Fold-change in $_mCa^{2+}$ uptake rate of N2a-*Mcu*-shRNA, APPswe and APPswe -*Mcu*-shRNA vs. N2a Scr-shRNA controls, $n = 3$ per group. (**G**) Quantification of CellRox green fluorescent intensity (total cellular ROS production); fold change vs. N2a Scr-shRNA controls. (**H, I**) Assessment for cell viability using Cell Titer Blue after treatment with, (**H**) Ionomycin (Ca^{2+} overload, 2–10 µM), (**I**) tert-Butyl hydroperoxide (TBH, oxidizing agent, 10–50 µM), $n = 12$ individual dots shown for each group. (**J–L**) Quantification of autophagic structures per cell (n (*Mcu*-shRNA) = 11, $n = 12$ for other groups), including: (**J**) RFP⁺ puncta representing total red puncta (**K**) GFP⁺RFP⁺ puncta representing autophagosomes (**L**) GFP⁻RFP⁺ puncta representing autolysosomes. (**M**) Representative western blot of LC3-I, LC3-II, p62 and tubulin (loading control) for cortex homogenate of 12-month-old mice, $n = 3$ for all groups. (**N, O**) Densitometry analysis of western blots shown in Fig. EV2M expressed as fold-change vs. Camk2a-Cre con. corrected to tubulin $n = 3$/groups. (**P**) Western blots for protein expression of NCT, PS-1, APH and tubulin (loading control) for cells homogenate of N2a Scr-shRNA, N2a-*Mcu*-shRNA, APPswe and APPswe -*Mcu*-shRNA. (**Q–S**) Densitometric analysis of western blots shown in (**P**), expressed as fold-change vs. N2a Scr-shRNA con. corrected to a loading control, tubulin, $n = 4$ per group in (**Q**), $n = 3$ per group in (**R**), $n = 5$ per group in (**S**) except for n (Scr-shRNA) = 6. (**T–W**) Metabolic profiling of neuronal cells, n (scr-shRNA) = 39, n (*Mcu*-shRNA) = 21, n (APPswe- scr-shRNA) = 54, n (APPswe-*Mcu*-shRNA) = 39. (**T**) Representative traces of oxygen consumption rate (OCR) analysis. (**U**) Representative extracellular acidification rate (ECAR) traces. (**V, W**) Quantification of basal respiration and ATP-linked respiration in N2a Scr-shRNA, N2a Mcu-shRNA, APPswe, and APPswe Mcu-shRNA cells. Data presented as mean ± SEM, ****$P < 0.0001$, ***$P < 0.001$, **$P < 0.01$, *$P < 0.05$. One-way ANOVA with Sidak's multiple comparisons test was performed in (**A, C–F, J–L, V, W**) with adjusted P values shown in the graphs left to right: (**A**) $P = 0.0025$, $P = 0.005$; (**C**) $P = 0.0036$, $P = 0.0037$, $P = 0.0045$, $P = 0.0047$; (**D**) $P = 0.0221$, $P = 0.0371$, $P = 0.0221$, $P = 0.0227$; (**F**) $P = 0.0398$, $P = 0.0136$; (**V**) $P = 0.000000286$; (**W**) $P = 0.000025$. One-way ANOVA with Tukey's multiple comparisons test was performed in (**B, N, O**) with adjusted P values shown in the graphs left to right: (**B**) $P = 0.0149$, $P = 0.0208$; (**C**) $P = 0.031$. Repeated measures ANOVA with Sidak's multiple comparisons test was performed in (**Q–R**) with adjusted P value in (**Q**) $P = 0.0401$. Two-way ANOVA with Sidak's multiple comparisons test was performed in (**G–I, S**) with adjusted P values shown in the graphs left to right: (**G**) $P = 0.000095$, $P = 0.000076$, $P = 0.0026$, $P = 0.0000049$, $P = 0.0000000005$; (**H**) $P = 0.000117$; (**I**) $P = 0.0002$. Source data are available online for this figure.

© The Author(s)
