# Supplementary material for: Genetic ablation of neuronal mitochondrial calcium uptake impedes Alzheimer’s disease progression
Source: EMBO J. 2026 May 22;45(13):4469–91. doi: 10.1038/s44318-026-00809-w (PMC13324160; doi:10.1038/s44318-026-00809-w)
Supplement: Supplementary file 2 — Peer Review File [file 44318_2026_809_MOESM2_ESM.pdf]

THE EMBO JOURNAL

Genetic ablation of neuronal mitochondrial calcium uptake impedes disease progression in models of Alzheimer's disease

Pooja Jadiya, Elena Berezhnaya, Devin Kolmetzky, Dhanendra Tomar, Henry Cohen, Shatakshi Shukla, Manfred Thomas, Salman Khaledi, Joanne Garbincius, Liam Kennedy, Oniel Salik, Darpan Raghav, Alycia Hildebrand, and John Elrod

Corresponding author(s): John Elrod (elrod@temple.edu)

Review Timeline:

Submission Date:	10th Oct 23
Editorial Decision:	12th Dec 23
Revision Received:	25th Sep 25
Editorial Decision:	11th Feb 26
Revision Received:	26th Feb 26
Accepted:	27th Apr 26

Editor: Ioannis Papaioannou

Transaction Report:

(Note: With the exception of the correction of typographical or spelling errors that could be a source of ambiguity, letters and reports are not edited. Depending on transfer agreements, referee reports obtained elsewhere may or may not be included in this compilation. Referee reports are anonymous unless the Referee chooses to sign their reports.)

Dear John,

Thank you for submitting your manuscript for consideration by The EMBO Journal. It has been seen by three experts in the field, and we have received the full set of their comments, a copy of which I have already sent you (included again below). I would also like to thank you for your detailed point-by-point response to their comments and concerns, and your provisional revision plan.

The referees recognize that the topic is interesting and relevant, the experiments largely convincing, and the findings interesting. However, they also raise a number of concerns and identify some limitations. Notably, there is a consensus among them that there are some ambiguities that have to be clarified and that additional mechanistic insight should be provided. According to your response to these comments, you are willing and able to address the referees' concerns and strengthen the manuscript further.

Given the referees' positive comments and recommendations, and your provisional revision plan, I would like to invite you to submit a revised version of your manuscript, addressing the comments of all three reviewers. I should add that it is EMBO Journal policy to allow only a single round of major revision, and acceptance of your manuscript will therefore depend on the completeness of your responses in this revised version. If you have any questions or comments, we can also discuss the revisions in a video chat, if you like.

We generally allow three months as standard revision time (11th March 2024). As a matter of policy, competing manuscripts published during this period will not negatively impact our assessment of the conceptual advance presented by your study. However, we request that you contact us as soon as possible upon publication of any related work, to discuss how to proceed. Should you foresee a problem in meeting this three-month deadline, please let us know in advance and we may be able to grant an extension.

Thank you for the opportunity to consider your work for publication in The EMBO Journal. I look forward to your revision.

Best regards,

Ioannis

Ioannis Papaioannou, PhD
Editor, The EMBO Journal
i.papaioannou@embojournal.org

*****
Instructions for preparing your revised manuscript
*****

1. When you are ready to submit the revision, please upload:

- A Word file of the manuscript text (including legends of main Figures, EV Figures and Tables). Please make sure that changes are highlighted (or "tracked") to be clearly visible.

- Individual production-quality figure files (one file per figure). When assembling your figures, please refer to our figure preparation guidelines in order to ensure proper formatting and readability in print as well as on screen:
https://bit.ly/EMBOPressFigurePreparationGuideline
If the data shown in a figure are obtained from n {less than or equal to} 2, please use scatter plots showing the individual data points.
Please also include scale bars in all microscopy images.
The following points must be specified in each figure legend:
i. the name of the statistical test used to generate error bars and P values
ii. the number (n) of independent experiments (please specify technical or biological replicates) underlying each data point (discussion of statistical methodology can be reported in the Materials and Methods section, but figure legends should contain a basic description of n, P, and the test applied)
iii. the nature of the bars and error bars (s.d., s.e.m.).
See also guidelines for figure legends: https://www.embopress.org/page/journal/14602075/authorguide#figureformat

- A point-by-point response to the referees' comments, with a detailed description of the changes made (as a word file). All referees' concerns must be fully addressed and their suggestions taken on board. When preparing your letter of response to the

referees' comments, please bear in mind that this will form part of the Review Process File and will therefore be available online to the community. Please note that you have the possibility to opt out of the transparent process at any stage prior to publication by letting the editorial office know (contact@embojournal.org); if you do opt out, the Review Process File link will point to the following statement: "No Review Process File is available with this article, as the authors have chosen not to make the review process public in this case.". For more details on our Transparent Editorial Process, please visit our website: https://www.embopress.org/page/journal/14602075/authorguide#transparentprocess

- Expanded View (EV) files (replacing Supplementary Information) that are collapsible/expandable online. A maximum of 5 EV Figures can be typeset. EV Figures should be cited as "Figure EV1, Figure EV2" etc. in the text, and their respective legends should be included in the manuscript file after the legends of regular figures. See detailed instructions regarding Expanded View files here:
https://www.embopress.org/page/journal/14602075/authorguide#expandedview

- For the figures that you do NOT wish to display as Expanded View figures, they should be bundled together with their legends in a single PDF file called "Appendix", which should start with a short Table of Contents (including page numbers). Appendix figures should be referred to in the main text as: "Appendix Figure S1, Appendix Figure S2" etc. Please see detailed instructions here: https://www.embopress.org/page/journal/14602075/authorguide#expandedview

- A complete author checklist, which you can download from our author guidelines (https://www.embopress.org/page/journal/14602075/authorguide). Please note that the checklist will also be part of the Review Process File.

2. Please note that no statistics should be calculated if n=2.

3. Please note that a "Data availability" section at the end of Materials and Methods is mandatory. In case you have no data that require deposition in a public database, please state so instead of referring to the database: "Our study includes no data deposited in public repositories." under the heading "Data availability".
See also https://www.embopress.org/page/journal/14602075/authorguide#dataavailability). Please note that the "Data availability" statement is restricted to new primary data that are part of this study.

4. Please check that the title and the abstract of the manuscript are brief, yet explicit, even to non-specialists. The length of the title should not exceed 100 characters, and the abstract should be a single paragraph not exceeding 175 words.

5. Please also note our reference format: https://www.embopress.org/page/journal/14602075/authorguide#referencesformat.

6. At EMBO Press we ask authors to provide source data for the main manuscript figures. Our source data coordinator will contact you to discuss which figure panels we would need source data for and will also provide you with helpful tips on how to upload and organize the files.

7. Please remember: digital image enhancement is acceptable practice, as long as it accurately represents the original data and conforms to community standards. If a figure has been subjected to significant electronic manipulation, this must be noted in the figure legend or in the "Materials and Methods" section. The editors reserve the right to request original versions of figures and the original images that were used to assemble the figure.

8. Our journal encourages inclusion of data citations in the reference list to directly cite datasets that were obtained from public databases. Data citations in the article text are distinct from normal bibliographical citations and should directly link to the database records from which the data can be accessed. In the main text, data citations are formatted as follows: "Data ref: Smith et al, 2001" or "Data ref: NCBI Sequence Read Archive PRJNA342805, 2017". In the Reference list, data citations must be labeled with "[DATASET]". A data reference must provide the database name, accession number/identifiers, and a resolvable link to the landing page from which the data can be accessed at the end of the reference. Further instructions are available at: https://www.embopress.org/page/journal/14602075/authorguide#referencesformat.

9. We request authors to consider both actual and perceived competing interests. Please review our policy (https://www.embopress.org/page/journal/14602075/authorguide#conflictsofinterest) and update your competing interests statement if necessary. Please name this section 'Disclosure and competing interests statement' and place it after the Acknowledgements section.

10. Please note that all corresponding authors are required to provide an ORCID ID upon submission of a revised manuscript (https://orcid.org/). Please find instructions on how to link your ORCID ID to your account in our manuscript tracking system in our Author guidelines
(https://www.embopress.org/page/journal/14602075/authorguide#authorshipguidelines).

11. We use CRediT to specify the contributions of each author in the journal submission system. CRediT replaces the author contribution section, which should be removed from the manuscript. Please use the free text box to provide more detailed

descriptions. See also guide to authors:
https://www.embopress.org/page/journal/14602075/authorguide#authorshipguidelines.

12. Further information is available in our Guide For Authors: https://www.embopress.org/page/journal/14602075/authorguide

13. We would also welcome the submission of cover suggestions or motifs to be used by our Graphics Illustrator in designing a cover.

14. Please use the link below to submit your revision:
https://emboj.msubmit.net/cgi-bin/main.plex

Referee #1:

Jadiya P. and colleagues investigated the contribution of mitochondrial calcium uniporter (MCU) in a mouse model of Alzheimer's disease (AD). To do so, Cre-mediated recombination of a floxed Mcu allele was specifically achieved in the forebrain (including CA1 pyramidal cell layer of the hippocampus) of 3xTg-AD transgenic mice expressing a Presenilin 1 (Psen1, M146V knockin) mutation and two mutant human transgenes for amyloid precursor protein (APPswe) and microtubule associated protein tau (MAPT, P301L). The authors claim that genetic inhibition of Mcu corrects mitochondrial calcium signaling and, as a consequence, aberrant mouse behaviours. Furthermore, the authors show that MCU loss reduces soluble and insoluble Abeta species in 15-months-old mice. The reduced amyloidosis is associated with a lower tau pathology, diminished neuroinflammation and higher expression of synaptic markers. Of note, genetic manipulation of Mcu attenuates mitochondrial ROS in vivo and in vitro. In summary, the authors propose MCU as a new molecular target for AD.

Although it was previously suggested that mitochondrial calcium uptake could play a role in amyloidosis, the newly submitted evidence conclusively describes that MCU inhibition may ameliorate cognitive performance in mice. Thus, the ms may attract the interest of the scientific community and could hold translational aspects helpful for the advance of new interventions against AD. While I agree that these data are of interest, I am not fully convinced about the proposed mechanisms. To improve this descriptive manuscript and provide more mechanistic evidence, I would kindly suggest the authors to consider the following comments.

1. At page 6 the authors wrote: "No significant changes in the expression of other mitochondrial calcium uniporter complex-associated components were observed". However, this is incorrect, as Supplementary figure S1 shows reduction of MCUB, MICU1, MICU3 and NCLX in the cortex of 12-months-old 3xTg-AD mice. Could the authors explain these changes and their biological meaning? As MICU1 and MICU3 regulates MCU-dependent calcium uptake, since they are downregulated in the transgenic AD model, could it be that MICU1 and MICU3 deficiency causes aberrant mitochondrial calcium update? If so, what would happen if MICU1 and/or MICU3 is overexpressed in 3xTg-AD and 3xTg-AD; MCU KO mice? Please, see Singh R et al, 2022 and Ashrafi G. et al, 2020.

2. The authors suggest that neuronal MCU loss may diminish Abeta levels in transgenic mice, possibly by direct or indirect modulation of the gamma-secretase pathway. At the current stage, these are observations and do not show any causative role. It would be important that the authors provide more solid evidence supporting their claims. For example, can they experimentally determine the molecular mechanisms by which MCU inhibition modulates gamma-secretase pathway?

3. The authors suggest that MCU inhibition reduces amyloidosis and tau pathology in 3xTg-AD mice, hence ameliorating behaviour defects. What is the mechanism leading to a reduced tau phosphorylation? Could the authors identify the kinases/phosphatases that are differentially regulated in MCU deficient animals?

4. An abundant literature reported that loss of dendritic spines causes neuronal hyperactivity. In AD models, amyloidosis shits the population of dendritic spines, such as immature filopodia-like structures and more mature mushroom-like spines. Can MCU inhibition ameliorate the loss of dendritic spines? Which dendritic spines are more susceptible to MCU-driven aberrant calcium signaling? If dendritic spines are indeed altered, can MCU inhibition correct neuronal firing defects?

5. Does MCU inhibition influence neuronal death in 3xTg-AD mice?

5. The authors provided very convincing in vivo data. In figure 5, they used a neuroblastoma cell line in which MCU was knockdown. In my opinion, this dataset can be removed, as it does not provide so much more of what shown in vivo.

Additional minor comments:
-The title is a bit too ambitious. I would recommend to tone down the message. The authors may consider: Genetic inhibition of

neuronal mitochondrial calcium uptake ameliorates amyloidosis in mice.
- Behavioural experiments in Figure 1: could the authors confirm whether the same mice were tested at different time points or new cohorts were used? This would influence the statistical analysis that needs to be employed.
- Page 8: the authors wrote "... whereas alpha-secretase (ADAM10) expression was reduced (Figure 2H; S2C-I)". I do not see Figure 2H. Please, adjust the text or figures accordingly.

Referee #2:

This study reports that genetic ablation of the mitochondrial Ca2+ uniporter (MCU) in neurons of the frontal cortex and hippocampus reduces behavioral and pathological alterations in a mouse model of Alzheimer's diseases (AD). By crossing MCU-floxed, CamK2a-Cre, and 3xTg-AD mice the authors show that neuronal-restricted MCU deletion prevents tau and amyloid neuropathology as well as cognitive decline in a triple transgenic mouse model of familial AD. The mitochondrial Ca2+ uniporter is the pore component of a multiprotein complex comprising MICU1, 2, and 3, MCUB, and EMRE. Convincing biochemical evidence show near complete loss of MCU but not of its associated components in MCU-deficient 3xTg-AD mice. Loss of Ca2+ uptake in MCU-deficient mitochondria is also convincingly documented. The behavioral data as well as the biochemical and histochemical evidence that MCU ablation prevents disease progression are solid and the results clear-cut. The mouse studies are complemented with experiments in N2a neuroblastoma cells bearing the Swedish APP mutation, that essentially reproduce the effect at the cellular level with MCU silencing. Overall, the results convincingly show that memory loss, amyloid accumulation, and tau phosphorylation are reduced by the neuronal deletion of MCU in a robust mouse model of AD. Together with earlier report that AAV-mediated MCU overexpression increases neuronal cell death and gliosis in the mouse cortex, these data make a strong case for a causal role of excessive mitochondrial Ca2+ in the pathogenesis of AD. I am quite enthusiastic about this paper but would like the authors to address the following queries to clarify potential ambiguities and provide additional mechanistic insight:

1. Using a similar strategy, the authors recently showed that neuronal loss of the mitochondrial Na/Ca2+ exchanger NCLX increases mitochondrial Ca2+ overload and promotes cognitive decline, amyloid deposition, and tau pathology in mice {PMID: 36936788 }. Surprisingly, this study is not in the reference list. This study should be cited and the similarities and differences between the two complementary animal models discussed in detail.

2. The reduced amyloid levels in 3xTg-AD-Mcu-floxed mice are attributed to a modulation of the gamma secretase pathway based on the reduced levels of secretase components in WB. Since expression levels do not correlate with enzymatic activity, additional evidence is required to establish that the observed effects result from a decrease in amyloid production rather than an increase of its clearance. Additional western blots for other APP processing fragments (C99, AICD, sAPPa; sAPPb) would be useful here.

3. The experiments in Figure 5 do not provide much mechanistic insight and basically replicates the in vivo findings in a cellular model. The recordings of mitochondrial Ca2+ uptake and of the free Ca2+ concentration within the mitochondrial matrix are very indirect. More direct measurements with mitochondria-targeted genetically encoded Ca2+ indicators are required to convincingly show that the basal matrix Ca2+ concentration is impacted by the Swedish mutation and that this change is reversed by MCU silencing. In its present state this figure does not add much to the study and should be removed.

4. The ultrastructural analysis of mitochondria (Fig. S4) fails to document two key parameters: ER-mitochondria contact sites and cristae density. I believe that these parameters would nicely complement the quantification of mitochondrial morphology, because 1) alterations in ER-Mito interface have been reported in several studies to be causally related to AD pathology and 2) cristae density directly correlates with bioenergetics. The authors only need to reanalyze their existing ultrastructural data to provide this complementary dataset.

p.3. The last sentence of the first paragraph seems truncated and would read better ending with "... as a causative event in AD".

p. 8, "Increased levels of soluble and insoluble Aβ1-40 and Aβ1-42 peptides were found in both the cortex and hippocampus of 3xTg-AD mice." This conclusion is not supported by data as the levels of soluble amyloid levels in controls mice are not shown.

p.9, last sentence of first paragraph- These results suggest that

p. 12, line 6: redox status is measured in cell lines in this Figure, not in 3xTg-AD mice. Please correct.

Referee #3:

The manuscript by Jadiya and colleagues aims to investigate the causal relationship between mitochondrial calcium (mCa2+) dysregulation and the pathogenesis of Alzheimer's disease (AD). Specifically, the authors investigated whether increased

mCa2+ uptake contributes to the development of AD. To achieve their goal, they generated 3xTg-AD mutant mice carrying a neuronal-specific deletion of the mitochondrial calcium uniporter (Mcu). These mice showed an amelioration of cognitive decline compared to age-matched controls, as evidenced by behavioral tests such as the Y-maze and fear conditioning. No changes in locomotor activity were observed between experimental and control groups. Furthermore, mice with neuron- specific ablation of Mcu showed decreased levels of soluble and insoluble Aβ 1-40/1-42 peptides in both the cortex and hippocampus compared to controls. In addition, neuron-specific deletion of Mcu expression rescued the increased expression of the γ-secretase complex-associated proteins presenilin 1 (PS-1) and nicastrin (NCT) to near control levels. Similarly, neuron-specific deletion of Mcu reduced levels of insoluble tau in the cortex of 15-month-old mice as well as tau phosphorylation specifically in pyramidal neurons in the CA1 region of the brain. In addition, the levels of post-synaptic density protein (PSD-95) and synaptophysin (SYP) in AD mice were corrected to wild-type levels after neuron-specific deletion of Mcu. Furthermore, GFAP levels were reduced in mice lacking Mcu in neurons, but Iba1 expression remained unchanged, suggesting a reduction in astrocyte immunoreactivity without an effect on microglial activation. It appears that neuron-specific deletion of Mcu has no effect on the number and area of mitochondria. A neuroblastoma cell line with a Swedish mutation in APP (APPswe) showed decreased levels of mtCU regulators, resulting in increased Ca2+ uptake. Targeting Mcu in APPswe cells increased mtCU levels and consequently reduced Ca2+ in the mitochondrial matrix. Under these conditions, mitochondrial damage, as evidenced by mitochondrial membrane potential, membrane rupture and increased mitochondrial ROS production was ameliorated.

Major concerns
This manuscript suggests a causative relationship between Mcu-dependent mCa2+ uptake and AD pathology. A growing body of evidence already links the pathogenesis of AD with dysregulation of neuronal calcium signaling (Ryan et al.,2020, Int J Mol Sci. 21(23): 9153; Jung et al., Front. Cell Dev. Biol., 18; Hedskog et al., 2013, PNAs 110(19): 7916-7921). In this respect, the results presented here support a role for MCU in AD pathology. However, the study in its current form is rather descriptive. In fact, it does not provide a real mechanistic insight into how MCU and the increased Ca2+ uptake contribute to AD. For example, previous studies have implicated MCU in presynaptic Ca2+ homeostasis and neurotransmitter release through a mechanism that relies on LKB1 (Kwon et al., PLOS Biology, doi: 10.1371/journal.pbio.1002516J). More importantly, the requirement of MCU for Aβ-induced mitochondrial Ca2+ elevation was recently documented in the APP/PS1 Tg mouse model of AD in vivo (Calvo-Rodriguez et al. 2020, Nat Commun 11, 2146).

Given that AD mice deficient in neuronal MCU have reduced levels of insoluble Aβ peptides and reduced tau phosphorylation compared to age-matched controls, it is reasonable to examine whether autophagy plays a role in the clearance of such aggregates that are hallmarks of AD pathology. Similarly, the authors claim that MCU deletion prevents mitochondrial dysfunction in the neuroblastoma cell line APPswe. To strengthen their finding, they should test additional parameters of mitochondrial function such as ATP production and oxygen consumption rates. They can extend these experiments using mitochondrial isolates from the AD mouse model after MCU deletion and their age-matched controls.

The fact that AD is associated with mitochondrial dysfunction and defective mitophagy (Kerr, J. S. et al., 2017; Trends Neurosci. 40, 151-166; Lustbader, J. W. et al. 2004, Science 304, 448-452; Fang et al., 2019, Nat. Neuroscience, vol. 22, 401-412) and the finding that loss of mCa2+ uptake prevents AD-associated mitochondrial dysfunction, suggests that the authors should investigate the possible crosstalk between MCU-dependent Ca2+ uptake and mitophagy in AD.

Other comments
Some controls are missing in several experiments. For example, quantification of the integrated optical density area of the AT8 and AT180 in CamK2a-Cre mice in Figures 3G and H. Similarly, the data for CamK2a-Cre mice are missing in Figures 4A-4F.

Some experimental issues need to be addressed. For example, the authors do not find microglial activation in the cortex of 3xTg-AD x McuFl/Fl x Camk2a-Cre mice. Since all other parameters of mitochondrial function were tested in both the cortex and the hippocampus, the authors should also examine whether microglia are activated in the hippocampus.

Page 7, Fig.1G: The authors state that "loss of neuronal MCU significantly improved age-associated impairments". From Fig.1G, one could say that there is an improvement on AD-associated impairments, rather age.

Page 7, end of the paragraph: Could you please provide the relevant WB for loss of MCU in wild type mice (Mcufl/fl x Camk2a-Cre)? The authors discuss that loss of MCU in wild type animals did not result in cognitive impairment in Y-maze testing but do not provide the relevant WB.

Page 9: The authors stated that "3xTg-AD x Mcufl/fl x Camk2a-Cre mice displayed a significant reduction in insoluble tau". However, this doesn't seem to be the case in the WB of the 2nd cohort (Fig.S3A). There, it seems as it is reduced but there is no quantification. How do these results, if quantified, affect the overall finding?

Page 10: Regarding the TEM imaging of mitochondria, could you please comment on the fact that there no changes in mitochondria? Mitochondria dysfunction, as the authors mentioned, is commonly found in AD models so I am a bit surprise there is no difference between wild and AD animals. Perhaps not all factors but for instance the circularity or the density.

Page 12: Is the sentence "To examine redox status in 3xTg-AD mice" a typo? At this stage, authors examine ROS levels in 2A

and APPswe cells.

Figure 3A, E and S3A: The normalized quantifications of PHF-13 in 1E seem not to correspond to the density of the bands of the two cohorts. At least two values of the 3xTg-AD x Mcufl/fl x Camk2a-Cre mice appear below the Camk2a-Cre control in 1E, which does not seem to be the case in any blot. Moreover. there is some apparently non-specific signal in the PHF-13 blot of the second cohort 3xTg-AD x Mcufl/fl x Camk2a-Cre samples (S3A).

Figure 3F-H: Authors should also validate the rest of the WB results (AT270 and PHF-13) with the respective immunohistochemical assay as performed for AT8 and AT180 if the same or similar antibodies allow such an application.

Figure S4 legend: Include mitochondrial number per area description in the legend (S4F) and correct the following letters from F to L.

As authors mention, predisposition towards increased mCa2+ uptake and overload due to decreased expression of the mtCU regulators MICU1, MICU2 and MCUB is possible in APPswe cells. Thus, the results derived from these cells (Ca2+ uptake, plasma membrane rapture and cellular viability) have to be carefully interpreted. This needs to be explicitly mentioned in the discussion as well.

While membrane rapture is decreased in Mcu knockdown APPswe cells in almost all tested conditions (Figure 5J, K), cellular viability is not enhanced upon Mcu knockdown at APPswe cells as authors claim (Figure S5F-G). Only in one case (2μM ionomycin), the viability is significantly enhanced (where the point distribution seems not to support it; please, double-check the statistics). The authors should reconsider their respective conclusions.

In general, a substantial amount of data is derived from WBs and associated densitometry. However, in some cases, the blot quality is not optimal (bubbles, progressive fainting, background, etc., examples: 1B-MICU3, 3A-HT7/AT180, 4G-PSD-95, 4H-GFAP, 5B-CV-Sα, S2C-PS1/NCT, S3A-PHF-13) which most likely affects the densitometric analysis, as well.

Figure 1 panels: Perhaps, change the order of plots I, J and K in figure 1 to match the order commented in the main text (p7, towards the end of the section). For instance, Fig.1I is commented in the manuscript after J and K.

I am sorry, but I have not been able to find Fig. 2H.

Please, mention Figure 5A in the text.

Response to Reviewers' Critique – Jadiya et al. EMBOJ-2023-115858

We extend our sincere gratitude to the editor and reviewers for their invaluable comments and suggestions. We have addressed all concerns, and we believe it has significantly improved the quality and impact of the manuscript. Please find a detailed point-by-point response to the reviewers' comments.

Referee #1

1. At page 6 the authors wrote: "No significant changes in the expression of other mitochondrial calcium uniporter complex-associated components were observed". However, this is incorrect, as Supplementary figure S1 shows reduction of MCUB, MICU1, MICU3 and NCLX in the cortex of 12-months-old 3xTg-AD mice. Could the authors explain these changes and their biological meaning? As MICU1 and MICU3 regulates MCU-dependent calcium uptake, since they are downregulated in the transgenic AD model, could it be that MICU1 and MICU3 deficiency causes aberrant mitochondrial calcium update? If so, what would happen if MICU1 and/or MICU3 is overexpressed in 3xTg-AD and 3xTg-AD; MCU KO mice? Please, see Singh R et al, 2022 and Ashrafi G. et al, 2020.

We appreciate the reviewer's keen observation and valuable feedback. We clarified our interpretation of these data, including a detailed explanation of the changes in expression that you highlighted. Western blot analysis revealed loss of MCU protein in the frontal cortex of 2-month-old 3xTg-AD x *Mcu*$^{fl/fl}$ x Camk2a-Cre mice and a corresponding reduction in 12-month-old mice compared to CamK2a-Cre and 3xTg-AD mice. No significant changes were observed in the expression of other mitochondrial calcium uniporter channel complex (mtCU)-associated components in the 3xTg-AD x *Mcu*$^{fl/fl}$ x Camk2a-Cre mice compared to 3xTg-AD x Camk2a-Cre mice. However, in 12-month-old 3xTg-AD and 3xTg-AD x *Mcu*$^{fl/fl}$ x Camk2a-Cre mice, there was a reduction in MCUB, MICU1, MICU3, and NCLX expression compared to Camk2a-Cre mice. This decrease in the expression of key mitochondrial calcium uniporter (mtCU) regulators likely contributes to elevated $_{m}Ca^{2+}$ levels, which are observed during AD progression (Jadiya et. al, *Nature Communication*, 2019; Calvo-Rodriguez et.al, *J Alzheimer's Dis*, 2020; Jadiya et.al, *iScience*, 2023). Previous studies have shown that neuron specific homozygous MICU1 deletion in mice leads to altered neuronal Ca^{2+} homeostasis and progressive motor and cognitive dysfunction (Singh, *Science Advances*, 2022). As MICU1 and MICU3 regulate MCU-dependent Ca^{2+} uptake (Ashrafi, *Frontiers in Cell Neuroscience*, 2020; Singh, *Science Advances*, 2022), their downregulation in the transgenic AD model suggests that MICU1 and MICU3 deficiency may cause aberrant $_{m}Ca^{2+}$ uptake. It is possible that rescued expression of MICU1 and MICU3 might prevent $_{m}Ca^{2+}$ dysregulation in AD mice, which can be examined in future studies. We have carefully rephrased these observations for clarity. Additionally, we have provided a thorough explanation of these changes and their biological significance in 3xTg-AD mice in the revised manuscript to address the reviewer's concerns.

2. The authors suggest that neuronal MCU loss may diminish A-beta levels in transgenic mice, possibly by direct or indirect modulation of the gamma-secretase pathway. At the current stage, these are observations and do not show any causative role. It would be important that the authors provide more solid evidence supporting their claims. For example, can they experimentally determine the molecular mechanisms by which MCU inhibition modulates gamma-secretase pathway?

We appreciate the reviewer's thoughtful feedback and understand the need for more concrete evidence regarding the suggested causative role of neuronal MCU loss in diminishing A-beta levels. After performing follow up experiments, we now hypothesize that MCU ablation reduces amyloid aggregation in neurons by promoting clearance via autophagy rather than by directly affecting the gamma-secretase pathway (**Figure 5 J-O, S5 J-S**). Autophagic capacity can be impaired by accumulation of damaged mitochondria thus creating a potential bottleneck in the clearance of damaged material (Nichenko et al., 2020; Call & Nichenko, 2020). Autophagic flux has been previously reported to be reduced by mitochondrial dysfunction and excessive ROS generation (reviewed in Vicente Roca-Agujetas, 2019). All of these are partially prevented by MCU loss (**Figure 4J-L, 5G, S5G**) thus directly linking inhibition of mCa2+ uptake in neurons with protection against AD

pathology. Please, see detailed description in the Results for additional experiments performed, as well as new figures below.

*“We further addressed molecular mechanisms behind protection against amyloid aggregation and AD pathology exerted by MCU loss in a cell model (N2a APPswe cells). We found that in contrast to our in vivo model, MCU ablation did not reduce expression of gamma-secretase components, Nicastrin (NCT), Presenelin-1, or APH in N2a APPswe cells (**Figure S5 P-S**). However, MCU loss did reduce Aβ accumulation 24 h after the addition of HiLyte™ Fluor 488-labeled Abeta to the cells (**Figure 5 J-K**) and protein aggregation both in the absence and presence of APPswe measured by a fluorescent dye Proteostat (**Figure 5 L-M**) indicating a possible upregulation of clearance. Indeed, we further found that APPswe reduces basal autophagy in N2a neurons and this is rescued by MCU ablation, measured as percentage of autolysosomes to the total number of LC3 positive structures in the resting condition using tandem fluorescent-tagged LC3 (mRFP-EGFP-LC3) (**Figure 5 N-O, S5J-L**). Autophagic capacity can be impaired by the accumulation of damaged mitochondria thus creating a potential bottleneck in the clearance of amyloid and proteotoxins (Nichenko et al., 2020; Call & Nichenko, 2020). It has been also previously reported to be reduced by mitochondrial dysfunction and excessive ROS generation (Vicente Roca-Agujetas, 2019). All of these are partially prevented by MCU loss thus directly linking inhibition of mCa2+ uptake in neurons with protection against AD pathology.”*

Figure 5

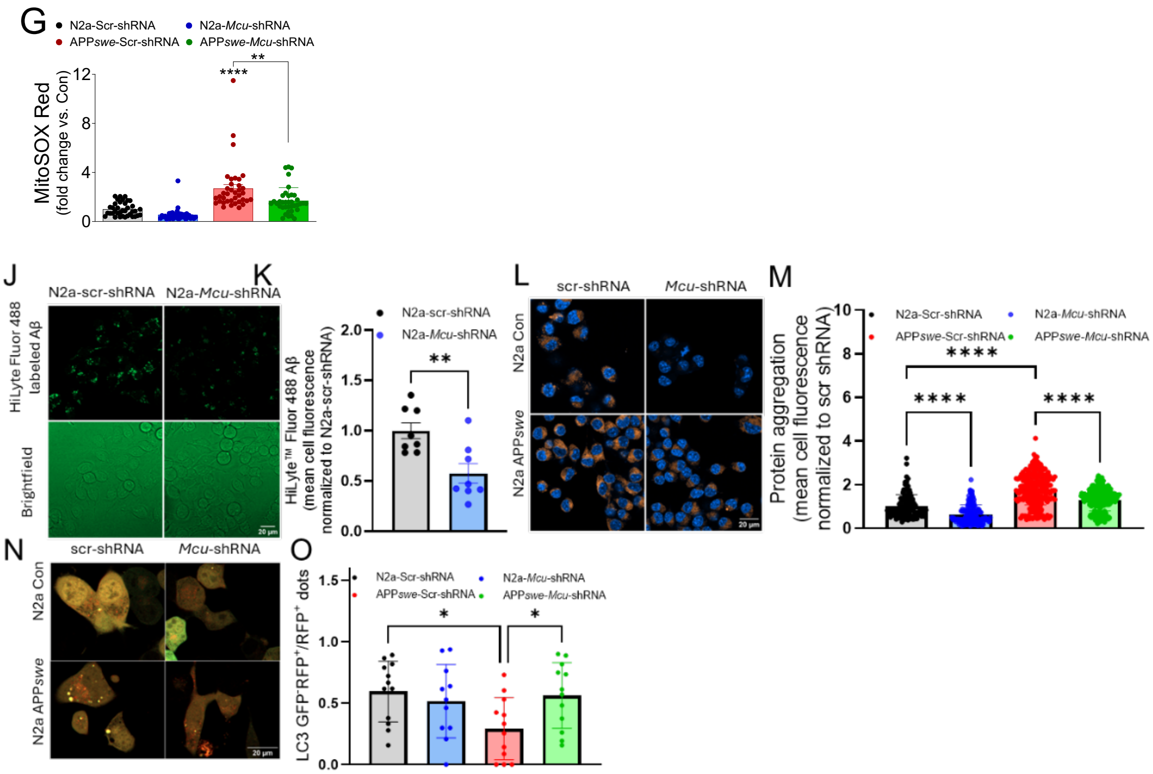

Supplementary Figure 5

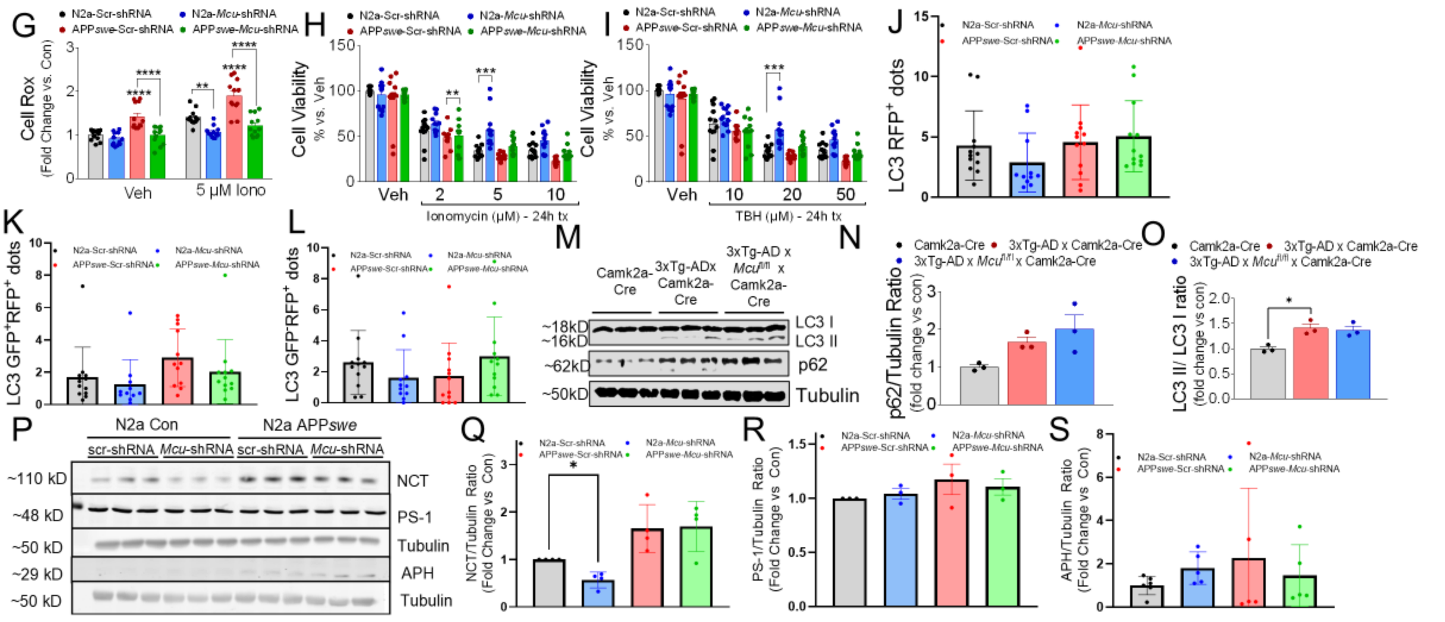

Figure 4

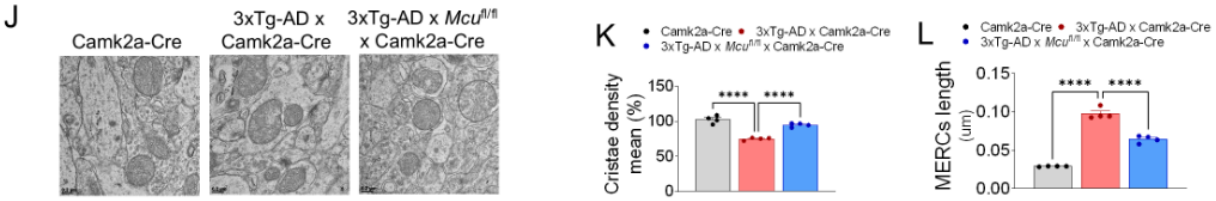

3. The authors suggest that MCU inhibition reduces amyloidosis and tau pathology in 3xTg-AD mice, hence ameliorating behavior defects. What is the mechanism leading to a reduced tau phosphorylation? Could the authors identify the kinases/phosphatases that are differentially regulated in MCU deficient animals?

To address the mechanism underlying reduced tau phosphorylation in MCU-deficient animals, we examined the levels of major tau kinases and phosphatases in protein lysate samples from the frontal cortex of 15-month-old control, 3xTg-AD, and 3xTg-AD x *Mcu*$^{fl/fl}$ x Camk2a-Cre mice. Specifically, we have investigated the expression of key tau kinases, including GSK-3β, CDK5, ERK2, CaMKII, MARK, and RACK1 **(Figure S3 E-K)**. We found that the expression of GSK-3β and CDK5 was significantly increased in 3xTg-AD mice, but this increase was reduced in 3xTg-AD x *Mcu*$^{fl/fl}$ x Camk2a-Cre mice (**Figure S3 E-G**). However, no significant changes were observed in the expression of MAPKs, CaMKII, MARK1, and ERK2 (**Figure S3 E,H-K**).

Increased GSK-3β expression has been associated with reduced neurogenesis and increased neuronal cell death in the hippocampus of AD patients (Martin et.al, Molecular Psychiatry 2013). CDK5, a serine/threonine kinase, plays a role in neuronal survival and cognitive function by regulating synaptic morphology in the central nervous system. The CDK5/p25 complex, which is significantly elevated in AD patients, phosphorylates APP, Thr-252 of β-secretase, and STAT3, thereby exacerbating AD pathology (Iijima et.al, Journal of Neurochemistry, 2000 and Cruz et.al, Neuron 2003)

We also examined the expression of major tau-related protein phosphatases, including Protein Phosphatase 2A (PP2A), Protein Phosphatase 1 (PP1), Protein Phosphatase 5 (PP5), and Calcineurin (PP2B). Our analysis revealed no significant changes in the levels of these phosphatases (**Figure S3 L-P**) between 3xTg-AD and 3xTg-AD x *Mcu*$^{fl/fl}$ x Camk2a-Cre mice. These findings suggest that the impact of MCU deletion on neuronal functions and cognitive performance may be mediated through downstream effects on kinases rather than phosphatases. This indicates that MCU's role in reducing tau pathology is related to its regulation of kinase activity rather than phosphatase activity. Overall, our comprehensive analysis highlights that while tau-related kinases show differential regulation, tau-related phosphatases remain unchanged.

Supp. Fig.3

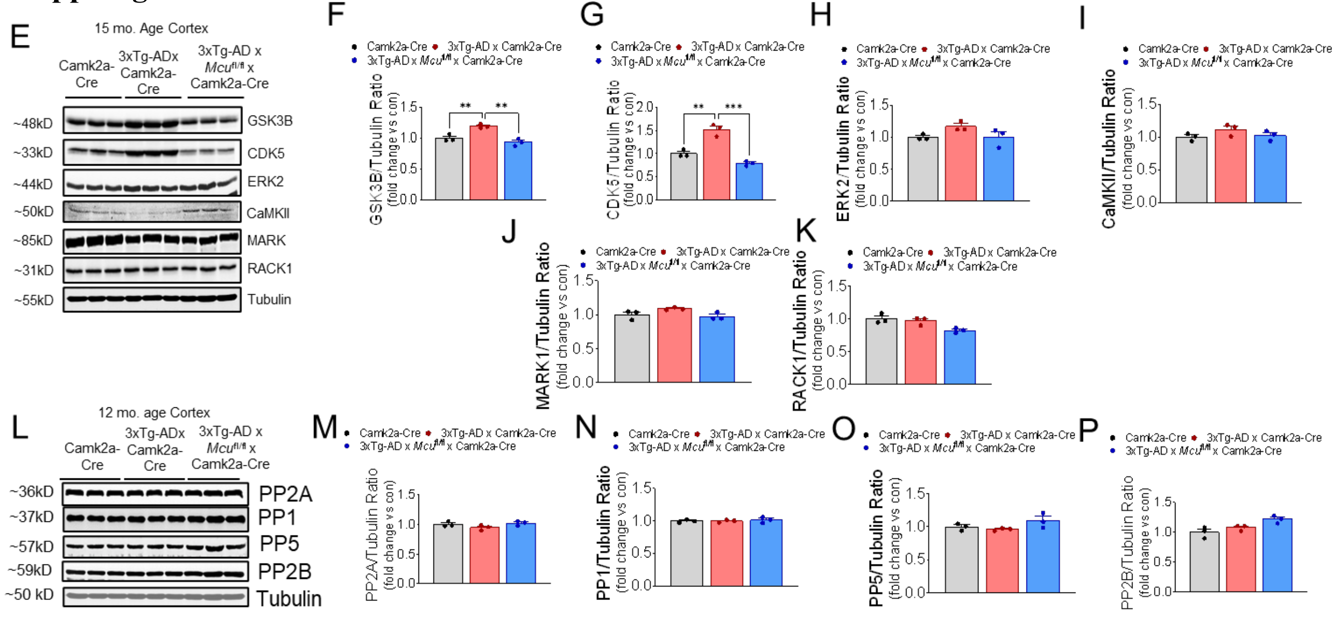

4. An abundant literature reported that loss of dendritic spines causes neuronal hyperactivity. In AD models, amyloidosis shits the population of dendritic spines, such as immature filopodia-like structures and more mature mushroom-like spines. Can MCU inhibition ameliorate the loss of dendritic spines? Which dendritic spines are more susceptible to MCU-driven aberrant calcium signaling? If dendritic spines are indeed altered, can MCU inhibition correct neuronal firing defects?

Thank you for your input. Investigating the impact of *Mcu* on dendritic spines and memory formation is indeed an intriguing avenue. However, we would like to draw the reviewer's attention to a recent study conducted by Cai et al. *Acta Biochim Biophys Sin*, 2022. In Figure 2F and 2G of their publication, they demonstrate that 3xTg-AD mice exhibit a reduction in dendritic spines, and this loss is ameliorated by MCU knockout using shRNA. Consequently, it is reasonable to anticipate that *MCU* knockout in our case might yield similar experimental outcomes. While we acknowledge the relevance of this aspect and added this information to the Introduction (page 6, paragraph 1) and Results (page 13, paragraph 2), a comprehensive exploration of MCU's impact on dendritic spines and its underlying molecular mechanisms requires a more in-depth investigation. Regrettably, such an extensive study falls beyond the scope of our current work. We appreciate the reviewer's consideration and are open to further discussion.

5. Does MCU inhibition influence neuronal death in 3xTg-AD mice?

We addressed this concern by examining the effect of two stressors in our cellular AD model, N2a APPswe cells. MCU ablation reduced membrane rupture, a characteristic of necrotic cell death, in the

presence of both, Ca^{2+} overload (exposure to ionomycin) and excess of ROS (exposure to *tert*-butyl hydroperoxide) (**Figure 5H-I**).

Figure 5

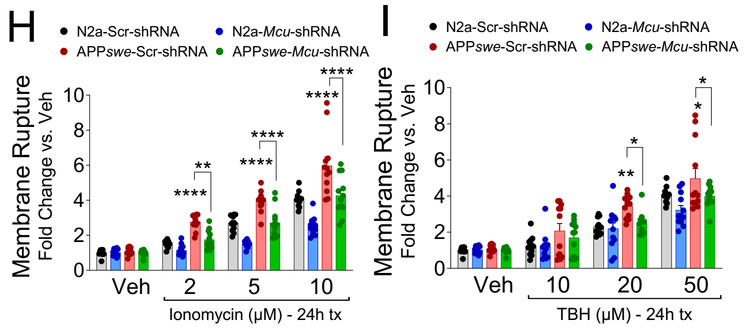

6. The authors provided very convincing in vivo data. In figure 5, they used a neuroblastoma cell line in which MCU was knockdown. In my opinion, this dataset can be removed, as it does not provide so much more of what shown in vivo.

We performed follow-up experiments in this cell line using overexpression of APPswe. This reductionist model helped us link the loss of MCU and reduced mitochondrial dysfunction and redox imbalance to augment the clearance the clearance of damaged organelles. Rescuing of autophagic capacity, seems to alleviate the bottleneck in the clearance of aggregated proteins and protects neurons against AD pathology (see our detailed response above). We believe this justifies leaving this figure in the main manuscript.

Additional minor comments:

7. The title is a bit too ambitious. I would recommend to tone down the message. The authors may consider: Genetic inhibition of neuronal mitochondrial calcium uptake ameliorates amyloidosis in mice.

We have revised the title as suggested to lessen the claims.

8. Behavioral experiments in Figure 1: could the authors confirm whether the same mice were tested at different time points or new cohorts were used? This would influence the statistical analysis that needs to be employed.

In our study, we used the same cohort of mice for the behavioral experiments shown in Figure 1, testing them at multiple time points. This longitudinal approach allows us to track changes within the same subjects over time, providing valuable insights into the progression of behavioral changes. The use of the same mice across different time points impacts the statistical analysis as it introduces intra-subject correlations. To account for this, we employed repeated measures ANOVA which is specifically designed for analyzing data from repeated measurements on the same subjects. This approach ensures that the statistical tests properly account for the within-subject correlation and provides more accurate results. We have included details about this analysis in the methods section of our manuscript to clarify the statistical approach used.

9. Page 8: the authors wrote "... whereas alpha-secretase (ADAM10) expression was reduced (Figure 2H; S2C-I)". I do not see Figure 2H. Please, adjust the text or figures accordingly.

We apologize for the oversight. We have corrected this in the revised manuscript.

Referee #2

This study reports that genetic ablation of the mitochondrial Ca2+ uniporter (MCU) in neurons of the frontal cortex and hippocampus reduces behavioral and pathological alterations in a mouse model of Alzheimer's diseases (AD). By crossing MCU-floxed, CamK2a-Cre, and 3xTg-AD mice the authors show that neuronal-restricted MCU deletion prevents tau and amyloid neuropathology as well as cognitive decline in a triple transgenic mouse model of familial AD. The mitochondrial Ca2+ uniporter is the pore component of a multiprotein complex comprising MICU1, 2, and 3, MCUB, and EMRE. Convincing biochemical evidence show near complete loss of MCU but not of its associated components in MCU-deficient 3xTg-AD mice. Loss of Ca2+ uptake in MCU-deficient mitochondria is also convincingly documented. The behavioral data as well as the biochemical and histochemical evidence that MCU ablation prevents disease progression are solid and the results clear-cut. The mouse studies are complemented with experiments in N2a neuroblastoma cells bearing the Swedish APP mutation, that essentially reproduce the effect at the cellular level with MCU silencing. Overall, the results convincingly show that memory loss, amyloid accumulation, and tau phosphorylation are reduced by the neuronal deletion of MCU in a robust mouse model of AD. Together with earlier report that AAV-mediated MCU overexpression increases neuronal cell death and gliosis in the mouse cortex, these data make a strong case for a causal role of excessive mitochondrial Ca2+ in the pathogenesis of AD. I am quite enthusiastic about this paper but would like the authors to address the following queries to clarify potential ambiguities and provide additional mechanistic insight:

Thank you for your thoughtful comments and praise of our work. Your insights have strengthened the impact of the manuscript.

1. Using a similar strategy, the authors recently showed that neuronal loss of the mitochondrial Na/Ca2+ exchanger NCLX increases mitochondrial Ca2+ overload and promotes cognitive decline, amyloid deposition, and tau pathology in mice {PMID: 36936788 }. Surprisingly, this study is not in the reference list. This study should be cited and the similarities and differences between the two complementary animal models discussed in detail.

We are embarrassed by this oversight. Our previous work is now cited and has been added to provide a comprehensive discussion on these two complementary animal models.

2. The reduced amyloid levels in 3xTg-AD-Mcu-floxed mice are attributed to a modulation of the gamma secretase pathway based on the reduced levels of secretase components in WB. Since expression levels do not correlate with enzymatic activity, additional evidence is required to establish that the observed effects result from a decrease in amyloid production rather than an increase of its clearance. Additional western blots for other APP processing fragments (C99, AICD, sAPPa; sAPPb) would be useful here.

Thank you for your insightful feedback. We have conducted additional Western blot analyses for APP processing fragments, including sAPPα, sAPPβ, AICD, and C99. Our results show no significant changes in these fragments between 3xTg-AD x *Mcu*$^{fl/fl}$ x Camk2a-Cre and 3xTg-AD mice (see **Figure 2G; S2 D-M**). These findings suggest that the reduction in Aβ levels observed in 3xTg-AD x *Mcu*$^{fl/fl}$ x Camk2a-Cre mice is likely not due to alterations in the cleavage of APP into these fragments. Instead, the decreased Aβ levels may result from changes in gamma secretase activity or other indirect mechanisms affecting Aβ production and/or clearance as discussed above (*Reviewer #1, comment* #2). This implies that MCU deletion influences Aβ levels through pathways other than direct alterations in APP processing. We will continue to examine these mechanisms to clarify the role of MCU in amyloid beta regulation in future studies.

Fig.2

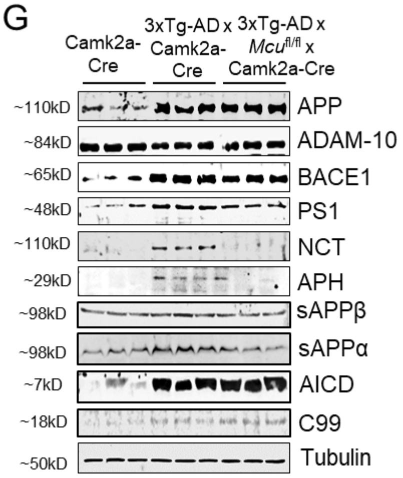

Supp.Fig.2

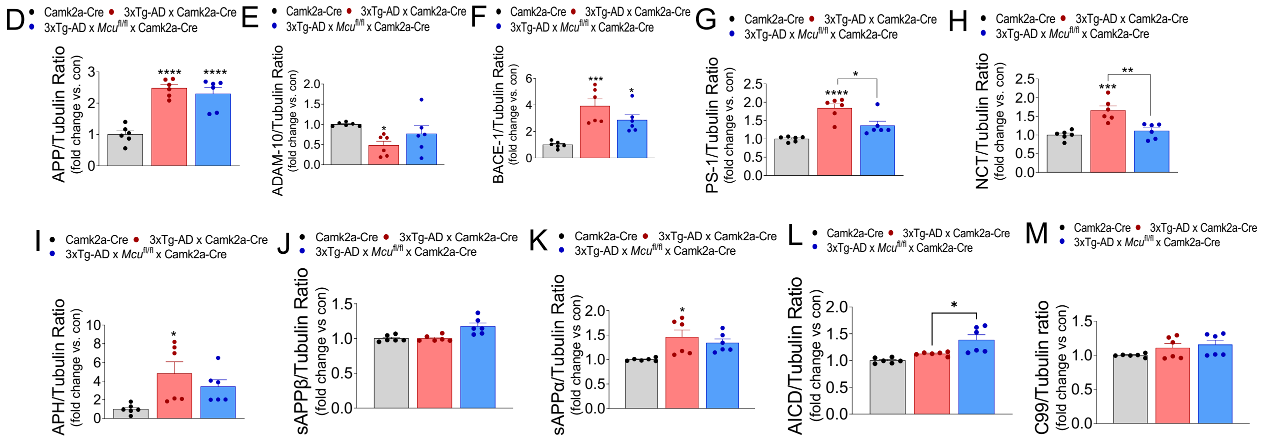

3. The experiments in Figure 5 do not provide much mechanistic insight and basically replicates the in vivo findings in a cellular model. The recordings of mitochondrial Ca2+ uptake and of the free Ca2+ concentration within the mitochondrial matrix are very indirect. More direct measurements with mitochondria-targeted genetically encoded Ca2+ indicators are required to convincingly show that the basal matrix Ca2+ concentration is impacted by the Swedish mutation and that this change is reversed by MCU silencing. In its present state this figure does not add much to the study and should be removed.

We performed follow-up experiments in this AD-model cell line that helped us to link decreased mitochondrial calcium uptake and consequent protection against mitochondrial dysfunction and redox imbalance. We now utilize this reductionist model to provide a molecular mechanism whereby the loss of MCU is cytoprotective in the context of AD.

We now hypothesize that rescuing mitochondrial function reduces the amount of dysfunctional mitochondrial that are targeted for autophagic clearance. Reducing the ‘mitophagy load’ allows for increased clearance of amyloid and other damaged material associated with AD progression. We postulate that preserving mitochondrial function eliminates the bottleneck in autophagic flux and protects neurons against AD pathology (**Figure 5, below**). We believe this justifies leaving this figure in the main section. We should note however that while addressing the second concern we were not able to detect increased matrix free-Ca2+ content in N2a APPswe cells using genetically encoded

ratiometric calcium indicator, mtGEM-GECO1, possibly due to the high affinity of the probe and its saturation within the range of interest (*mtGEM-GECO1 Figure, is provided below for the reviewer*).

Figure 5

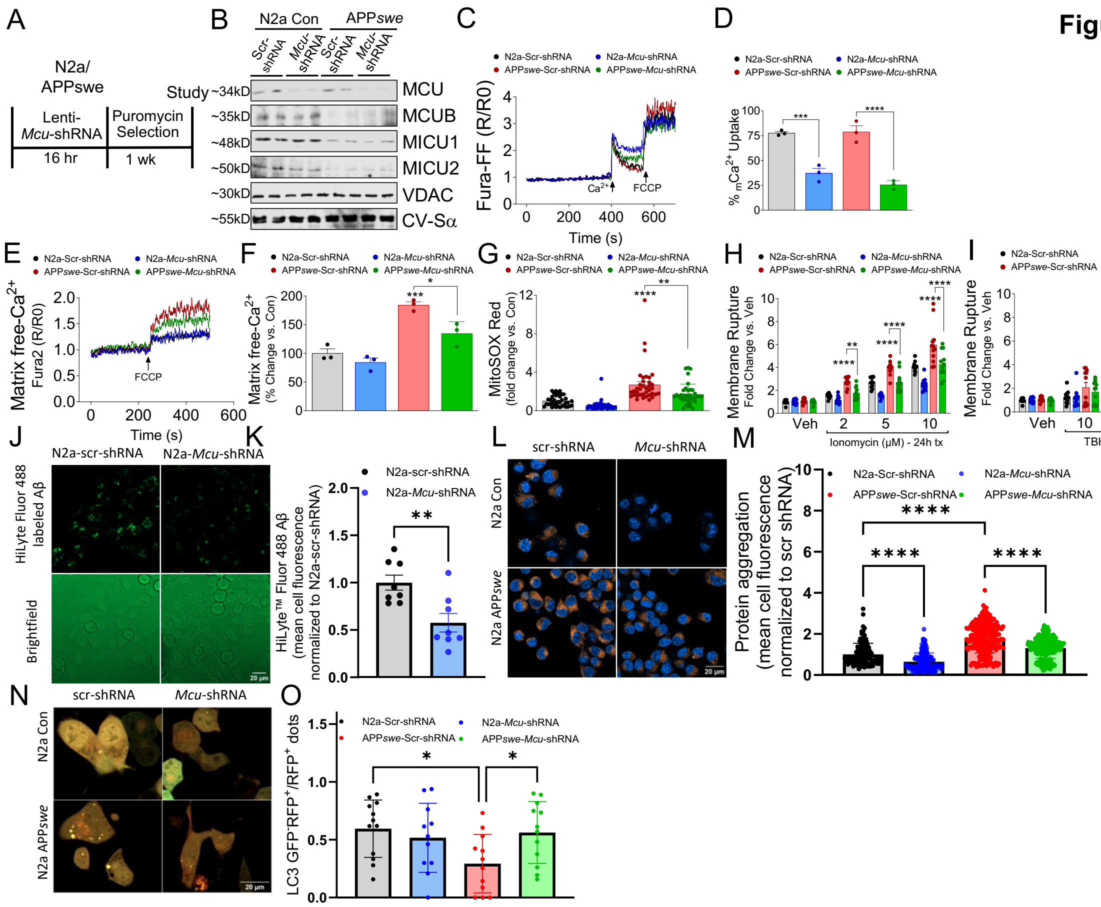

mtGEM-GECO1

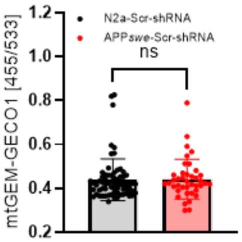

4. The ultrastructural analysis of mitochondria (Fig. S4) fails to document two key parameters: ER-mitochondria contact sites and cristae density. I believe that these parameters would nicely complement the quantification of mitochondrial morphology, because 1) alterations in ER-Mito interface have been reported in several studies to be causally related to AD pathology and 2) cristae density directly correlates with bioenergetics. The authors only need to reanalyze their existing ultrastructural data to provide this complementary dataset.

Thank you for this suggestion. We have reanalyzed the ultrastructural data to specifically include information on ER-mitochondria contact sites and cristae density. We observed significant changes in cristae density and the distance between mitochondria and endoplasmic reticulum contacts (MERC).

Specifically, cristae density was increased in 3xTg-AD x *Mcu*$^{fl/fl}$ x Camk2a-Cre mice compared to 3xTg-AD mice. Additionally, the mean MERC length was reduced in 3xTg-AD x *Mcu*$^{fl/fl}$ x Camk2a-Cre mice compared to 3xTg-AD mice (**Figure 4 J-L)**.

Fig.4

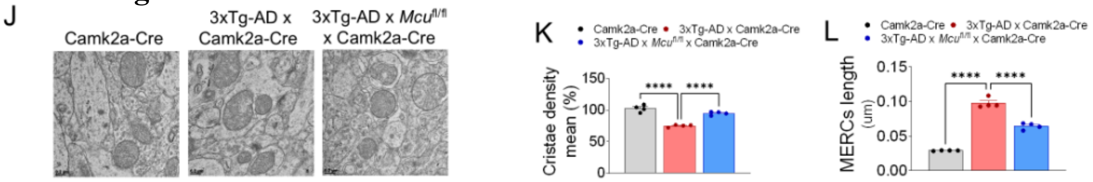

5. p.3. The last sentence of the first paragraph seems truncated and would read better ending with "... as a causative event in AD".

We have revised the sentence as suggested.

6. p. 8, "Increased levels of soluble and insoluble Aβ1-40 and Aβ1-42 peptides were found in both the cortex and hippocampus of 3xTg-AD mice." This conclusion is not supported by data as the levels of soluble amyloid levels in controls mice are not shown.

We have revised the statement to accurately reflect our findings. The updated conclusion now reads: *"The loss of neuronal $_m$Ca^{2+} uptake in AD mice resulted in a significant decrease in both soluble and insoluble Aβ$_{1-40}$ and Aβ$_{1-42}$ levels in the cortex and hippocampus (Figure 2A-D). Additionally, there was a reduction in the Aβ$_{42/40}$ ratio in soluble fractions from the brain cortex of 3xTg-AD x Mcu$^{fl/fl}$ x Camk2a-Cre mice compared to 3xTg-AD mice (Figure S2A).*

7. p.9, last sentence of first paragraph- These results suggest that

We have fixed this.

8. p. 12, line 6: redox status is measured in cell lines in this Figure, not in 3xTg-AD mice. Please correct.

Corrected as requested.

Referee #3

The manuscript by Jadiya and colleagues aims to investigate the causal relationship between mitochondrial calcium (mCa2+) dysregulation and the pathogenesis of Alzheimer's disease (AD). Specifically, the authors investigated whether increased mCa2+ uptake contributes to the development of AD. To achieve their goal, they generated 3xTg-AD mutant mice carrying a neuronal-specific deletion of the mitochondrial calcium uniporter (Mcu). These mice showed an amelioration of cognitive decline compared to age-matched controls, as evidenced by behavioral tests such as the Y-maze and fear conditioning. No changes in locomotor activity were observed between experimental and control groups. Furthermore, mice with neuron- specific ablation of Mcu showed decreased levels of soluble and insoluble Aβ 1-40/1-42 peptides in both the cortex and hippocampus compared to controls. In addition, neuron-specific deletion of Mcu expression rescued the increased expression of the γ-secretase complex-associated proteins presenilin 1 (PS-1) and nicastrin (NCT) to near control levels. Similarly, neuron-specific deletion of Mcu reduced levels of insoluble tau in the cortex of 15-month-old mice as well as tau phosphorylation specifically in pyramidal neurons in the CA1 region of the brain. In addition, the levels of post-synaptic density protein (PSD-95) and synaptophysin (SYP) in AD mice were corrected to wild-type levels after neuron-specific deletion of Mcu. Furthermore, GFAP levels were reduced in mice lacking Mcu in neurons, but Iba1 expression remained unchanged, suggesting a reduction in astrocyte immunoreactivity without an effect on microglial activation. It

appears that neuron-specific deletion of Mcu has no effect on the number and area of mitochondria. A neuroblastoma cell line with a Swedish mutation in APP (APPswe) showed decreased levels of mtCU regulators, resulting in increased Ca2+ uptake. Targeting Mcu in APPswe cells increased mtCU levels and consequently reduced Ca2+ in the mitochondrial matrix. Under these conditions, mitochondrial damage, as evidenced by mitochondrial membrane potential, membrane rupture and increased mitochondrial ROS production was ameliorated.

Thank you for this extensive summary and your helpful comments.

Major concerns

1. This manuscript suggests a causative relationship between Mcu-dependent mCa2+ uptake and AD pathology. A growing body of evidence already links the pathogenesis of AD with dysregulation of neuronal calcium signaling (Ryan et al.,2020, Int J Mol Sci. 21(23): 9153; Jung et al., Front. Cell Dev. Biol., 18; Hedskog et al., 2013, PNAs 110(19): 7916-7921). In this respect, the results presented here support a role for MCU in AD pathology. However, the study in its current form is rather descriptive. In fact, it does not provide a real mechanistic insight into how MCU and the increased Ca2+ uptake contribute to AD. For example, previous studies have implicated MCU in presynaptic Ca2+ homeostasis and neurotransmitter release through a mechanism that relies on LKB1 (Kwon et al., PLOS Biology, doi: 10.1371/journal.pbio.1002516J). More importantly, the requirement of MCU for Aβ-induced mitochondrial Ca2+ elevation was recently documented in the APP/PS1 Tg mouse model of AD in vivo (Calvo-Rodriguez et al. 2020, Nat Commun 11, 2146).

We respectfully disagree that our study is merely descriptive and does not provide mechanistic insight. Though prior studies have suggested a link between mitochondrial calcium overload and AD pathology, the role of mCa2+ uptake via MCU was not addressed and limited by the use of the MCU inhibitor Ru360 that is known to have several off-target effects. The only study examining Mcu knockdown in AD background (Cai H, 2022) suggests some phenotypical improvement in mice but fails to convincingly show a decrease in MCU expression and is lacking important controls, further this study does not provide any mechanistic details including functional validation of MCU loss. Indeed, not a single mitochondrial parameter is experimentally examined and the functionality of the model system is questionable.

Here, we show that MCU ablation reduces mCa2+ uptake, alleviates mitochondrial dysfunction, and redox imbalance both in vivo and vitro thus eliminating a bottleneck in the clearance of damaged cell material and rescuing autophagic capacity, which ultimately results in protection of neurons against amyloidosis and cell death. (**Figure 4 J-L, 5**). Altogether this contributes to mitigating AD pathology in vivo as we have shown that MCU ablation rescues synaptic integrity and behavioral deficit in 3xTg-AD mice. Thus, we provide conclusive evidence using conditional genetic models that mCa2+ uptake contributes to AD pathology.

Fig.4

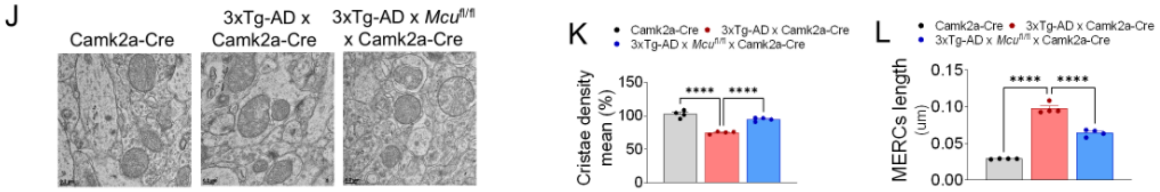

Figure 5

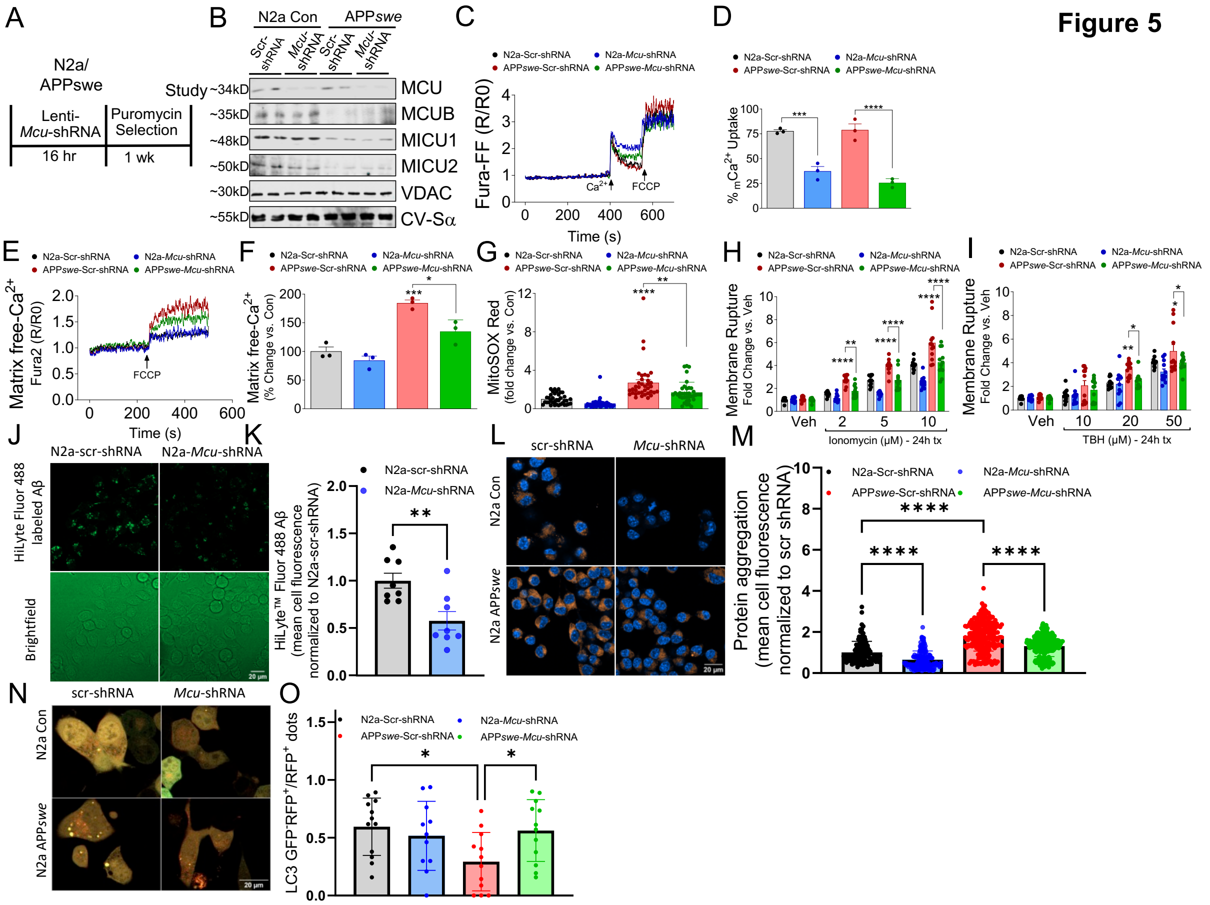

2. Given that AD mice deficient in neuronal MCU have reduced levels of insoluble Aβ peptides and reduced tau phosphorylation compared to age-matched controls, it is reasonable to examine whether autophagy plays a role in the clearance of such aggregates that are hallmarks of AD pathology.

We appreciate the reviewer's thoughtful feedback and addressed this concern both *in vivo* and using a reductionists cell model. While we did not detect altered autophagy in vivo, probably due to difficulty of detection of such changes in heterogenous tissue homogenate that contains numerous non-neuronal cells that were not targeted by our conditional genetic model system (**Figure S5 M-O**), we did find that the loss of MCU rescued the autophagic capacity in the N2a APPswe AD cell model (**Figure 5**). We hypothesize that blocking mitochondrial calcium overload in the context of AD stress reduces mitochondrial dysfunction, excessive ROS generation, and thereby eliminates the bottleneck in the clearance of damaged cell material (i.e., dysfunctional mitochondria) and allows for the enhanced clearance of amyloid and damaged cellular material (Vicente Roca-Agujetas, 2019; Nichenko et al., 2020; Call & Nichenko, 2020). Please see our new results and figures below.

“We analyzed autophagy markers in protein lysates from the frontal cortex of 15-month-old control, 3xTg-AD, and 3xTg-AD x Mcufl/fl x Camk2a-Cre mice (**Figure S5 M-O**). However, we did not detect significant changes in the expression levels of LC3-II/LC3-I or p62, which are typically associated with autophagy activity. This might be due to difficulty of detection of such changes in tissue homogenate that contains also non-neuronal cells. Alternatively, these findings may suggest that the clearance of Aβ in these mice does not occur through the traditional LC3-associated autophagy pathway and rather through alternative mechanisms outside of the well-known autophagy pathways.”

“However, MCU loss did reduce Aβ accumulation 24h after the addition of HiLyte™ Fluor 488-labeled Abeta to the cells (**Figure 5 J-K**) and protein aggregation both in the absence and presence of APPswe measured by a fluorescent dye Proteostat (**Figure 5 L-M**) indicating enhanced amyloid clearance. Indeed, we further found that APPswe reduces basal autophagy in N2a and this is rescued by MCU ablation measured as percentage of autolysosomes to the total number of LC3 positive structures in the resting condition using tandem fluorescent-tagged LC3 (mRFP-EGFP-LC3) (**Figure 5 N-O, S5J-L**). Autophagic capacity can be impaired by the accumulation of damaged mitochondria thus creating a potential bottleneck in the clearance of amyloid and damaged material (Nichenko et al., 2020; Call & Nichenko, 2020). Indeed, autophagic capacity was previously reported to be reduced by mitochondrial dysfunction and excessive ROS generation (Vicente Roca-Agujetas, 2019). Since mitochondrial dysfunction is reduced by the loss of MCU our results provide a direct link between reduced mCa2+ uptake and neuronal protection against AD pathology.”

Figure S5

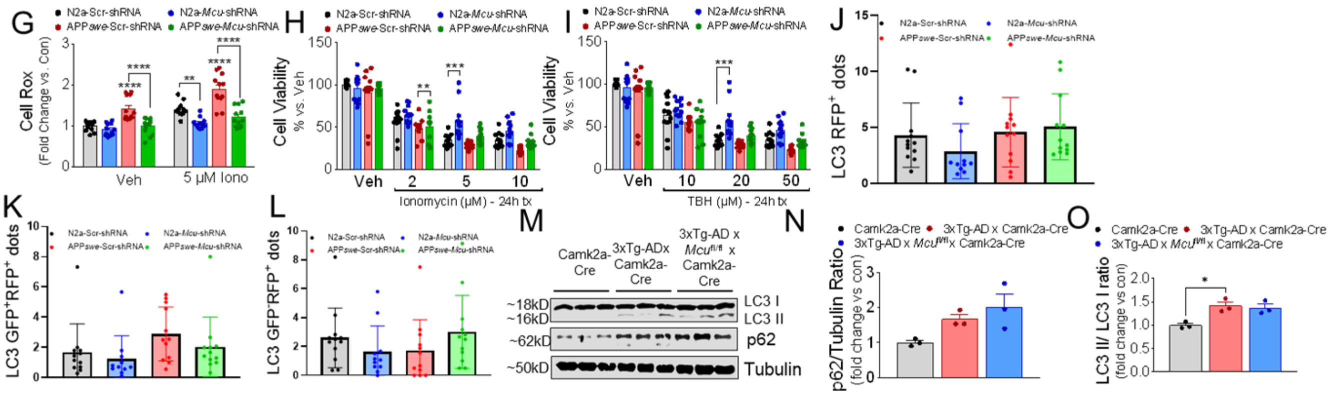

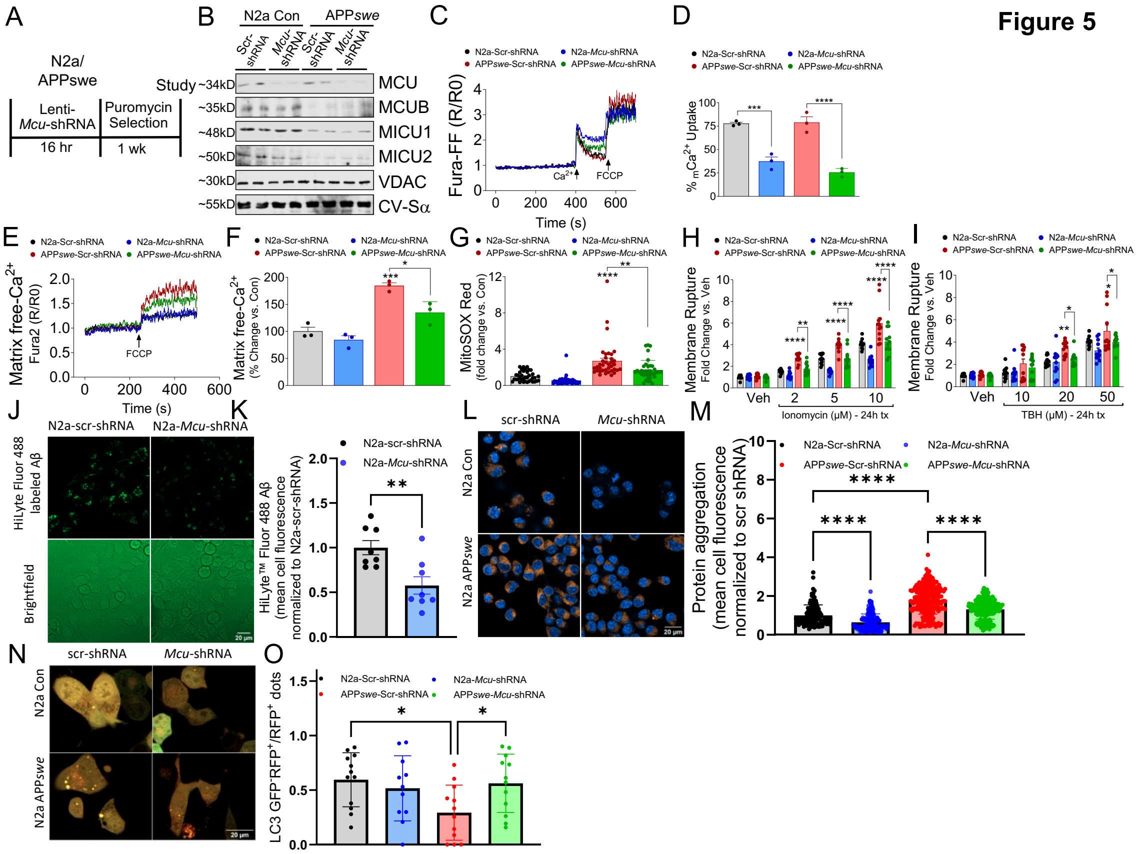
Figure 5

3. Similarly, the authors claim that MCU deletion prevents mitochondrial dysfunction in the neuroblastoma cell line APPswe. To strengthen their finding, they should test additional parameters of mitochondrial function such as ATP production and oxygen consumption rates. They can extend these experiments using mitochondrial isolates from the AD mouse model after MCU deletion and their age-matched controls.

As suggested by the reviewer, we monitored the cells for changes in OxPhos by measuring mitochondrial oxygen consumption rates (OCR) in a Seahorse assay (**Figure S5 T-W**). The stable expression of mutant APPswe elicited a significant decrease in basal respiration and ATP-linked respiration, however, this was not significantly rescued by the loss of MCU (**Figure S5 T-W**). This result might be explained by a smaller role for mitochondrial calcium uptake in mitochondrial bioenergetics in immature neurons that mostly rely on glycolysis and not oxidative phosphorylation. In support of this, APPswe mutation in N2a cells seems to have a more pronounced effect on glycolysis measured as extracellular acidification rate (ECAR) when compared to OCR (**Figure S5U**). Further, given our new results, it's likely that the autophagic capacity mechanism is likely more important for the observed neuronal protection.

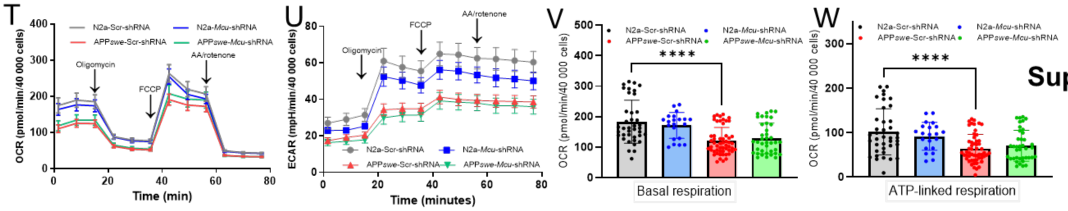

Supplementary Figure 5

4. The fact that AD is associated with mitochondrial dysfunction and defective mitophagy (Kerr, J. S. et al., 2017; Trends Neurosci. 40, 151-166; Lustbader, J. W. et al. 2004, Science 304, 448-452; Fang et al., 2019, Nat. Neuroscience, vol. 22, 401-412) and the finding that loss of mCa2+ uptake prevents AD-associated mitochondrial dysfunction, suggests that the authors should investigate the possible crosstalk between MCU-dependent Ca2+ uptake and mitophagy in AD.

Thank you for this suggestion. Since the loss of MCU and mitochondrial calcium uptake rescues the overall autophagic capacity (see our detailed response above), we believe that mitophagy is most likely affected as well. In addition, another study suggests that mitophagic signaling may be improved with loss of MCU and we have added this to the discussion. "In line with this, in a recent study MCU knockdown using shRNA in hippocampal neurons in 3xTg-AD mice improved mitophagic signaling (Cai H, 2022)."

Other minor comments

5. Some controls are missing in several experiments. For example, quantification of the integrated optical density area of the AT8 and AT180 in CamK2a-Cre mice in Figures 3G and H. Similarly, the data for CamK2a-Cre mice are missing in Figures 4A-4F.

For Figures 3G and H, we did not include wild-type controls for AT8 and AT180 staining in CamK2a-Cre mice because Western blot analysis (**Figure 3A**) shows that these controls do not exhibit immunoreactivity for AT8 and AT180. As a result, we did not add further control data for IHC since the staining was completely absent. (i.e., There is zero expression in non-AD mice.)

Similarly, in Figures 4A-4F, the staining in control mice was absent under the same conditions, and including additional controls did not provide new insights. Our focus in these experiments was to compare AD mice and AD mice with MCU deletion to evaluate the effects of MCU deletion on AD pathology. We believe this approach maintains the clarity of our comparative analysis and aligns with

the goal of highlighting the effects of MCU deletion on AD pathology. We included extensive controls in all experiments.

6. Some experimental issues need to be addressed. For example, the authors do not find microglial activation in the cortex of 3xTg-AD x McuFl/Fl x Camk2a-Cre mice. Since all other parameters of mitochondrial function were tested in both the cortex and the hippocampus, the authors should also examine whether microglia are activated in the hippocampus.

As suggested, we have examined microglia markers in the hippocampus of the AD mice model of the same age group. GFAP expression was significantly decreased while IBA1 expression was unchanged in hippocampus homogenates of 3xTg-AD x *Mcu*$^{fl/fl}$ x Camk2a-Cre, when compared to 3xTg-AD mice (**Figure 4I; S4 E-F**). This suggests that astrocyte mediated inflammatory response is rescued; however, microglia mediated inflammation was not changed in the hippocampus region of the AD mice brain with MCU inhibition. We imagine these results are secondary to preserving neuronal mitochondrial dysfunction.

Fig.4

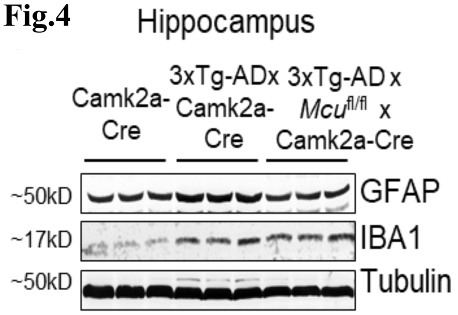

Supp. Fig.4

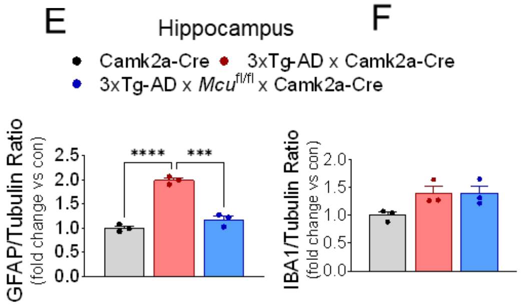

7. Page 7, Fig.1G: The authors state that "loss of neuronal MCU significantly improved age-associated impairments". From Fig.1G, one could say that there is an improvement on AD-associated impairments, rather age.

We have revised the text as suggested.

8. Page 7, end of the paragraph: Could you please provide the relevant WB for loss of MCU in wild type mice (Mcufl/fl x Camk2a-Cre)? The authors discuss that loss of MCU in wild type animals did not result in cognitive impairment in Y-maze testing but do not provide the relevant WB.

The relevant WB with MCU expression has been incorporated. MCU expression was significantly down in the Cre-control background (*Mcu*$^{fl/fl}$ x Camk2a-Cre), further validating the experimental model (**Figure S1 O-P**)

Supp. Fig. 1

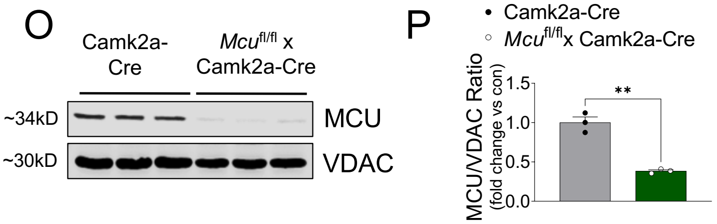

9. Page 9: The authors stated that "3xTg-AD x Mcufl/fl x Camk2a-Cre mice displayed a significant reduction in insoluble tau". However, this doesn't seem to be the case in the WB of the 2nd cohort

(Fig.S3A). There, it seems as it is reduced but there is no quantification. How do these results, if quantified, affect the overall finding?

We carefully quantified the protein expression from both cohorts and affirm here that the HT7 insoluble fraction is significantly reduced with MCU neuronal deletion. Similarly, AT8, AT180 and PHF13 levels are reduced with neuronal MCU deletion in AD mice, but not much change is observed for AT270 **(Figure 3 B-F; S3 B).**

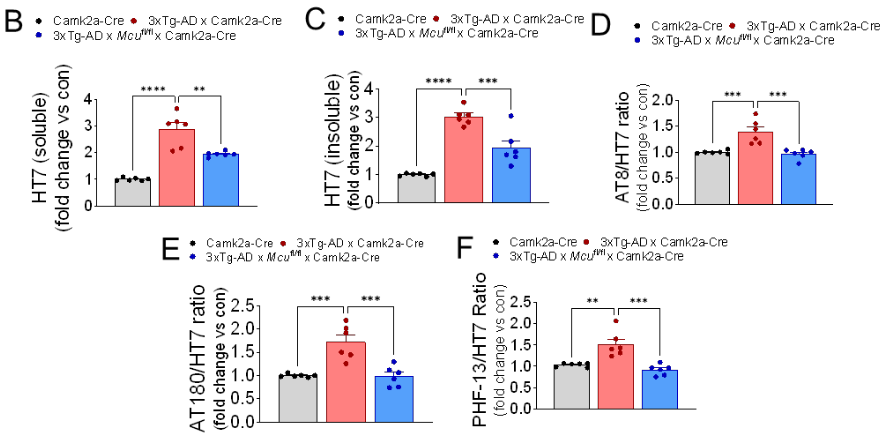

Supp. Fig.3

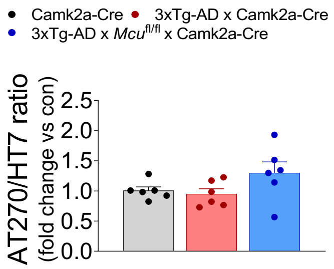

10. Page 10: Regarding the TEM imaging of mitochondria, could you please comment on the fact that there no changes in mitochondria? Mitochondria dysfunction, as the authors mentioned, is commonly found in AD models so I am a bit surprise there is no difference between wild and AD animals. Perhaps not all factors but for instance the circularity or the density.

As requested by Reviewer #2, we reanalyzed the ultrastructural data and included the quantification of ER-mitochondria contact sites (MERCs) and cristae density**.** We observed significant changes in cristae density and the distance between mitochondria and endoplasmic reticulum contacts (MERC). Specifically, cristae density was increased in 3xTg-AD x *Mcu*$^{fl/fl}$ x Camk2a-Cre mice compared to 3xTg-AD mice. Additionally, the mean MERC length was reduced in 3xTg-AD x *Mcu*$^{fl/fl}$ x Camk2a-Cre mice compared to 3xTg-AD mice. See new figures below. These data fit the overall hypothesis and suggest decreased mitochondrial calcium uptake preserves mitochondrial structure and apposition.

Figure 4.

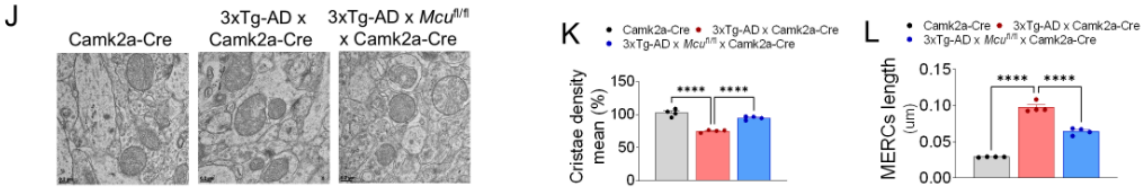

11. Page 12: Is the sentence "To examine redox status in 3xTg-AD mice" a typo? At this stage, authors examine ROS levels in 2A and APPswe cells.

We have fixed this error.

12. Figure 3A, E and S3A: The normalized quantifications of PHF-13 in 1E seem not to correspond to the density of the bands of the two cohorts. At least two values of the 3xTg-AD x Mcufl/fl x Camk2a-Cre mice appear below the Camk2a-Cre control in 1E, which does not seem to be the case in any

blot. Moreover. there is some apparently non-specific signal in the PHF-13 blot of the second cohort 3xTg-AD x Mcufl/fl x Camk2a-Cre samples (S3A).

We have carefully re-analyzed band density**.** The quantification of expression ratio is show below **(new Figure 3 F)**.

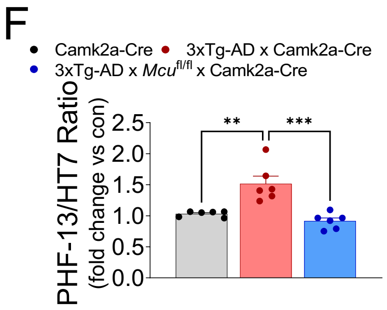

13. Figure 3F-H: Authors should also validate the rest of the WB results (AT270 and PHF-13) with the respective immunohistochemical assay as performed for AT8 and AT180 if the same or similar antibodies allow such an application.

As requested, we performed immunohistochemical assays for AT270 and PHF-13. There was a reduction in PHF-13 tau phosphorylation (~45%) in *Mcu*-deleted 3xTg-AD with no observed change in AT270 (**Figure 3 G-J, 3S C-D**). *Figures below.

Fig.3

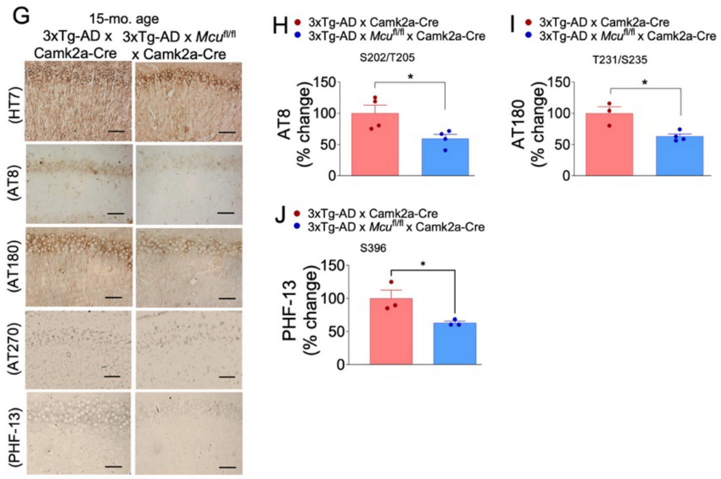

Supp. Fig.3

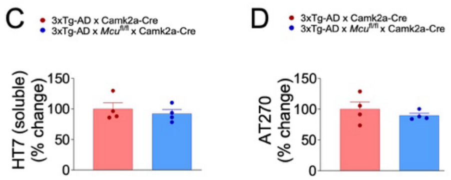

14. Figure S4 legend: Include mitochondrial number per area description in the legend (S4F) and correct the following letters from F to L.

This has been edited.

15. As authors mention, predisposition towards increased mCa2+ uptake and overload due to decreased expression of the mtCU regulators MICU1, MICU2 and MCUB is possible in APPswe cells. Thus, the results derived from these cells (Ca2+ uptake, plasma membrane rapture and cellular viability) have to be carefully interpreted. This needs to be explicitly mentioned in the discussion as well.

This is discussed in detail in the revised discussion. *“Mitochondrial matrix calcium can be increased*

by decreasing NCLX-dependent $_mCa^{2+}$ efflux or reducing the threshold for mtCU Ca^{2+} uptake, e.g. by decreasing MICU1 and MICU2 protein levels or reducing MCUB's inhibitory activity on the mtCU complex. These molecular phenomena were observed in both in vivo and cellular AD models (Jadiya et al., 2019), suggesting that expression changes in mtCU regulators may be an early adaptive response to augment cellular energetics during stress, which subsequently becomes maladaptive and results in $_mCa^{2+}$ overload, mitochondrial dysfunction, and oxidative stress creating a bottleneck in autophagic clearance of damaged cell material (Nichenko et al., 2020; Call & Nichenko, 2020)."

16. While membrane rapture is decreased in Mcu knockdown APPswe cells in almost all tested conditions (Figure 5J, K), cellular viability is not enhanced upon Mcu knockdown at APPswe cells as authors claim (Figure S5F-G). Only in one case (2μM ionomycin), the viability is significantly enhanced (where the point distribution seems not to support it; please, double-check the statistics). The authors should reconsider their respective conclusions.

This is a good catch by the reviewer. We have restated our conclusions: "We observed a significant decrease in membrane rupture, a characteristic of necrotic cell death, and tendency toward enhanced cellular viability in *Mcu* knockdown APPswe cell lines (Figure 5 H-I, S5 H-I)." We should note that membrane rupture is a better indicator of cell death, as all available reagents to monitor cell viability are general indicators of metabolic health and thus are indirect indicators of actual cell viability.

17. In general, a substantial amount of data is derived from WBs and associated densitometry. However, in some cases, the blot quality is not optimal (bubbles, progressive fainting, background, etc., examples: 1B-MICU3, 3A-HT7/AT180, 4G-PSD-95, 4H-GFAP, 5B-CV-Sα, S2C-PS1/NCT, S3A-PHF-13) which most likely affects the densitometric analysis, as well.

We appreciate your feedback regarding the quality of certain Western blots and the potential impact on densitometric analysis. To address this, we have included all raw Western blot images in the Supplementary Material for transparency. We conducted the densitometric analysis with great care, utilizing software tools specifically designed to minimize the impact of background noise, etc. Additionally, we have cross-validated the results with alternative methods wherever possible to ensure the accuracy and reliability of our findings. We hope the editors agree that our study is rigorous.

18. Figure 1 panels: Perhaps, change the order of plots I, J and K in figure 1 to match the order commented in the main text (p7, towards the end of the section). For instance, Fig.1I is commented in the manuscript after J and K.

Changed as requested.

19. I am sorry, but I have not been able to find Fig. 2H.

Apologies, we have fixed this error.

20. Please, mention Figure 5A in the text.

We have fixed this issue.

Dear John,

Thank you again for the submission of your revised manuscript (EMBOJ-2023-115858R) to The EMBO Journal for our consideration, and for your patience during re-review. Your revised manuscript has now been seen by the three referees who had also assessed the previous version of your manuscript, and we have received their comments, which you can find appended below.

I am pleased to say that all three referees recognize that the revised version of the study is significantly improved. Referees #2 and #3 find the revision and your responses to the previously raised concerns satisfactory and now recommend publication without any further comments. Referee #1, on the other hand, has two remaining points, which we kindly request you address in a final version of the manuscript, either by providing further experimental evidence, as suggested by the referee, or by appropriately revising the text. While we find the referees' suggestions relevant and we think that the additional data they suggest would strengthen the manuscript, we will not require new experiments at this stage. However, we request that the points of the referee be taken into consideration carefully and addressed either experimentally or textually in a final version of the manuscript.

Please include in your revision a point-by-point response to the referee's comments with detailed explanations and a description of any changes to the manuscript.

From the editorial side, there are also a few changes we need you to make in the final version of your manuscript, before we can move forward with its processing:

- Please note that our notifications to the e-mail address manfred@temple.edu of your co-author Manfred Thomas could not be delivered. Please make sure that the profile of your co-author is updated with the current/valid e-mail address, or send us the current/new e-mail address so that we can update the account on your co-author's behalf.

- Please make sure that the funding information provided in the Acknowledgements section of the manuscript matches the information entered in our online manuscript handling system. The following grants seem to be currently missing in the online system: NIH grants: R01HL136954, R01HL142271, 3R01HL123966-05S1; NIH K99AG065445, Alzheimer's Association 24AARG-D-1191292 , American Heart Association 24IPA1273195, Wake ADRC REC and Development grant (P30AG072947), NIH K99DK120876 and NIH F32HL151146.

- Please note that the Data Availability section is restricted to new primary datasets that are part of this study and require deposition in structured databases/repositories (such as DNA/RNA sequencing or mass spectrometry data). Your study appears not to include such data - if this is the case, please state so in the Data Availability section: "Our study includes no data deposited in public repositories.". The uploaded Source Data for your Figures, which will become available alongside the article, do not need to be mentioned in this section.

- We noticed that the manuscript contains two conflict-of-interest statements (on the title and the last page). Please avoid this redundancy and include only one such statement in the revised manuscript, with title "Disclosure and competing interests statement", placed before the References list.

- The author contributions statement should be removed from the manuscript file. Instead, we use CRediT to specify the contributions of each author in the journal submission system. Please feel free to use the free text box to provide more detailed descriptions during submission. See also our guide to authors for more information: https://link.springer.com/partners/embo-press/editorial-policies#Authorship.

- Please upload your Figures in individual high-quality production-quality files (one file per Figure). You can find more information on how to prepare and upload your Figures in our author guidelines: https://link.springer.com/journal/44318/submission-guidelines#cms-Figure-and-data-presentation. Please note that our team will have to check the resolution of the individual files before we can move forward with the processing of your manuscript.

- The title page of the Appendix PDF file should include heading "Appendix for:" followed by the manuscript's title and a Table of Contents including page numbers for all listed items. Please remove "Supplementary Information". The nomenclature for the Appendix items must be "Appendix Figure Sx" and "Appendix Table Sx" throughout the Appendix and the callouts in the manuscript.

- "Supplementary Information" should be removed from the manuscript and the Appendix file.

- Thank you for uploading the Source Data for your Figures. We kindly request that you re-organize/re-upload the Source Data for the main Figures in one zip folder (named, for example, "Source Data for Figure 1") per Figure.

- Please note that EMBO press papers are accompanied online by:
A) a short (2 sentences) summary of the findings and their significance,
B) 2-5 short bullet points highlighting the key results, and
C) a synopsis image in .jpg or .png format that is exactly 550 pixels wide and 300-600 pixels high (the height is variable). Please note that all text needs to be legible at the final size.
Please upload this information along with your revised manuscript (the text for A and B should be provided in a separate Word file).

- Our data editors have checked your Figures and their legends, and raised the following queries. Please address all points below completely in your revised manuscript (all changes should be highlighted or "tracked" in the revised manuscript):
1. Please note that the legend for Figure S3 F-J is missing in the manuscript. This needs to be rectified.
2. Please define the annotated p values ****/***/**/* as well as provide the exact p-values for the same in the legends of Figure 3B, C, D, E, F, H, I, J as appropriate.
3. Please note that the exact p-values must be provided in the legends of Figures 1E, F, J, K; 2A, B, C, D, F; 4B, C, E, F, K, L; 5D, F, G, H, I, K, M, O; S1 A-N, P-U; S2 D, F, G, H, I, K, L; S3 F, G; S4A-F; S5 A-D, F, G, H, I, O, Q, V, W.
4. Please indicate the statistical test used for data analysis in the legends of Figures 3B, C, D, E, F, H, I, J.
5. Please note that information related to "n" is missing in the legends of Figures 1G-K; 2A-D; 4D, K, L; 5F, G, H, I, K, M, O; S1 A-N, P-U; S2 A, B, D-M; S3 F-J, M-P; S4G-L; S5A-L, Q-W.
6. Please note that the error bars should be defined in the legends of Figures 3B, C, D, E, F, H, I, J.

- The manuscript sections need to be named and ordered as follows: Title page - Abstract - Introduction - Results - Discussion - Methods - Data Availability - Acknowledgements - Disclosure and Competing Interests Statement - References - Figure Legends - main Tables (if there are any) - Expanded View Figure Legends.

- Please also note that as part of the EMBO Press transparent editorial process, The EMBO Journal publishes online a Peer Review File along with each accepted manuscript. This File will be published in conjunction with your paper and will include the referee reports, your point-by-point responses and all pertinent correspondence relating to the manuscript. Your Author's Checklist will also be published at the end of the Peer Review File. Please let us know in case you want to remove any data or figures from your point-by-point responses before they are published as part of the Peer Review File. Retaining unpublished data in the Peer Review File means that these count as published and that the Peer Review File would need to be referenced in future publications. Please let the editorial office know in case you want to remove any data from this file (contact@embojournal.org).

We look forward to seeing a final version of your manuscript as soon as possible. Please let us know if you have any questions and use this link to submit your revision: https://emboj.msubmit.net/cgi-bin/main.plex.

Best regards,

Ioannis

Ioannis Papaioannou, PhD
Editor, The EMBO Journal
i.papaioannou@embojournal.org

Referee #1:

Summary: The authors studied the role of the mitochondrial calcium uniporter (MCU) in a mouse model of Alzheimer's disease. The authors report that MCU inhibition rescues calcium signaling and improves behavioral deficits. Furthermore, MCU inhibition improve soluble and insoluble Abeta levels in aged mice. The authors suggest that autophagy may play a role in the clearance of protein aggregates and secondary inflammation.

The authors have made a clear effort to address my concerns and have generated additional data that support their conclusion. I appreciate their efforts.

I would like to raise two points for the authors' consideration:

First, the authors report that MCU loss reduces Aβ accumulation and that APPswe expression inhibits basal autophagy in N2a neurons, an effect that is rescued by MCU ablation. However, they also state that autophagy markers (LC3-II/LC3-I and p62) are not differentially regulated in protein lysates from the frontal cortex of 15-month-old mice. If this is the case, the N2a cell model may not accurately recapitulate the in vivo findings, which would weaken the authors' conclusions. That said, a closer look of Figures S5M-O suggests changes (some statistically different, others with a trend) in p62 expression levels and in the LC3-II/LC3-I ratio. Therefore, I would encourage the authors to re-evaluate these data and/or to consider providing additional evidence that more directly assesses autophagic flux in vivo.

Second, several studies (four recent publications, one from John Elrod's group) have identified TMEM65 as an important regulator of mitochondrial calcium signaling. It would strengthen the manuscript if the authors could complete their experimental panel by including an immunoblot analysis of TMEM65, although I recognize that the availability of well-validated antibodies may be a limitation. Moreover, in light of two recent manuscript (PMID: 40691517 and PMID: 40931067) demonstrating that TMEM65 functions as a mitochondrial Na^{+}/Ca^{2+} exchanger, whereas NCLX acts as an H^{+}/Ca^{2+} exchanger, I kindly suggest that the authors revise some sections of the manuscript (pages 4, 17, and 39) to reflect these novel findings.

Referee #2:

The authors have satisfactorily addressed all the points raised in the initial round of review. I have no further suggestions for change and congratulate them for carrying on the additional experiments suggested.

Referee #3:

The authors have responded adequately to my comments and suggestions, both in the rebuttal letter and by appropriately revising their manuscript. The study has now been improved substantially and can be considered for publication.

Point-by-Point Response to Critique

We thank all three reviewers for their input during the revision process. Please find reviewer comments summarized below in addition to point-by-point responses.

Referee #1:

Summary: The authors studied the role of the mitochondrial calcium uniporter (MCU) in a mouse model of Alzheimer's disease. The authors report that MCU inhibition rescues calcium signaling and improves behavioral deficits. Furthermore, MCU inhibition improve soluble and insoluble Abeta levels in aged mice. The authors suggest that autophagy may play a role in the clearance of protein aggregates and secondary inflammation.

The authors have made a clear effort to address my concerns and have generated additional data that support their conclusion. I appreciate their efforts.

We thank the reviewer for their thoughtful and critical evaluation of our revised manuscript.

I would like to raise two points for the authors' consideration:
First, the authors report that MCU loss reduces Aβ accumulation and that APPswe expression inhibits basal autophagy in N2a neurons, an effect that is rescued by MCU ablation. However, they also state that autophagy markers (LC3-II/LC3-I and p62) are not differentially regulated in protein lysates from the frontal cortex of 15-month-old mice. If this is the case, the N2a cell model may not accurately recapitulate the in vivo findings, which would weaken the authors' conclusions. That said, a closer look of Figures S5M-O suggests changes (some statistically different, others with a trend) in p62 expression levels and in the LC3-II/LC3-I ratio. Therefore, I would encourage the authors to re-evaluate these data and/or to consider providing additional evidence that more directly assesses autophagic flux in vivo.

We thank the reviewer for this insight and agree that there are differences between the *in vivo* and *in vitro* models that were utilized in this study. We agree with the reviewer that the data presented regarding autophagic flux (expression of autophagy markers LC3-II/LC3-I and p62) in the *in vivo* model (cre-driven deletion of MCU from Camk2a-cre expressing neurons in the hippocampus) demonstrates mixed statistical significant difference (specifically in Figures S5M-O). We believe this approach enables detection of increased autophagy resulting from the enhanced cellular burden associated with AD pathology, which likely impacts multiple cell types. However, neuron-specific deletion of MCU may restore basal autophagic capacity only within neurons (i.e., the in vivo signal and readouts for autophagy include numerous other cell types that are not targeted for loss of MCU). Furthermore, in our *in vitro* model, MCU deletion does not alter overall autophagic flux. Altogether it makes it difficult to assess equivalent parameters *in vivo* through protein expression analysis alone. Measuring basal neuronal autophagic capacity in vivo would require time- and cost-intensive viral delivery approaches and experiments in live tissue slices — efforts that fall beyond the current scope of this study but should be pursued in future work.

Second, several studies (four recent publications, one from John Elrod's group) have identified TMEM65 as an important regulator of mitochondrial calcium signaling. It would strengthen the manuscript if the authors could complete their experimental panel by including an immunoblot analysis of TMEM65, although I recognize that the availability of well-validated antibodies may be a limitation. Moreover, in light of two recent manuscript (PMID: 40691517 and PMID: 40931067) demonstrating that TMEM65 functions as a mitochondrial Na^+/Ca^{2+} exchanger,

whereas NCLX acts as an H^+/Ca^{2+} exchanger, I kindly suggest that the authors revise some sections of the manuscript (pages 4, 17, and 39) to reflect these novel findings.

We thank the reviewer for their thorough review of our ongoing work. The studies referenced from our group apply similar experimental paradigms to interrogate NCLX, the primary Ca^{2+} efflux pathway, in genetic models of AD (PMID: 31467276) . The more recent of the two publications identify TMEM65 as a critical regulator of NCLX activity (PMID: 40200126). Thus, we agree with the reviewer that there is strong rationale to interrogate TMEM65 within the context of neurodegeneration and neurodegenerative diseases. Indeed, this is an area of ongoing work in our group. Even so, we believe including these data would be distracting to the reader given that the primary pathway of interest in our study is Ca^{2+} uptake into the mitochondria by the uniporter. Additionally, the studies by other groups regarding the fundamental function of TMEM65, as a regulator or standalone transporter of Ca^{2+}, remains controversial. Therefore, we have decided not to include this data in our manuscript.

Referee #2:

The authors have satisfactorily addressed all the points raised in the initial round of review. I have no further suggestions for change and congratulate them for carrying on the additional experiments suggested.

We thank the reviewer for their feedback and contributions to our manuscript throughout the review process.

Referee #3:

The authors have responded adequately to my comments and suggestions, both in the rebuttal letter and by appropriately revising their manuscript. The study has now been improved substantially and can be considered for publication.

We thank the reviewer for their feedback and contributions to our manuscript throughout the review process.

2nd Revision - Editorial Decision 27th Apr 2026

Dear John,

Congratulations on an excellent work! I am very pleased to inform you that your manuscript has been accepted for publication in The EMBO Journal. Thank you for comprehensively addressing the initially raised referee concerns and all editorial requests for corrections and changes.

Before we can proceed with the next steps towards publication of your article, I would kindly request that you send me a final version of the main manuscript file, in Word format, textually addressing the last two concerns/suggestions of referee #1, in the Discussion. While we recognize that you have already responded to these comments in your point-by-point response, we request that they also be addressed in the Discussion of the manuscript, which should transparently cover limitations of the work and provide suggestions for future research. Please make sure that any additions/changes in the Discussion are highlighted in the Word file, and that no other changes are introduced in the manuscript.

Your manuscript will then be copy edited and you will receive page proofs prior to publication. Please note that you will be contacted by Springer Nature Author Services to complete licensing and payment information.

You may qualify for financial assistance for your publication charges - either via a Springer Nature fully open access agreement or an EMBO initiative. Check your eligibility: https://link.springer.com/journal/44318/how-to-publish-with-us

Should you be planning a Press Release on your article, please get in contact with embo_production@springernature.com as early as possible in order to coordinate publication and release dates.

If you have any questions, please do not hesitate to contact the Editorial Office. Thank you for your contribution to The EMBO Journal. Working with you has been a pleasure.

Best regards,

Ioannis

Ioannis Papaioannou, PhD
Editor, The EMBO Journal
i.papaioannou@embojournal.org

*****
Please note that it is The EMBO Journal policy for the transcript of the editorial process (containing referee reports and your response letters) to be published as an online supplement to each paper. If you should prefer removal of any referee-only figures included in the point-by-point response(s), e.g. because they may still be used for future publication or because they have been reproduced from published work by others, please do let us know immediately via response email.
More information is available here: https://link.springer.com/partners/embo-press/editorial-policies#Peer%20review

** Click here to be directed to your login page: https://emboj.msubmit.net

EMBO Press Author Checklist

Corresponding Author Name: John W. Elrod
Journal Submitted to: The EMBO Journal
Manuscript Number: EMBOJ-2023-115858

USEFUL LINKS FOR COMPLETING THIS FORM
The EMBO Journal - Author Guidelines
EMBO Reports - Author Guidelines
Molecular Systems Biology - Author Guidelines
EMBO Molecular Medicine - Author Guidelines

Reporting Checklist for Life Science Articles (updated January
This checklist is adapted from Materials Design Analysis Reporting (MDAR) Checklist for Authors. MDAR establishes a minimum set of requirements in transparent reporting in the life sciences (see Statement of Task: 10.31222/osf.io/9sm4x). Please follow the journal's guidelines in preparing your
Please note that a copy of this checklist will be published alongside your article.

Abridged guidelines for figures
1. Data
The data shown in figures should satisfy the following conditions:
- the data were obtained and processed according to the field's best practice and are presented to reflect the results of the experiments in an accurate and unbiased manner.
- ideally, figure panels should include only measurements that are directly comparable to each other and obtained with the same assay.
- plots include clearly labeled error bars for independent experiments and sample sizes. Unless justified, error bars should not be shown for technical
- if n<5, the individual data points from each experiment should be plotted. Any statistical test employed should be justified.
- Source Data should be included to report the data underlying figures according to the guidelines set out in the authorship guidelines on Data

2. Captions
Each figure caption should contain the following information, for each panel where they are relevant:
- a specification of the experimental system investigated (eg cell line, species name).
- the assay(s) and method(s) used to carry out the reported observations and measurements.
- an explicit mention of the biological and chemical entity(ies) that are being measured.
- an explicit mention of the biological and chemical entity(ies) that are altered/varied/perturbed in a controlled manner.
- the exact sample size (n) for each experimental group/condition, given as a number, not a range;
- a description of the sample collection allowing the reader to understand whether the samples represent technical or biological replicates (including how many animals, litters, cultures, etc.).
- a statement of how many times the experiment shown was independently replicated in the laboratory.
- definitions of statistical methods and measures:
  - common tests, such as t-test (please specify whether paired vs. unpaired), simple χ2 tests, Wilcoxon and Mann-Whitney tests, can be unambiguously identified by name only, but more complex techniques should be described in the methods section;
  - are tests one-sided or two-sided?
  - are there adjustments for multiple comparisons?
  - exact statistical test results, e.g., P values = x but not P values < x;
  - definition of 'center values' as median or average;
  - definition of error bars as s.d. or s.e.m.

Please complete ALL of the questions below.
Select "Not Applicable" only when the requested information is not relevant for your study.

Materials

Newly Created Materials	Information included in the manuscript?	In which section is the information available? (Reagents and Tools Table, Materials and Methods, Figures, Data Availability Section)
New materials and reagents need to be available; do any restrictions apply?	Yes	Section: Materials and Methods, Figures; Restrictions: None reported; materials available upon request

Antibodies	Information included in the manuscript?	In which section is the information available? (Reagents and Tools Table, Materials and Methods, Figures, Data Availability Section)
For **antibodies** provide the following information:<br>- Commercial antibodies: RRID (if possible) or supplier name, catalogue number and or/clone number<br>- Non-commercial: RRID or citation	Yes	Materials and Methods

DNA and RNA sequences	Information included in the manuscript?	In which section is the information available? (Reagents and Tools Table, Materials and Methods, Figures, Data Availability Section)
**Short novel DNA or RNA including primers, probes**: provide the sequences.	Yes	Materials and Methods

Cell materials	Information included in the manuscript?	In which section is the information available? (Reagents and Tools Table, Materials and Methods, Figures, Data Availability Section)
**Cell lines:** Provide species information, strain. Provide accession number in repository **OR** supplier name, catalog number, clone number, and/**OR** RRID.	Yes	Materials and Methods
**Primary cultures:** Provide species, strain, sex of origin, genetic modification status.	Not Applicable	
Report if the cell lines were recently **authenticated** (e.g., by STR profiling) and tested for mycoplasma contamination.	Not Applicable	

Experimental animals	Information included in the manuscript?	In which section is the information available? (Reagents and Tools Table, Materials and Methods, Figures, Data Availability Section)
**Laboratory animals or Model organisms:** Provide species, strain, sex, age, genetic modification status. Provide accession number in repository **OR** supplier name, catalog number, clone number, **OR** RRID.	Yes	Materials and Methods
**Animal observed in or captured from the field:** Provide species, sex, and age where possible.	Yes	Materials and Methods, Figures, Figure Legends
Please detail **housing and husbandry conditions**.	Yes	Materials and Methods

Plants and microbes	Information included in the manuscript?	In which section is the information available? (Reagents and Tools Table, Materials and Methods, Figures, Data Availability Section)
**Plants:** provide species and strain, ecotype and cultivar where relevant, unique accession number if available, and source (including location for collected wild specimens).	Not Applicable	
**Microbes:** provide species and strain, unique accession number if available, and source.	Not Applicable	

Human research participants	Information included in the manuscript?	In which section is the information available? (Reagents and Tools Table, Materials and Methods, Figures, Data Availability Section)
If collected and within the bounds of privacy constraints report on age, sex and gender or ethnicity for all study participants.	Not Applicable	

Core facilities	Information included in the manuscript?	In which section is the information available? (Reagents and Tools Table, Materials and Methods, Figures, Data Availability Section)
If your work benefited from core facilities, was their service mentioned in the acknowledgments section?	Not Applicable	

Design

Study protocol	Information included in the manuscript?	In which section is the information available? (Reagents and Tools Table, Materials and Methods, Figures, Data Availability Section)
If study protocol has been **pre-registered, provide DOI in the manuscript**. For clinical trials, provide the trial registration number **OR** cite DOI.	Not Applicable	
Report the **clinical trial registration number** (at ClinicalTrials.gov or equivalent), where applicable.	Not Applicable	

Laboratory protocol	Information included in the manuscript?	In which section is the information available? (Reagents and Tools Table, Materials and Methods, Figures, Data Availability Section)
Provide DOI OR other citation details if **external detailed step-by-step protocols** are available.	Yes	Materials and Methods

Experimental study design and statistics	Information included in the manuscript?	In which section is the information available? (Reagents and Tools Table, Materials and Methods, Figures, Data Availability Section)
Include a statement about **sample size** estimate even if no statistical methods were used.	Yes	Figure Legends
Were any steps taken to minimize the effects of subjective bias when allocating animals/samples to treatment (e.g. **randomization procedure**)? If yes, have they been described?	Yes	Materials and Methods
Include a statement about **blinding** even if no blinding was done.	Yes	Materials and Methods
Describe **inclusion/exclusion criteria** if samples or animals were excluded from the analysis. Were the criteria pre-established?<br><br>If sample or data points were omitted from analysis, report if this was due to attrition or intentional exclusion and provide justification.	Not Applicable	
For every figure, are **statistical tests** justified as appropriate? Do the data meet the assumptions of the tests (e.g., normal distribution)? Describe any methods used to assess it. Is there an estimate of variation within each group of data? Is the variance similar between the groups that are being statistically compared?	Yes	Materials and Methods and Figure legends

Sample definition and in-laboratory replication	Information included in the manuscript?	In which section is the information available? (Reagents and Tools Table, Materials and Methods, Figures, Data Availability Section)
In the figure legends: state number of times the experiment was **replicated** in laboratory.	Yes	Figure legends
In the figure legends: define whether data describe **technical or biological replicates**.	Yes	Figure legends

Ethics

Ethics	Information included in the manuscript?	In which section is the information available? (Reagents and Tools Table, Materials and Methods, Figures, Data Availability Section)
Studies involving **human participants**: State details of **authority granting ethics approval** (IRB or equivalent committee(s), provide reference number for approval.	Not Applicable	
Studies involving **human participants**: Include a statement confirming that **informed consent** was obtained from all subjects and that the experiments conformed to the principles set out in the WMA Declaration of Helsinki and the Department of Health and Human Services Belmont Report.	Not Applicable	
Studies involving **human participants:** For publication of **patient photos**, include a statement confirming that consent to publish was obtained.	Not Applicable	
Studies involving experimental **animals**: State details of **authority granting ethics approval** (IRB or equivalent committee(s), provide reference number for approval. Include a statement of compliance with ethical regulations.	Not Applicable	
Studies involving **specimen and field samples:** State if relevant **permits** obtained, provide details of authority approving study; if none were required, explain why.	Not Applicable	

Dual Use Research of Concern (DURC)	Information included in the manuscript?	In which section is the information available? (Reagents and Tools Table, Materials and Methods, Figures, Data Availability Section)
Could your study fall under dual use research restrictions? Please check biosecurity documents and list of **select agents and toxins** (CDC): https://www.selectagents.gov/sat/list.htm	Not Applicable	
If you used a select agent, is the security level of the lab appropriate and reported in the manuscript?	Not Applicable	
If a study is subject to dual use research of concern regulations, is the name of the **authority granting approval and reference number** for the regulatory approval provided in the manuscript?	Not Applicable	

Reporting

The MDAR framework recommends adoption of discipline-specific guidelines, established and endorsed through community initiatives. Journals have their own policy about requiring specific guidelines and recommendations to complement MDAR.

Adherence to community standards	Information included in the manuscript?	In which section is the information available? (Reagents and Tools Table, Materials and Methods, Figures, Data Availability Section)
State if relevant guidelines or checklists (e.g., **ICMJE, MIBBI, ARRIVE, PRISMA**) have been followed or provided.	Not Applicable	
For **tumor marker prognostic studies,** we recommend that you follow the **REMARK** reporting guidelines (see link list at top right). See author guidelines, under 'Reporting Guidelines'. Please confirm you have followed these guidelines.	Not Applicable	
For **phase II and III randomized controlled trials**, please refer to the **CONSORT** flow diagram (see link list at top right) and submit the CONSORT checklist (see link list at top right) with your submission. See author guidelines, under 'Reporting Guidelines'. Please confirm you have submitted this list.	Not Applicable	

Data Availability

Data availability	Information included in the manuscript?	In which section is the information available? (Reagents and Tools Table, Materials and Methods, Figures, Data Availability Section)
Have **primary datasets** been deposited according to the journal's guidelines (see 'Data Deposition' section) and the respective accession numbers provided in the Data Availability Section?	Not Applicable	
Were **human clinical and genomic datasets** deposited in a public access-controlled repository in accordance to ethical obligations to the patients and to the applicable consent agreement?	Not Applicable	
Are **computational models** that are central and integral to a study available without restrictions in a machine-readable form? Were the relevant accession numbers or links provided?	Not Applicable	
If publicly available data were reused, provide the respective **data citations in the reference list**.	Not Applicable	
